# Supplementary material for: Rapid Hypermutation B Cell Trajectory Recruits Previously Primed B Cells Upon Third SARS-Cov-2 mRNA Vaccination
Source: Front Immunol. 2022 May 9;13:876306. doi: 10.3389/fimmu.2022.876306 (PMC9126551; doi:10.3389/fimmu.2022.876306)
Supplement: Supplementary file 1 [file DataSheet_1.docx]

Supplementary Material

# Supplementary Figures and Tables

## Supplementary Figures


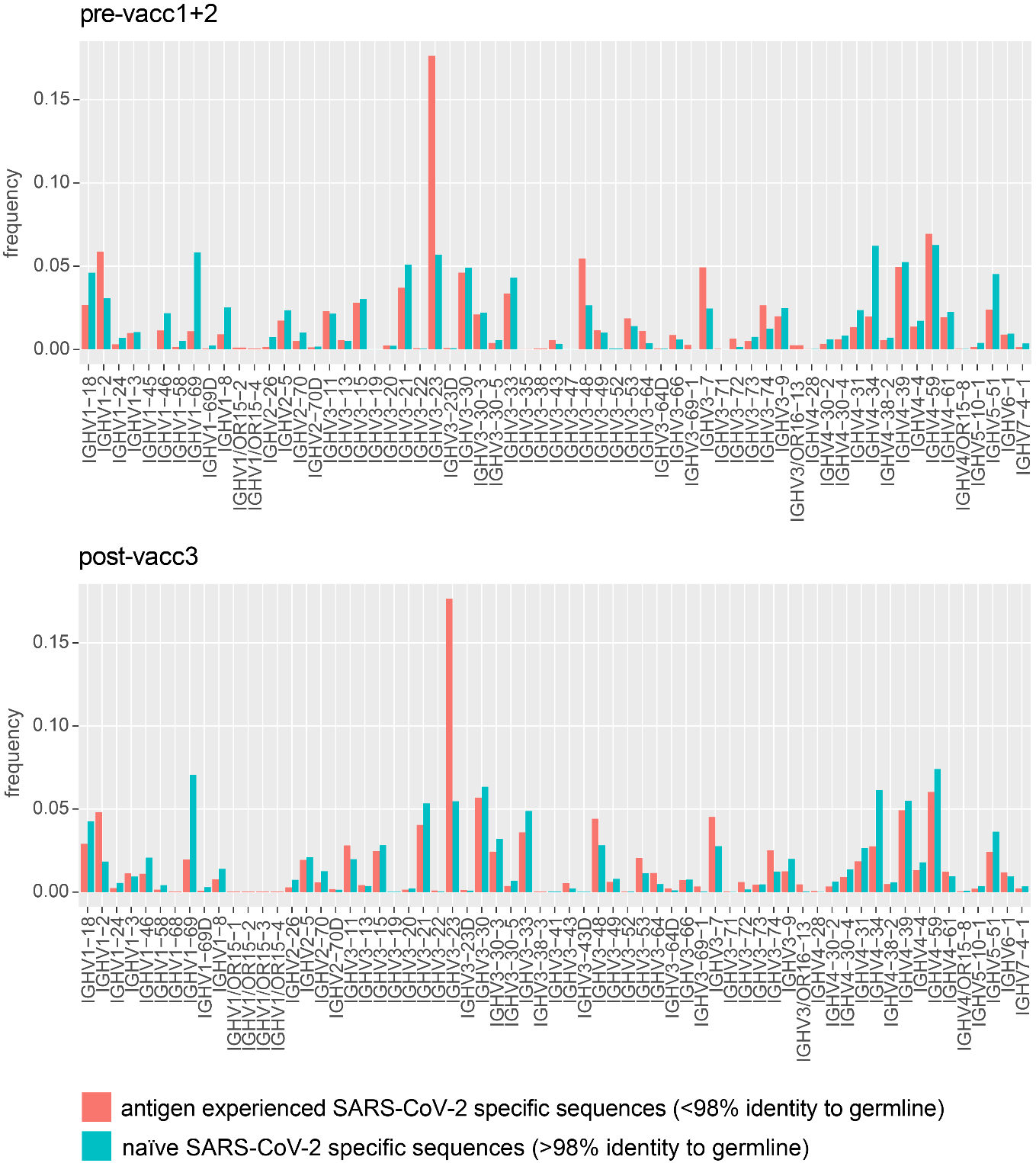


**Supplementary Figure 1. IGHV gene usage in naïve versus antigen-experienced B cell in unselected B cells of unvaccinated repertoires (pre-vacc1+2) and post-vacc3 samples.**


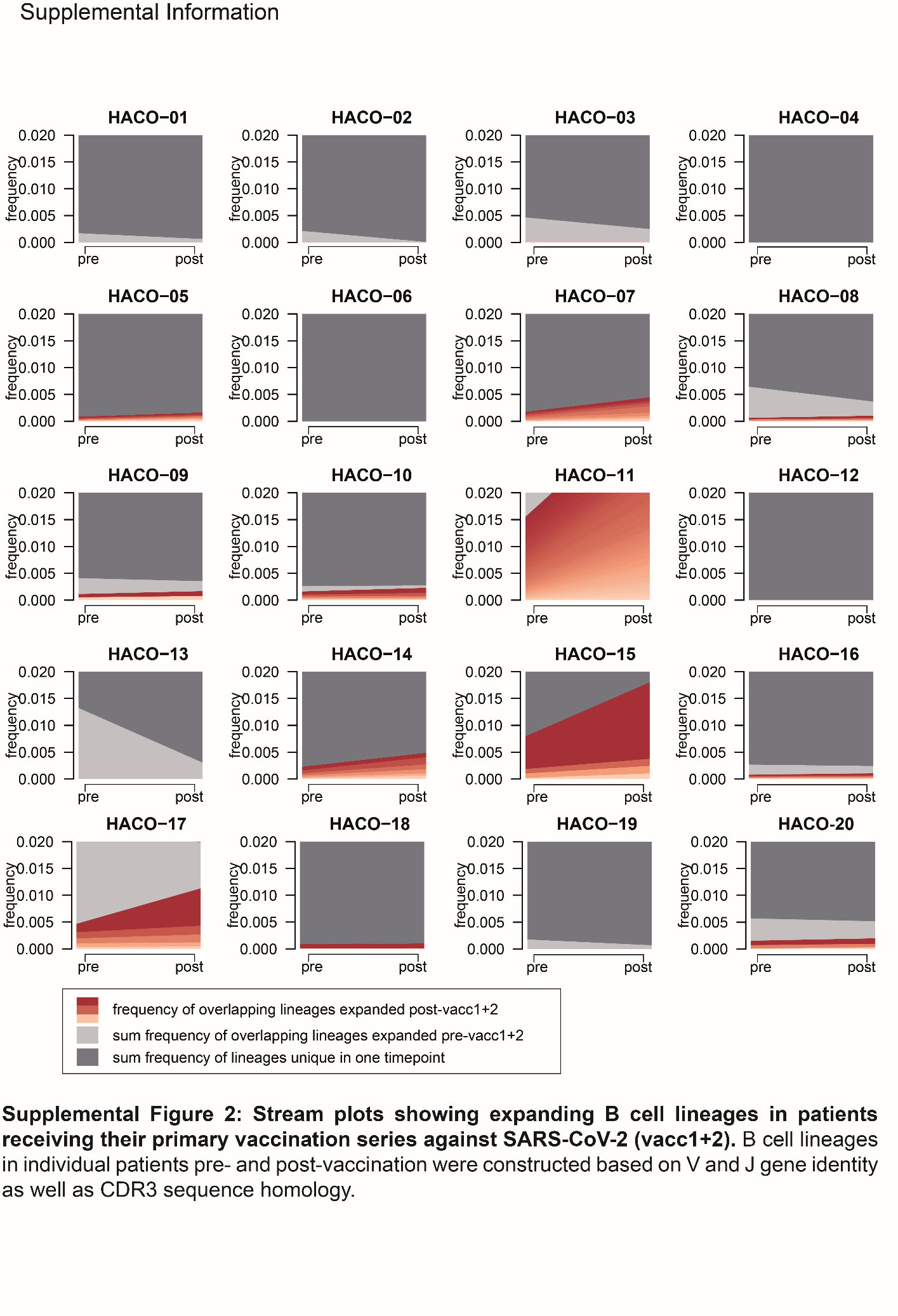


**Supplementary Figure 2.** **Stream plots showing expanding B cell lineages in patients receiving their primary vaccination series against SARS-CoV-2 (vacc1+2).** B cell lineages in individual patients pre- and post-vaccination were constructed based on V and J gene identity as well as CDR3 sequence homology.


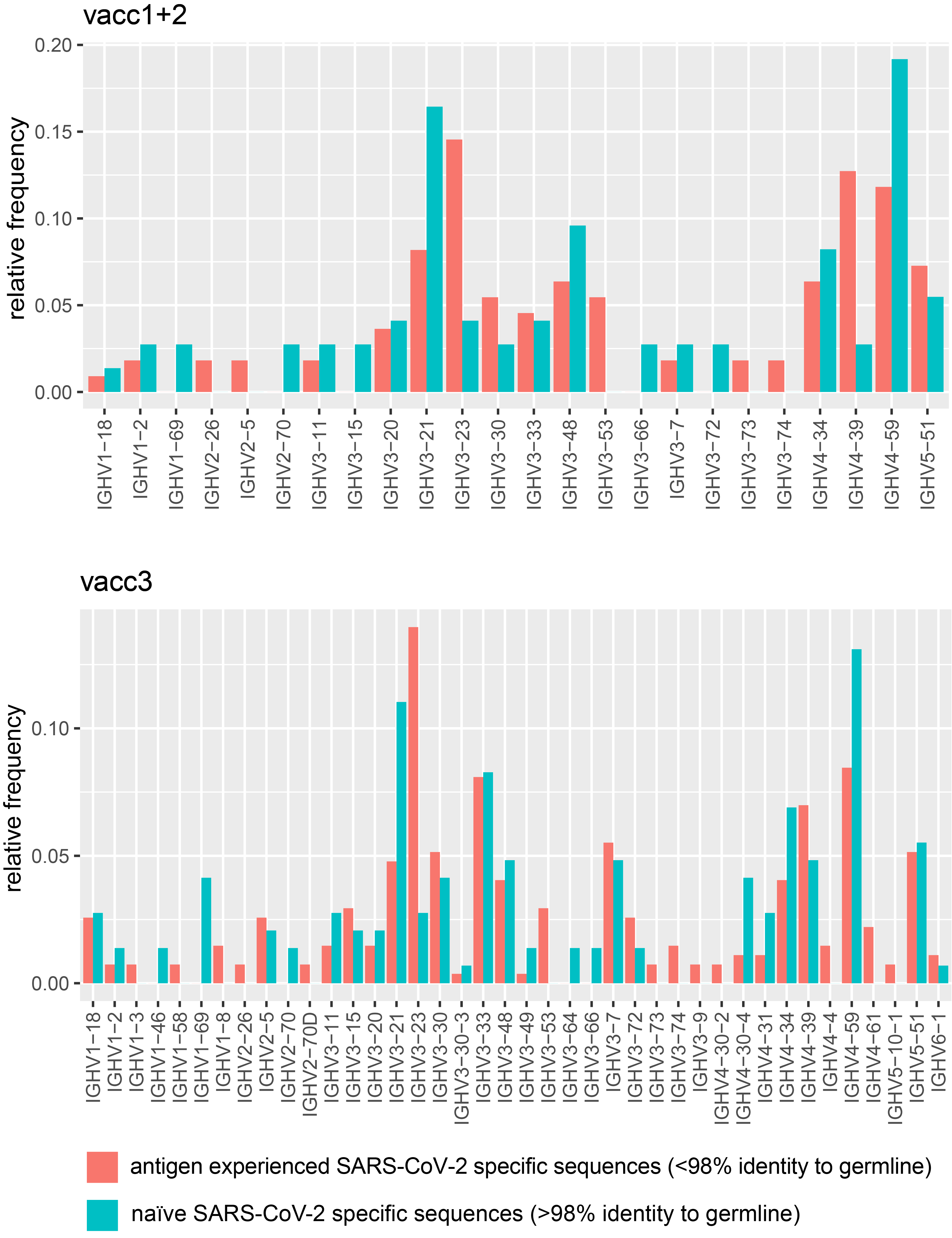


**Supplementary Figure 3. IGHV gene usage in naïve versus antigen-experienced B cell in expanding lineages.**

## Supplementary Tables

| **Supplementary Table 1:** List of SARS-CoV-2 directed sequences used in our search algorithm. Sequences were derived from CoV-AbDab (1), accessed 17th December 2021. | | | |
| --- | --- | --- | --- |
| 1. Raybould MIJ, Kovaltsuk A, Marks C, and Deane CM. CoV-AbDab: the Coronavirus Antibody Database. Accessed 2021-12-17. | | | |
| **Set1: 3,195 total SARS-CoV2 binding antibody sequences** | | | |
| **IGHV gene** | **IGHJ gene** | **CDRH3** | |
| IGHV3-23 (Human) | IGHJ4 (Human) | VSNWASGSTGDY | |
| IGHV3-23 (Human) | IGHJ4 (Human) | VSNWASGSTGDY | |
| IGHV1-2 (Human) | IGHJ5 (Human) | ATDGDDGENWFDP | |
| IGHV1-24 (Human) | IGHJ6 (Human) | ATAGAITGTPRNFYYYYGMDV | |
| IGHV1-24 (Human) | IGHJ5 (Human) | VTSQPFVSPNWFDP | |
| IGHV1-24 (Human) | IGHJ5 (Human) | ATSPAVVERGWFDP | |
| IGHV1-24 (Human) | IGHJ6 (Human) | ATAGAITGTPTNYSYYYGMDF | |
| IGHV1-24 (Human) | IGHJ4 (Human) | TTGPAVTNRPADY | |
| IGHV1-24 (Human) | IGHJ4 (Human) | ATTPAFMAAAGDFDY | |
| IGHV4-4 (Human) | IGHJ4 (Human) | ARGWNYDY | |
| IGHV3-21 (Human) | IGHJ2 (Human) | ARAPEPQSVIYAYWYFDL | |
| IGHV1-24 (Human) | IGHJ4 (Human) | ATGKINYYDSSGYYGTFDY | |
| IGHV3-30 (Human) | IGHJ4 (Human) | ARDTGGIIDY | |
| IGHV3-30-3 (Human) | IGHJ4 (Human) | ARDGDGLWLGESFYFDD | |
| IGHV3-33 (Human) | IGHJ3 (Human) | ARDSGDDYDSSGYPIGNAFDL | |
| IGHV1-24 (Human) | IGHJ4 (Human) | ATGSVGPDY | |
| IGHV3-53 (Human) | IGHJ4 (Human) | ASFLVGATGQPWY | |
| IGHV3-30-3 (Human) | IGHJ4 (Human) | ARDGVSVTMVRGVIGPLCDY | |
| IGHV3-30 (Human) | IGHJ1 (Human) | AKDSPYYYDSSGYYPGYFQD | |
| IGHV3-48 (Human) | IGHJ4 (Human) | ARVARRDVAPATIAAYFLDY | |
| IGHV3-30 (Human) | IGHJ4 (Human) | AKGGERWELFTSFDY | |
| IGHV4-39 (Human) | IGHJ5 (Human) | ARERPPFDVVVVPAARPYNWFDP | |
| IGHV3-11 (Human) | IGHJ6 (Human) | ARDWSVVVVPAAPAVDV | |
| IGHV1-69 (Human) | IGHJ4 (Human) | AKVADRVGASTGELDY | |
| IGHV3-30 (Human) | IGHJ3 (Human) | AKVLMMGDSSGFSLGDAFDI | |
| IGHV5-51 (Human) | IGHJ4 (Human) | ARLLYYSDSSPLDS | |
| IGHV3-33 (Human) | IGHJ3 (Human) | ARGPLGSNYFNYDAFDI | |
| IGHV3-15 (Human) | IGHJ3 (Human) | TTEYSSIAARIDAFDI | |
| IGHV3-33 (Human) | IGHJ6 (Human) | ARDLSSSSGWDDYYYYYGMDV | |
| IGHV3-30-3 (Human) | IGHJ4 (Human) | ARADKNTAMFFDY | |
| IGHV3-30-3 (Human) | IGHJ4 (Human) | ARDSGDYVDYFDY | |
| IGHV3-53 (Human) | IGHJ6 (Human) | ARDLSEKGGMDV | |
| IGHV5-51 (Human) | IGHJ4 (Human) | ARQNVGYFGDFDY | |
| IGHV4-59 (Human) | IGHJ6 (Human) | ASYYYDSSGYYYGMDV | |
| IGHV3-48 (Human) | IGHJ6 (Human) | ARHGGPTSYYYYGMDV | |
| IGHV3-30 (Human) | IGHJ4 (Human) | AKLGGPYCSGGSCYSGYFDY | |
| IGHV5-51 (Human) | IGHJ4 (Human) | ARQVLTYYDILTGYSFEYYFDY | |
| IGHV3-23 (Human) | IGHJ6 (Human) | AKDLYYDSSGIDDYYYYYGMDV | |
| IGHV3-9 (Human) | IGHJ6 (Human) | AKDYCGGDCYSPTRGAGYGMDV | |
| IGHV3-21 (Human) | IGHJ5 (Human) | AREVPIGMEYDFWSGRRNWFDP | |
| IGHV1-46 (Human) | IGHJ4 (Human) | ARDSGFIVVVEAFDY | |
| IGHV3-23 (Human) | IGHJ6 (Human) | AKDLHYDNSGIDDYYYYYGMDV | |
| IGHV3-30 (Human) | IGHJ4 (Human) | AKLGGPYCSGGNCYSGYFDY | |
| IGHV3-9 (Human) | IGHJ6 (Human) | AKDYCGGDCYSPTRGAGYGMDV | |
| IGHV3-48 (Human) | IGHJ6 (Human) | ARDLVPAALSYYAYGMDV | |
| IGHV3-66 (Human) | IGHJ4 (Human) | ARGPYPRGFDY | |
| IGHV3-33 (Human) | IGHJ5 (Human) | ARDSSGSLLEFPVTWFDP | |
| IGHV3-53 (Human) | IGHJ4 (Human) | ARDYGDYYFDY | |
| IGHV3-30 (Human) | IGHJ4 (Human) | ASLITMVRGVPDY | |
| IGHV3-15 (Human) | IGHJ4 (Human) | TTISDYGDYSPVY | |
| IGHV4-59 (Human) | IGHJ4 (Human) | ARWWRVLPFSSFDY | |
| IGHV4-59 (Human) | IGHJ6 (Human) | ARGTVIKVGRTSYSYGMDV | |
| IGHV3-48 (Human) | IGHJ6 (Human) | AGGILGLGSLHYYYGLDV | |
| IGHV1-18 (Human) | IGHJ4 (Human) | AGGLFPRGYMYGSRETPALDY | |
| IGHV3-23 (Human) | IGHJ3 (Human) | AKVLHGGWFPFDAFDI | |
| IGHV4-59 (Human) | IGHJ5 (Human) | ARDSPYYYDSSDYYPASFDP | |
| IGHV3-53 (Human) | IGHJ4 (Human) | ARDYGDFYFDY | |
| IGHV3-13 (Human) | IGHJ4 (Human) | VRGVDFRYCSGGRCFSHYYFDY | |
| IGHV1-18 (Human) | IGHJ4 (Human) | ARVTGTYHSDRSGLPGMIYYFDY | |
| IGHV3-66 (Human) | IGHJ4 (Human) | ARSYGDFYMDY | |
| IGHV1-69 (Human) | IGHJ4 (Human) | ASRRLSTDYDRHY | |
| IGHV4-31 (Human) | IGHJ3 (Human) | ARDTLWGAFDI | |
| IGHV1-46 (Human) | IGHJ6 (Human) | ARGGFVPAAGDYGFDV | |
| IGHV3-30 (Human) | IGHJ4 (Human) | VSLITMIRGVPDC | |
| IGHV3-15 (Human) | IGHJ4 (Human) | TTTSDYGDYSPVY | |
| IGHV3-23 (Human) | IGHJ4 (Human) | TLGSGSSSLGNNY | |
| IGHV1-8 (Human) | IGHJ5 (Human) | ARGTYCSSTSCYTRDWFDP | |
| IGHV3-11 (Human) | IGHJ6 (Human) | ARDSAGNWFDSRHYGMDV | |
| IGHV3-53 (Human) | IGHJ4 (Human) | ARDYGDFYFDY | |
| IGHV3-66 (Human) | IGHJ3 (Human) | ARETYAFDI | |
| IGHV3-49 (Human) | IGHJ1 (Human) | TRDGDYCGGDCPEYFQH | |
| IGHV1-69D (Human) | IGHJ6 (Human) | ARETRGFGDLFGKGYFYYSGMDV | |
| IGHV1-46 (Human) | IGHJ3 (Human) | ARDIVVVPAKNAFDI | |
| IGHV3-9 (Human) | IGHJ6 (Human) | AKDIMVWYGLSRDGYNFAYYGMDV | |
| IGHV1-18 (Human) | IGHJ6 (Human) | ARASGFMVRGVNPNYYYYMDV | |
| IGHV3-66 (Human) | IGHJ4 (Human) | ARDLVVRGTSY | |
| IGHV5-51 (Human) | IGHJ5 (Human) | AIAKYMYYDILTGYYNDGWSFDP | |
| IGHV3-9 (Human) | IGHJ5 (Human) | AKAHVDTAMVKGPFWFDP | |
| IGHV1-8 (Human) | IGHJ5 (Human) | ARGTYCSTTICYTRDWFDP | |
| IGHV3-53 (Human) | IGHJ4 (Human) | ARDYGDFYFDY | |
| IGHV3-66 (Human) | IGHJ6 (Human) | ARGHCSTTSCFRDGQDV | |
| IGHV1-18 (Human) | IGHJ6 (Human) | ARASGFMVRGVNPNYYYYMDV | |
| IGHV3-66 (Human) | IGHJ4 (Human) | ARDLVVRGTSY | |
| IGHV5-51 (Human) | IGHJ5 (Human) | AIAKYMYYDILTGYYKDGWSFDP | |
| IGHV3-9 (Human) | IGHJ5 (Human) | AKAHVVTVMIKGPFWFDP | |
| IGHV3-30 (Human) | IGHJ4 (Human) | AKAGGAYCGGDCPSSLDY | |
| IGHV3-33 (Human) | IGHJ6 (Human) | ARGDSSGWFPLGMDV | |
| IGHV1-46 (Human) | IGHJ5 (Human) | ARDFIEDGPNILTGFNWFDP | |
| IGHV1-46 (Human) | IGHJ4 (Human) | ARGPPPTVLTPFDF | |
| IGHV5-51 (Human) | IGHJ4 (Human) | ARRTSYYDSGGFYYLDY | |
| IGHV4-39 (Human) | IGHJ4 (Human) | ARHGGIYYYDSSGYFFDY | |
| IGHV3-53 (Human) | IGHJ6 (Human) | ARYMGDGMDV | |
| IGHV3-66 (Human) | IGHJ3 (Human) | ARDLEERGAFDI | |
| IGHV1-69D (Human) | IGHJ4 (Human) | AREPGLHTGHLDH | |
| IGHV3-53 (Human) | IGHJ6 (Human) | ARHPLPVSHYYYGMDV | |
| IGHV4-59 (Human) | IGHJ4 (Human) | ARLAPFFYDSSGYYSRSFDY | |
| IGHV1-69D (Human) | IGHJ2 (Human) | ARDLSPCLGGDCDLFGGGRKPHWYFGL | |
| IGHV4-34 (Human) | IGHJ4 (Human) | ARSPGWTTVTRPFDY | |
| IGHV4-34 (Human) | IGHJ4 (Human) | ARSPLWTTVARIFDY | |
| IGHV3-33 (Human) | IGHJ4 (Human) | AKNGAPYCSGGSCYSGYFDY | |
| IGHV3-15 (Human) | IGHJ3 (Human) | TTGSLSELSLIMWGAFDI | |
| IGHV3-53 (Human) | IGHJ4 (Human) | ARELDTIFDY | |
| IGHV1-2 (Human) | IGHJ4 (Human) | ARELVGRYYYDSSGYADY | |
| IGHV3-9 (Human) | IGHJ4 (Human) | AKDAGKGQWLVDLYFDY | |
| IGHV3-53 (Human) | IGHJ6 (Human) | ARDLSYYGMDV | |
| IGHV3-53 (Human) | IGHJ4 (Human) | ARDYGDLYFDY | |
| IGHV3-43 (Human) | IGHJ6 (Human) | AKDLGDDILTGYYTGYYYYYGMDV | |
| IGHV3-53 (Human) | IGHJ3 (Human) | ARDLELKGAFDI | |
| IGHV1-46 (Human) | IGHJ6 (Human) | ARDLVVVPAAMGMDV | |
| IGHV3-30 (Human) | IGHJ4 (Human) | AKVGGPYCSGGSCYGGYFDY | |
| IGHV3-53 (Human) | IGHJ4 (Human) | ARGYGDLYFDY | |
| IGHV3-13 (Human) | IGHJ2 (Human) | ARGADSSNLWYFDF | |
| IGHV3-33 (Human) | IGHJ4 (Human) | AKVGGPYCGGGSCYGGYFDY | |
| IGHV3-53 (Human) | IGHJ3 (Human) | ARDLELKGAFDI | |
| IGHV1-46 (Human) | IGHJ6 (Human) | ARDLVVVPAAMGMDV | |
| IGHV1-8 (Human) | IGHJ4 (Human) | ARVSDILTGYIDY | |
| IGHV1-69D (Human) | IGHJ6 (Human) | ARNSREDGVWSGYYTSVYYGMDV | |
| IGHV3-53 (Human) | IGHJ4 (Human) | ARELKGRFDY | |
| IGHV3-53 (Human) | IGHJ4 (Human) | ARDLEEAGGFDY | |
| IGHV1-2 (Human) | IGHJ6 (Human) | ARNYPPLLQQLGFMDV | |
| IGHV1-69D (Human) | IGHJ5 (Human) | ARAGYCTSTVCSRLAPRGWLDP | |
| IGHV1-69 (Human) | IGHJ6 (Human) | ARDARGCSHDSVWGSFQDCYHGMDV | |
| IGHV3-33 (Human) | IGHJ1 (Human) | ASTSYYYDSSGYTRSEYFQH | |
| IGHV3-7 (Human) | IGHJ4 (Human) | ARDSRGHYYDSSGYPAFDY | |
| IGHV3-9 (Human) | IGHJ6 (Human) | AKERAGPQIKTYYYYGLDV | |
| IGHV4-39 (Human) | IGHJ5 (Human) | ARLVGGNGWYGRSLGDNRFDP | |
| IGHV1-69 (Human) | IGHJ6 (Human) | ARDARGCFHDDVWGSFRDCYNGMAV | |
| IGHV3-53 (Human) | IGHJ4 (Human) | ARGYGDLYFDY | |
| IGHV3-13 (Human) | IGHJ2 (Human) | ARGADSSNLWYFDL | |
| IGHV1-69D (Human) | IGHJ6 (Human) | ARDIEYSPLYGMDV | |
| IGHV4-31 (Human) | IGHJ4 (Human) | ARVGDFDWFDT | |
| IGHV1-46 (Human) | IGHJ4 (Human) | ARASWGSTLDY | |
| IGHV3-23 (Human) | IGHJ4 (Human) | AKVWPRSSEGYFFDL | |
| IGHV1-2 (Human) | IGHJ4 (Human) | ARDLSWSRLSGGTDF | |
| IGHV1-2 (Human) | IGHJ4 (Human) | ARDLSWSRLSGGTDF | |
| IGHV3-53 (Human) | IGHJ6 (Human) | ARDAQIYGMDV | |
| IGHV3-9 (Human) | IGHJ6 (Human) | AKDILGNYYYYSMHV | |
| IGHV4-61 (Human) | IGHJ6 (Human) | AREGLRTLGPPQLPDYFGIDV | |
| IGHV4-59 (Human) | IGHJ4 (Human) | ASGRDESKTGY | |
| IGHV4-39 (Human) | IGHJ4 (Human) | ARQKWLRSDFDY | |
| IGHV3-66 (Human) | IGHJ4 (Human) | ARDRGLVSDY | |
| IGHV3-53 (Human) | IGHJ4 (Human) | AREYGDWE | |
| IGHV3-30 (Human) | IGHJ4 (Human) | AKPQYSYGYEEGIYFDY | |
| IGHV5-51 (Human) | IGHJ6 (Human) | ARGEVPAAYRPMDYGMDV | |
| IGHV3-23 (Human) | IGHJ4 (Human) | AKTPMASPATRYDY | |
| IGHV3-9 (Human) | IGHJ6 (Human) | AKGLAARHYYYYGMDV | |
| IGHV3-66 (Human) | IGHJ3 (Human) | ARDLEEKGAFDI | |
| IGHV1-18 (Human) | IGHJ5 (Human) | ARDRVLRFLGFDP | |
| IGHV3-9 (Human) | IGHJ3 (Human) | AKDIRGPYDRTEEGAFDI | |
| IGHV3-48 (Human) | IGHJ6 (Human) | ARHGGAMSYYYYGMDV | |
| IGHV3-30 (Human) | IGHJ4 (Human) | AKPIYSYGSGSYSYFDY | |
| IGHV3-21 (Human) | IGHJ5 (Human) | AREGFYCSGGGCRWFDP | |
| IGHV1-2 (Human) | IGHJ5 (Human) | ARGPLVPAAIRGNWFDP | |
| IGHV3-23 (Human) | IGHJ3 (Human) | AKDPFYRWFNHGDAFDI | |
| IGHV3-66 (Human) | IGHJ4 (Human) | ARGYGDYYFDY | |
| IGHV4-59 (Human) | IGHJ4 (Human) | ASGRDESKTGY | |
| IGHV3-53 (Human) | IGHJ4 (Human) | AREYGDWE | |
| IGHV1-2 (Human) | IGHJ5 (Human) | ARGPLLPVAIRGNWFDP | |
| IGHV3-23 (Human) | IGHJ3 (Human) | AKDPFYRWFNHGDAFDI | |
| IGHV3-66 (Human) | IGHJ4 (Human) | ARGYGDYYFDY | |
| IGHV1-3 (Human) | IGHJ3 (Human) | ARDFGGRESRGGAFDI | |
| IGHV3-30 (Human) | IGHJ4 (Human) | AKNFYSYGYQGYYFDY | |
| IGHV3-7 (Human) | IGHJ6 (Human) | ARDRWLRGDMDV | |
| IGHV3-53 (Human) | IGHJ6 (Human) | ARDLQQLGGMDV | |
| IGHV3-53 (Human) | IGHJ6 (Human) | ARFGGYELGGVDV | |
| IGHV3-30 (Human) | IGHJ4 (Human) | AKDGGYYYESSGYFDY | |
| IGHV3-53 (Human) | IGHJ4 (Human) | ARDFGGLRFDY | |
| IGHV3-21 (Human) | IGHJ4 (Human) | ARERRSDYSSDWYDPPTTPRSFDY | |
| IGHV3-53 (Human) | IGHJ4 (Human) | ARDRGDSLFDY | |
| IGHV3-53 (Human) | IGHJ6 (Human) | AREVYAMDV | |
| IGHV3-23 (Human) | IGHJ4 (Human) | AKEKFNGDYGGVVDY | |
| IGHV3-11 (Human) | IGHJ5 (Human) | ARGEQTGVPGGWLDS | |
| IGHV3-30 (Human) | IGHJ4 (Human) | AKQGGIYCSGGSCYSGYFDY | |
| IGHV1-8 (Human) | IGHJ4 (Human) | ARGSLIFGVVTHFDY | |
| IGHV1-18 (Human) | IGHJ5 (Human) | ARALTGVYNWFDP | |
| IGHV3-66 (Human) | IGHJ4 (Human) | ARDRGLVSDY | |
| IGHV3-53 (Human) | IGHJ6 (Human) | ARDLQQRGGMDV | |
| IGHV3-30 (Human) | IGHJ4 (Human) | AKQGEIYCSAGNCYSGYYDY | |
| IGHV1-8 (Human) | IGHJ4 (Human) | ARGSLIFGVVTHFDY | |
| IGHV1-18 (Human) | IGHJ5 (Human) | ARALTGVYNWFDP | |
| IGHV3-53 (Human) | IGHJ6 (Human) | ARDLLEAGGMDV | |
| IGHV3-30 (Human) | IGHJ4 (Human) | AKDPMPGAVATTGSFDY | |
| IGHV3-9 (Human) | IGHJ3 (Human) | AKDMGTGTTTMRPSDAFDI | |
| IGHV4-31 (Human) | IGHJ4 (Human) | AGSSGWFGEFFDY | |
| IGHV4-31 (Human) | IGHJ4 (Human) | ARDYGGNANYFHY | |
| IGHV1-18 (Human) | IGHJ5 (Human) | ARDGELMGWFDP | |
| IGHV4-39 (Human) | IGHJ6 (Human) | ARLPYYYGSGSYYNRDYYYYGMDV | |
| IGHV3-23 (Human) | IGHJ4 (Human) | AKGKHWNDDYFEY | |
| IGHV3-13 (Human) | IGHJ2 (Human) | ARADYGDYGFYWYFDL | |
| IGHV3-9 (Human) | IGHJ4 (Human) | AKDRTDRLLTFDY | |
| IGHV4-39 (Human) | IGHJ1 (Human) | ARHETDYGGVAGHFQH | |
| IGHV3-30 (Human) | IGHJ4 (Human) | ARDMGSAMVDYFDY | |
| IGHV5-51 (Human) | IGHJ4 (Human) | ARLYCGGDCPFDY | |
| IGHV3-53 (Human) | IGHJ4 (Human) | ARDFGEFYFDY | |
| IGHV4-61 (Human) | IGHJ6 (Human) | ARDRIVVVPAATPDYYGMDV | |
| IGHV3-13 (Human) | IGHJ4 (Human) | ARGGMYSSTWYFDY | |
| IGHV1-46 (Human) | IGHJ6 (Human) | ARDLNRITIFGGMDV | |
| IGHV3-9 (Human) | IGHJ3 (Human) | AKDISVLDYGVYGAIDV | |
| IGHV3-64D (Human) | IGHJ4 (Human) | VKDDFSSPTVLFDY | |
| IGHV3-64D (Human) | IGHJ4 (Human) | VKDLGLNTYYDVFDY | |
| IGHV1-46 (Human) | IGHJ4 (Human) | AREDSGSSRLDY | |
| IGHV3-9 (Human) | IGHJ4 (Human) | AKDRTDRLLTFDY | |
| IGHV4-39 (Human) | IGHJ1 (Human) | ARHETDYGGVAGHFQH | |
| IGHV5-51 (Human) | IGHJ4 (Human) | ARQYCGGDCPFDY | |
| IGHV3-64D (Human) | IGHJ4 (Human) | VKDDFSSPTVLFDY | |
| IGHV3-64D (Human) | IGHJ4 (Human) | VKDLGVNTYYDVFDF | |
| IGHV1-46 (Human) | IGHJ6 (Human) | ARDNDILTGYYLGHPHGMDV | |
| IGHV1-69D (Human) | IGHJ4 (Human) | ASCRYYYDSSGYAQPEDYFDY | |
| IGHV3-23 (Human) | IGHJ4 (Human) | AKDLAAGGTMIVVVQEFDY | |
| IGHV3-21 (Human) | IGHJ5 (Human) | AREGSTSCYPSFICDWFDP | |
| IGHV3-53 (Human) | IGHJ6 (Human) | ARDIVGAIRGMDV | |
| IGHV4-59 (Human) | IGHJ3 (Human) | ARDLPHYDFWSGSSLDAFDI | |
| IGHV1-46 (Human) | IGHJ4 (Human) | ARVEPPITQEMATILPYFDY | |
| IGHV3-13 (Human) | IGHJ6 (Human) | ARANYFDSSGYGYYYYGMDV | |
| IGHV5-51 (Human) | IGHJ6 (Human) | ARLAGYYGSGRSDYYYGMDV | |
| IGHV3-53 (Human) | IGHJ4 (Human) | ARDLGPSGGIDY | |
| IGHV3-48 (Human) | IGHJ4 (Human) | ARDLFMYYDILTGYYNTPTYFDY | |
| IGHV3-30 (Human) | IGHJ4 (Human) | AKAPGAYCSGGSCYSPSYFDY | |
| IGHV3-53 (Human) | IGHJ3 (Human) | ARDLYSSGGTDI | |
| IGHV3-30 (Human) | IGHJ4 (Human) | AKRGGGGYYDSSGYLDY | |
| IGHV3-21 (Human) | IGHJ4 (Human) | ARETPAPVSPTQEFDY | |
| IGHV3-43 (Human) | IGHJ4 (Human) | AKPGDDFWSGYFKGGVDY | |
| IGHV3-30 (Human) | IGHJ4 (Human) | AKGGPFGYSFPLKYFDY | |
| IGHV3-53 (Human) | IGHJ6 (Human) | ARDLLAYGMDV | |
| IGHV3-13 (Human) | IGHJ4 (Human) | ARDSGTTVTTALDY | |
| IGHV3-23 (Human) | IGHJ4 (Human) | AKDLGYCSGGSCFSGSPG | |
| IGHV1-3 (Human) | IGHJ3 (Human) | AREPPSHSSGYYNESGGAFDI | |
| IGHV3-74 (Human) | IGHJ4 (Human) | ARVGGTIVGDADYFDY | |
| IGHV3-53 (Human) | IGHJ6 (Human) | ARDLYVYGMDV | |
| IGHV3-53 (Human) | IGHJ4 (Human) | ARVLPMNWYYFDY | |
| IGHV5-51 (Human) | IGHJ4 (Human) | ARQICGGDCPIDY | |
| IGHV3-30 (Human) | IGHJ4 (Human) | AKNLGIYCSGGSCYSGSFDY | |
| IGHV3-64D (Human) | IGHJ4 (Human) | VKDREDIVVVHFDY | |
| IGHV3-30-3 (Human) | IGHJ4 (Human) | AKRGGTYCSGGSCGSGYFDY | |
| IGHV4-30-2 (Human) | IGHJ4 (Human) | ARAYCGGDCPFDY | |
| IGHV1-3 (Human) | IGHJ3 (Human) | AREPPSHSSGYYYGSGGAFDI | |
| IGHV3-33 (Human) | IGHJ4 (Human) | AKAGLYGDYVRFGY | |
| IGHV3-30 (Human) | IGHJ6 (Human) | ARGNVDIAMARVNYFYYGMDV | |
| IGHV3-64D (Human) | IGHJ4 (Human) | VKDREDIVVVHFDY | |
| IGHV3-33 (Human) | IGHJ4 (Human) | AKQGGTYCSGGSCGSGYFDY | |
| IGHV1-58 (Human) | IGHJ3 (Human) | AANYCSGGSCSDAFDI | |
| IGHV4-30-2 (Human) | IGHJ4 (Human) | ARTLCGGDCPFDY | |
| IGHV3-30 (Human) | IGHJ4 (Human) | AKVGYSYGYSYNYFDY | |
| IGHV3-30 (Human) | IGHJ4 (Human) | AKANAPYCSGGNCYSGYFDY | |
| IGHV3-23 (Human) | IGHJ1 (Human) | AKGGDTAMVNAEYFQH | |
| IGHV3-23 (Human) | IGHJ4 (Human) | AKDRYNGPREAYFDD | |
| IGHV5-51 (Human) | IGHJ4 (Human) | ARQGPMGGGPFDY | |
| IGHV1-69D (Human) | IGHJ5 (Human) | ARGRAAAAPPFTWFDP | |
| IGHV1-46 (Human) | IGHJ6 (Human) | ARDSAFIPHIEYGMDV | |
| IGHV3-30 (Human) | IGHJ4 (Human) | AKDPRNGISGTTGYFDY | |
| IGHV3-9 (Human) | IGHJ5 (Human) | AKDIYPLDYGDAFFFDS | |
| IGHV3-11 (Human) | IGHJ4 (Human) | APQLWLRGAFDY | |
| IGHV4-39 (Human) | IGHJ4 (Human) | ATQGWLRGYFDY | |
| IGHV4-39 (Human) | IGHJ4 (Human) | ARREWLRGGFDY | |
| IGHV4-39 (Human) | IGHJ4 (Human) | ARLTWLRGNFDY | |
| IGHV4-31 (Human) | IGHJ5 (Human) | ARGTFFSWWFDP | |
| IGHV3-30 (Human) | IGHJ4 (Human) | AKGGWYSSPTYYFDY | |
| IGHV3-30 (Human) | IGHJ5 (Human) | ARDWGSTVTLFDS | |
| IGHV3-23 (Human) | IGHJ4 (Human) | AKTNTGWYLGIDY | |
| IGHV3-66 (Human) | IGHJ4 (Human) | ARDYGAYGRRNDF | |
| IGHV3-9 (Human) | IGHJ4 (Human) | AKDQGNYGDYFDY | |
| IGHV3-30 (Human) | IGHJ3 (Human) | ARDGRTITMVQGVISGAFDI | |
| IGHV4-31 (Human) | IGHJ4 (Human) | ASGRWLPEFDY | |
| IGHV4-39 (Human) | IGHJ3 (Human) | ARSLLRWNGAFDI | |
| IGHV4-34 (Human) | IGHJ4 (Human) | ARDDGGFRELFLDY | |
| IGHV3-30 (Human) | IGHJ4 (Human) | AKGGLYSSSSLDDY | |
| IGHV3-30 (Human) | IGHJ4 (Human) | AKDSTPISWIQLWGTDYFDY | |
| IGHV3-33 (Human) | IGHJ4 (Human) | AKQAGPYCSGGNCYGGTLDY | |
| IGHV3-30-3 (Human) | IGHJ4 (Human) | AKTLYSYAYDQYFFDY | |
| IGHV1-46 (Human) | IGHJ6 (Human) | ARDSAFIPHMDYGLDV | |
| IGHV1-46 (Human) | IGHJ4 (Human) | AREHGGNSYFDQ | |
| IGHV3-15 (Human) | IGHJ6 (Human) | IASDLLRYYSYGIDV | |
| IGHV3-7 (Human) | IGHJ5 (Human) | ARPRGGWYMGLDS | |
| IGHV4-39 (Human) | IGHJ3 (Human) | ARRRRWGFGDGFDI | |
| IGHV4-31 (Human) | IGHJ3 (Human) | ARVPRHYGDNVHHAFDI | |
| IGHV3-30 (Human) | IGHJ4 (Human) | AKGGYSYAYPETFFDY | |
| IGHV1-46 (Human) | IGHJ4 (Human) | ARDAGFIVVVTGGFDY | |
| IGHV3-30 (Human) | IGHJ3 (Human) | AREPHCTGGVCDAFDM | |
| IGHV3-30 (Human) | IGHJ3 (Human) | ASHSAGYGDSYETFDI | |
| IGHV3-13 (Human) | IGHJ4 (Human) | ARGVGGTVMDPYRFDY | |
| IGHV4-39 (Human) | IGHJ6 (Human) | ARRRLPPSSYYSAMDV | |
| IGHV3-66 (Human) | IGHJ5 (Human) | ARGEGYDIPFDP | |
| IGHV3-7 (Human) | IGHJ4 (Human) | ARDAYYYGDYDY | |
| IGHV4-59 (Human) | IGHJ4 (Human) | ARHKWLRGELDY | |
| IGHV4-59 (Human) | IGHJ2 (Human) | ARQGWLRGNFDL | |
| IGHV3-66 (Human) | IGHJ2 (Human) | ARALPFFGDLGYFDF | |
| IGHV1-46 (Human) | IGHJ6 (Human) | ARDENFIPARGAMDV | |
| IGHV1-46 (Human) | IGHJ4 (Human) | ARDEVLIPAARALDY | |
| IGHV1-46 (Human) | IGHJ4 (Human) | ARDFALIPASMGLEY | |
| IGHV5-51 (Human) | IGHJ5 (Human) | ARRFGDTYPNVWFDP | |
| IGHV4-4 (Human) | IGHJ2 (Human) | ARDREDGMGLWYFDL | |
| IGHV3-30 (Human) | IGHJ4 (Human) | AITFPRGELTGILHY | |
| IGHV3-66 (Human) | IGHJ5 (Human) | ARFGSYGRRLYDS | |
| IGHV4-59 (Human) | IGHJ4 (Human) | ARHRLGLILGSDY | |
| IGHV3-48 (Human) | IGHJ5 (Human) | ARERRPYYDFWSGPDSGFDP | |
| IGHV3-33 (Human) | IGHJ2 (Human) | AREGDCSGGNCWKGIWYFDL | |
| IGHV4-31 (Human) | IGHJ4 (Human) | ARLKRYLEWSKETYEYYFDY | |
| IGHV4-31 (Human) | IGHJ6 (Human) | ARRGVDSILIGASVIDYYYYGMDV | |
| IGHV1-18 (Human) | IGHJ4 (Human) | ARDRLAAAIDYFDY | |
| IGHV3-33 (Human) | IGHJ4 (Human) | AKGAEPYCSAGNCYSNYFDY | |
| IGHV3-30 (Human) | IGHJ5 (Human) | ARDWGSTVTLFDS | |
| IGHV3-9 (Human) | IGHJ5 (Human) | AKDIYPLDYGDAFFFDS | |
| IGHV3-66 (Human) | IGHJ6 (Human) | ARESYGMDV | |
| IGHV3-23 (Human) | IGHJ6 (Human) | AKLASTSWTVYFYHAMDV | |
| IGHV1-69 (Human) | IGHJ4 (Human) | ARDQWESDYYDNSDYYYVDY | |
| IGHV1-69 (Human) | IGHJ1 (Human) | ARGSFRDPAMWGPMPEYFQH | |
| IGHV3-30 (Human) | IGHJ6 (Human) | VKDPVGGYCRGGNCYDFYAMDV | |
| IGHV1-69 (Human) | IGHJ4 (Human) | ARASYYYDSSGYHFDY | |
| IGHV3-64D (Human) | IGHJ4 (Human) | VKDKEADKAMYVLFDY | |
| IGHV3-30 (Human) | IGHJ4 (Human) | ARDYCGSTSCPYLFDY | |
| IGHV5-10-1 (Human) | IGHJ3 (Human) | ARQEYYDSSGYQGDAFDI | |
| IGHV4-34 (Human) | IGHJ4 (Human) | ARLVREYSFGLDY | |
| IGHV1-69 (Human) | IGHJ3 (Human) | ARVLYYYDSSGYYRTDDAFDI | |
| IGHV3-30 (Human) | IGHJ3 (Human) | AKGGLGIMIFGVDDDFDI | |
| IGHV3-64D (Human) | IGHJ3 (Human) | VKDWSTCSSTSCYPAGDAFDI | |
| IGHV4-38-2 (Human) | IGHJ4 (Human) | ARDENDDYDSSDYYYPRQKCYFEY | |
| IGHV3-30 (Human) | IGHJ6 (Human) | AKDGGPYYYDRSGYARDDYYGMDV | |
| IGHV1-69 (Human) | IGHJ4 (Human) | ARQGVLYYESSGLMAHYFDY | |
| IGHV1-69 (Human) | IGHJ3 (Human) | ARVLFYYDTSGPRSDDAFDI | |
| IGHV3-23 (Human) | IGHJ6 (Human) | AKGQGAYSAYDPDYYYGMDV | |
| IGHV3-23 (Human) | IGHJ6 (Human) | AGRYCSSNTYSGCYYYGMDV | |
| IGHV1-69 (Human) | IGHJ4 (Human) | ARETWYYDRSGYPRSDEYFQY | |
| IGHV1-18 (Human) | IGHJ4 (Human) | ARDLGSSGYYDSSDSYVDFDY | |
| IGHV3-53 (Human) | IGHJ3 (Human) | ARDRGNDAFDI | |
| IGHV3-66 (Human) | IGHJ4 (Human) | ARELRGYFDY | |
| IGHV3-23 (Human) | IGHJ4 (Human) | VMDTVLITDRYFDY | |
| IGHV3-33 (Human) | IGHJ4 (Human) | ARDRMSSSWANFDY | |
| IGHV3-30 (Human) | IGHJ4 (Human) | ARESNHMVTVVFDY | |
| IGHV3-66 (Human) | IGHJ6 (Human) | ARESYGMDV | |
| IGHV1-69 (Human) | IGHJ4 (Human) | ARDQWESDYYDNSDYYYVDY | |
| IGHV1-69 (Human) | IGHJ1 (Human) | ARGSFRDPAMWGPMPEKFQH | |
| IGHV4-38-2 (Human) | IGHJ4 (Human) | ASNKWLRGNFDY | |
| IGHV3-23 (Human) | IGHJ4 (Human) | AKDIGSTIAVEIMEY | |
| IGHV4-30-4 (Human) | IGHJ5 (Human) | AREVLTTVGGGNWFDP | |
| IGHV3-66 (Human) | IGHJ6 (Human) | ARDITIFGVGNYGMDV | |
| IGHV3-13 (Human) | IGHJ5 (Human) | VRATFDSRNYHNWFDP | |
| IGHV1-46 (Human) | IGHJ4 (Human) | GRFYDSTGVFDY | |
| IGHV4-59 (Human) | IGHJ4 (Human) | ARLEWLQGRFDY | |
| IGHV4-39 (Human) | IGHJ5 (Human) | ARLRWLRGKFDP | |
| IGHV3-13 (Human) | IGHJ4 (Human) | ARGVFDSSGFYSSFDY | |
| IGHV3-30 (Human) | IGHJ4 (Human) | ARDQDVDTSMVPIFAY | |
| IGHV3-53 (Human) | IGHJ5 (Human) | ARDLEMAGGFDS | |
| IGHV3-66 (Human) | IGHJ4 (Human) | ARDLQQAGGFDY | |
| IGHV3-30 (Human) | IGHJ4 (Human) | ARDEGPTVTLFDH | |
| IGHV3-66 (Human) | IGHJ6 (Human) | ARGAGYYYGMDV | |
| IGHV3-23 (Human) | IGHJ4 (Human) | AKTLGRSGFLWFGAVFDY | |
| IGHV3-48 (Human) | IGHJ4 (Human) | AREYEGSYGAFDY | |
| IGHV1-18 (Human) | IGHJ4 (Human) | ARGPSGSYLDLDY | |
| IGHV3-64D (Human) | IGHJ4 (Human) | VKDTLDHFTVFDY | |
| IGHV3-23 (Human) | IGHJ4 (Human) | AKTRTGWYVGFDY | |
| IGHV1-18 (Human) | IGHJ3 (Human) | ARVGVGYCTSTSCYNQPSFDI | |
| IGHV4-4 (Human) | IGHJ4 (Human) | ARSSGLLDY | |
| IGHV4-39 (Human) | IGHJ4 (Human) | ARLLGAVFG | |
| IGHV3-53 (Human) | IGHJ6 (Human) | ARDLQVYGMDV | |
| IGHV1-46 (Human) | IGHJ4 (Human) | ARDIVFVPAAMGLDY | |
| IGHV4-59 (Human) | IGHJ5 (Human) | ARGPPGFDS | |
| IGHV3-49 (Human) | IGHJ3 (Human) | TRVVDILTGYYYPDAFDI | |
| IGHV3-13 (Human) | IGHJ6 (Human) | ARGDTSMISETSQNYYYYYMDV | |
| IGHV1-69 (Human) | IGHJ3 (Human) | AREDIEEVPAAFAYAFDI | |
| IGHV1-69 (Human) | IGHJ3 (Human) | ATHIVLVEFGGGDDAFDI | |
| IGHV1-69 (Human) | IGHJ5 (Human) | ARDNVEGYNSSSIYWFDP | |
| IGHV1-46 (Human) | IGHJ5 (Human) | ARGGLIPDSTNWFDP | |
| IGHV1-18 (Human) | IGHJ4 (Human) | ARVETYYCSGGSCYFYDY | |
| IGHV3-13 (Human) | IGHJ6 (Human) | ARGTTVTTGPIRSYYYYMDV | |
| IGHV3-30-3 (Human) | IGHJ4 (Human) | AKGGGEYCSGGNCYQSYFDY | |
| IGHV3-30 (Human) | IGHJ3 (Human) | ARQLYYYDRSGFLHGDAFDI | |
| IGHV3-21 (Human) | IGHJ6 (Human) | ARAFPGDYASDDYYYYYGMDV | |
| IGHV4-34 (Human) | IGHJ5 (Human) | ARATYYYDSSSYGINWFAP | |
| IGHV1-69 (Human) | IGHJ6 (Human) | ATGVVHIVVVPTYHSYYGLDV | |
| IGHV4-4 (Human) | IGHJ4 (Human) | ARSSGLLDY | |
| IGHV4-39 (Human) | IGHJ4 (Human) | ARLPLGYCSGGTCFPKSL | |
| IGHV4-39 (Human) | IGHJ4 (Human) | ARGLGAFDY | |
| IGHV5-10-1 (Human) | IGHJ3 (Human) | ARPEYCSTTTCSDDAFDI | |
| IGHV1-69D (Human) | IGHJ6 (Human) | ARDRGPPPGGRYGFYYGMDV | |
| IGHV1-18 (Human) | IGHJ4 (Human) | ARVATAYSSGWSMYFFDF | |
| IGHV4-59 (Human) | IGHJ4 (Human) | ARSFSGDEIDF | |
| IGHV4-31 (Human) | IGHJ5 (Human) | ASGRVPYYFDS | |
| IGHV1-69D (Human) | IGHJ6 (Human) | ARDLGEGILHDYGGPYYYYAMDV | |
| IGHV4-4 (Human) | IGHJ3 (Human) | ARERSAVTTWGSAAFHI | |
| IGHV1-46 (Human) | IGHJ5 (Human) | ARDWGGIPAATYFDP | |
| IGHV4-39 (Human) | IGHJ3 (Human) | ARHRTSGTWAPFDAFDI | |
| IGHV3-7 (Human) | IGHJ4 (Human) | ARGRTGPYLGIDY | |
| IGHV3-30 (Human) | IGHJ4 (Human) | AKDARGRYYYDSSGYFDY | |
| IGHV3-66 (Human) | IGHJ3 (Human) | AREGSLEVAGPKLDAFDI | |
| IGHV3-30 (Human) | IGHJ3 (Human) | AKNSDYGDPSALSDDAVDI | |
| IGHV3-30 (Human) | IGHJ4 (Human) | AKAPRGYYDSSGYYRLQDYFDY | |
| IGHV3-13 (Human) | IGHJ2 (Human) | ARAVDSSSWYSNWYFDL | |
| IGHV3-30 (Human) | IGHJ6 (Human) | ARGWFGEEVGYYYFGLDV | |
| IGHV5-51 (Human) | IGHJ4 (Human) | ARTYGSGSYSDFDY | |
| IGHV1-24 (Human) | IGHJ6 (Human) | ATALITMVRGGEYHMDV | |
| IGHV5-51 (Human) | IGHJ4 (Human) | ARSETYVIPPDY | |
| IGHV3-9 (Human) | IGHJ4 (Human) | AKDKIHSSWYGEEAGDFDY | |
| IGHV3-66 (Human) | IGHJ4 (Human) | ARDRGGFIDY | |
| IGHV3-13 (Human) | IGHJ2 (Human) | ARDVGGYWYFDL | |
| IGHV5-51 (Human) | IGHJ4 (Human) | ARQPNVYNWLDT | |
| IGHV3-13 (Human) | IGHJ6 (Human) | ARGDSWNSYFYVFDV | |
| IGHV3-53 (Human) | IGHJ3 (Human) | ARDLAAAGAFDI | |
| IGHV3-66 (Human) | IGHJ6 (Human) | ARDLSVRGGMDV | |
| IGHV3-9 (Human) | IGHJ4 (Human) | AKSARLPGDYFDY | |
| IGHV5-51 (Human) | IGHJ4 (Human) | ARPVGDYPMRHFDY | |
| IGHV3-13 (Human) | IGHJ4 (Human) | ARVNYDSSSFYMFFDY | |
| IGHV3-53 (Human) | IGHJ4 (Human) | ARDVPRNSGGY | |
| IGHV3-66 (Human) | IGHJ4 (Human) | ARDYGDFYFDY | |
| IGHV5-51 (Human) | IGHJ4 (Human) | ARQYCGGDCPFDY | |
| IGHV3-53 (Human) | IGHJ3 (Human) | ARDLSTMGAFDI | |
| IGHV1-69 (Human) | IGHJ6 (Human) | ARDPRPLVPAAINYGMDV | |
| IGHV1-24 (Human) | IGHJ6 (Human) | ATVLISMIRGGEYHMNV | |
| IGHV3-13 (Human) | IGHJ2 (Human) | ARAVDSSSWHSNWYFDL | |
| IGHV3-30 (Human) | IGHJ4 (Human) | AKAPRGYYDRSGYYRIQDNFDY | |
| IGHV3-30 (Human) | IGHJ6 (Human) | ARGWFGEEVGYYYFGLDV | |
| IGHV5-51 (Human) | IGHJ4 (Human) | ARTYGSGSYSDFDY | |
| IGHV5-51 (Human) | IGHJ4 (Human) | ARSETYVMPPDY | |
| IGHV3-33 (Human) | IGHJ6 (Human) | ARDGPANQVDEQLFTITDYYYGMDV | |
| IGHV3-9 (Human) | IGHJ6 (Human) | VKDYHSVRYCSSTSCYRALDDYYGMDV | |
| IGHV3-66 (Human) | IGHJ4 (Human) | ASLEQLTYYFDY | |
| IGHV5-51 (Human) | IGHJ3 (Human) | ARRDRWELEPFDI | |
| IGHV3-53 (Human) | IGHJ4 (Human) | ARDVPRNSGGY | |
| IGHV3-9 (Human) | IGHJ6 (Human) | ATQHIRFLEWGRDELYSGMDV | |
| IGHV3-30 (Human) | IGHJ4 (Human) | AKGHDFGAPDY | |
| IGHV3-33 (Human) | IGHJ6 (Human) | ARDGGYCTNGVCSWGGANYYYYYGMDV | |
| IGHV4-39 (Human) | IGHJ4 (Human) | ARQNGLCDY | |
| IGHV4-59 (Human) | IGHJ4 (Human) | ARGGSTLWFGSEPNFDY | |
| IGHV4-34 (Human) | IGHJ4 (Human) | ARGRVFCTSTSCSFDY | |
| IGHV3-9 (Human) | IGHJ4 (Human) | AKDTVRGIIITKENYYFDY | |
| IGHV3-30 (Human) | IGHJ4 (Human) | AKDAALYYDLWSGYNYFDY | |
| IGHV3-66 (Human) | IGHJ4 (Human) | ARDLHIYGLDC | |
| IGHV4-59 (Human) | IGHJ5 (Human) | ARHYDILTALSWFDP | |
| IGHV1-46 (Human) | IGHJ6 (Human) | ARDVVFVPAASGMDV | |
| IGHV3-9 (Human) | IGHJ3 (Human) | AKVHGSGTYLRGVFDV | |
| IGHV4-39 (Human) | IGHJ4 (Human) | ARHVRVDYDWDY | |
| IGHV1-46 (Human) | IGHJ4 (Human) | ARDQGIIVVISGGLDY | |
| IGHV4-39 (Human) | IGHJ5 (Human) | ASQFVGYSGWYLGNNWFDS | |
| IGHV3-13 (Human) | IGHJ2 (Human) | ARGPRGYYDRTGHFYRNWYFEL | |
| IGHV4-39 (Human) | IGHJ4 (Human) | ARLVRHFGSRGFGAFDY | |
| IGHV4-39 (Human) | IGHJ4 (Human) | ARLVRVFGSGYSRTFDY | |
| IGHV3-66 (Human) | IGHJ3 (Human) | ARDVYEGAFDI | |
| IGHV3-9 (Human) | IGHJ4 (Human) | TKARVTTAMVLTHFDY | |
| IGHV1-69D (Human) | IGHJ4 (Human) | ARGLIPAAWYFDR | |
| IGHV3-9 (Human) | IGHJ6 (Human) | AKARLTGTNPWKFGEDAMDV | |
| IGHV1-46 (Human) | IGHJ3 (Human) | ARGGIVADTKGAFDI | |
| IGHV3-9 (Human) | IGHJ4 (Human) | AKNVYDSSGFYLDY | |
| IGHV4-59 (Human) | IGHJ4 (Human) | ARQRRSTSWSLLDY | |
| IGHV3-53 (Human) | IGHJ4 (Human) | ARDYMLRGGED | |
| IGHV4-39 (Human) | IGHJ5 (Human) | ARHSRMYSDAYYTHSFDS | |
| IGHV4-59 (Human) | IGHJ4 (Human) | ARARTYYYDSSGYPATSFDH | |
| IGHV1-46 (Human) | IGHJ4 (Human) | ARGALVPDTAFGGDY | |
| IGHV5-10-1 (Human) | IGHJ5 (Human) | ARHDRSPGIQYYDILTDNWRDRDWFDP | |
| IGHV3-13 (Human) | IGHJ2 (Human) | ARGGRGYKYGNWYFDL | |
| IGHV1-69D (Human) | IGHJ4 (Human) | ARDVVPAAIAVGGYFDY | |
| IGHV3-9 (Human) | IGHJ5 (Human) | AKSIRQWQVRGAEYYFDS | |
| IGHV3-33 (Human) | IGHJ4 (Human) | ARDEEPENMATRGNLDY | |
| IGHV3-66 (Human) | IGHJ4 (Human) | ARDLHTYGLDY | |
| IGHV3-53 (Human) | IGHJ6 (Human) | ARGGGHYYGMDV | |
| IGHV3-66 (Human) | IGHJ4 (Human) | ARDLGYFDF | |
| IGHV3-33 (Human) | IGHJ3 (Human) | ARPSRTRDPYGYGLGAFDF | |
| IGHV3-9 (Human) | IGHJ4 (Human) | AKDLGSGDGYNFSLDY | |
| IGHV4-39 (Human) | IGHJ6 (Human) | ASRRPPFYHDSHDMDV | |
| IGHV1-46 (Human) | IGHJ4 (Human) | ARDIAGVPAAIGCEY | |
| IGHV4-31 (Human) | IGHJ6 (Human) | ARESIRVVPGASPDYYGMDV | |
| IGHV4-4 (Human) | IGHJ3 (Human) | ARVALWGINLPSYFSRGFDI | |
| IGHV3-48 (Human) | IGHJ6 (Human) | ARGLGPKSYYYYGMDV | |
| IGHV4-59 (Human) | IGHJ5 (Human) | ARHYDSSGYTYNWFDP | |
| IGHV1-69 (Human) | IGHJ6 (Human) | ASPHCSSTSCLYHYYYYGMDV | |
| IGHV1-69 (Human) | IGHJ5 (Human) | ARTSYASFAGLGWFDP | |
| IGHV2-70 (Human) | IGHJ4 (Human) | ARIRATSGWYFFDY | |
| IGHV3-33 (Human) | IGHJ4 (Human) | ARDSDYGDSYNDY | |
| IGHV3-9 (Human) | IGHJ6 (Human) | AKDSGAAYYYYYMDV | |
| IGHV4-59 (Human) | IGHJ6 (Human) | ARGRRIWSKGHYYYYMDV | |
| IGHV5-51 (Human) | IGHJ4 (Human) | ARSDGYSYGYY | |
| IGHV1-69 (Human) | IGHJ3 (Human) | ARACTEWEPFDAFDI | |
| IGHV1-18 (Human) | IGHJ4 (Human) | ARGSRYYGSGSYYNVFDY | |
| IGHV1-69 (Human) | IGHJ3 (Human) | ARQGKRTYYDILTGSPAGAFDI | |
| IGHV3-23 (Human) | IGHJ2 (Human) | AKPPRGYYDSSGYYYEGWYFDL | |
| IGHV3-24 (Human) | IGHJ3 (Human) | AKPPRGYYDSSGYYYEGWYFDL | |
| IGHV3-25 (Human) | IGHJ4 (Human) | AKSPRGYYDSSGYYYEGWYFDL | |
| IGHV3-26 (Human) | IGHJ5 (Human) | AKPPRGYYDSSGYYYEGWYFDL | |
| IGHV3-9 (Human) | IGHJ6 (Human) | AKDRGHDYGDYVQLD | |
| IGHV3-9 (Human) | IGHJ3 (Human) | AKAGHSSGWEGGPDAFDI | |
| IGHV4-31 (Human) | IGHJ4 (Human) | ARVIRQLWLRFDY | |
| IGHV1-2 (Human) | IGHJ3 (Human) | ARHYGDYGEDAFDI | |
| IGHV1-3 (Human) | IGHJ4 (Human) | ARHYGDYGEDAFDI | |
| IGHV1-4 (Human) | IGHJ5 (Human) | ARHYGDYGEDAFDI | |
| IGHV1-5 (Human) | IGHJ6 (Human) | ARHYGDYGEDAFDI | |
| IGHV1-69 (Human) | IGHJ6 (Human) | ARAGATRLYYYYYMDV | |
| IGHV3-30-3 (Human) | IGHJ4 (Human) | ARDQEYYDILTGASGGFDY | |
| IGHV3-33 (Human) | IGHJ4 (Human) | ARDVSSGWSPGDFDY | |
| IGHV3-49 (Human) | IGHJ4 (Human) | TRVGITFGGVIVHPFDY | |
| IGHV3-53 (Human) | IGHJ5 (Human) | ARDLRGIT | |
| IGHV3-9 (Human) | IGHJ4 (Human) | AKGQRFRELLIDY | |
| IGHV4-39 (Human) | IGHJ3 (Human) | ARRDIVVVVAATDDAFDI | |
| IGHV5-51 (Human) | IGHJ3 (Human) | ARALGGYKLDAFDI | |
| IGHV1-69 (Human) | IGHJ5 (Human) | ARVVSNWFDP | |
| IGHV3-21 (Human) | IGHJ5 (Human) | AMGAMSSSWPTNWFDP | |
| IGHV3-23 (Human) | IGHJ4 (Human) | AKDSLFCPQCSPAYYFDY | |
| IGHV3-30-3 (Human) | IGHJ4 (Human) | ARDFRGGLVGGGSCLDY | |
| IGHV3-66 (Human) | IGHJ4 (Human) | ARGYKSHY | |
| IGHV4-4 (Human) | IGHJ4 (Human) | ARSGTYYYDSSGYYQASHFDY | |
| IGHV5-10-1 (Human) | IGHJ6 (Human) | ARQAEEDISAESRRRGVYYYYYGMDV | |
| IGHV3-21 (Human) | IGHJ4 (Human) | ARDFSGHTAWAGTGFEY | |
| IGHV1-18 (Human) | IGHJ4 (Human) | ARDYTRGAWFGESLIGGFDN | |
| IGHV3-66 (Human) | IGHJ4 (Human) | AAPKVGLGPRTALGHLAFMTLPALNY | |
| IGHV7-4-1 (Human) | IGHJ6 (Human) | SSEITTLGGMDV | |
| IGHV4-61 (Human) | IGHJ6 (Human) | AREYSSYYYFYYMDV | |
| IGHV3-23 (Human) | IGHJ4 (Human) | ARDKILWFGETTYYFDY | |
| IGHV5-51 (Human) | IGHJ4 (Human) | ARGDYGDYGEFDY | |
| IGHV3-21 (Human) | IGHJ4 (Human) | ARPSGADYYDSSGDYDLRY | |
| IGHV3-23 (Human) | IGHJ6 (Human) | AKSQGDDSSGYYPLYYYYYGMDV | |
| IGHV5-51 (Human) | IGHJ4 (Human) | ARVGSYQPSFDY | |
| IGHV1-69 (Human) | IGHJ6 (Human) | ARDIDSSGYYYGTYYYYGMDV | |
| IGHV3-30 (Human) | IGHJ1 (Human) | AGEPYLRGYCRGGSCYGPSAGYFQH | |
| IGHV3-23 (Human) | IGHJ4 (Human) | ARALYGPPGHAIWFGELLEPGFDY | |
| IGHV3-23 (Human) | IGHJ3 (Human) | AAVGAAAGTGAFDI | |
| IGHV4-39 (Human) | IGHJ3 (Human) | VKSQWLRGAFDI | |
| IGHV1-69 (Human) | IGHJ4 (Human) | ALPLGQPYYFDY | |
| IGHV3-30 (Human) | IGHJ6 (Human) | AKDKLTGYLYYYYGMDV | |
| IGHV1-69 (Human) | IGHJ6 (Human) | AREAGYMGYYYYYMDV | |
| IGHV1-69 (Human) | IGHJ3 (Human) | ARDGTKGGPAFNAFDI | |
| IGHV1-69 (Human) | IGHJ5 (Human) | ARDPTRDCSGGSCYSGWFDP | |
| IGHV1-69 (Human) | IGHJ5 (Human) | AREVDTAMVIAGDDAFDI | |
| IGHV1-2 (Human) | IGHJ3 (Human) | ARVTHSSSWADAFDI | |
| IGHV4-4 (Human) | IGHJ4 (Human) | ARLTSNDY | |
| IGHV1-69 (Human) | IGHJ4 (Human) | ARDPAYCGGDCYSVPWVK | |
| IGHV5-51 (Human) | IGHJ3 (Human) | ARPYLRYFDYDAFDI | |
| IGHV3-30 (Human) | IGHJ6 (Human) | ALRGYSYGSIKYGMDV | |
| IGHV3-30 (Human) | IGHJ6 (Human) | ARARGYDSSGYYFFYYYYGMDV | |
| IGHV3-9 (Human) | IGHJ3 (Human) | AKGQLWGGYDAFDI | |
| IGHV3-9 (Human) | IGHJ6 (Human) | AKPIFVRGYSGYDSLEYGMDV | |
| IGHV1-69 (Human) | IGHJ3 (Human) | ARGWVYSSGWYDAFDI | |
| IGHV1-46 (Human) | IGHJ4 (Human) | ARETSLWGLGY | |
| IGHV1-69 (Human) | IGHJ5 (Human) | ATSPQWLLYNWFDP | |
| IGHV3-23 (Human) | IGHJ5 (Human) | AKSRQLAFDP | |
| IGHV5-51 (Human) | IGHJ3 (Human) | ARGGYSSGFDI | |
| IGHV1-69 (Human) | IGHJ4 (Human) | AWGILDY | |
| IGHV1-69 (Human) | IGHJ6 (Human) | ARDRYYYDSSGYYYDYYYGMDV | |
| IGHV3-9 (Human) | IGHJ4 (Human) | AKDIYSTYGAHFDY | |
| IGHV3-30 (Human) | IGHJ6 (Human) | ARALTGGYYYGMDV | |
| IGHV1-69 (Human) | IGHJ4 (Human) | AREELGCSSTSCALIDY | |
| IGHV3-21 (Human) | IGHJ4 (Human) | ARPSGADYYDSSGDYDLRY | |
| IGHV3-20 (Human) | IGHJ4 (Human) | ARGSGGLDY | |
| IGHV1-69 (Human) | IGHJ6 (Human) | ARDDIGYCSSTSCSYYYYGMDV | |
| IGHV1-2 (Human) | IGHJ4 (Human) | ARGKIDY | |
| IGHV3-30 (Human) | IGHJ4 (Human) | AKDRGNGWSSFDY | |
| IGHV3-21 (Human) | IGHJ4 (Human) | ARAGHRFLEWLPSYIDY | |
| IGHV3-53 (Human) | IGHJ6 (Human) | AKIDHYYGMDV | |
| IGHV1-46 (Human) | IGHJ6 (Human) | ARDLTEDYGDYARIYYYYGMDV | |
| IGHV3-30 (Human) | IGHJ4 (Human) | ARPRGGGYNFQWDY | |
| IGHV1-46 (Human) | IGHJ6 (Human) | ARGGENYDFWSGYSISYYYYGMDV | |
| IGHV1-18 (Human) | IGHJ4 (Human) | ARDRGLNWWLQFDGKDY | |
| IGHV3-30 (Human) | IGHJ5 (Human) | ARAFRGSYFSGIDP | |
| IGHV3-48 (Human) | IGHJ3 (Human) | ARGGNEYCSGSRCYQDAFDT | |
| IGHV3-30 (Human) | IGHJ6 (Human) | ARALYGTYYYGMDV | |
| IGHV3-66 (Human) | IGHJ4 (Human) | ATAAYYYDSSYYQY | |
| IGHV3-30 (Human) | IGHJ4 (Human) | ARDSWGDY | |
| IGHV3-30 (Human) | IGHJ4 (Human) | ARAKGGYYYFDY | |
| IGHV3-21 (Human) | IGHJ4 (Human) | ARAWPRFLEWLAPLFDY | |
| IGHV1-69D (Human) | IGHJ4 (Human) | ARVTRIPADFGLGFDY | |
| IGHV3-23 (Human) | IGHJ4 (Human) | AKDQGYDYVWGSYRPTPCFDY | |
| IGHV1-69D (Human) | IGHJ6 (Human) | ARARDFWSGYYPPYYYYYMDV | |
| IGHV3-30 (Human) | IGHJ4 (Human) | ARGGGGSYTYFDY | |
| IGHV5-51 (Human) | IGHJ4 (Human) | ARYGYSYGSDY | |
| IGHV3-53 (Human) | IGHJ6 (Human) | AKIDHYYGMDV | |
| IGHV1-69D (Human) | IGHJ3 (Human) | AREGEIKYYDSSGYWSDAFDI | |
| IGHV3-21 (Human) | IGHJ3 (Human) | TRGDAFDI | |
| IGHV3-66 (Human) | IGHJ1 (Human) | TTDEWLAFQH | |
| IGHV1-2 (Human) | IGHJ4 (Human) | ARGKIDY | |
| IGHV3-23 (Human) | IGHJ4 (Human) | ASSLWLAFDY | |
| IGHV3-9 (Human) | IGHJ4 (Human) | AKDFDTIWFGEFQPPDY | |
| IGHV3-30 (Human) | IGHJ6 (Human) | AREYDFWSGYSPESEAYYYYMDV | |
| IGHV3-23 (Human) | IGHJ3 (Human) | ARVSLLAFDI | |
| IGHV3-23 (Human) | IGHJ3 (Human) | AAVGAAAGTGAFDI | |
| IGHV1-2 (Human) | IGHJ6 (Human) | ARSPVADPWELLRATYYYYGMDV | |
| IGHV3-30 (Human) | IGHJ5 (Human) | TKCGSPYCSGGSCYSCWFDP | |
| IGHV1-69D (Human) | IGHJ6 (Human) | VRGLYASGNFRGSDFFYGMDV | |
| IGHV3-30 (Human) | IGHJ4 (Human) | AKVQDPYCSPTNCYTSNYYFDD | |
| IGHV3-30 (Human) | IGHJ3 (Human) | ARASSGSYFSAFDI | |
| IGHV4-31 (Human) | IGHJ6 (Human) | AGRRITMVRGVITPHYYYYDMDV | |
| IGHV1-69D (Human) | IGHJ1 (Human) | ARGYSYDNNGWAH | |
| IGHV3-30 (Human) | IGHJ4 (Human) | AKGGYGYGYQAYYFDY | |
| IGHV3-53 (Human) | IGHJ3 (Human) | ARGGRYDYDVFDI | |
| IGHV1-58 (Human) | IGHJ3 (Human) | AAVSCGVRGRFVCMDGFDI | |
| IGHV3-53 (Human) | IGHJ6 (Human) | ARDLDYYGMDV | |
| IGHV3-30 (Human) | IGHJ4 (Human) | ARDSEDCSSLSCYLDF | |
| IGHV3-66 (Human) | IGHJ6 (Human) | ARDPIQLWPYYYYYYYMDV | |
| IGHV4-31 (Human) | IGHJ4 (Human) | ARDYGGNSNYFGY | |
| IGHV5-10-1 (Human) | IGHJ4 (Human) | ARLGDYDFWSGYYTIDY | |
| IGHV1-69D (Human) | IGHJ6 (Human) | ARVGAPIERTSSSWHYYYYGMDV | |
| IGHV3-30 (Human) | IGHJ6 (Human) | AKEMATYYHYYYGLDV | |
| IGHV3-23 (Human) | IGHJ6 (Human) | AKDPSPTNYYYYYYMDV | |
| IGHV4-39 (Human) | IGHJ4 (Human) | ASASKRGYSGYDFISPYYFDY | |
| IGHV3-53 (Human) | IGHJ4 (Human) | ARDYGDFYFDY | |
| IGHV3-49 (Human) | IGHJ4 (Human) | TRGMEFYDILTGNHPYFFDY | |
| IGHV1-46 (Human) | IGHJ4 (Human) | ARDIVVVPAATGVDY | |
| IGHV3-23 (Human) | IGHJ5 (Human) | AKGVVPEAIPWFDP | |
| IGHV1-69D (Human) | IGHJ6 (Human) | VRGLYASGSYRGSDFFYGMDV | |
| IGHV1-3 (Human) | IGHJ4 (Human) | ARGLYYYDRIGSQSTEDYFDY | |
| IGHV4-31 (Human) | IGHJ5 (Human) | ARVSDFDWFDP | |
| IGHV3-13 (Human) | IGHJ6 (Human) | ARGKQQLVRAYYYYYMDV | |
| IGHV3-13 (Human) | IGHJ6 (Human) | ARGTHIYHHERERGYYYYYMDV | |
| IGHV3-33 (Human) | IGHJ5 (Human) | ARATMAAIRGGWFDP | |
| IGHV3-30 (Human) | IGHJ5 (Human) | ARDATTVTILVSWFDP | |
| IGHV3-13 (Human) | IGHJ6 (Human) | ARGRAIQLWLYYYYYMDV | |
| IGHV1-46 (Human) | IGHJ3 (Human) | ARDLVLVPAANAFDI | |
| IGHV3-30 (Human) | IGHJ4 (Human) | AREFYRGIDY | |
| IGHV1-46 (Human) | IGHJ4 (Human) | ARDIVAKRKWGLLGLIDY | |
| IGHV3-11 (Human) | IGHJ4 (Human) | ARPEKDIVVIPTV | |
| IGHV3-7 (Human) | IGHJ4 (Human) | ARDLEDPYGDFAGTFGY | |
| IGHV1-2 (Human) | IGHJ6 (Human) | ARAGSTSCYDPILDPCYYYMDV | |
| IGHV3-7 (Human) | IGHJ6 (Human) | ARDQVVAVPAAMGGFDYYYGMDV | |
| IGHV4-4 (Human) | IGHJ5 (Human) | ARPRQGGGWFDP | |
| IGHV1-24 (Human) | IGHJ6 (Human) | ATGFAVAGTSEAYYYHYGMDV | |
| IGHV4-59 (Human) | IGHJ5 (Human) | ARGFDP | |
| IGHV1-46 (Human) | IGHJ3 (Human) | ASKSESGYSYAFDI | |
| IGHV1-69D (Human) | IGHJ4 (Human) | ARVYYYDSSGYYYDY | |
| IGHV1-24 (Human) | IGHJ6 (Human) | ATGVAVAGTPSSYYYYYYMDV | |
| IGHV1-69D (Human) | IGHJ4 (Human) | ANNLPFANCSRTSCYYFDY | |
| IGHV1-3 (Human) | IGHJ6 (Human) | ARDTQIADVWGRAFLTGTPFGRGWFDP | |
| IGHV1-69D (Human) | IGHJ4 (Human) | ARTTHYDSSGYYRDY | |
| IGHV5-51 (Human) | IGHJ3 (Human) | ASHGGGGYDYVGGAFDI | |
| IGHV3-53 (Human) | IGHJ6 (Human) | ARDLVARGMDV | |
| IGHV3-7 (Human) | IGHJ4 (Human) | ARVKINPYYDYVWGSYRYSRILDY | |
| IGHV5-10-1 (Human) | IGHJ6 (Human) | ARHETKAKYSSGWYHYYYYGMDV | |
| IGHV3-15 (Human) | IGHJ3 (Human) | TTGSFYYDSSAYVNDAFEI | |
| IGHV5-10-1 (Human) | IGHJ5 (Human) | ARGGIRSIAVAYEMTAPLYRGFDP | |
| IGHV3-21 (Human) | IGHJ6 (Human) | ARDPSLHRFFRYYYIDV | |
| IGHV3-30 (Human) | IGHJ4 (Human) | AKDRAGSYSRLFDY | |
| IGHV3-48 (Human) | IGHJ6 (Human) | ASELGTDYYYYYGMDV | |
| IGHV4-59 (Human) | IGHJ6 (Human) | ARDREGGYCSGGRCYSSHYMDV | |
| IGHV5-10-1 (Human) | IGHJ4 (Human) | ARHELVCSGGSCYLEEYFQY | |
| IGHV3-30 (Human) | IGHJ4 (Human) | ARDGAQLRFLEWLQPAESCYFDY | |
| IGHV3-30 (Human) | IGHJ3 (Human) | ARVDCTSTSCPGYMAFDV | |
| IGHV3-21 (Human) | IGHJ4 (Human) | ARSPEWYSSGWYWRELDY | |
| IGHV3-30 (Human) | IGHJ6 (Human) | ARDLGIVRFLEWLAPQENYGMDV | |
| IGHV3-23 (Human) | IGHJ4 (Human) | AKGRYYDFWSRYDVPFDY | |
| IGHV3-15 (Human) | IGHJ4 (Human) | TTGSGLGSYPAFDY | |
| IGHV3-30 (Human) | IGHJ6 (Human) | ARDRSGNYYYGMDV | |
| IGHV1-69D (Human) | IGHJ6 (Human) | ARDKGYDNTYYHYMDV | |
| IGHV1-58 (Human) | IGHJ3 (Human) | AAPYCSGGSCHDGFDI | |
| IGHV3-30 (Human) | IGHJ5 (Human) | ARDHGVDTVMVTWFDP | |
| IGHV4-4 (Human) | IGHJ5 (Human) | ARDLVWESAVAGTGFVSWIDP | |
| IGHV3-30 (Human) | IGHJ4 (Human) | ARDQGGSYDYVGGSYRLGPTYFDY | |
| IGHV4-59 (Human) | IGHJ4 (Human) | TRGFDY | |
| IGHV1-69D (Human) | IGHJ5 (Human) | ARDLWVLRFLEWFPGWFDP | |
| IGHV1-46 (Human) | IGHJ6 (Human) | ARGGATPHLRGGMDV | |
| IGHV3-53 (Human) | IGHJ4 (Human) | VGGYSSSVR | |
| IGHV5-10-1 (Human) | IGHJ6 (Human) | ARQGRWLVPEAEGAWDYNYMDV | |
| IGHV1-69D (Human) | IGHJ4 (Human) | ASFGGDDGLDK | |
| IGHV3-30 (Human) | IGHJ4 (Human) | ARDRSGGYSYNFDY | |
| IGHV3-30 (Human) | IGHJ6 (Human) | AKDWDDAYYFYYYMDI | |
| IGHV1-24 (Human) | IGHJ6 (Human) | ATLAYCTNGVCYILGPPPLRKHEELYYFYMDV | |
| IGHV3-15 (Human) | IGHJ3 (Human) | TTTVSGWFWGDAFDI | |
| IGHV1-46 (Human) | IGHJ3 (Human) | ARDEVLIPAARAFDI | |
| IGHV3-30 (Human) | IGHJ4 (Human) | AKSVGEYCGGGSCYSYFDY | |
| IGHV1-69D (Human) | IGHJ4 (Human) | ARLGRGDYDSSGYYKVYFDY | |
| IGHV3-53 (Human) | IGHJ3 (Human) | ARDQGEDVFDI | |
| IGHV3-30 (Human) | IGHJ4 (Human) | AKDLYYYDTGAPYYFDY | |
| IGHV3-30-3 (Human) | IGHJ4 (Human) | AKQLGQYCSGGNCYSGYFDY | |
| IGHV3-33 (Human) | IGHJ4 (Human) | VKGGWYGDSLRVDY | |
| IGHV3-33 (Human) | IGHJ6 (Human) | AREWHHGSGEGYMDV | |
| IGHV4-34 (Human) | IGHJ5 (Human) | SRGVVLNNVVWFDP | |
| IGHV3-66 (Human) | IGHJ3 (Human) | ARNIYDAFDI | |
| IGHV3-66 (Human) | IGHJ4 (Human) | ASPGIVPAAMGVFDY | |
| IGHV3-53 (Human) | IGHJ4 (Human) | ARLPYGDPA | |
| IGHV1-24 (Human) | IGHJ6 (Human) | AASFAVSGTPGPPSHYNYYYGMDV | |
| IGHV3-21 (Human) | IGHJ6 (Human) | ARDPTLHRFFQYYYMDV | |
| IGHV1-24 (Human) | IGHJ6 (Human) | ATGWSITGTPSNYKYYYGMDV | |
| IGHV1-24 (Human) | IGHJ6 (Human) | ATSTAVRGERPGGYNYYYGMDV | |
| IGHV1-69D (Human) | IGHJ4 (Human) | ARLGRGDYDSSGYYKVYFDY | |
| IGHV4-4 (Human) | IGHJ5 (Human) | ARDRSGGPHGGFDP | |
| IGHV1-46 (Human) | IGHJ6 (Human) | ARLVVVSSEYSSSSGYYYYYFMDV | |
| IGHV4-39 (Human) | IGHJ6 (Human) | ATDSPVVGAAAPEHMFYSYYYMDF | |
| IGHV3-21 (Human) | IGHJ6 (Human) | ASILEWFAGYMDV | |
| IGHV1-58 (Human) | IGHJ3 (Human) | AAPYCYSTSCADGFDI | |
| IGHV1-58 (Human) | IGHJ3 (Human) | AAPYCNKTRCSDGFDI | |
| IGHV3-30 (Human) | IGHJ4 (Human) | AREELRALADY | |
| IGHV1-24 (Human) | IGHJ5 (Human) | TTQGSGPFGEPRGWFDP | |
| IGHV4-30-4 (Human) | IGHJ4 (Human) | ARVTGVSAAGYYFDY | |
| IGHV1-58 (Human) | IGHJ3 (Human) | AANNCGRTTCSDAFDI | |
| IGHV1-58 (Human) | IGHJ3 (Human) | AAPYCGGDCNDGFDV | |
| IGHV5-10-1 (Human) | IGHJ5 (Human) | AKHKFFGELPIRGFDP | |
| IGHV1-69D (Human) | IGHJ6 (Human) | ARTYSTYPKNRLDWDYYYYYGMDV | |
| IGHV3-15 (Human) | IGHJ4 (Human) | TTDPGWWRIAVAGTNY | |
| IGHV3-30 (Human) | IGHJ4 (Human) | ARDSEDCSSLSCYLDY | |
| IGHV1-58 (Human) | IGHJ3 (Human) | AAPYCNTTSCDDGFDI | |
| IGHV3-7 (Human) | IGHJ5 (Human) | AADLGILWFGDLRKSEP | |
| IGHV1-58 (Human) | IGHJ3 (Human) | AAPNCNRTICADGFDI | |
| IGHV3-15 (Human) | IGHJ4 (Human) | TTEGVQSSGWYLSFDF | |
| IGHV1-58 (Human) | IGHJ3 (Human) | AAPGCNTTICPDGFDI | |
| IGHV5-10-1 (Human) | IGHJ6 (Human) | ARENFWSVYYTGIDYYMDV | |
| IGHV4-39 (Human) | IGHJ2 (Human) | ARRGWLRGYFDL | |
| IGHV4-39 (Human) | IGHJ5 (Human) | ATQLWLRSNFDS | |
| IGHV3-13 (Human) | IGHJ2 (Human) | ARDLTQDWYFDL | |
| IGHV3-15 (Human) | IGHJ3 (Human) | TTDKARNYYDSSGYEHDAFDI | |
| IGHV1-58 (Human) | IGHJ3 (Human) | AAPYCSGGTCHDGFDI | |
| IGHV4-39 (Human) | IGHJ3 (Human) | ARQSSPKLGDDAFDI | |
| IGHV3-53 (Human) | IGHJ3 (Human) | ARGKWLRGAFDI | |
| IGHV3-66 (Human) | IGHJ6 (Human) | ARDLAVYGMDV | |
| IGHV4-4 (Human) | IGHJ4 (Human) | ASAPGTPWFDY | |
| IGHV1-58 (Human) | IGHJ3 (Human) | AAPYCSGGSCFDAFDM | |
| IGHV4-31 (Human) | IGHJ4 (Human) | ARDYGGNQNYFGY | |
| IGHV3-53 (Human) | IGHJ3 (Human) | ARDLHSSGPFDAFDI | |
| IGHV4-38-2 (Human) | IGHJ4 (Human) | ARDDYGDYAVSY | |
| IGHV3-11 (Human) | IGHJ4 (Human) | ARELLLLGYCSGGSCYPVGPDY | |
| IGHV1-18 (Human) | IGHJ4 (Human) | ARDYDYVWGSYPSACCY | |
| IGHV3-53 (Human) | IGHJ6 (Human) | ARDLSVAGGMDV | |
| IGHV3-53 (Human) | IGHJ6 (Human) | ARWAGADGMDV | |
| IGHV3-23 (Human) | IGHJ4 (Human) | AKDFVVVVAARSHDDYYFDY | |
| IGHV1-69D (Human) | IGHJ4 (Human) | ATQGGRFYCSGGSCYRYYFDY | |
| IGHV1-58 (Human) | IGHJ3 (Human) | AAPHCGGGSCYDGFDI | |
| IGHV3-13 (Human) | IGHJ2 (Human) | ARGGGDGYNLGLWYFDL | |
| IGHV3-64D (Human) | IGHJ4 (Human) | VKDVTVDTAMVTIFDN | |
| IGHV3-15 (Human) | IGHJ4 (Human) | TTDPGYTYSPAY | |
| IGHV3-9 (Human) | IGHJ6 (Human) | AKDAFGDPQGLYGMDV | |
| IGHV3-30 (Human) | IGHJ4 (Human) | AKVLGSYCSGGSCYGGSFDY | |
| IGHV4-59 (Human) | IGHJ5 (Human) | ARSRGYNYGLGLGWFDP | |
| IGHV4-39 (Human) | IGHJ1 (Human) | ARHTGYYDSSGYYRLEYFQH | |
| IGHV1-58 (Human) | IGHJ3 (Human) | AAPNCSGGSCYDAFDI | |
| IGHV3-48 (Human) | IGHJ4 (Human) | ARQPREYYDFWSGYRRLFYFDY | |
| IGHV3-23 (Human) | IGHJ5 (Human) | AKGTQPIPDYGDFFDP | |
| IGHV3-30 (Human) | IGHJ4 (Human) | AKGGGWYDYKGYYFDY | |
| IGHV1-46 (Human) | IGHJ5 (Human) | ARGGIVPAATLLFDP | |
| IGHV1-58 (Human) | IGHJ3 (Human) | AAPYCSGSSCLDGFDI | |
| IGHV3-23 (Human) | IGHJ3 (Human) | AKDTYYDIFPDVFDI | |
| IGHV3-13 (Human) | IGHJ6 (Human) | ARGHFYGLIGYMDV | |
| IGHV4-39 (Human) | IGHJ4 (Human) | ARFTRFAGLYYFDY | |
| IGHV3-33 (Human) | IGHJ3 (Human) | ARDRFYDYSSSGYSLDAFDI | |
| IGHV3-30 (Human) | IGHJ4 (Human) | AKDPPQFAVAGTGYFDY | |
| IGHV3-30 (Human) | IGHJ4 (Human) | AKAVYSYAYAVLYFDY | |
| IGHV3-13 (Human) | IGHJ2 (Human) | ARGGAVIPVWYFDL | |
| IGHV1-69D (Human) | IGHJ5 (Human) | ARREYSSTDWFDP | |
| IGHV3-66 (Human) | IGHJ3 (Human) | ARWGRVGATGLAFDI | |
| IGHV1-58 (Human) | IGHJ3 (Human) | AAPACSSTRCYDGFDI | |
| IGHV3-53 (Human) | IGHJ6 (Human) | ARDLITYGMDV | |
| IGHV3-7 (Human) | IGHJ3 (Human) | TRAGWVRGAFDI | |
| IGHV3-66 (Human) | IGHJ4 (Human) | ARDRGDYLFDY | |
| IGHV3-66 (Human) | IGHJ6 (Human) | ARDAVYYGMDV | |
| IGHV1-2 (Human) | IGHJ4 (Human) | ARSYYDY | |
| IGHV3-23 (Human) | IGHJ3 (Human) | AKVGWGAFDI | |
| IGHV3-15 (Human) | IGHJ3 (Human) | TTDLCRSTSCEHDAFDI | |
| IGHV1-46 (Human) | IGHJ6 (Human) | AKDRVTIFWGNGMDV | |
| IGHV4-34 (Human) | IGHJ5 (Human) | VGGVVLDNVVWFDP | |
| IGHV3-11 (Human) | IGHJ6 (Human) | ARSPPTGDSSDWYDSPAYNNYYMDV | |
| IGHV1-58 (Human) | IGHJ3 (Human) | AAPNCSRTLCYDGFNM | |
| IGHV3-53 (Human) | IGHJ6 (Human) | ARVGGYCSSANCVSDV | |
| IGHV1-58 (Human) | IGHJ3 (Human) | AAPNCSGGSCYDGFDL | |
| IGHV3-23 (Human) | IGHJ4 (Human) | ARDLWGSGFFAFDV | |
| IGHV3-23 (Human) | IGHJ4 (Human) | ARGRDLAAFTKTAFDV | |
| IGHV3-23 (Human) | IGHJ4 (Human) | ARDRWASGWLAFDV | |
| IGHV3-23 (Human) | IGHJ4 (Human) | ARDRDHAYDWGFDV | |
| IGHV3-15 (Human) | IGHJ4 (Human) | TTAGSYYYDTVGPGLPEGKFDY | |
| IGHV1-58 (Human) | IGHJ3 (Human) | AAPYCSSISCNDGFDI | |
| IGHV3-33 (Human) | IGHJ6 (Human) | ARETVSYGMDV | |
| IGHV4-59 (Human) | IGHJ3 (Human) | ARDRGYSSGWTDGFDI | |
| IGHV1-2 (Human) | IGHJ6 (Human) | ARGLGVGCSGGNCYLDYYYMDV | |
| IGHV1-24 (Human) | IGHJ6 (Human) | ATAPAVAGPFYYYYYGMDV | |
| IGHV1-24 (Human) | IGHJ6 (Human) | ATAPAVAGPFYYYYYGMDV | |
| IGHV1-24 (Human) | IGHJ6 (Human) | ATAPAVAGPFYYYYYGMDV | |
| IGHV3-33 (Human) | IGHJ6 (Human) | VRETVDGMDV | |
| IGHV1-24 (Human) | IGHJ6 (Human) | ATAPAVAGPLYYYYYGMDV | |
| IGHV1-24 (Human) | IGHJ6 (Human) | ATAPAVAGPFYYYYYGMDV | |
| IGHV3-33 (Human) | IGHJ6 (Human) | VRETVDGMDV | |
| IGHV3-33 (Human) | IGHJ6 (Human) | VRETVDGMDV | |
| IGHV1-24 (Human) | IGHJ6 (Human) | ATAPAVAGPFYNFYYGIDV | |
| IGHV1-2 (Human) | IGHJ3 (Human) | ARGGSRCSGGNCYGWAYDAFDI | |
| IGHV1-2 (Human) | IGHJ4 (Human) | ARAAPFYDFWSGYSYFDY | |
| IGHV3-21 (Human) | IGHJ4 (Human) | ARDGNAYKWLLAENVRFDY | |
| IGHV3-33 (Human) | IGHJ4 (Human) | ARAVAGEWYFDY | |
| IGHV3-33 (Human) | IGHJ4 (Human) | ARAFPDSSSWSGFTIDY | |
| IGHV1-2 (Human) | IGHJ2 (Human) | ARVPYCSSTSCHRDWYFDL | |
| IGHV1-69 (Human) | IGHJ4 (Human) | ARADYYYDSSGYFFDY | |
| IGHV5-51 (Human) | IGHJ4 (Human) | ARIRGVYSSGWIGGDY | |
| IGHV5-51 (Human) | IGHJ2 (Human) | ARGVAVDWYFDL | |
| IGHV3-53 (Human) | IGHJ4 (Human) | AREGDVEGFSDLWSGYSRDRYYFDY | |
| IGHV1-69 (Human) | IGHJ6 (Human) | ARREAYGPRDYYYYYGMDV | |
| IGHV1-46 (Human) | IGHJ4 (Human) | ARGSPKGAFDY | |
| IGHV1-46 (Human) | IGHJ6 (Human) | ARDREPHSDSSGYWDSLKYYYYYALDV | |
| IGHV1-69 (Human) | IGHJ4 (Human) | ARGFNGNYYGWGDDDAFDI | |
| IGHV3-30 (Human) | IGHJ4 (Human) | ASGSDYGDYLLVY | |
| IGHV3-53 (Human) | IGHJ6 (Human) | ARDLSEGGMDV | |
| IGHV1-69 (Human) | IGHJ6 (Human) | ATRKETTVTTSLVYGMDV | |
| IGHV1-2 (Human) | IGHJ6 (Human) | ARGPFYYDNSGTLGGLDV | |
| IGHV3-11 (Human) | IGHJ4 (Human) | ARDGVIPPRFDY | |
| IGHV1-2 (Human) | IGHJ3 (Human) | ARGPYYYDSSGSLGAFDI | |
| IGHV1-18 (Human) | IGHJ6 (Human) | AGSDNYGFPYNGMDV | |
| IGHV1-2 (Human) | IGHJ4 (Human) | ARDSRFSYVNGEFDY | |
| IGHV1-2 (Human) | IGHJ6 (Human) | ARVGWYDFGTPGDYYYYYGMDV | |
| IGHV1-2 (Human) | IGHJ6 (Human) | VRGPFYYDSSGPLGGMDV | |
| IGHV3-9 (Human) | IGHJ6 (Human) | AKDFLWDLHPPRYYGMDV | |
| IGHV3-53 (Human) | IGHJ4 (Human) | ARGGRLADAAGDY | |
| IGHV3-53 (Human) | IGHJ4 (Human) | ARGHYDLFDY | |
| IGHV3-53 (Human) | IGHJ6 (Human) | ARGDGWDNYYYGMDV | |
| IGHV3-66 (Human) | IGHJ4 (Human) | ARGFGDYYFDY | |
| IGHV1-2 (Human) | IGHJ4 (Human) | ARGPRYSGTHFDY | |
| IGHV1-2 (Human) | IGHJ4 (Human) | ARGPRYSGTYFDY | |
| IGHV1-2 (Human) | IGHJ4 (Human) | ARGPRYSGTYFDY | |
| IGHV3-53 (Human) | IGHJ4 (Human) | ARDPYGYSSIWDGQGGH | |
| IGHV3-21 (Human) | IGHJ6 (Human) | ARGGYCSDGSCYVQDRLIYYYSGLDV | |
| IGHV3-48 (Human) | IGHJ4 (Human) | ARDRRRRYCTNGVCYRPEEIDY | |
| IGHV3-33 (Human) | IGHJ4 (Human) | ARDPFPGAVAGTGYLQY | |
| IGHV3-30 (Human) | IGHJ6 (Human) | AKSSGSYYYYYYGMDV | |
| IGHV1-46 (Human) | IGHJ4 (Human) | ARLHCGGDCYLDY | |
| IGHV3-23 (Human) | IGHJ6 (Human) | AKGSGSGSYPNYYYYYGMDV | |
| IGHV3-53 (Human) | IGHJ4 (Human) | ARDYGDLYFDY | |
| IGHV3-30 (Human) | IGHJ6 (Human) | AKDQAYYDILTGYLNPPKNYYYYGMDV | |
| IGHV1-24 (Human) | IGHJ4 (Human) | ATAFSIFGVVPPDY | |
| IGHV4-39 (Human) | IGHJ4 (Human) | ARQGDCSTTSCAYDY | |
| IGHV3-30 (Human) | IGHJ6 (Human) | AKDRTGNYYYGMDV | |
| IGHV3-23 (Human) | IGHJ5 (Human) | AKDRYYEFWSGYSNWFDP | |
| IGHV5-51 (Human) | IGHJ4 (Human) | ARVNYYDSSGYPSFHFDY | |
| IGHV1-2 (Human) | IGHJ6 (Human) | AREMPAAMGYYYYGMDV | |
| IGHV3-23 (Human) | IGHJ4 (Human) | AKANKYSSSEFDF | |
| IGHV1-2 (Human) | IGHJ3 (Human) | ATESWVYGSGSYSSGAFDI | |
| IGHV1-2 (Human) | IGHJ4 (Human) | ARGPRYSGTYFDY | |
| IGHV1-2 (Human) | IGHJ4 (Human) | ARGPRYSGTYFDY | |
| IGHV1-2 (Human) | IGHJ4 (Human) | ARGPRYSGTYFDY | |
| IGHV1-2 (Human) | IGHJ4 (Human) | ARGPRYSGTYFDY | |
| IGHV7-4-1 (Human) | IGHJ5 (Human) | AVYYYDSGSPGWFDP | |
| IGHV1-69 (Human) | IGHJ5 (Human) | ARDFRYCSSTRCYFWFDP | |
| IGHV1-46 (Human) | IGHJ4 (Human) | ARWYDSTGSIDY | |
| IGHV3-9 (Human) | IGHJ4 (Human) | AKDQGYSYGNYFDY | |
| IGHV1-69 (Human) | IGHJ4 (Human) | ALRNQWDLLVY | |
| IGHV1-69 (Human) | IGHJ6 (Human) | ARDSEYSSSWYSRGYYGMDV | |
| IGHV3-7 (Human) | IGHJ4 (Human) | ARQPESTIWYYFDY | |
| IGHV1-58 (Human) | IGHJ3 (Human) | AAPYCSGGTCLDGFDI | |
| IGHV3-48 (Human) | IGHJ5 (Human) | AREAHDGALTDYGDYLNWFDP | |
| IGHV3-48 (Human) | IGHJ5 (Human) | AREAHDGALTDYGDYLNWFDP | |
| IGHV3-9 (Human) | IGHJ4 (Human) | AKAGVRGIAAAGPDLNFDY | |
| IGHV4-39 (Human) | IGHJ5 (Human) | ARRLRQLWFGPWFDP | |
| IGHV4-61 (Human) | IGHJ3 (Human) | ARETYYYDSSGYYISDAFDI | |
| IGHV4-61 (Human) | IGHJ3 (Human) | ARESFYYDRSGYYGSDAFDI | |
| IGHV3-53 (Human) | IGHJ4 (Human) | AREGDVEGLHDFWSGYSRDRYYFDY | |
| IGHV1-2 (Human) | IGHJ3 (Human) | ARGPLFHKLVYDSWSGYHDGFDI | |
| IGHV1-2 (Human) | IGHJ3 (Human) | ARGPLFHRLVYDFWSGYHDGFDM | |
| IGHV1-2 (Human) | IGHJ3 (Human) | ARGPLFHKLVYDSWTGYHDGFDI | |
| IGHV3-66 (Human) | IGHJ6 (Human) | ARIANYMDV | |
| IGHV3-21 (Human) | IGHJ4 (Human) | ARERGYYGGKTPPFL | |
| IGHV4-31 (Human) | IGHJ3 (Human) | ARVSTTVTTYLVGGFDI | |
| IGHV3-53 (Human) | IGHJ6 (Human) | ARDVGDYYGMDV | |
| IGHV3-30 (Human) | IGHJ3 (Human) | ARDMEVDYYDRSGHYHVFHAFDI | |
| IGHV3-33 (Human) | IGHJ6 (Human) | ARDHSSSSFVYYYYMDV | |
| IGHV4-39 (Human) | IGHJ4 (Human) | ARRSITLAGRDCLDF | |
| IGHV1-4 (Human) | IGHJ5 (Human) | AREGALTNWFDP | |
| IGHV4-34 (Human) | IGHJ4 (Human) | ARPGITATTGFDF | |
| IGHV3-15 (Human) | IGHJ4 (Human) | TTTNDYGDYSPAY | |
| IGHV3-21 (Human) | IGHJ4 (Human) | ARERYGDN | |
| IGHV4-59 (Human) | IGHJ5 (Human) | ARLKQQLVGFGWFDP | |
| IGHV4-59 (Human) | IGHJ5 (Human) | ARLKQQLVGFGWFDP | |
| IGHV1-46 (Human) | IGHJ5 (Human) | ARASTSTTSWSDALSLGS | |
| IGHV3-33 (Human) | IGHJ4 (Human) | AREQEANYYDISGYYHWGESLGY | |
| IGHV3-33 (Human) | IGHJ4 (Human) | AREQEANYYDISGYYHWGESLGY | |
| IGHV1-69 (Human) | IGHJ6 (Human) | ARDSGYSGYGSTYYMDV | |
| IGHV1-69 (Human) | IGHJ6 (Human) | ARDSGYSGYGSTYYMDV | |
| IGHV4-59 (Human) | IGHJ5 (Human) | ARHYDILTALSWFDP | |
| IGHV4-59 (Human) | IGHJ5 (Human) | ARHYDILTSLSWFDP | |
| IGHV3-30 (Human) | IGHJ4 (Human) | VKEGRPSDTVVVVAFDY | |
| IGHV3-15 (Human) | IGHJ4 (Human) | TTGPQYDDFGHSYIVDS | |
| IGHV4-31 (Human) | IGHJ4 (Human) | ARVWQYYDSTGSFDY | |
| IGHV3-21 (Human) | IGHJ4 (Human) | ARVQVGARGWVDY | |
| IGHV3-21 (Human) | IGHJ4 (Human) | ARVQVGARGWVDY | |
| IGHV1-69 (Human) | IGHJ3 (Human) | ARGVVGATPGSFDL | |
| IGHV4-4 (Human) | IGHJ5 (Human) | ARGDVLDWFDP | |
| IGHV4-59 (Human) | IGHJ4 (Human) | AAGLKGRSSSWYEY | |
| IGHV3-23 (Human) | IGHJ6 (Human) | AKNIAEMSTFDDYFYYYGMDV | |
| IGHV3-13 (Human) | IGHJ2 (Human) | ARMVYDSSGFKGYFDL | |
| IGHV3-30 (Human) | IGHJ4 (Human) | AKAALGYCTNGVCYCDN | |
| IGHV4-4 (Human) | IGHJ5 (Human) | AGSYSNYIGGVWFDP | |
| IGHV4-34 (Human) | IGHJ6 (Human) | ARGGFGVVINYYYSGMDV | |
| IGHV3-66 (Human) | IGHJ4 (Human) | ARDLVVYGLDC | |
| IGHV3-23 (Human) | IGHJ6 (Human) | ANHPLASGDEYYYYYMDV | |
| IGHV3-66 (Human) | IGHJ4 (Human) | ARDLAGRLDY | |
| IGHV3-66 (Human) | IGHJ4 (Human) | ARDLVVYGADY | |
| IGHV1-69 (Human) | IGHJ3 (Human) | ARGVVAATPGNFDI | |
| IGHV3-66 (Human) | IGHJ4 (Human) | ARDIAGRLDY | |
| IGHV3-53 (Human) | IGHJ6 (Human) | ARDLIALGVDV | |
| IGHV4-4 (Human) | IGHJ4 (Human) | GVCAGDCYAASVFDY | |
| IGHV3-66 (Human) | IGHJ6 (Human) | ANHGYYYYMDV | |
| IGHV3-53 (Human) | IGHJ4 (Human) | ARDRGGGILDY | |
| IGHV4-59 (Human) | IGHJ4 (Human) | ARGWPYCGVDCYSGFDY | |
| IGHV3-53 (Human) | IGHJ4 (Human) | AKTPRGDYDSSGTSAY | |
| IGHV3-9 (Human) | IGHJ4 (Human) | AKGLDSSSSASPDY | |
| IGHV1-46 (Human) | IGHJ4 (Human) | ARGGYCGSTSCSPDDYFDY | |
| IGHV3-33 (Human) | IGHJ6 (Human) | ARDGIPFRYGMDV | |
| IGHV3-13 (Human) | IGHJ2 (Human) | VRDREISGWTGWYFDL | |
| IGHV3-23 (Human) | IGHJ3 (Human) | AKVLSPTYYDSWSGPDAFDF | |
| IGHV3-33 (Human) | IGHJ4 (Human) | ARGGRPDHETGIAVLGEYYFDS | |
| IGHV3-9 (Human) | IGHJ4 (Human) | VKGYRYYYDILTGYYNDAGAFDY | |
| IGHV5-51 (Human) | IGHJ5 (Human) | ARLSERWYSPFDS | |
| IGHV3-15 (Human) | IGHJ3 (Human) | TTDCFWRLGGTTCYEHDAFDV | |
| IGHV1-69 (Human) | IGHJ6 (Human) | AREDFILVSAPIRENSYYYYGMDV | |
| IGHV3-9 (Human) | IGHJ4 (Human) | ARGLDGSSSASPDS | |
| IGHV3-49 (Human) | IGHJ3 (Human) | SRGGYYDGSPYYWNRPDAFDI | |
| IGHV4-39 (Human) | IGHJ3 (Human) | ARLETSGWYTEDVFDI | |
| IGHV4-39 (Human) | IGHJ4 (Human) | ARQHRYGSGSSELL | |
| IGHV3-9 (Human) | IGHJ4 (Human) | AKGVEYSSSSNCDY | |
| IGHV3-13 (Human) | IGHJ2 (Human) | VRDREISGWTGWYFDL | |
| IGHV3-66 (Human) | IGHJ4 (Human) | ARETLGRGGDC | |
| IGHV3-30 (Human) | IGHJ6 (Human) | AKDTPGGDDIMTGWGLYGMDV | |
| IGHV1-8 (Human) | IGHJ2 (Human) | ARGFSLTWYFDL | |
| IGHV3-21 (Human) | IGHJ4 (Human) | TRVQVGARGWADY | |
| IGHV3-23 (Human) | IGHJ5 (Human) | ATERIAVSDTRMYNWFDP | |
| IGHV3-30 (Human) | IGHJ6 (Human) | AKDTPGGDDILTGWGLYGMDV | |
| IGHV1-69 (Human) | IGHJ6 (Human) | ARLSDRWYSPFDP | |
| IGHV1-69 (Human) | IGHJ6 (Human) | ARAGTTNSDYFDY | |
| IGHV5-51 (Human) | IGHJ5 (Human) | ARAGTTNSDYFDY | |
| IGHV3-30 (Human) | IGHJ4 (Human) | TTDDPGSYYYGMDV | |
| IGHV3-30 (Human) | IGHJ4 (Human) | TTDDPGSYYYGMDV | |
| IGHV3-15 (Human) | IGHJ6 (Human) | ARPLLPGETGSLNRLDY | |
| IGHV1-46 (Human) | IGHJ4 (Human) | ARVPSSSDYGDYGGFEY | |
| IGHV1-46 (Human) | IGHJ4 (Human) | ARVPSSSDYGDYGGFEY | |
| IGHV3-9 (Human) | IGHJ3 (Human) | AKIADIVRAYDFWSGQHFDAFDI | |
| IGHV3-9 (Human) | IGHJ3 (Human) | AKIADLVGAYDFRSGQHFAAFDV | |
| IGHV3-33 (Human) | IGHJ4 (Human) | ARDSNVDTVMVTWFDY | |
| IGHV3-15 (Human) | IGHJ3 (Human) | STVGSYYYDSRGPTSDAFDI | |
| IGHV3-23 (Human) | IGHJ5 (Human) | ATERIAVAGTRMYNWFDP | |
| IGHV3-53 (Human) | IGHJ3 (Human) | ARDLSVVGAFDI | |
| IGHV4-31 (Human) | IGHJ5 (Human) | ARAIVVVTLNWFDL | |
| IGHV4-61 (Human) | IGHJ4 (Human) | ATGSKSSYYFDY | |
| IGHV1-46 (Human) | IGHJ4 (Human) | ARDEAFLPSAIFVGDY | |
| IGHV3-30 (Human) | IGHJ4 (Human) | AKGGPYGDHVRSDY | |
| IGHV4-59 (Human) | IGHJ6 (Human) | ATYYFDNSGYSYGLDV | |
| IGHV3-53 (Human) | IGHJ4 (Human) | ARGDGELIFDQ | |
| IGHV3-49 (Human) | IGHJ4 (Human) | TRWDGWSQHDY | |
| IGHV4-4 (Human) | IGHJ3 (Human) | ARDGGRPGDAFDL | |
| IGHV1-18 (Human) | IGHJ4 (Human) | ARAIAVAGTSGEFDY | |
| IGHV1-18 (Human) | IGHJ4 (Human) | ARAMAVAGTSGDFDY | |
| IGHV1-69 (Human) | IGHJ4 (Human) | ASFHVAYGDYIPFDY | |
| IGHV1-69 (Human) | IGHJ4 (Human) | ATFHVAYGDYIPFDS | |
| IGHV3-30 (Human) | IGHJ6 (Human) | AKDPLPFRDFFYYYMDV | |
| IGHV3-30 (Human) | IGHJ6 (Human) | AKDPLPFRDYYYYYMDV | |
| IGHV5-51 (Human) | IGHJ4 (Human) | ARGGPPGGVKLELTDF | |
| IGHV5-51 (Human) | IGHJ4 (Human) | ARGGPPGGVKLELTDY | |
| IGHV3-30 (Human) | IGHJ4 (Human) | AKPVDAAMFDF | |
| IGHV3-53 (Human) | IGHJ6 (Human) | ARPVVGGRAGMDV | |
| IGHV3-13 (Human) | IGHJ2 (Human) | VRAGYSSGWPLYWYFDL | |
| IGHV4-59 (Human) | IGHJ4 (Human) | ARCAWLRGSFDY | |
| IGHV3-53 (Human) | IGHJ6 (Human) | ARHPYGTDV | |
| IGHV1-69 (Human) | IGHJ4 (Human) | ASRWEQLNGGSWHYFDY | |
| IGHV3-73 (Human) | IGHJ5 (Human) | TKDIAAGIPALNWFDS | |
| IGHV4-30 (Human) | IGHJ1 (Human) | ARDAIGSASYGVEYFQH | |
| IGHV1-24 (Human) | IGHJ4 (Human) | ATGGLFMIRGLEI | |
| IGHV1-2 (Human) | IGHJ6 (Human) | ARYKGTTVNTNYYYGMDV | |
| IGHV1-2 (Human) | IGHJ6 (Human) | ARYKGTTVNTNYYYGMDV | |
| IGHV1-8 (Human) | IGHJ6 (Human) | ASRRWDPLTFYYYMVV | |
| IGHV3-30 (Human) | IGHJ5 (Human) | AKSWWLSENWFDP | |
| IGHV3-30 (Human) | IGHJ5 (Human) | AKSWWLSENWFDP | |
| IGHV3-30 (Human) | IGHJ4 (Human) | AKDLGYYYGPPYGPDY | |
| IGHV1-58 (Human) | IGHJ3 (Human) | AAVDCNSTSCYDAFDI | |
| IGHV1-58 (Human) | IGHJ3 (Human) | AAPYCNVTTCFDGFNI | |
| IGHV3-23 (Human) | IGHJ4 (Human) | AKGGDFWSGYLIPFDS | |
| IGHV3-30 (Human) | IGHJ4 (Human) | AKTGASYCGGDCPFHFDY | |
| IGHV3-15 (Human) | IGHJ4 (Human) | TTDHGREPPVH | |
| IGHV3-21 (Human) | IGHJ4 (Human) | ARVPSWAPYQLLPGPFDY | |
| IGHV3-7 (Human) | IGHJ6 (Human) | AREMAGSGNYYWFGYGMDV | |
| IGHV1-8 (Human) | IGHJ4 (Human) | ARGADMLNVAVGADFDY | |
| IGHV4-39 (Human) | IGHJ5 (Human) | ARRLRQLWFGPWFDP | |
| IGHV3-66 (Human) | IGHJ6 (Human) | ARDLMEVGGMDV | |
| IGHV5-51 (Human) | IGHJ3 (Human) | ARSFRDDPRIAVAGPADAFDI | |
| IGHV3-53 (Human) | IGHJ4 (Human) | ARGDVSGYRYGLDY | |
| IGHV3-53 (Human) | IGHJ4 (Human) | ARGDVSGYRYGLDY | |
| IGHV3-72 (Human) | IGHJ4 (Human) | ARGISPFYFDY | |
| IGHV3-72 (Human) | IGHJ4 (Human) | ARGISPFYFDY | |
| IGHV5-51 (Human) | IGHJ6 (Human) | AGGSGISTPMDV | |
| IGHV5-51 (Human) | IGHJ6 (Human) | AGGDGVSTPMDV | |
| IGHV3-53 (Human) | IGHJ5 (Human) | ARIYGDYA | |
| IGHV3-53 (Human) | IGHJ4 (Human) | ARDLGTGLFDY | |
| IGHV3-53 (Human) | IGHJ4 (Human) | ARDYGDYYFDY | |
| IGHV3-30 (Human) | IGHJ4 (Human) | AKEGRPSDIVVVVAFDY | |
| IGHV4-59 (Human) | IGHJ6 (Human) | ARAAGVRSVLAAASSYYYYYGLDV | |
| IGHV3-49 (Human) | IGHJ4 (Human) | ARGLRPTRKADY | |
| IGHV1-69 (Human) | IGHJ5 (Human) | ARGGEEGIAVAAKGWTEQNYENWFDP | |
| IGHV1-46 (Human) | IGHJ3 (Human) | ASGYCSSGSCHAGDAFDI | |
| IGHV4-39 (Human) | IGHJ4 (Human) | AQSTWLRGSFDY | |
| IGHV4-4 (Human) | IGHJ4 (Human) | ARNCAWGSCD | |
| IGHV1-18 (Human) | IGHJ4 (Human) | ARGIAALFGFDY | |
| IGHV1-46 (Human) | IGHJ4 (Human) | AREDVGGTGYFDY | |
| IGHV1-69 (Human) | IGHJ1 (Human) | AREGSAHYDSSGLNAEYFQH | |
| IGHV1-8 (Human) | IGHJ4 (Human) | ARGVGLTYYFDY | |
| IGHV3-53 (Human) | IGHJ6 (Human) | ARDLEAAGGMDV | |
| IGHV3-53 (Human) | IGHJ6 (Human) | ARDLDYYGMDV | |
| IGHV3-53 (Human) | IGHJ4 (Human) | ARDWGEYYFDY | |
| IGHV3-53 (Human) | IGHJ6 (Human) | ARDHVAAMVQGQGV | |
| IGHV3-53 (Human) | IGHJ6 (Human) | ARDLGVSGMDV | |
| IGHV3-66 (Human) | IGHJ4 (Human) | ARDRGGALHDY | |
| IGHV3-23 (Human) | IGHJ5 (Human) | AKDLNALQHVETFDP | |
| IGHV3-30 (Human) | IGHJ4 (Human) | AKGSYYDILTGYYGLDY | |
| IGHV3-30 (Human) | IGHJ4 (Human) | AKDSHYYYYDSSGYYGSIPDY | |
| IGHV3-48 (Human) | IGHJ4 (Human) | ARVGARITGTSTYYFDY | |
| IGHV3-13 (Human) | IGHJ4 (Human) | ARARGGTAMAPYYFDY | |
| IGHV1-69 (Human) | IGHJ6 (Human) | AIERYCTNGVCSAPGPSYYYYAMDV | |
| IGHV1-69 (Human) | IGHJ5 (Human) | AREFAVVPVASTWWFDP | |
| IGHV1-8 (Human) | IGHJ6 (Human) | ARATRSGSYSFVMDV | |
| IGHV3-11 (Human) | IGHJ3 (Human) | ARVFLRSAYPSSRVVWAFDI | |
| IGHV3-11 (Human) | IGHJ5 (Human) | ARGGYCTSTSCYKGFPNWFDP | |
| IGHV3-13 (Human) | IGHJ2 (Human) | ARGGRWLQTVFDL | |
| IGHV3-23 (Human) | IGHJ1 (Human) | AKDVMVWSGSYLPEYFQH | |
| IGHV3-23 (Human) | IGHJ4 (Human) | AKDRYAGPYSSSSDY | |
| IGHV3-30 (Human) | IGHJ4 (Human) | AKVNSQYSSDWYDRAFDY | |
| IGHV3-33 (Human) | IGHJ3 (Human) | ARSQYYYDRSGYANHDAFDI | |
| IGHV3-48 (Human) | IGHJ6 (Human) | ARVKGRSSYYYYGMDV | |
| IGHV3-53 (Human) | IGHJ4 (Human) | ARVVPGNED | |
| IGHV3-53 (Human) | IGHJ6 (Human) | ARGGGHYYGMDV | |
| IGHV3-64 (Human) | IGHJ5 (Human) | ARDGCSSTSCPEINWFDP | |
| IGHV4-39 (Human) | IGHJ3 (Human) | ARRGRVYGGNSGNAFDI | |
| IGHV4-59 (Human) | IGHJ4 (Human) | ARAHYYVTSDFDY | |
| IGHV1-46 (Human) | IGHJ4 (Human) | AREGSTGTTFLDY | |
| IGHV1-69 (Human) | IGHJ6 (Human) | ARDDYGTLWAHYYYGMVV | |
| IGHV3-23 (Human) | IGHJ4 (Human) | AKVTFRTELTQVVPNYFDF | |
| IGHV3-30 (Human) | IGHJ4 (Human) | ATDEGEATTYPGY | |
| IGHV3-30 (Human) | IGHJ6 (Human) | AKSHSTAYSPSTFYYYGLDV | |
| IGHV3-49 (Human) | IGHJ4 (Human) | TRRIMYYSDNSGSRTFDY | |
| IGHV3-30 (Human) | IGHJ4 (Human) | AKNAGPYCSAHNCYSGPFDY | |
| IGHV1-69 (Human) | IGHJ4 (Human) | ARVGDSGGYYPAGLDYFDY | |
| IGHV1-69 (Human) | IGHJ6 (Human) | ARDTVVSPLYGLDV | |
| IGHV3-30 (Human) | IGHJ4 (Human) | AKKSGLYCGGSSCHSGTFDY | |
| IGHV3-30 (Human) | IGHJ4 (Human) | AKCFYSYGYPCYYFDY | |
| IGHV3-33 (Human) | IGHJ3 (Human) | ARELYYYDRSGYYGPDDYAFDI | |
| IGHV3-53 (Human) | IGHJ4 (Human) | ARPRYGSRSYYAGDY | |
| IGHV3-53 (Human) | IGHJ4 (Human) | ARDLGAAGALDF | |
| IGHV3-64D (Human) | IGHJ4 (Human) | QQYGSSPDT | |
| IGHV3-7 (Human) | IGHJ4 (Human) | ARGRLGVVLGVDY | |
| IGHV5-51 (Human) | IGHJ4 (Human) | ARHDSSYVPPGL | |
| IGHV5-51 (Human) | IGHJ6 (Human) | ARIYSSSDNGMDV | |
| IGHV3-53 (Human) | IGHJ4 (Human) | ARSYGDYYLDF | |
| IGHV3-53 (Human) | IGHJ4 (Human) | ARDWGDNYFDY | |
| IGHV3-30 (Human) | IGHJ4 (Human) | AKQISRYCGGGSCYSLTIDY | |
| IGHV3-53 (Human) | IGHJ6 (Human) | AREVYGMDV | |
| IGHV3-53 (Human) | IGHJ6 (Human) | ARSLEAYGMDV | |
| IGHV1-18 (Human) | IGHJ4 (Human) | ARVGTYYSDSSAYYFDY | |
| IGHV3-53 (Human) | IGHJ3 (Human) | ARFIPPDSRGYSSGAFDI | |
| IGHV5-51 (Human) | IGHJ4 (Human) | ARTLSYYDSSGTLLYEDYFDY | |
| IGHV1-46 (Human) | IGHJ4 (Human) | ARGGSPIPAPGSDFDY | |
| IGHV1-69 (Human) | IGHJ4 (Human) | ARVRGTAAAGPNEHIAAAGTFDY | |
| IGHV1-69 (Human) | IGHJ5 (Human) | ARGGGSSGYNWFDP | |
| IGHV1-8 (Human) | IGHJ5 (Human) | ARGGRYCSGDSCYSNIWFDP | |
| IGHV3-13 (Human) | IGHJ6 (Human) | ARERRYYDSSGYHRRDGMDV | |
| IGHV3-30 (Human) | IGHJ4 (Human) | AKQAELYCSAGSCYEGYIDY | |
| IGHV3-33 (Human) | IGHJ4 (Human) | ARDYGDYVTYPDY | |
| IGHV3-53 (Human) | IGHJ4 (Human) | ARGPYPAADNY | |
| IGHV4-30-4 (Human) | IGHJ5 (Human) | ARDGCSGGSCYSYWFDP | |
| IGHV1-69 (Human) | IGHJ4 (Human) | ARFGDYHDNRGYSYSDY | |
| IGHV1-69 (Human) | IGHJ6 (Human) | ARDHEMATIYGMDV | |
| IGHV3-30 (Human) | IGHJ4 (Human) | AKGGYYYYGSGSYNYGFDY | |
| IGHV3-30 (Human) | IGHJ4 (Human) | AKERSWYYYDSSGSDY | |
| IGHV3-48 (Human) | IGHJ6 (Human) | ARHQGSLSYYYHGMDV | |
| IGHV3-53 (Human) | IGHJ4 (Human) | ARDYGDYYFDY | |
| IGHV3-53 (Human) | IGHJ5 (Human) | ARWARGFDP | |
| IGHV3-64D (Human) | IGHJ4 (Human) | VKDLGLGTVDYFDY | |
| IGHV3-7 (Human) | IGHJ4 (Human) | ARARLGQLLGFDY | |
| IGHV5-51 (Human) | IGHJ4 (Human) | ARRGSAWELDY | |
| IGHV5-51 (Human) | IGHJ6 (Human) | GRQVADAASGMDV | |
| IGHV3-9 (Human) | IGHJ6 (Human) | AKDMSGPDYGGIEEYGMDV | |
| IGHV4-39 (Human) | IGHJ4 (Human) | ARLLGLIDY | |
| IGHV4-31 (Human) | IGHJ4 (Human) | ARALGLIDY | |
| IGHV4-31 (Human) | IGHJ4 (Human) | ARAFTPYNSSPFDY | |
| IGHV4-38-2 (Human) | IGHJ3 (Human) | VRALGAFDI | |
| IGHV4-59 (Human) | IGHJ3 (Human) | ARDRGYDSSGPDAFDI | |
| IGHV1-18 (Human) | IGHJ4 (Human) | ARDLGWFGELSVGRDFDY | |
| IGHV1-18 (Human) | IGHJ4 (Human) | ARHLIAVAGTLILSPPGWGYDY | |
| IGHV1-46 (Human) | IGHJ4 (Human) | ARGGHIPYVRGAFDY | |
| IGHV1-69 (Human) | IGHJ5 (Human) | ARDRVESSGWGYWFDP | |
| IGHV3-23 (Human) | IGHJ5 (Human) | AKDASDYNDYVEWFDP | |
| IGHV3-53 (Human) | IGHJ4 (Human) | ARVAYHMGVTHFDS | |
| IGHV3-53 (Human) | IGHJ3 (Human) | ASLYTSGWNFRGAPQV | |
| IGHV3-23 (Human) | IGHJ6 (Human) | AKDGMEWVLVDYYDYYGMDV | |
| IGHV1-69 (Human) | IGHJ4 (Human) | ARGRGYSGYGASYYFDY | |
| IGHV4-39 (Human) | IGHJ4 (Human) | ARLLSPNHYYDSGSYYL | |
| IGHV1-46 (Human) | IGHJ4 (Human) | ARGGVVPAASGEFDY | |
| IGHV3-30 (Human) | IGHJ4 (Human) | AKNIYSYGSTSYFDY | |
| IGHV4-39 (Human) | IGHJ4 (Human) | ARLMWLRGPFDY | |
| IGHV3-13 (Human) | IGHJ2 (Human) | ARVEYSSGIYWYFDL | |
| IGHV3-9 (Human) | IGHJ6 (Human) | AKDLRFLDNGGDYYMDV | |
| IGHV3-30 (Human) | IGHJ4 (Human) | AKTLYSYAYDQYYFDY | |
| IGHV4-4 (Human) | IGHJ4 (Human) | ARAFQDGTSGPYYFDS | |
| IGHV4-59 (Human) | IGHJ4 (Human) | ARETSYYDSSGSFSGSHPYYFDS | |
| IGHV4-39 (Human) | IGHJ4 (Human) | ARLRWLRGDFEY | |
| IGHV3-53 (Human) | IGHJ6 (Human) | ARDLDYYGMDV | |
| IGHV1-18 (Human) | IGHJ6 (Human) | ARDLRTYIGESYYYYGMDV | |
| IGHV1-18 (Human) | IGHJ6 (Human) | ARVKSNSGPQLRFLEWLLFDYYMDV | |
| IGHV1-3 (Human) | IGHJ3 (Human) | ARDTSVLYDFWSAYFQNAFDI | |
| IGHV1-46 (Human) | IGHJ6 (Human) | ARVGSVTYYDILTGHPPYYYYGMDV | |
| IGHV1-69 (Human) | IGHJ6 (Human) | AKDVTTSPLYGMDV | |
| IGHV1-58 (Human) | IGHJ3 (Human) | AAPHCNRTSCFDGFDI | |
| IGHV3-7 (Human) | IGHJ6 (Human) | ARDLGLLWFGEDPRARDV | |
| IGHV4-34 (Human) | IGHJ5 (Human) | ARATYYYDSSGYGINWFDP | |
| IGHV1-69 (Human) | IGHJ6 (Human) | ATGGVHIVVVPTYHNYYGLDV | |
| IGHV1-69 (Human) | IGHJ4 (Human) | ARDRWLADVVVPAAIYPLGY | |
| IGHV1-69 (Human) | IGHJ6 (Human) | ARDRGYSDYGSHYYMDV | |
| IGHV1-18 (Human) | IGHJ4 (Human) | ARSTGILDC | |
| IGHV3-53 (Human) | IGHJ6 (Human) | ARDLYYYGMDV | |
| IGHV2-5 (Human) | IGHJ4 (Human) | AHRPIAIAAAAFDY | |
| IGHV3-15 (Human) | IGHJ6 (Human) | TTATYVLRFLYYYYMDV | |
| IGHV3-30 (Human) | IGHJ4 (Human) | AKDDNRENYDVLTGYYGPFDY | |
| IGHV3-7 (Human) | IGHJ4 (Human) | ASYRYGDYVFDY | |
| IGHV4-61 (Human) | IGHJ3 (Human) | ARALLVTHDAFDI | |
| IGHV3-30 (Human) | IGHJ4 (Human) | ARGRYTYDYAEGFGF | |
| IGHV1-2 (Human) | IGHJ6 (Human) | AVSGVAARLYYGMDV | |
| IGHV2-26 (Human) | IGHJ5 (Human) | ARIDDEMGATAGWFDP | |
| IGHV3-30 (Human) | IGHJ6 (Human) | AKRGEFFQLMDYYGMDV | |
| IGHV3-30 (Human) | IGHJ6 (Human) | AKSLDIFWFRSSYAMDV | |
| IGHV3-53 (Human) | IGHJ6 (Human) | ARDSHGLVLPYYYGMDV | |
| IGHV3-23 (Human) | IGHJ4 (Human) | AKDPVGCINGVCYGAWYYFDS | |
| IGHV1-24 (Human) | IGHJ6 (Human) | ATGVAVAGTPINYYYYYGMDV | |
| IGHV3-53 (Human) | IGHJ5 (Human) | VRCSNYGSWFDP | |
| IGHV3-30 (Human) | IGHJ4 (Human) | AKASALFMITFGGVMGVDY | |
| IGHV3-30 (Human) | IGHJ4 (Human) | AKVKPILGFAQGWFLDY | |
| IGHV3-23 (Human) | IGHJ5 (Human) | AKPNPTYDYVWGSYRYGWFDP | |
| IGHV3-21 (Human) | IGHJ4 (Human) | VRNM | |
| IGHV3-66 (Human) | IGHJ6 (Human) | ARSPEPGFGDLLQNRPYFYYAMDV | |
| IGHV4-39 (Human) | IGHJ3 (Human) | ARDARGILTGFYWSAFDI | |
| IGHV4-4 (Human) | IGHJ5 (Human) | ARPMPGKGFDH | |
| IGHV4-4 (Human) | IGHJ6 (Human) | ATRMVYLDGMDV | |
| IGHV4-28 (Human) | IGHJ5 (Human) | ARGEVLTSGGYNARFDP | |
| IGHV3-30 (Human) | IGHJ4 (Human) | AKDALASLRGATSGQLDY | |
| IGHV4-31 (Human) | IGHJ4 (Human) | ARGPKVLRLLEWLTPSFED | |
| IGHV1-24 (Human) | IGHJ3 (Human) | ATEGLVMVITYAFET | |
| IGHV1-69 (Human) | IGHJ3 (Human) | ARGRDIVVEVSDSDPFDI | |
| IGHV3-64 (Human) | IGHJ5 (Human) | VKDSGWFGELAEGLIYWFDP | |
| IGHV4-39 (Human) | IGHJ4 (Human) | ARDRTPAYEILTGHHLLPKYYFDF | |
| IGHV3-53 (Human) | IGHJ6 (Human) | ARDLHIYGMDV | |
| IGHV3-21 (Human) | IGHJ2 (Human) | ARDFFGGDSGDYWYFDL | |
| IGHV3-21 (Human) | IGHJ3 (Human) | ARDGGVDIVDTSTDAFDI | |
| IGHV1-69 (Human) | IGHJ6 (Human) | ARGNDDSSGYYSHYYYGMDV | |
| IGHV4-34 (Human) | IGHJ1 (Human) | CARGVPGDW | |
| IGHV4-39 (Human) | IGHJ4 (Human) | GRHPPYSNFDY | |
| IGHV3-30 (Human) | IGHJ4 (Human) | AKDYGDYGGLDY | |
| IGHV2-70 (Human) | IGHJ3 (Human) | ARTRATALMAFDI | |
| IGHV1-2 (Human) | IGHJ6 (Human) | ARARGSSGWYRIGTRWGNWFDP | |
| IGHV1-58 (Human) | IGHJ3 (Human) | AAPYCSSTNCYDAFDI | |
| IGHV3-30 (Human) | IGHJ6 (Human) | ARGWAYWELLPDYYYGMDV | |
| IGHV3-30 (Human) | IGHJ6 (Human) | ARDLAIAVAGTWHYYNGMDV | |
| IGHV1-58 (Human) | IGHJ3 (Human) | AAPYCSGGSCFDGFDI | |
| IGHV3-23 (Human) | IGHJ4 (Human) | AKGYYYDSSGYYFREDAFDI | |
| IGHV5-51 (Human) | IGHJ6 (Human) | AGGSGISTPMDV | |
| IGHV1-69 (Human) | IGHJ6 (Human) | ARDTATGGMDV | |
| IGHV3-53 (Human) | IGHJ4 (Human) | ARDLGTGLFDY | |
| IGHV3-23 (Human) | IGHJ4 (Human) | ARSYYYGGFGMDY | |
| IGHV3-23 (Human) | IGHJ4 (Human) | ARSYYYGGFGMDY | |
| IGHV3-23 (Human) | IGHJ4 (Human) | AKDADSFDY | |
| IGHV3-23 (Human) | IGHJ3 (Human) | AKDDYGDYVLGAFDI | |
| IGHV3-23 (Human) | IGHJ4 (Human) | VKDFVGADGPFVFDY | |
| IGHV6-1 (Human) | IGHJ6 (Human) | AREQQQLVPHYYYYGMDV | |
| IGHV3-64 (Human) | IGHJ6 (Human) | AKTYYDFWRTYYGMDV | |
| IGHV3-23 (Human) | IGHJ6 (Human) | ARDRYYTMDV | |
| IGHV4-34 (Human) | IGHJ4 (Human) | VRLPMIKKSFDI | |
| IGHV3-23 (Human) | IGHJ6 (Human) | AKDVSYHADV | |
| IGHV3-66 (Human) | IGHJ4 (Human) | ARGGITGTTPIDY | |
| IGHV4-59 (Human) | IGHJ6 (Human) | ARSAKHWLAPPGDYYYYMDV | |
| IGHV1-46 (Human) | IGHJ4 (Human) | ARPGGGSYQEFDY | |
| IGHV1-24 (Human) | IGHJ6 (Human) | ATGWAVAGSSDVWYYYYGMDV | |
| IGHV1-69 (Human) | IGHJ5 (Human) | ARGVGYRGVIPLNWFDP | |
| IGHV1-24 (Human) | IGHJ4 (Human) | ATGWAYKSTWYFGY | |
| IGHV1-69 (Human) | IGHJ6 (Human) | ASLQTVDTAIEKYYGMDV | |
| IGHV4-59 (Human) | IGHJ4 (Human) | ARGFDY | |
| IGHV4-59 (Human) | IGHJ4 (Human) | ARGFDF | |
| IGHV2-70 (Human) | IGHJ4 (Human) | ARMIVTTSTYFDY | |
| IGHV2-70 (Human) | IGHJ4 (Human) | ARMIVTTSTYFDY | |
| IGHV2-5 (Human) | IGHJ4 (Human) | ARLTAADTIFDC | |
| IGHV1-24 (Human) | IGHJ5 (Human) | ATTTVHCSGGSCSSYWFDP | |
| IGHV3-11 (Human) | IGHJ4 (Human) | VRGALLLWFGELSAEPFDY | |
| IGHV1-69 (Human) | IGHJ4 (Human) | ARDAPDYDFWSGRPRYFDS | |
| IGHV1-8 (Human) | IGHJ5 (Human) | ARGGRYCSGGSCFSGIWFDP | |
| IGHV4-59 (Human) | IGHJ4 (Human) | ASDYSDSTGYYYGFDH | |
| IGHV3-13 (Human) | IGHJ6 (Human) | ARVKFRFLEWFLDV | |
| IGHV4-34 (Human) | IGHJ2 (Human) | ARGVGWFYYWYFDL | |
| IGHV2-70 (Human) | IGHJ4 (Human) | ARMRVGGYDSYFDY | |
| IGHV5-51 (Human) | IGHJ6 (Human) | TRHQYGYNYGYFYYYIDV | |
| IGHV4-4 (Human) | IGHJ4 (Human) | ARRTGYCGSTSCYNFDH | |
| IGHV2-5 (Human) | IGHJ4 (Human) | AHYSSSSLYFAY | |
| IGHV3-66 (Human) | IGHJ4 (Human) | ARGEGWDLPFDF | |
| IGHV3-11 (Human) | IGHJ4 (Human) | ARDVSSDWRRGYFDY | |
| IGHV4-59 (Human) | IGHJ6 (Human) | ARALDPHSRSYYMDV | |
| IGHV3-30 (Human) | IGHJ3 (Human) | ARVYGGNYFNGFDI | |
| IGHV1-69 (Human) | IGHJ6 (Human) | ARDGSNSRENRVSYMDV | |
| IGHV3-43 (Human) | IGHJ4 (Human) | GRDLYRRISGYFGTTFDY | |
| IGHV1-69 (Human) | IGHJ1 (Human) | ATHYYDSSVYYDEFQADYFHY | |
| IGHV4-39 (Human) | IGHJ2 (Human) | ARPVGEVYNSGLDVDYWYFDL | |
| IGHV3-33 (Human) | IGHJ3 (Human) | ARGRIGNSMIVDVTGEDAFDI | |
| IGHV4-39 (Human) | IGHJ4 (Human) | ARLCRFVVIATASFDY | |
| IGHV3-15 (Human) | IGHJ4 (Human) | TTESEGYSYGYEDFDY | |
| IGHV3-21 (Human) | IGHJ3 (Human) | VRARMNYYDSKGYYPDAFDI | |
| IGHV1-24 (Human) | IGHJ4 (Human) | ATDYAAVQLGLAF | |
| IGHV1-24 (Human) | IGHJ5 (Human) | ATSSPFTSYNWFDP | |
| IGHV2-5 (Human) | IGHJ4 (Human) | AHRCVDTAVLAFDY | |
| IGHV1-69 (Human) | IGHJ6 (Human) | ARGLGSLDYYYMDV | |
| IGHV3-33 (Human) | IGHJ4 (Human) | ARDGGFTGYPYFEN | |
| IGHV4-39 (Human) | IGHJ4 (Human) | ARSGIVAVPAAIWPVDY | |
| IGHV4-39 (Human) | IGHJ5 (Human) | ARPIAVYQVLKQHNNWFDP | |
| IGHV1-69 (Human) | IGHJ5 (Human) | ARERVYSGSGAAYWFDP | |
| IGHV3-30 (Human) | IGHJ4 (Human) | ARDHHRRPDND | |
| IGHV1-18 (Human) | IGHJ4 (Human) | ARDLELGGAFDY | |
| IGHV4-39 (Human) | IGHJ4 (Human) | ARMSGSGNYNLADYHFDS | |
| IGHV3-7 (Human) | IGHJ6 (Human) | ARIKIVVEPPGGGVFQGYYYNYYMDV | |
| IGHV1-46 (Human) | IGHJ5 (Human) | ARSRVAPSEDWFDP | |
| IGHV1-69 (Human) | IGHJ4 (Human) | ARGGGTLTGTTAFDY | |
| IGHV3-20 (Human) | IGHJ6 (Human) | ARVGSTIFGVVRRNYYYYMDV | |
| IGHV3-30 (Human) | IGHJ3 (Human) | ARVYGGNYFNGFDI | |
| IGHV3-30 (Human) | IGHJ4 (Human) | ARPHTGNYYDYFDF | |
| IGHV4-59 (Human) | IGHJ6 (Human) | ALGPLGYYYYYMDV | |
| IGHV4-59 (Human) | IGHJ4 (Human) | ARLPLFSAFDY | |
| IGHV3-53 (Human) | IGHJ4 (Human) | AREVAGTYDY | |
| IGHV3-53 (Human) | IGHJ5 (Human) | ARSPYGGNS | |
| IGHV5-51 (Human) | IGHJ6 (Human) | ARQGSHYGMDV | |
| IGHV3-30 (Human) | IGHJ3 (Human) | AGGGVLVTSDPDAFDI | |
| IGHV5-51 (Human) | IGHJ1 (Human) | ARIEYYNDSSGYYQF | |
| IGHV1-8 (Human) | IGHJ5 (Human) | ARGLWFGDLTRTKYNWFDP | |
| IGHV5-51 (Human) | IGHJ4 (Human) | ARQESGWSFDY | |
| IGHV2-5 (Human) | IGHJ5 (Human) | AHTTWYYYGSGWFDP | |
| IGHV3-23 (Human) | IGHJ4 (Human) | AKEPSFGLWFGELSG | |
| IGHV3-13 (Human) | IGHJ3 (Human) | ARGSLRGGILSGYAFDI | |
| IGHV5-51 (Human) | IGHJ4 (Human) | ARHPVLRGNIDY | |
| IGHV3-13 (Human) | IGHJ2 (Human) | ARVGYDSSGYYWYLDL | |
| IGHV3-33 (Human) | IGHJ4 (Human) | AKDGSGSYYNSGALDY | |
| IGHV3-66 (Human) | IGHJ4 (Human) | ARDYGDYYFDY | |
| IGHV1-18 (Human) | IGHJ4 (Human) | ATDDPDIVLVPAAMSLDY | |
| IGHV1-18 (Human) | IGHJ4 (Human) | ATDDPDIVLVPAAMSLDY | |
| IGHV4-59 (Human) | IGHJ6 (Human) | ATDYYDSSGYRYGMDV | |
| IGHV1-69 (Human) | IGHJ4 (Human) | ASFGSLWDLRDY | |
| IGHV3-23 (Human) | IGHJ4 (Human) | AKDVGSRLIYDVFDY | |
| IGHV2-70 (Human) | IGHJ4 (Human) | TRTATVVKDY | |
| IGHV2-70 (Human) | IGHJ4 (Human) | ARMIPIPALDY | |
| IGHV2-70 (Human) | IGHJ4 (Human) | AREEAAGTKLDY | |
| IGHV3-30-3 (Human) | IGHJ4 (Human) | AKRGGTYCSGGICYGGYFDY | |
| IGHV1-69 (Human) | IGHJ3 (Human) | ARGPAYVSGTYYWNAFDI | |
| IGHV3-30 (Human) | IGHJ3 (Human) | ARDPPPGNMGSMAQHLVLLVVFDI | |
| IGHV3-66 (Human) | IGHJ6 (Human) | AREGLGMDV | |
| IGHV3-33 (Human) | IGHJ4 (Human) | AKGGFGYASGWYYLDY | |
| IGHV3-13 (Human) | IGHJ4 (Human) | ARAYYDTSGYYNYFDH | |
| IGHV1-46 (Human) | IGHJ4 (Human) | ARDQAFIVATLGPDY | |
| IGHV3-53 (Human) | IGHJ5 (Human) | AKMLWLRGWFDP | |
| IGHV2-70 (Human) | IGHJ5 (Human) | ARFLVGGFKAWFDP | |
| IGHV5-51 (Human) | IGHJ4 (Human) | ARQESGWSFDY | |
| IGHV1-58 (Human) | IGHJ3 (Human) | AAPYCSSTSCRDGFDI | |
| IGHV1-46 (Human) | IGHJ6 (Human) | ARDGSPWGDDYDIYGMDV | |
| IGHV3-53 (Human) | IGHJ3 (Human) | ARDGGYSAYTLFYSDFAFDI | |
| IGHV3-9 (Human) | IGHJ5 (Human) | AKDRSGDYGDYEAGFDP | |
| IGHV1-18 (Human) | IGHJ5 (Human) | ARVQEFWLDP | |
| IGHV3-66 (Human) | IGHJ4 (Human) | ARDGLVGATLAFDF | |
| IGHV1-2 (Human) | IGHJ5 (Human) | ARGTPPDSVVVPAAIHPYDWFDP | |
| IGHV4-39 (Human) | IGHJ4 (Human) | ATLEWLRAPFDD | |
| IGHV2-5 (Human) | IGHJ4 (Human) | AHRPAGFWSAHFDY | |
| IGHV2-70 (Human) | IGHJ4 (Human) | ALGRAGTMDY | |
| IGHV3-9 (Human) | IGHJ6 (Human) | AKDMVAGPHYYGMDV | |
| IGHV5-51 (Human) | IGHJ6 (Human) | ARHPSNFYDSGGDYYAMDV | |
| IGHV3-9 (Human) | IGHJ4 (Human) | AKDLSSGWDLFDY | |
| IGHV5-51 (Human) | IGHJ4 (Human) | VRSDGDYVIGHDY | |
| IGHV3-48 (Human) | IGHJ4 (Human) | ARGRDDYGDYRGGDFDY | |
| IGHV3-53 (Human) | IGHJ4 (Human) | ARFRYGDYPDY | |
| IGHV1-18 (Human) | IGHJ4 (Human) | ARDYGWFGELSTEGQFDY | |
| IGHV2-70 (Human) | IGHJ4 (Human) | ARVQVAAAGSPYDY | |
| IGHV1-58 (Human) | IGHJ6 (Human) | AADRMRIVGGKGYYYGMDV | |
| IGHV1-18 (Human) | IGHJ4 (Human) | ARVQWLRLDY | |
| IGHV1-18 (Human) | IGHJ4 (Human) | ARDYGWFGELSTEGQFDY | |
| IGHV4-4 (Human) | IGHJ3 (Human) | ARDQGYSSGWNDAFDI | |
| IGHV3-7 (Human) | IGHJ4 (Human) | ARSPHYYGGFDY | |
| IGHV4-31 (Human) | IGHJ4 (Human) | ARVSRYTMVRGVIFDY | |
| IGHV4-38-2 (Human) | IGHJ4 (Human) | ARVGVATILGVDY | |
| IGHV3-30 (Human) | IGHJ6 (Human) | AKGGGYISAWSTRYYAMDV | |
| IGHV3-23 (Human) | IGHJ4 (Human) | AESSSLTGNFNY | |
| IGHV1-46 (Human) | IGHJ6 (Human) | ARDPPSGNYDNIDEWTRSENHYNYGMDA | |
| IGHV3-11 (Human) | IGHJ4 (Human) | ARDGSAVAGPMSYFDY | |
| IGHV2-70 (Human) | IGHJ4 (Human) | AREVAGAVHLDY | |
| IGHV3-7 (Human) | IGHJ4 (Human) | ARDLGVLWFGDLLF | |
| IGHV4-59 (Human) | IGHJ4 (Human) | ARGGYYYGPPRDFDY | |
| IGHV2-5 (Human) | IGHJ4 (Human) | AHSPDHRYFDVLTGYFNSERFYFDY | |
| IGHV3-30 (Human) | IGHJ4 (Human) | AKAAGGGYSYIYWGGDY | |
| IGHV3-13 (Human) | IGHJ4 (Human) | ARGFDTTTGFYFDY | |
| IGHV1-69 (Human) | IGHJ6 (Human) | ARTEYSYDSGSSRAYSMDV | |
| IGHV3-33 (Human) | IGHJ4 (Human) | AKGGWYSSKWYYFDY | |
| IGHV7-4-1 (Human) | IGHJ5 (Human) | ARVGPSSSWPS | |
| IGHV3-7 (Human) | IGHJ4 (Human) | ARDLGVLWFGDPY | |
| IGHV3-30 (Human) | IGHJ4 (Human) | ARDWVHYGSGSYPPDY | |
| IGHV4-39 (Human) | IGHJ3 (Human) | ARYYGPGTYYDAFDI | |
| IGHV3-66 (Human) | IGHJ4 (Human) | ARELGPVGGTDQ | |
| IGHV3-30 (Human) | IGHJ4 (Human) | AKGGWYGDIRVDY | |
| IGHV2-5 (Human) | IGHJ4 (Human) | AHSLPSKYSYSYGSFDY | |
| IGHV3-23 (Human) | IGHJ5 (Human) | AKGELLWFGELLENWFDP | |
| IGHV5-51 (Human) | IGHJ3 (Human) | ARLPPHFGSGSYYGNAFDI | |
| IGHV1-46 (Human) | IGHJ4 (Human) | ARGGLVPAVMPALDY | |
| IGHV3-13 (Human) | IGHJ6 (Human) | VRSDHSSGWYGTYYYYMDV | |
| IGHV3-66 (Human) | IGHJ3 (Human) | ARDLDIAGAFDI | |
| IGHV3-66 (Human) | IGHJ4 (Human) | ARDLVVAGIDY | |
| IGHV3-66 (Human) | IGHJ6 (Human) | ARDLGPLGMDV | |
| IGHV4-4 (Human) | IGHJ4 (Human) | ARVQGLIDY | |
| IGHV3-23 (Human) | IGHJ5 (Human) | AKQTDYGVGWFDP | |
| IGHV1-69 (Human) | IGHJ4 (Human) | AVLPLHSSYNWYYFDY | |
| IGHV3-13 (Human) | IGHJ3 (Human) | ARGSDTVTTAFDI | |
| IGHV3-66 (Human) | IGHJ4 (Human) | AREAPNSRGSGTNFDY | |
| IGHV3-33 (Human) | IGHJ4 (Human) | AKNGYSYAYPRQYFDY | |
| IGHV1-24 (Human) | IGHJ5 (Human) | ATGSPFGVVTDWFDP | |
| IGHV1-46 (Human) | IGHJ4 (Human) | ARDVRVDDSWSGYDLLSGGTYFDY | |
| IGHV1-2 (Human) | IGHJ6 (Human) | ARAPLFPTGVLAGDYYYYGMDV | |
| IGHV3-30 (Human) | IGHJ4 (Human) | ARHATLMNNKDI | |
| IGHV1-69 (Human) | IGHJ6 (Human) | ARGYYEARHYYYYYAMDV | |
| IGHV4-39 (Human) | IGHJ2 (Human) | ARQWKWFGEAWYFDL | |
| IGHV1-69 (Human) | IGHJ4 (Human) | ARSCGDCYSADLDF | |
| IGHV5-51 (Human) | IGHJ4 (Human) | ARQESGWSFDY | |
| IGHV3-23 (Human) | IGHJ4 (Human) | AKGQRGSPDFFDY | |
| IGHV1-3 (Human) | IGHJ4 (Human) | ARAGWELNY | |
| IGHV3-53 (Human) | IGHJ4 (Human) | ARDLVTWGLDY | |
| IGHV3-15 (Human) | IGHJ5 (Human) | STTNDYGDYSANY | |
| IGHV1-8 (Human) | IGHJ5 (Human) | ARGLWFGDLTRTKYNWFDP | |
| IGHV2-70 (Human) | IGHJ4 (Human) | ARITPHLVYDY | |
| IGHV4-59 (Human) | IGHJ5 (Human) | ARTLGAYYDILTGFRTPGGWFAP | |
| IGHV3-11 (Human) | IGHJ2 (Human) | ASPLLSHNYGSGSYYNVYWYFEL | |
| IGHV3-53 (Human) | IGHJ6 (Human) | ARDLENGGLDV | |
| IGHV1-58 (Human) | IGHJ3 (Human) | AAPNCNSTTCHDGFDI | |
| IGHV3-53 (Human) | IGHJ4 (Human) | AREGLVGTALAFDY | |
| IGHV3-30 (Human) | IGHJ5 (Human) | AKDPTSLYCSGGSCYNNWFDP | |
| IGHV3-66 (Human) | IGHJ6 (Human) | ARDLDYYGMDV | |
| IGHV3-30 (Human) | IGHJ3 (Human) | AGGGVLVTSDPDAFDI | |
| IGHV4-4 (Human) | IGHJ4 (Human) | AGEQHIVTTIIDY | |
| IGHV3-66 (Human) | IGHJ4 (Human) | ARDYGDYYFDY | |
| IGHV3-9 (Human) | IGHJ6 (Human) | AKDIGVMVPGVTPYGMDV | |
| IGHV3-66 (Human) | IGHJ6 (Human) | ARDPMRPGMDV | |
| IGHV3-43 (Human) | IGHJ4 (Human) | ARESPKLTGYFDY | |
| IGHV1-69 (Human) | IGHJ4 (Human) | ARGRYTYGTEGYFDN | |
| IGHV3-66 (Human) | IGHJ6 (Human) | ARDAVGSYYYGMEV | |
| IGHV3-53 (Human) | IGHJ4 (Human) | AREGLVGTTLTFDY | |
| IGHV3-66 (Human) | IGHJ3 (Human) | ARYYGPQGRAFDI | |
| IGHV4-34 (Human) | IGHJ5 (Human) | ARGWTVPPLWVLNWFDP | |
| IGHV1-69 (Human) | IGHJ4 (Human) | ATGRYTYGYGYYFDY | |
| IGHV3-66 (Human) | IGHJ3 (Human) | ARDLNIAGGFDI | |
| IGHV1-18 (Human) | IGHJ4 (Human) | ARDLAWFGELSESPIEY | |
| IGHV2-5 (Human) | IGHJ3 (Human) | AHRLAPDYDFLTGYYNGDDAFDV | |
| IGHV3-66 (Human) | IGHJ6 (Human) | AREGLLVGPTGRGLGMDV | |
| IGHV2-70 (Human) | IGHJ4 (Human) | ARMVVRGVMLDY | |
| IGHV5-51 (Human) | IGHJ3 (Human) | ATRTGWTNDAFDI | |
| IGHV1-18 (Human) | IGHJ2 (Human) | ARARQLVLNWYFDL | |
| IGHV3-7 (Human) | IGHJ4 (Human) | ARLMYYYGNFDY | |
| IGHV3-13 (Human) | IGHJ2 (Human) | ARVGYYGSGSYPLYWYFDL | |
| IGHV3-66 (Human) | IGHJ4 (Human) | ARLASDGSGSYLDYFDY | |
| IGHV1-69 (Human) | IGHJ6 (Human) | ATDGGGGSYYYAHYYYGMDV | |
| IGHV2-5 (Human) | IGHJ4 (Human) | AHSMVRGVLFGADFDY | |
| IGHV1-69 (Human) | IGHJ2 (Human) | AREAGTTDWYFDL | |
| IGHV4-39 (Human) | IGHJ4 (Human) | ARHPRFSWRGNDSGYFDY | |
| IGHV3-33 (Human) | IGHJ4 (Human) | ARDGVDFGMVTLFDY | |
| IGHV1-24 (Human) | IGHJ4 (Human) | AITSVARGLRGYFDT | |
| IGHV1-24 (Human) | IGHJ4 (Human) | AITSLARGLKGYFDS | |
| IGHV4-61 (Human) | IGHJ5 (Human) | AMTYYDYIWGRVDPQFDP | |
| IGHV4-39 (Human) | IGHJ4 (Human) | ARFITDGYSSGSDS | |
| IGHV3-30 (Human) | IGHJ4 (Human) | AKQASPYCSGGSCYSGNFDY | |
| IGHV4-31 (Human) | IGHJ4 (Human) | ARDYGGNSNYFHY | |
| IGHV2-70 (Human) | IGHJ4 (Human) | ARIQRGIAADY | |
| IGHV3-66 (Human) | IGHJ4 (Human) | ARDKWEGTFDY | |
| IGHV3-66 (Human) | IGHJ6 (Human) | AETGWDGMDV | |
| IGHV1-46 (Human) | IGHJ4 (Human) | ARDGALYSNSPTEFDY | |
| IGHV3-23 (Human) | IGHJ6 (Human) | ARGLQYYYDTSGYYKDSYYYGVDV | |
| IGHV3-66 (Human) | IGHJ4 (Human) | ARDLPLHGDYFDY | |
| IGHV2-70 (Human) | IGHJ4 (Human) | ARVQVAAAGSPYDY | |
| IGHV3-9 (Human) | IGHJ4 (Human) | AKDRGYEILTPASFDY | |
| IGHV4-31 (Human) | IGHJ6 (Human) | AREKIRSIAAAGTVYYYGMDV | |
| IGHV4-59 (Human) | IGHJ5 (Human) | ASTYWDSSGYYYGVDY | |
| IGHV1-18 (Human) | IGHJ6 (Human) | AVLDYCSGGSSSSGYYNYGMDV | |
| IGHV1-18 (Human) | IGHJ4 (Human) | AREGAGLIIAYDY | |
| IGHV1-69 (Human) | IGHJ6 (Human) | ARYMVTRDQYYYDMDV | |
| IGHV4-39 (Human) | IGHJ4 (Human) | ASQQWLRGNFDY | |
| IGHV3-23 (Human) | IGHJ6 (Human) | AKDLFYDFWSGFDFGPGESFGMDV | |
| IGHV3-48 (Human) | IGHJ4 (Human) | ARMAIVGAYANWGFDY | |
| IGHV1-24 (Human) | IGHJ5 (Human) | ATTTPFGVVNAIWFDP | |
| IGHV1-24 (Human) | IGHJ5 (Human) | ATGSPFGVVTSWFDP | |
| IGHV3-33 (Human) | IGHJ4 (Human) | ARDRVDSSSWGFYFDY | |
| IGHV3-43 (Human) | IGHJ4 (Human) | AKAKDPYTEYFDY | |
| IGHV3-53 (Human) | IGHJ3 (Human) | ARGDIVGATWDPAFDI | |
| IGHV2-70 (Human) | IGHJ4 (Human) | ARINAYSSSWPTFDY | |
| IGHV4-39 (Human) | IGHJ5 (Human) | ARSSSGFSYDTPLDP | |
| IGHV1-46 (Human) | IGHJ3 (Human) | ARDPRTSGDNDGFDM | |
| IGHV1-46 (Human) | IGHJ3 (Human) | ARDPRSSGDNDGFDM | |
| IGHV3-30 (Human) | IGHJ6 (Human) | AKENRDRNYDSWSASYSDYYYGMDV | |
| IGHV3-30 (Human) | IGHJ6 (Human) | AKENRDRNYDSWSGSYSDYYYGMDV | |
| IGHV3-30 (Human) | IGHJ6 (Human) | AKENRDRNYGSWSGSNSDYYYGMDV | |
| IGHV1-69 (Human) | IGHJ4 (Human) | ARDDGKMNFGSAPWDFFHS | |
| IGHV3-33 (Human) | IGHJ5 (Human) | ARDVAHCTTVGCHGNWFDT | |
| IGHV3-30 (Human) | IGHJ4 (Human) | AKKGSPYCGVDCYKGYFDY | |
| IGHV4-34 (Human) | IGHJ3 (Human) | ARDLSRTYRFQYYDSGVTEAFDI | |
| IGHV4-4 (Human) | IGHJ3 (Human) | ARVRIGASYYDFWSGYYSDAFDI | |
| IGHV4-59 (Human) | IGHJ3 (Human) | ARGLVIRYSDSFPSGPIIAAFDI | |
| IGHV3-23 (Human) | IGHJ4 (Human) | AKDAGSDSLWGNLRYTDVGRYFDY | |
| IGHV4-34 (Human) | IGHJ4 (Human) | ARGRRGIVVGIDDPNYDY | |
| IGHV4-4 (Human) | IGHJ3 (Human) | ARAQPNDFWSGYYTAAFDM | |
| IGHV3-30 (Human) | IGHJ4 (Human) | AKAPYSYAYSVYFFDY | |
| IGHV1-46 (Human) | IGHJ6 (Human) | ARDYFLIPAANTMEV | |
| IGHV3-30-3 (Human) | IGHJ4 (Human) | ARDQLLDY | |
| IGHV3-21 (Human) | IGHJ6 (Human) | AAEGISVFGVVTHFYALAA | |
| IGHV3-21 (Human) | IGHJ6 (Human) | AREGITSFGIVTHYYALDV | |
| IGHV3-30-3 (Human) | IGHJ4 (Human) | ARGGGGTYSYFDF | |
| IGHV1-18 (Human) | IGHJ4 (Human) | ARAGPEVPFDH | |
| IGHV3-11 (Human) | IGHJ5 (Human) | ARGHRFLEFPLNYLDP | |
| IGHV3-48 (Human) | IGHJ4 (Human) | ACMVGPTAPFDF | |
| IGHV3-74 (Human) | IGHJ4 (Human) | VREGIVATIYFDY | |
| IGHV3-30-3 (Human) | IGHJ4 (Human) | ARDRAANYGRGGFDY | |
| IGHV3-23 (Human) | IGHJ6 (Human) | AKLEGGHYYGSGSYYISTPLAMDV | |
| IGHV3-23 (Human) | IGHJ3 (Human) | ARDFHVRFLNYYTPVGGAFDI | |
| IGHV1-69-2 (Human) | IGHJ4 (Human) | ATETIFGVSPRVEGN | |
| IGHV3-9 (Human) | IGHJ6 (Human) | ARGGFKSGFYIHGMDV | |
| IGHV4-4 (Human) | IGHJ5 (Human) | ARLFSDSGNYFWFDP | |
| IGHV3-7 (Human) | IGHJ4 (Human) | ARDESTYYYDSSGYYYEGYFDY | |
| IGHV3-30 (Human) | IGHJ4 (Human) | AKEGNNYDFWSGYSYYFDY | |
| IGHV3-9 (Human) | IGHJ4 (Human) | AKGDCSSTSCDDPYFDY | |
| IGHV1-69 (Human) | IGHJ5 (Human) | AREEFVVVPGNWFDP | |
| IGHV3-33 (Human) | IGHJ4 (Human) | TTDIPALTENPFDY | |
| IGHV4-30-4 (Human) | IGHJ4 (Human) | ARDQRTGSYFGYYFDY | |
| IGHV4-59 (Human) | IGHJ3 (Human) | ASYYYDSSGYYYGFDI | |
| IGHV1-18 (Human) | IGHJ5 (Human) | AICWGPYNWNEG | |
| IGHV3-23 (Human) | IGHJ4 (Human) | ARDWPTNVRYCSSDICPLFDY | |
| IGHV3-30-3 (Human) | IGHJ4 (Human) | ARGRGSYNTYFDY | |
| IGHV3-30-3 (Human) | IGHJ4 (Human) | ARGGGNYREPFDY | |
| IGHV3-53 (Human) | IGHJ4 (Human) | VRVGGSSGWVSDYYFDS | |
| IGHV3-30 (Human) | IGHJ4 (Human) | AKDYGDYGAFDY | |
| IGHV3-23 (Human) | IGHJ6 (Human) | ARGPQCTSAGCYVVGAMDV | |
| IGHV3-30-3 (Human) | IGHJ6 (Human) | ARQGAHCSGGVCYGMDV | |
| IGHV3-30-3 (Human) | IGHJ6 (Human) | ARQGAHCSGGVCYGMDV | |
| IGHV4-34 (Human) | IGHJ5 (Human) | ARVPTVTVYGVDVINVSWFDP | |
| IGHV4-31 (Human) | IGHJ5 (Human) | TRAIRYCLGGYCHHPWFDP | |
| IGHV3-30 (Human) | IGHJ3 (Human) | ARAYSGSYFNAFNI | |
| IGHV3-33 (Human) | IGHJ4 (Human) | ARGDGGGYWSGYGEVK | |
| IGHV4-34 (Human) | IGHJ5 (Human) | ARGTTIGLYTNTWNFPRLGFDP | |
| IGHV3-23 (Human) | IGHJ4 (Human) | AKLAVAGGRGANY | |
| IGHV1-8 (Human) | IGHJ5 (Human) | AKGPDDYWSGYGRFDP | |
| IGHV1-69 (Human) | IGHJ4 (Human) | ARERYNTRNFDY | |
| IGHV4-34 (Human) | IGHJ5 (Human) | ARGVTTPVVHLYSSGRPSRWFDS | |
| IGHV1-8 (Human) | IGHJ4 (Human) | ARGPSGYYYGSGDF | |
| IGHV3-64D (Human) | IGHJ3 (Human) | VKDKVTTIFDVFDL | |
| IGHV3-15 (Human) | IGHJ4 (Human) | TTDRIYDYVWGSYRYKDY | |
| IGHV3-15 (Human) | IGHJ4 (Human) | TTDRDYDYIWGSYRYRDY | |
| IGHV1-69 (Human) | IGHJ6 (Human) | GHMAATGRISFYYHGLDV | |
| IGHV5-10-1 (Human) | IGHJ5 (Human) | ARHFPRTQWLPWGFDP | |
| IGHV3-53 (Human) | IGHJ4 (Human) | ARDYGDYYFDY | |
| IGHV3-53 (Human) | IGHJ4 (Human) | ARDLQQLGIDY | |
| IGHV3-30 (Human) | IGHJ4 (Human) | AKQGGGPYYFGSGYFDY | |
| IGHV3-23 (Human) | IGHJ4 (Human) | ARHNELDY | |
| IGHV3-23 (Human) | IGHJ4 (Human) | ARHNELDY | |
| IGHV3-23 (Human) | IGHJ4 (Human) | ARHNELDH | |
| IGHV1-69 (Human) | IGHJ4 (Human) | ARSFCSSGACYFDY | |
| IGHV3-30-3 (Human) | IGHJ3 (Human) | ARARGGSYAGGFDI | |
| IGHV3-30-3 (Human) | IGHJ3 (Human) | AKSRGGNYFDAFDI | |
| IGHV3-30-3 (Human) | IGHJ3 (Human) | ARVFGGSYLGAFDV | |
| IGHV3-30 (Human) | IGHJ4 (Human) | AKTIWGTYRSPFDY | |
| IGHV3-15 (Human) | IGHJ4 (Human) | TTVHSDYLWGSYRKGYFDY | |
| IGHV3-15 (Human) | IGHJ4 (Human) | TTVHSDYVWGSYRKGYFDY | |
| IGHV4-61 (Human) | IGHJ4 (Human) | ARDVDIVGVFDY | |
| IGHV3-48 (Human) | IGHJ3 (Human) | ARDRNRGYNKLGDAFDI | |
| IGHV4-4 (Human) | IGHJ2 (Human) | ARGFTNWYFDL | |
| IGHV3-30-3 (Human) | IGHJ3 (Human) | ARTDGYNQAFDI | |
| IGHV3-30-3 (Human) | IGHJ5 (Human) | ARSGSGNYYHWFDP | |
| IGHV3-30-3 (Human) | IGHJ5 (Human) | ARSGSGNYYHWFDP | |
| IGHV3-30-3 (Human) | IGHJ5 (Human) | ASHDGVTETTWKYYAMDV | |
| IGHV1-46 (Human) | IGHJ5 (Human) | ARERVGVGIYSRSSESDFPTFDP | |
| IGHV1-69 (Human) | IGHJ6 (Human) | ARVVITGLDYYYGMDV | |
| IGHV3-30-3 (Human) | IGHJ6 (Human) | ARANGGNYYYGMDV | |
| IGHV3-30-3 (Human) | IGHJ6 (Human) | ARARGGNYYYGMDV | |
| IGHV3-30-3 (Human) | IGHJ6 (Human) | ASTFGGAYSYGMDV | |
| IGHV3-30-3 (Human) | IGHJ6 (Human) | ARATRGSYYYGMDV | |
| IGHV3-9 (Human) | IGHJ6 (Human) | AKDILSRVHYYDSSGSYGMDV | |
| IGHV1-2 (Human) | IGHJ6 (Human) | ARDLEDFWSGYPPLGYALDV | |
| IGHV3-7 (Human) | IGHJ5 (Human) | ARATYYYDSSDYGIN | |
| IGHV2-26 (Human) | IGHJ6 (Human) | ARIQLWLDGGGMDV | |
| IGHV3-53 (Human) | IGHJ3 (Human) | ARDLGSRGAFDI | |
| IGHV4-4 (Human) | IGHJ4 (Human) | AMVQGVMVY | |
| IGHV3-13 (Human) | IGHJ6 (Human) | ARSRYYDFWSGYYSVDDYGMDV | |
| IGHV1-46 (Human) | IGHJ4 (Human) | ARGRSDFLLQNFDY | |
| IGHV1-46 (Human) | IGHJ4 (Human) | ARGRSDFLLQNFDY | |
| IGHV4-61 (Human) | IGHJ4 (Human) | ARGLRFCSDTRCYPYFDY | |
| IGHV1-46 (Human) | IGHJ4 (Human) | ARGRSDFLLQNFDY | |
| IGHV1-18 (Human) | IGHJ6 (Human) | ARWIHRDFDWLPYGMFMDV | |
| IGHV1-18 (Human) | IGHJ6 (Human) | ARWIHRDFDWLPYGMFMDV | |
| IGHV1-18 (Human) | IGHJ6 (Human) | ARWIHRDFDWLPYGMFMDV | |
| IGHV3-30-3 (Human) | IGHJ4 (Human) | ARDLHEWELLAAGLDY | |
| IGHV4-31 (Human) | IGHJ3 (Human) | ATSLYGSSYYGTFNI | |
| IGHV4-59 (Human) | IGHJ5 (Human) | ARVVRWFDP | |
| IGHV3-30 (Human) | IGHJ3 (Human) | ARPRLGNYFDAFDI | |
| IGHV3-13 (Human) | IGHJ2 (Human) | ARVGYTYGFRYWYFDL | |
| IGHV1-69 (Human) | IGHJ6 (Human) | SGFGGDDGMDV | |
| IGHV3-53 (Human) | IGHJ4 (Human) | ATGRYDSIH | |
| IGHV2-26 (Human) | IGHJ3 (Human) | ARPLNPGFPDVFDI | |
| IGHV2-26 (Human) | IGHJ3 (Human) | ARPLNPGFPDVFDI | |
| IGHV4-31 (Human) | IGHJ4 (Human) | ARADIVVVPAAAFDY | |
| IGHV4-31 (Human) | IGHJ4 (Human) | ARSYLSGYEGHSFDY | |
| IGHV1-2 (Human) | IGHJ6 (Human) | ARETSFAIFGGGGMDV | |
| IGHV3-30-3 (Human) | IGHJ4 (Human) | ARTYSGSYYSYFDY | |
| IGHV3-30-3 (Human) | IGHJ4 (Human) | ARSSSGSYLYYFDY | |
| IGHV3-30-3 (Human) | IGHJ4 (Human) | ARPESGSYLGHFDS | |
| IGHV3-30-3 (Human) | IGHJ4 (Human) | ARDRSGSYFGPFDY | |
| IGHV3-30-3 (Human) | IGHJ4 (Human) | ARPRGGNYVGPFDY | |
| IGHV3-30-3 (Human) | IGHJ4 (Human) | AREDYYDSSGSFDH | |
| IGHV3-30-3 (Human) | IGHJ4 (Human) | AREDYYDSSGSLDY | |
| IGHV3-30-3 (Human) | IGHJ4 (Human) | ARTHGGNYGRYFDY | |
| IGHV3-30-3 (Human) | IGHJ4 (Human) | ARARGGGYNRLFDY | |
| IGHV3-30-3 (Human) | IGHJ4 (Human) | AREDYYDSSGSLDY | |
| IGHV1-69 (Human) | IGHJ4 (Human) | ATKPYYYDSSGYYGGFDY | |
| IGHV1-69 (Human) | IGHJ4 (Human) | ATKPYYYDSSGYYGGFDY | |
| IGHV1-69 (Human) | IGHJ4 (Human) | ARANYYYESSGYYFDY | |
| IGHV1-46 (Human) | IGHJ4 (Human) | ARDGPVLDWLFVFDY | |
| IGHV3-30-3 (Human) | IGHJ6 (Human) | ARGFGGNYYGMDV | |
| IGHV5-10-1 (Human) | IGHJ4 (Human) | ARLVPGQWHFDY | |
| IGHV3-33 (Human) | IGHJ6 (Human) | ARSFNEGQLGDAYHGMDV | |
| IGHV3-30 (Human) | IGHJ6 (Human) | AKVLTWSIHPEDYYYGMDV | |
| IGHV1-2 (Human) | IGHJ5 (Human) | ARDSSSWRYNWFDP | |
| IGHV4-39 (Human) | IGHJ2 (Human) | AGEGLRWYQLAHWYFDL | |
| IGHV7-4-1 (Human) | IGHJ4 (Human) | AGGYDFWSGYPNPTDY | |
| IGHV3-66 (Human) | IGHJ6 (Human) | ARDPSSRGYYGMDV | |
| IGHV4-61 (Human) | IGHJ6 (Human) | ARDLGLRYFDWLLRSDYYYYGMDV | |
| IGHV4-4 (Human) | IGHJ6 (Human) | ARAATILGLYYYYGMDV | |
| IGHV2-5 (Human) | IGHJ6 (Human) | AHRNPYGGEAGYYYYGLDV | |
| IGHV4-4 (Human) | IGHJ4 (Human) | AREPSYSSGCLDY | |
| IGHV3-30 (Human) | IGHJ6 (Human) | ARAYSGGYYYGMDV | |
| IGHV3-15 (Human) | IGHJ6 (Human) | TTSDYLRYYYYGIDV | |
| IGHV1-46 (Human) | IGHJ6 (Human) | ARDITVTTRDVYDYYYGMDV | |
| IGHV3-53 (Human) | IGHJ6 (Human) | ARGEYNWNDEDYYYGMDV | |
| IGHV3-21 (Human) | IGHJ3 (Human) | ARVRNHYYDSSGYYPDAFDI | |
| IGHV4-39 (Human) | IGHJ6 (Human) | AGESTDNNYYYYYAMDV | |
| IGHV3-30 (Human) | IGHJ4 (Human) | AKSDSELLPHFDY | |
| IGHV3-7 (Human) | IGHJ4 (Human) | ARARRAGSSGYYGFHFDY | |
| IGHV1-69-2 (Human) | IGHJ4 (Human) | GSGSEAHYGIDY | |
| IGHV4-31 (Human) | IGHJ6 (Human) | ATESLHYDILTGYSPTNYGMDV | |
| IGHV1-69 (Human) | IGHJ4 (Human) | ARVESYDNSGYYFDY | |
| IGHV1-69 (Human) | IGHJ4 (Human) | AENLQDDSGTLYSGG | |
| IGHV1-69 (Human) | IGHJ4 (Human) | AENLQDDSGTLYSGG | |
| IGHV4-38-2 (Human) | IGHJ2 (Human) | ARFGGEVLRYTDWPKPAWVDL | |
| IGHV1-58 (Human) | IGHJ3 (Human) | AAPHCSSTSCNDAFDI | |
| IGHV5-51 (Human) | IGHJ4 (Human) | ARHPSPYYYGSGSYSGGFDY | |
| IGHV1-58 (Human) | IGHJ3 (Human) | AAPDCSSTSCYDAFDI | |
| IGHV3-66 (Human) | IGHJ6 (Human) | ARDLVAYGMDV | |
| IGHV3-66 (Human) | IGHJ4 (Human) | ARGEGGSIVGVTSDY | |
| IGHV3-21 (Human) | IGHJ4 (Human) | ARKGSDRLSDNDPFDA | |
| IGHV4-31 (Human) | IGHJ2 (Human) | ARRGTDWYFDL | |
| IGHV5-51 (Human) | IGHJ3 (Human) | VREEIVGGARGPIDT | |
| IGHV3-64D (Human) | IGHJ3 (Human) | VKDLAVQDYDSSGYFDPGDI | |
| IGHV3-21 (Human) | IGHJ6 (Human) | AREGITSFGVVTHYYALDV | |
| IGHV1-18 (Human) | IGHJ6 (Human) | ARVNYSYYSSSFFDYFYYYGMDV | |
| IGHV3-30-3 (Human) | IGHJ4 (Human) | ARPHGGSYYSYFDY | |
| IGHV5-51 (Human) | IGHJ6 (Human) | ARHETQHDSSGTYYLGMDV | |
| IGHV3-30-3 (Human) | IGHJ3 (Human) | VRFYSPLHDLVIHYGNY | |
| IGHV5-51 (Human) | IGHJ3 (Human) | TREAIVAGARGPIDF | |
| IGHV5-51 (Human) | IGHJ3 (Human) | AREAIVEGARGPFDF | |
| IGHV5-51 (Human) | IGHJ3 (Human) | AREEIVEAARGPFDV | |
| IGHV3-30-3 (Human) | IGHJ3 (Human) | ARTYSGSYWDAFDI | |
| IGHV3-30-3 (Human) | IGHJ4 (Human) | VRFYSPLHDLVIHYGNY | |
| IGHV1-24 (Human) | IGHJ3 (Human) | ATDSIFGVVIYAFAF | |
| IGHV5-51 (Human) | IGHJ3 (Human) | AREEVLEAARGPFDV | |
| IGHV4-4 (Human) | IGHJ5 (Human) | ARLFSDSGNYFWFDP | |
| IGHV5-51 (Human) | IGHJ6 (Human) | ARHETQHDSSGTYYLGMDV | |
| IGHV5-51 (Human) | IGHJ3 (Human) | ARHETQHDSSGTYYLGMDV | |
| IGHV3-21 (Human) | IGHJ6 (Human) | AREGITNFGVVTHYYALDV | |
| IGHV3-30-3 (Human) | IGHJ4 (Human) | VRFYSPLHDLVIHYGNY | |
| IGHV1-24 (Human) | IGHJ4 (Human) | TTESIFGVVIYAFAI | |
| IGHV2-5 (Human) | IGHJ4 (Human) | AHIVIAVARYYFDY | |
| IGHV5-51 (Human) | IGHJ2 (Human) | TREAILGGARGPIDL | |
| IGHV3-21 (Human) | IGHJ6 (Human) | AREGITSFGIVTHYYALDV | |
| IGHV4-4 (Human) | IGHJ4 (Human) | AMVQGVMVY | |
| IGHV1-46 (Human) | IGHJ4 (Human) | ARGRSDFLLQNFDY | |
| IGHV3-30 (Human) | IGHJ6 (Human) | AQLEGDSNSYGYDYYYYYGMDV | |
| IGHV4-30-2 (Human) | IGHJ4 (Human) | ARVQGYCTGGVCYSVPYYFDF | |
| IGHV1-69-2 (Human) | IGHJ4 (Human) | GSGSEAHYGIDY | |
| IGHV3-30-3 (Human) | IGHJ4 (Human) | ARDSRGYGHFDY | |
| IGHV3-30-5 (Human) | IGHJ4 (Human) | AKTIWGTYRSPFDY | |
| IGHV4-30-4 (Human) | IGHJ5 (Human) | ARGQSHVLLWFGELLGWFDP | |
| IGHV4-30-4 (Human) | IGHJ5 (Human) | ARGQSHVLLWFGELLGWFDP | |
| IGHV1-18 (Human) | IGHJ6 (Human) | ARWIHRDFDWLPYGMFMDV | |
| IGHV1-46 (Human) | IGHJ4 (Human) | ARDGPVLDWLFVFDY | |
| IGHV3-23 (Human) | IGHJ4 (Human) | AKGVHGTSGWYPPRAFDY | |
| IGHV1-46 (Human) | IGHJ4 (Human) | AILVVYGMRDY | |
| IGHV3-30-3 (Human) | IGHJ5 (Human) | ARSGSGSYFNWFDP | |
| IGHV3-33 (Human) | IGHJ2 (Human) | ARGPQEQYDILTGYYFPANWYFDL | |
| IGHV4-59 (Human) | IGHJ6 (Human) | ARGPEYYDFWSGYPGGMDV | |
| IGHV3-7 (Human) | IGHJ4 (Human) | ARLGGSSWYWDY | |
| IGHV1-69 (Human) | IGHJ4 (Human) | ARANYYYESSGYYFDY | |
| IGHV4-61 (Human) | IGHJ3 (Human) | ARADRERVPYFGYDFWSGYPDAFDI | |
| IGHV4-59 (Human) | IGHJ4 (Human) | AREVRWNWFDT | |
| IGHV3-30-3 (Human) | IGHJ4 (Human) | ARSLAAYWYFDY | |
| IGHV4-39 (Human) | IGHJ4 (Human) | ARRMNYDSSGYYYVHFDY | |
| IGHV2-26 (Human) | IGHJ3 (Human) | ARPLSPGFPDVFDI | |
| IGHV2-5 (Human) | IGHJ5 (Human) | THRPIPAAGTLVFLNWFDP | |
| IGHV1-18 (Human) | IGHJ6 (Human) | ARWIHRDFDWLPYGMFMDV | |
| IGHV1-69 (Human) | IGHJ6 (Human) | ARAPQATVTTFNYYYGLDV | |
| IGHV6-1 (Human) | IGHJ6 (Human) | ARAPGIAARLGLYGMDV | |
| IGHV3-33 (Human) | IGHJ6 (Human) | ARGADILTGYYNYYYYGMDV | |
| IGHV3-53 (Human) | IGHJ5 (Human) | AREGAAANTHGWFDP | |
| IGHV3-53 (Human) | IGHJ4 (Human) | ARQLGDYVDY | |
| IGHV4-4 (Human) | IGHJ2 (Human) | ARGFTNWYFDL | |
| IGHV1-69 (Human) | IGHJ4 (Human) | ARAGYYYDSSGYPFDY | |
| IGHV1-69 (Human) | IGHJ4 (Human) | ARAGYYYESSGYHFDY | |
| IGHV4-59 (Human) | IGHJ6 (Human) | ARGTFTLGYYYGMDV | |
| IGHV1-2 (Human) | IGHJ4 (Human) | ATARCSGDLCPFDY | |
| IGHV3-21 (Human) | IGHJ6 (Human) | AREGITSFGIVTHYYALDV | |
| IGHV1-24 (Human) | IGHJ6 (Human) | ATDSIFGVVIYAFAF | |
| IGHV3-73 (Human) | IGHJ4 (Human) | TVRLVPVDKFDY | |
| IGHV4-4 (Human) | IGHJ5 (Human) | ARLFADRGGYFWFDP | |
| IGHV3-33 (Human) | IGHJ5 (Human) | ARDQDFWSGYYLP | |
| IGHV4-4 (Human) | IGHJ3 (Human) | ARWGSGWSNDAFDI | |
| IGHV3-33 (Human) | IGHJ4 (Human) | ASEPIVAARTSFETDY | |
| IGHV5-51 (Human) | IGHJ3 (Human) | AREEIVEAARGPFDV | |
| IGHV3-33 (Human) | IGHJ4 (Human) | ARDRPFPSSWFGEDFDY | |
| IGHV5-51 (Human) | IGHJ6 (Human) | ARHETQHDSSGTYYLGMDV | |
| IGHV3-21 (Human) | IGHJ6 (Human) | AAEGISVFGVVTHFYALAA | |
| IGHV1-2 (Human) | IGHJ4 (Human) | ASPKTDSTGSTLPLFD | |
| IGHV1-24 (Human) | IGHJ4 (Human) | TTESIFGVVIYAFAI | |
| IGHV3-30 (Human) | IGHJ6 (Human) | AKENRDRNYDSWSASYSDYYYGMDV | |
| IGHV3-33 (Human) | IGHJ4 (Human) | ARAPGGGDLGFY | |
| IGHV3-33 (Human) | IGHJ4 (Human) | ASDYYDSSGYPWDYYFDY | |
| IGHV4-4 (Human) | IGHJ5 (Human) | VRLTYVNSGYNYFDP | |
| IGHV3-21 (Human) | IGHJ6 (Human) | ARPHNPAAYCSGGSCYSAYYYYYGMDV | |
| IGHV1-2 (Human) | IGHJ3 (Human) | ARSGGLGILAFDL | |
| IGHV4-39 (Human) | IGHJ4 (Human) | AGASDLDYFDY | |
| IGHV4-39 (Human) | IGHJ4 (Human) | ARRGYYDFWSGPIFDY | |
| IGHV3-30 (Human) | IGHJ6 (Human) | AKENRDRNYDSWSGSYSDYYYGMDV | |
| IGHV3-33 (Human) | IGHJ4 (Human) | ARDFMWKDQLLFGYQ | |
| IGHV3-30-3 (Human) | IGHJ4 (Human) | VRFYSPLHDLVIHYGNY | |
| IGHV3-30-3 (Human) | IGHJ4 (Human) | VRFYSPLHDLVIHYGNY | |
| IGHV5-51 (Human) | IGHJ3 (Human) | AREEIVEAARGPFDV | |
| IGHV5-51 (Human) | IGHJ3 (Human) | AREEIVEGARGPFDV | |
| IGHV3-30-3 (Human) | IGHJ4 (Human) | VRFYSPLHDLVIHYGNY | |
| IGHV3-53 (Human) | IGHJ4 (Human) | ARAYGDYFFDY | |
| IGHV1-2 (Human) | IGHJ5 (Human) | ARENRGSGRGWFDP | |
| IGHV4-4 (Human) | IGHJ4 (Human) | ARRYCSGGTCSPFDY | |
| IGHV3-30 (Human) | IGHJ4 (Human) | AKDTSVVVIPIFLFDY | |
| IGHV3-30-3 (Human) | IGHJ5 (Human) | ARSGSGNYYHWFDP | |
| IGHV3-30-3 (Human) | IGHJ5 (Human) | ARPESGSYLGHFDS | |
| IGHV1-18 (Human) | IGHJ6 (Human) | ARWIHRDFDWLPYGMFMDV | |
| IGHV3-53 (Human) | IGHJ6 (Human) | ARDRRITMVRGVIYYYYGMDV | |
| IGHV3-30-3 (Human) | IGHJ6 (Human) | ARANGGNYYYGMDV | |
| IGHV3-30-3 (Human) | IGHJ6 (Human) | ARANGGNYYYGMDV | |
| IGHV1-18 (Human) | IGHJ6 (Human) | ARWIHRDFDWLPYGMFMDV | |
| IGHV1-3 (Human) | IGHJ6 (Human) | ARDLSPKDFLGSSLGGRDV | |
| IGHV3-30-3 (Human) | IGHJ3 (Human) | ARPRLGNYFDAFDI | |
| IGHV4-34 (Human) | IGHJ6 (Human) | ARMVVQGHLQPYYYGMDV | |
| IGHV3-30-3 (Human) | IGHJ4 (Human) | AKDLAFSGSFPFLDY | |
| IGHV3-33 (Human) | IGHJ4 (Human) | ARDGMVGATTGIDY | |
| IGHV3-30 (Human) | IGHJ4 (Human) | AKSDSELLPHFDY | |
| IGHV3-11 (Human) | IGHJ6 (Human) | ARDAVAGPRRELYYYYGMDI | |
| IGHV1-46 (Human) | IGHJ4 (Human) | ARDGPVLDWLFVFDY | |
| IGHV3-30-3 (Human) | IGHJ4 (Human) | ARDSRGYGHFDY | |
| IGHV3-30-3 (Human) | IGHJ5 (Human) | AKSPPASYYTWFHP | |
| IGHV1-69 (Human) | IGHJ4 (Human) | ARVPPSTGYDFYFDY | |
| IGHV3-30 (Human) | IGHJ4 (Human) | AKAQWVGFSPLDY | |
| IGHV3-30-3 (Human) | IGHJ5 (Human) | ARSGSGNYYHWFDP | |
| IGHV3-30-3 (Human) | IGHJ5 (Human) | ARSGSGNYYHWFDP | |
| IGHV3-15 (Human) | IGHJ4 (Human) | TTDRDYDYIWGSYRYRDY | |
| IGHV4-4 (Human) | IGHJ2 (Human) | ARGFTNWYFDL | |
| IGHV3-30-3 (Human) | IGHJ4 (Human) | ARPRGGNYVGPFDY | |
| IGHV3-30 (Human) | IGHJ6 (Human) | ATDPASDSLYYYYGMDV | |
| IGHV3-48 (Human) | IGHJ4 (Human) | ARDGPTVTHYFDY | |
| IGHV2-26 (Human) | IGHJ6 (Human) | ARIQLWLDGGGMDV | |
| IGHV3-30-3 (Human) | IGHJ4 (Human) | ARDREGAYWGPIDY | |
| IGHV4-59 (Human) | IGHJ5 (Human) | AGSYSYGYSWFDP | |
| IGHV3-53 (Human) | IGHJ1 (Human) | ATGRYDSIH | |
| IGHV4-59 (Human) | IGHJ4 (Human) | AREVRWNWFDT | |
| IGHV3-33 (Human) | IGHJ4 (Human) | ARATPHRRDQGPFHY | |
| IGHV4-31 (Human) | IGHJ5 (Human) | SKDQGPVFGVDNWFDP | |
| IGHV3-33 (Human) | IGHJ4 (Human) | ARDYQVGGDYRSDF | |
| IGHV4-61 (Human) | IGHJ6 (Human) | AREVAFFDWLLQSDYYHGMDV | |
| IGHV5-51 (Human) | IGHJ3 (Human) | ARRGNGGYDAFDV | |
| IGHV4-4 (Human) | IGHJ4 (Human) | ARRYCSGGTCSPFDY | |
| IGHV3-48 (Human) | IGHJ4 (Human) | ATSAVGATLGY | |
| IGHV3-33 (Human) | IGHJ6 (Human) | ARDDSSGWYRPLYYYYYGMDV | |
| IGHV1-46 (Human) | IGHJ6 (Human) | ARDTSNAEDFWSAPILGYYYGMDV | |
| IGHV4-31 (Human) | IGHJ3 (Human) | ARGGPEITMIAIDAFDI | |
| IGHV4-39 (Human) | IGHJ6 (Human) | ARPGVYCSGASCYYYLNMDV | |
| IGHV4-34 (Human) | IGHJ4 (Human) | ARIEYSYGRRGLDY | |
| IGHV4-59 (Human) | IGHJ4 (Human) | ARGFDY | |
| IGHV1-69 (Human) | IGHJ6 (Human) | ARIHTDLAVADPDLGFYSMDV | |
| IGHV3-30-3 (Human) | IGHJ6 (Human) | ARSTGGSYYYGMDV | |
| IGHV3-53 (Human) | IGHJ4 (Human) | ARDYGDFYFDY | |
| IGHV4-59 (Human) | IGHJ4 (Human) | ARGFDY | |
| IGHV3-30-3 (Human) | IGHJ4 (Human) | ASLIAAAGGRDY | |
| IGHV3-30-3 (Human) | IGHJ6 (Human) | ARSTGGSYYYGMDV | |
| IGHV1-46 (Human) | IGHJ4 (Human) | ARDHRKRGGDYGGNIFPGFDY | |
| IGHV3-30 (Human) | IGHJ4 (Human) | AKEVGYSDYDGWDY | |
| IGHV3-30-3 (Human) | IGHJ5 (Human) | ARGGGGNYFFFDS | |
| IGHV3-33 (Human) | IGHJ3 (Human) | VRDRPPDYFDSSATPDAFDI | |
| IGHV3-15 (Human) | IGHJ4 (Human) | TTVHSDYVWGSYRKGYFDY | |
| IGHV4-4 (Human) | IGHJ2 (Human) | ARGFTNWYFDL | |
| IGHV3-48 (Human) | IGHJ4 (Human) | ARMGSRHTRPYSSGWYPDPDDY | |
| IGHV1-18 (Human) | IGHJ3 (Human) | ARDGYYDSSGYRFYWRDDAFDI | |
| IGHV1-18 (Human) | IGHJ6 (Human) | SSTSCYGGNYYYYGMDV | |
| IGHV2-5 (Human) | IGHJ4 (Human) | AHYIAIRPFDY | |
| IGHV4-39 (Human) | IGHJ3 (Human) | VRQNHNMLQGPHHVFDI | |
| IGHV3-23 (Human) | IGHJ5 (Human) | ARNRTDLSGAAWFDS | |
| IGHV3-7 (Human) | IGHJ4 (Human) | AGLSSSSWNLDY | |
| IGHV3-30-3 (Human) | IGHJ6 (Human) | ARARSGSYYYGMDV | |
| IGHV1-69-2 (Human) | IGHJ4 (Human) | GSGSEAHYGIDY | |
| IGHV4-59 (Human) | IGHJ6 (Human) | ARVLVPISGVDV | |
| IGHV1-46 (Human) | IGHJ3 (Human) | ARDPRSSGDNDGFDM | |
| IGHV3-66 (Human) | IGHJ6 (Human) | ARDLNYYGMDV | |
| IGHV1-69-2 (Human) | IGHJ1 (Human) | ATGPGATRATIYGFAH | |
| IGHV3-7 (Human) | IGHJ2 (Human) | ARLSVLGWYFDL | |
| IGHV5-51 (Human) | IGHJ5 (Human) | ARLWHCGGDCYPKLNWLDP | |
| IGHV3-48 (Human) | IGHJ5 (Human) | ARDSGAIFDS | |
| IGHV3-21 (Human) | IGHJ4 (Human) | AREGRIAGIPTGGLFDY | |
| IGHV1-8 (Human) | IGHJ5 (Human) | ARGELDDDIWGTYSMRWFDP | |
| IGHV2-5 (Human) | IGHJ5 (Human) | AHRSGELLWFRGEDWFDP | |
| IGHV3-30-3 (Human) | IGHJ6 (Human) | ARDLAYWDTDYGMDV | |
| IGHV2-5 (Human) | IGHJ5 (Human) | AHRRGLLMYNCFDP | |
| IGHV3-53 (Human) | IGHJ6 (Human) | ARDLAERGGMDV | |
| IGHV6-1 (Human) | IGHJ5 (Human) | ARDWGPQYCSGGSCYSTSRWFDP | |
| IGHV6-1 (Human) | IGHJ5 (Human) | ARDWGPQYCSGGSCYSTSRWFDP | |
| IGHV4-31 (Human) | IGHJ4 (Human) | ASSGRLTIYY | |
| IGHV4-39 (Human) | IGHJ4 (Human) | ASQQWLRGNFDY | |
| IGHV1-69 (Human) | IGHJ5 (Human) | ARLQNDRLFLSWFDP | |
| IGHV3-33 (Human) | IGHJ4 (Human) | AREFGSTPIYDIMTGPFDY | |
| IGHV1-69 (Human) | IGHJ4 (Human) | ARAGYYYDSSPYPFDY | |
| IGHV1-69 (Human) | IGHJ4 (Human) | ARAGYYYDSSPYPFDY | |
| IGHV3-21 (Human) | IGHJ4 (Human) | ARDFSGHTAVAGTGFEY | |
| IGHV3-66 (Human) | IGHJ4 (Human) | VRASPPGGNTGWPFFED | |
| IGHV3-30 (Human) | IGHJ4 (Human) | AKKGSPYCGVDCYKGYFDY | |
| IGHV1-69 (Human) | IGHJ6 (Human) | VRDSDPYTATVTSNHYWYAMDV | |
| IGHV1-69 (Human) | IGHJ6 (Human) | AREEYSGTVHNFFGMDV | |
| IGHV3-30 (Human) | IGHJ4 (Human) | ARAKGGSYSNAFDY | |
| IGHV1-69 (Human) | IGHJ3 (Human) | ARDPSIHYTGNHHWYDLDI | |
| IGHV1-69 (Human) | IGHJ6 (Human) | VRDSDPYTATYRNNHYWYAMDV | |
| IGHV1-69 (Human) | IGHJ3 (Human) | ARDPSIHYTGNHHWYDLDI | |
| IGHV4-34 (Human) | IGHJ5 (Human) | ARGQESPIVGVTGRWFDP | |
| IGHV4-34 (Human) | IGHJ5 (Human) | ARGSLSREYDFLTAPQNGPWFDS | |
| IGHV3-64D (Human) | IGHJ1 (Human) | VKDGGYYDSSGPGH | |
| IGHV4-59 (Human) | IGHJ2 (Human) | ARVGWVRYFDWSKPYYYFDL | |
| IGHV3-23 (Human) | IGHJ5 (Human) | AKCDLVRYFDWLGEENNWFDP | |
| IGHV1-69 (Human) | IGHJ3 (Human) | VRDSDPYTATVRNNHYWYALDV | |
| IGHV3-66 (Human) | IGHJ4 (Human) | ARATPPGGGTGWPYFDF | |
| IGHV1-69 (Human) | IGHJ6 (Human) | AREEPSGTYHNYYGLDV | |
| IGHV4-4 (Human) | IGHJ3 (Human) | AARIRGATHYDFWSGFWAGPFDI | |
| IGHV4-4 (Human) | IGHJ3 (Human) | ARTQSNDFWSGYYTAAFDL | |
| IGHV4-34 (Human) | IGHJ5 (Human) | ARGSLSREYDFLTAPQNGPWFDS | |
| IGHV1-69 (Human) | IGHJ6 (Human) | VLDTTSANPHNWYGMDV | |
| IGHV3-11 (Human) | IGHJ4 (Human) | ARMGPYGSGSFDY | |
| IGHV4-38-2 (Human) | IGHJ4 (Human) | AGRHQELLPMGSFDM | |
| IGHV1-69 (Human) | IGHJ3 (Human) | ARDPSILNTGNHHWYDLDI | |
| IGHV1-69 (Human) | IGHJ4 (Human) | VRDGAYDSSGYYSTQ | |
| IGHV1-69 (Human) | IGHJ6 (Human) | ARDPTFLNSGNHFWYAVDI | |
| IGHV1-69 (Human) | IGHJ6 (Human) | ARDPTFLNTGNHFWYAVDI | |
| IGHV1-2 (Human) | IGHJ5 (Human) | ARDFGVRYDDSRQLMKYCDS | |
| IGHV1-69 (Human) | IGHJ6 (Human) | ARDPSILNTGNHHWYDLDL | |
| IGHV1-69 (Human) | IGHJ6 (Human) | VVDTTMADPHNWYGLDV | |
| IGHV4-4 (Human) | IGHJ3 (Human) | ARVRVGASHHNFWSGYYTDAFDI | |
| IGHV1-69 (Human) | IGHJ4 (Human) | ARDPSFLNTGNHFWYDFDM | |
| IGHV1-69 (Human) | IGHJ4 (Human) | VRDGAYDSSGYYSTQ | |
| IGHV3-21 (Human) | IGHJ4 (Human) | ARDFPGDTAVAGTGFNY | |
| IGHV3-49 (Human) | IGHJ4 (Human) | SRDLRRGYYDSNGHQQFDL | |
| IGHV3-30 (Human) | IGHJ6 (Human) | ARDPGPITFFDWSPDKSRKSYYDYNGMDV | |
| IGHV3-30 (Human) | IGHJ4 (Human) | ARDLPPLDY | |
| IGHV1-69 (Human) | IGHJ6 (Human) | VRDSDPYTTTFSHNHYWYAMDV | |
| IGHV3-64D (Human) | IGHJ4 (Human) | VKDNVILPGAIVRPQFDY | |
| IGHV1-69 (Human) | IGHJ6 (Human) | ARDPSILNTGNHHWYDLDM | |
| IGHV3-30 (Human) | IGHJ4 (Human) | ARDNVVQQNADNVGYFDF | |
| IGHV1-69 (Human) | IGHJ6 (Human) | ARDPSILNTGNHHWYDLDL | |
| IGHV1-69 (Human) | IGHJ6 (Human) | VRDSDPYTATSRNNHYWYGMDV | |
| IGHV1-69 (Human) | IGHJ3 (Human) | ARDPSILNTGNHHWYDLDM | |
| IGHV3-49 (Human) | IGHJ6 (Human) | AREGTSLGYYYYYAMDV | |
| IGHV1-69 (Human) | IGHJ6 (Human) | ARDPSILNTGNHHWYDLDM | |
| IGHV3-7 (Human) | IGHJ3 (Human) | ARGPIRHFGLDAFDI | |
| IGHV1-69 (Human) | IGHJ6 (Human) | VRDSDPYTATSRNNHYWYGMDV | |
| IGHV1-69 (Human) | IGHJ3 (Human) | ARDPSILNTGNHHWYDLDM | |
| IGHV3-64D (Human) | IGHJ3 (Human) | VKDLGATVTYDVFDV | |
| IGHV3-23 (Human) | IGHJ4 (Human) | AKERDLPGRGGYFDH | |
| IGHV4-34 (Human) | IGHJ5 (Human) | ARGYTAPIIREVPITFRPRWFDP | |
| IGHV3-11 (Human) | IGHJ4 (Human) | ARMGPYGSGTFDY | |
| IGHV3-30 (Human) | IGHJ5 (Human) | ARDHALQNGRPGYFDS | |
| IGHV3-64 (Human) | IGHJ4 (Human) | VREGQQWLGLYFDH | |
| IGHV1-8 (Human) | IGHJ4 (Human) | AREKKSFGPQYYYGSGED | |
| IGHV5-51 (Human) | IGHJ3 (Human) | VRQRYCSGGSCFLFEDAFEI | |
| IGHV3-48 (Human) | IGHJ5 (Human) | VRDTDWAFDS | |
| IGHV2-5 (Human) | IGHJ4 (Human) | AHTSELPPRRPYAAFDF | |
| IGHV3-23 (Human) | IGHJ4 (Human) | ANTNFLDY | |
| IGHV4-4 (Human) | IGHJ6 (Human) | TRSFISFDSSGHPYYYYAMDV | |
| IGHV1-69 (Human) | IGHJ4 (Human) | TRETGTDEFDF | |
| IGHV4-4 (Human) | IGHJ6 (Human) | ATMWGGLCTASNCYGNPMDV | |
| IGHV4-4 (Human) | IGHJ6 (Human) | ATMWGGLCTASNCYGNPMDV | |
| IGHV4-4 (Human) | IGHJ6 (Human) | ATMWGGLCTASNCYGNPMDV | |
| IGHV3-74 (Human) | IGHJ4 (Human) | ARDLAWTFFDY | |
| IGHV3-66 (Human) | IGHJ4 (Human) | ARATPPGGTTGWPYIDL | |
| IGHV3-23 (Human) | IGHJ4 (Human) | ANTNFLDY | |
| IGHV3-9 (Human) | IGHJ6 (Human) | AKLGTDHPIGVDV | |
| IGHV3-64D (Human) | IGHJ4 (Human) | VKALYSSSWCPFDY | |
| IGHV3-48 (Human) | IGHJ6 (Human) | ARELDSETYYNYNSLDV | |
| IGHV4-59 (Human) | IGHJ5 (Human) | AKAQGIYYRGWSYWFDP | |
| IGHV1-46 (Human) | IGHJ5 (Human) | AREARRQVTQWFGEFWGPYNWFDP | |
| IGHV4-34 (Human) | IGHJ4 (Human) | ARGLISYTLWLRESYFDY | |
| IGHV1-69 (Human) | IGHJ6 (Human) | ARDPSILNTGNHHWYDLDI | |
| IGHV1-18 (Human) | IGHJ4 (Human) | ARDYGDGPPDH | |
| IGHV1-69 (Human) | IGHJ6 (Human) | VLDTTMSHPHNWYGMDV | |
| IGHV1-69 (Human) | IGHJ4 (Human) | ASEYFDGRSYHSFCGLDV | |
| IGHV1-69 (Human) | IGHJ6 (Human) | ARDPSILNTGPHHWYDLDI | |
| IGHV1-69 (Human) | IGHJ6 (Human) | VRDSEPYTATRSQNHYWYDMDV | |
| IGHV4-4 (Human) | IGHJ6 (Human) | AREFGVRFLDRSLFGAMDV | |
| IGHV3-30 (Human) | IGHJ3 (Human) | ARAGGYLSAFDI | |
| IGHV1-69 (Human) | IGHJ3 (Human) | ARDPSFLNAGNHFYYDFDV | |
| IGHV4-4 (Human) | IGHJ4 (Human) | ARAGNIVVMPAAQYYFDY | |
| IGHV3-30 (Human) | IGHJ5 (Human) | VRDDVLQHSRPSGPGYFVS | |
| IGHV4-59 (Human) | IGHJ4 (Human) | ARGSQIDLRGGLGATFFDY | |
| IGHV1-69 (Human) | IGHJ6 (Human) | ARDSDPYTATRRHNHYWYAMDV | |
| IGHV3-48 (Human) | IGHJ5 (Human) | VRDTDWAFDS | |
| IGHV1-8 (Human) | IGHJ6 (Human) | ARRGNNFGYYYYYTVDV | |
| IGHV1-46 (Human) | IGHJ3 (Human) | ARVLAGSSHEWQLTHDAFDI | |
| IGHV3-23 (Human) | IGHJ4 (Human) | AKGDWIRYFDWSLPISFFDY | |
| IGHV4-61 (Human) | IGHJ4 (Human) | ARTTSPLTYSGHWPLFDY | |
| IGHV1-69 (Human) | IGHJ6 (Human) | VRDSDPYTATSRNNHYWYGMDV | |
| IGHV1-69 (Human) | IGHJ6 (Human) | ARDPSILNTGNHHWYDLDI | |
| IGHV1-69 (Human) | IGHJ3 (Human) | ARDPSILNTGNHHWYDLDI | |
| IGHV3-66 (Human) | IGHJ5 (Human) | VRMDWMEWMKYYFDS | |
| IGHV1-69 (Human) | IGHJ3 (Human) | ARDPSILNTGNHHWYDLDM | |
| IGHV3-30-3 (Human) | IGHJ4 (Human) | ARPQSGGYYAPLDY | |
| IGHV3-30 (Human) | IGHJ6 (Human) | AKVSATYYYYYYGMDV | |
| IGHV3-30 (Human) | IGHJ4 (Human) | ARDTATYVLLWSGDFNLDY | |
| IGHV3-30-3 (Human) | IGHJ6 (Human) | ARGHTGNYYYGMDV | |
| IGHV3-30 (Human) | IGHJ4 (Human) | AKGGDSSGWAWDGDNPPTDY | |
| IGHV3-9 (Human) | IGHJ4 (Human) | AMGPFGELLPYYFDY | |
| IGHV1-24 (Human) | IGHJ5 (Human) | AAAPAVMTAGWFDP | |
| IGHV4-59 (Human) | IGHJ5 (Human) | VRGAMAWFDP | |
| IGHV1-24 (Human) | IGHJ4 (Human) | ATQPAAIGGTPPYY | |
| IGHV1-18 (Human) | IGHJ6 (Human) | ARDQGPTYYYGSGSPHYGMDV | |
| IGHV3-30 (Human) | IGHJ4 (Human) | AKGGDSSGWAWDGDNPPTDY | |
| IGHV1-24 (Human) | IGHJ6 (Human) | ATSFPIRGDPSYYYYYYGMDV | |
| IGHV3-30 (Human) | IGHJ6 (Human) | AIYGYYYYGLDV | |
| IGHV3-30 (Human) | IGHJ4 (Human) | AKGGDSSGWAWDGDNPPTDY | |
| IGHV4-4 (Human) | IGHJ3 (Human) | ARPTAGAGGAFDI | |
| IGHV2-26 (Human) | IGHJ5 (Human) | ARTEWLLSDNWFDS | |
| IGHV1-46 (Human) | IGHJ3 (Human) | ARGGLVPAARNAFDI | |
| IGHV4-4 (Human) | IGHJ3 (Human) | ARPTAGAGGAFDT | |
| IGHV1-18 (Human) | IGHJ5 (Human) | ARDLPIKVVVPAADYNWFDP | |
| IGHV2-5 (Human) | IGHJ3 (Human) | AHRRGILTEDAFDI | |
| IGHV3-53 (Human) | IGHJ6 (Human) | ARDLNEHGLDV | |
| IGHV1-46 (Human) | IGHJ3 (Human) | ARGTLIPAHRGAFDI | |
| IGHV4-61 (Human) | IGHJ6 (Human) | ARARPDYYYYYAMDV | |
| IGHV3-9 (Human) | IGHJ6 (Human) | AKAHSTGHQYYYGMDV | |
| IGHV1-2 (Human) | IGHJ4 (Human) | ARVVVLGYGRPNNYYDGRNVWDY | |
| IGHV2-70 (Human) | IGHJ4 (Human) | ARGVVTYDY | |
| IGHV1-69 (Human) | IGHJ6 (Human) | ARGRGYSNYGASYYMDV | |
| IGHV3-9 (Human) | IGHJ6 (Human) | AKGRGAGYTSYMDV | |
| IGHV1-8 (Human) | IGHJ4 (Human) | ARMRTGWPTHGRPDDF | |
| IGHV3-53 (Human) | IGHJ3 (Human) | ARSYDILTGYRDAFDI | |
| IGHV4-34 (Human) | IGHJ6 (Human) | ARVGYSQGYYYYYMDV | |
| IGHV1-58 (Human) | IGHJ3 (Human) | AAPHCNRTSCYDAFDL | |
| IGHV1-69 (Human) | IGHJ2 (Human) | ARVGVSGFKSGSNWYFDL | |
| IGHV3-66 (Human) | IGHJ6 (Human) | ARDLVTYGLDV | |
| IGHV3-20 (Human) | IGHJ3 (Human) | AVIMSPIPRYSGYDWAGGAFDI | |
| IGHV3-30 (Human) | IGHJ4 (Human) | AKNLGPYCSGGTCYSLVGDY | |
| IGHV3-20 (Human) | IGHJ3 (Human) | AVIMSPIPRYSGYDWAGDAFDI | |
| IGHV1-8 (Human) | IGHJ4 (Human) | ARMRSGWPTHGRPDDF | |
| IGHV3-9 (Human) | IGHJ6 (Human) | AKDIIRQGEDGMDV | |
| IGHV3-23 (Human) | IGHJ3 (Human) | VKGLFDWFPL | |
| IGHV3-7 (Human) | IGHJ4 (Human) | ARLGFYYGGADY | |
| IGHV3-9 (Human) | IGHJ4 (Human) | AKGVDYGGKLAYFDS | |
| IGHV3-30-3 (Human) | IGHJ4 (Human) | ARDIDSGYDPTPVFDY | |
| IGHV3-13 (Human) | IGHJ5 (Human) | ARVLYDSSGFYNWFDP | |
| IGHV3-53 (Human) | IGHJ5 (Human) | ARDFLRWHDL | |
| IGHV1-69 (Human) | IGHJ6 (Human) | ARGRGYSNYGASYYMDV | |
| IGHV4-39 (Human) | IGHJ5 (Human) | ARILVIFTLNWFDP | |
| IGHV3-53 (Human) | IGHJ5 (Human) | ARDFLRWHDL | |
| IGHV4-61 (Human) | IGHJ4 (Human) | AGSPVPPTIVGASY | |
| IGHV5-51 (Human) | IGHJ2 (Human) | ARRGEAAGIWYFDL | |
| IGHV3-66 (Human) | IGHJ6 (Human) | AKEGGSGSLRYYYYGMDV | |
| IGHV3-48 (Human) | IGHJ6 (Human) | ARRSYRSSWYYYYGMDV | |
| IGHV3-30-3 (Human) | IGHJ6 (Human) | ARSTSGSYYYGMDV | |
| IGHV3-30-3 (Human) | IGHJ3 (Human) | ARDWAPTYYDMPSAFDI | |
| IGHV1-69 (Human) | IGHJ1 (Human) | ARIGSYPEYFQH | |
| IGHV4-31 (Human) | IGHJ4 (Human) | ARGGSGSYSLFDY | |
| IGHV3-30-3 (Human) | IGHJ6 (Human) | ARSTSGSYYYGMDV | |
| IGHV3-30-3 (Human) | IGHJ6 (Human) | ARSTSGSYYYGMDV | |
| IGHV3-66 (Human) | IGHJ6 (Human) | ARDLVTYGLDV | |
| IGHV1-69 (Human) | IGHJ4 (Human) | ARIGHFDSSGYYLDY | |
| IGHV3-7 (Human) | IGHJ4 (Human) | ARLSGSSWDFDY | |
| IGHV3-20 (Human) | IGHJ6 (Human) | ARRRSSSRYSSGWYMYYYYMDV | |
| IGHV3-7 (Human) | IGHJ4 (Human) | ARLSGSSWDFDY | |
| IGHV2-5 (Human) | IGHJ3 (Human) | AHRLWFRDAFDI | |
| IGHV3-7 (Human) | IGHJ4 (Human) | ARVGSSSWYFDY | |
| IGHV3-30-3 (Human) | IGHJ4 (Human) | ARADTMVRGTYFEY | |
| IGHV3-30-3 (Human) | IGHJ4 (Human) | ARGGATNFDY | |
| IGHV3-9 (Human) | IGHJ5 (Human) | TKASRYCSSTICYWNWFDP | |
| IGHV3-48 (Human) | IGHJ4 (Human) | AREARSRYFDWLPSYYFDY | |
| IGHV3-20 (Human) | IGHJ6 (Human) | ARRRSSSRYSSGWYMYYYYMDV | |
| IGHV3-30 (Human) | IGHJ4 (Human) | AKGGDGSGWAWDGDNPPTDY | |
| IGHV5-51 (Human) | IGHJ2 (Human) | ARPDYSSGWFSYWYFDL | |
| IGHV1-24 (Human) | IGHJ4 (Human) | ATGFAVFGRAAVPY | |
| IGHV3-30 (Human) | IGHJ6 (Human) | ARGSAGNYYYGMDV | |
| IGHV5-51 (Human) | IGHJ4 (Human) | ASALRERGVQLWSV | |
| IGHV3-21 (Human) | IGHJ6 (Human) | ARVNGNSNWNFGSYYYYYMDV | |
| IGHV1-69 (Human) | IGHJ4 (Human) | ARIGHFDSSGYYLDY | |
| IGHV3-21 (Human) | IGHJ6 (Human) | ARWLQLRSDYYYFGMDV | |
| IGHV1-69 (Human) | IGHJ4 (Human) | ARTSHYDSSGSYFEY | |
| IGHV3-30 (Human) | IGHJ6 (Human) | ARPYTGSYKSYMDV | |
| IGHV4-59 (Human) | IGHJ4 (Human) | ARAPRERLQWGEYYFDY | |
| IGHV3-30 (Human) | IGHJ4 (Human) | AKDGSIAAADY | |
| IGHV5-51 (Human) | IGHJ4 (Human) | ATHRCSGGFCYLAY | |
| IGHV3-33 (Human) | IGHJ6 (Human) | ARDQSQGAYILTGYRGYGMDV | |
| IGHV4-34 (Human) | IGHJ6 (Human) | ARPPQAARIHYYYYMDV | |
| IGHV3-9 (Human) | IGHJ6 (Human) | AKGRGAGYTSYMDV | |
| IGHV3-53 (Human) | IGHJ4 (Human) | ARESTQ | |
| IGHV2-5 (Human) | IGHJ4 (Human) | AHRAVILNFDH | |
| IGHV3-30-3 (Human) | IGHJ4 (Human) | ARPRSGSYYAYFDY | |
| IGHV3-9 (Human) | IGHJ4 (Human) | AKVGYTISRQWLVGEFDY | |
| IGHV5-51 (Human) | IGHJ2 (Human) | ARRGEAAGIWYFDL | |
| IGHV4-39 (Human) | IGHJ4 (Human) | ARQSRGYSYAWSFDY | |
| IGHV3-33 (Human) | IGHJ6 (Human) | AGSSGEGGLYYYYGMDV | |
| IGHV3-30 (Human) | IGHJ4 (Human) | ARDSGGNYGDSYFDY | |
| IGHV3-30 (Human) | IGHJ6 (Human) | ARGSAGNYYYGMDV | |
| IGHV2-26 (Human) | IGHJ5 (Human) | ARIVLGASGTYPSPGFDP | |
| IGHV3-74 (Human) | IGHJ4 (Human) | AGSPWLRGDIDY | |
| IGHV7-4-1 (Human) | IGHJ5 (Human) | ARDPSYCSSTRCYTVGWFDP | |
| IGHV4-61 (Human) | IGHJ4 (Human) | ARGHVAAWESCYY | |
| IGHV3-20 (Human) | IGHJ3 (Human) | ARARGPSEQYYDLLTGYYDAFDI | |
| IGHV3-23 (Human) | IGHJ4 (Human) | AQMGPLGSTSSAADY | |
| IGHV1-8 (Human) | IGHJ6 (Human) | AREARYFDWIFEGSDYYYYGMDV | |
| IGHV3-48 (Human) | IGHJ6 (Human) | ARRSYRSSWYYYYGMDV | |
| IGHV3-49 (Human) | IGHJ3 (Human) | IRDYDFWGGYYYHPLRAFDI | |
| IGHV2-5 (Human) | IGHJ3 (Human) | ARHQIVVLFDM | |
| IGHV1-69 (Human) | IGHJ4 (Human) | AITYYYDSSGYWWDD | |
| IGHV4-39 (Human) | IGHJ6 (Human) | AGEEVRGVKLYYYYAMDV | |
| IGHV3-48 (Human) | IGHJ4 (Human) | ARDRGWNYGLDY | |
| IGHV3-30 (Human) | IGHJ4 (Human) | ARDVPTTVTAFTVFTY | |
| IGHV3-30-3 (Human) | IGHJ4 (Human) | ARVPVMVRGVYFDY | |
| IGHV1-8 (Human) | IGHJ6 (Human) | ARGYGLTYYMDV | |
| IGHV1-8 (Human) | IGHJ4 (Human) | ARMRSGWPTHGRPDDF | |
| IGHV3-23 (Human) | IGHJ4 (Human) | AKNERITMLVVVTLFDY | |
| IGHV3-66 (Human) | IGHJ6 (Human) | ARDPSAYYDILTGYSGDV | |
| IGHV3-30-3 (Human) | IGHJ4 (Human) | ARDIDSGYDPTPVFDY | |
| IGHV4-4 (Human) | IGHJ4 (Human) | ASRWGDYFDSSGAYDS | |
| IGHV3-21 (Human) | IGHJ4 (Human) | ARDPVWVDGELLSGGIPFDY | |
| IGHV4-39 (Human) | IGHJ6 (Human) | AILWRGSSWADRHYYYYSMDV | |
| IGHV3-30-3 (Human) | IGHJ5 (Human) | ARDLGRGLDP | |
| IGHV3-23 (Human) | IGHJ3 (Human) | VKGLFDWFPL | |
| IGHV4-61 (Human) | IGHJ6 (Human) | ATGYIGTYYYYMDV | |
| IGHV3-9 (Human) | IGHJ4 (Human) | AKVSSITSLLGYYFDS | |
| IGHV2-70 (Human) | IGHJ4 (Human) | ARGVVTYDY | |
| IGHV3-30 (Human) | IGHJ5 (Human) | ARGDGYRSQFDP | |
| IGHV2-5 (Human) | IGHJ3 (Human) | AHRLWFRDAFDI | |
| IGHV3-23 (Human) | IGHJ6 (Human) | ARVEGDWLLGGPYYHYYGMDV | |
| IGHV4-39 (Human) | IGHJ5 (Human) | ARILVIFTLNWFDP | |
| IGHV4-39 (Human) | IGHJ6 (Human) | ARHDGSGEMDTITWGPIYYYMDV | |
| IGHV2-5 (Human) | IGHJ3 (Human) | AHRLWFRDAFDI | |
| IGHV1-3 (Human) | IGHJ5 (Human) | AMGPSAFSWLDP | |
| IGHV3-30-3 (Human) | IGHJ6 (Human) | ARSTSGSYYYGMDV | |
| IGHV2-5 (Human) | IGHJ4 (Human) | AHRPPSYHGWCYFDY | |
| IGHV1-18 (Human) | IGHJ4 (Human) | ARVQRRRLDY | |
| IGHV4-34 (Human) | IGHJ6 (Human) | ARPPQAARIHYYYYMDV | |
| IGHV5-51 (Human) | IGHJ4 (Human) | ARRFYGPSSFDY | |
| IGHV1-46 (Human) | IGHJ4 (Human) | ARGGEWRIVPGGRDYFDY | |
| IGHV5-51 (Human) | IGHJ5 (Human) | ARLGVSKYCSGGRCLSGGSNWFDP | |
| IGHV3-53 (Human) | IGHJ3 (Human) | ASSSWLRGAFDI | |
| IGHV1-58 (Human) | IGHJ3 (Human) | AAPHCNRTSCYDAFDL | |
| IGHV3-49 (Human) | IGHJ6 (Human) | SRVRGSFYGSVGKNYGMDV | |
| IGHV3-30-3 (Human) | IGHJ4 (Human) | ARDIDSGYDPTPVFDY | |
| IGHV1-69 (Human) | IGHJ4 (Human) | ARTSHYDSSGSYFEY | |
| IGHV5-51 (Human) | IGHJ6 (Human) | ARRRGGIGIEYGMDV | |
| IGHV4-61 (Human) | IGHJ4 (Human) | AGSPVPPTIVGASY | |
| IGHV3-30 (Human) | IGHJ4 (Human) | AKGRGNYLTFFDS | |
| IGHV3-66 (Human) | IGHJ6 (Human) | ARDPGSRYSGGWYDYYYAMDV | |
| IGHV5-51 (Human) | IGHJ4 (Human) | ARRDTDFDY | |
| IGHV1-58 (Human) | IGHJ3 (Human) | AAPYCSRTSCHDAFDI | |
| IGHV3-30 (Human) | IGHJ4 (Human) | AKDLPPYASGWYEGGFDY | |
| IGHV3-30 (Human) | IGHJ4 (Human) | AQGRGGYYSPFDD | |
| IGHV2-26 (Human) | IGHJ2 (Human) | ARTTWGTWIQAWYFDI | |
| IGHV3-33 (Human) | IGHJ5 (Human) | AREGDFWSGYYTGWFDP | |
| IGHV3-30 (Human) | IGHJ4 (Human) | AKDLTIVVIPAAPNFDY | |
| IGHV3-23 (Human) | IGHJ6 (Human) | AKDQARVQDYIWGSYRSYGMDV | |
| IGHV3-9 (Human) | IGHJ6 (Human) | AKDIIRQGEDGMDV | |
| IGHV1-8 (Human) | IGHJ4 (Human) | ARMRSGWPTHGRPDDF | |
| IGHV3-21 (Human) | IGHJ6 (Human) | ARWLQLRSDYYYFGMDV | |
| IGHV3-7 (Human) | IGHJ4 (Human) | ARLSGSSWDFDY | |
| IGHV3-66 (Human) | IGHJ4 (Human) | ARDYRDWI | |
| IGHV2-5 (Human) | IGHJ4 (Human) | AHNRFQYCSSTTCYTLLPFDY | |
| IGHV3-30 (Human) | IGHJ6 (Human) | ARPYTGSYKSYMDV | |
| IGHV3-30 (Human) | IGHJ5 (Human) | ARGDGDVYNFLLVRNWFDP | |
| IGHV4-39 (Human) | IGHJ4 (Human) | ASLWFGDLYSFDY | |
| IGHV3-7 (Human) | IGHJ4 (Human) | ARLGRSSWNFDY | |
| IGHV4-61 (Human) | IGHJ6 (Human) | ARVGGISPYYYYYYMDV | |
| IGHV1-8 (Human) | IGHJ4 (Human) | ARMRSGWPTHGRPDDY | |
| IGHV4-31 (Human) | IGHJ4 (Human) | ASAKLVATISYFDY | |
| IGHV4-34 (Human) | IGHJ6 (Human) | ARVGGYYYYYMDV | |
| IGHV3-23 (Human) | IGHJ6 (Human) | AKPYGMDV | |
| IGHV3-30 (Human) | IGHJ3 (Human) | ARDRSNLERLVMTFGGIIAGAFDI | |
| IGHV3-30 (Human) | IGHJ4 (Human) | ARVNSGSYYSYFDY | |
| IGHV4-4 (Human) | IGHJ4 (Human) | ASRWGDYFDSSGAYDS | |
| IGHV3-33 (Human) | IGHJ4 (Human) | ARKGPLWRFDY | |
| IGHV3-53 (Human) | IGHJ4 (Human) | ARESTQ | |
| IGHV4-39 (Human) | IGHJ6 (Human) | ARAPFQLLDKYYFFYYMDV | |
| IGHV3-53 (Human) | IGHJ4 (Human) | ARHIPAWGYK | |
| IGHV3-23 (Human) | IGHJ3 (Human) | AKLLGSGITLDNDAFDI | |
| IGHV1-69 (Human) | IGHJ4 (Human) | ARIGHFDSSGYYLDY | |
| IGHV3-23 (Human) | IGHJ4 (Human) | AKDFGSGIVGATGFDF | |
| IGHV3-20 (Human) | IGHJ6 (Human) | ARRRSSSRYSSGWYMYYYYMDV | |
| IGHV5-51 (Human) | IGHJ4 (Human) | ATHRCSGGFCYLAY | |
| IGHV3-30 (Human) | IGHJ1 (Human) | ARASYNSNWSIGEYFRD | |
| IGHV3-30 (Human) | IGHJ4 (Human) | AKQGGLYCSGTNCWGGYLDY | |
| IGHV2-5 (Human) | IGHJ4 (Human) | VHRHVSGAFDY | |
| IGHV7-4-1 (Human) | IGHJ3 (Human) | ARARLLGYCSSTSCYTIGWGAFDI | |
| IGHV3-33 (Human) | IGHJ4 (Human) | AREGQGTYLDY | |
| IGHV3-48 (Human) | IGHJ4 (Human) | AREARSRYFDWLPSYYFDY | |
| IGHV3-30 (Human) | IGHJ4 (Human) | ARDPSPLVLITSIDY | |
| IGHV4-39 (Human) | IGHJ5 (Human) | ARRGNYYDSKNWFDP | |
| IGHV4-31 (Human) | IGHJ4 (Human) | ARGGSGSYSLFDY | |
| IGHV3-13 (Human) | IGHJ5 (Human) | ARVLYDSSGFYNWFDP | |
| IGHV4-31 (Human) | IGHJ2 (Human) | ARDLGDGYNLRVPAYFDL | |
| IGHV4-4 (Human) | IGHJ4 (Human) | ARVDHVNVRDY | |
| IGHV3-21 (Human) | IGHJ6 (Human) | ARVNGNSNWNFGSYYYYYMDV | |
| IGHV3-33 (Human) | IGHJ4 (Human) | AREGQMAATTGIDY | |
| IGHV3-15 (Human) | IGHJ3 (Human) | TTLTYYYDSSAYLNDAFDI | |
| IGHV3-9 (Human) | IGHJ4 (Human) | AKGIYYDIFMPLLD | |
| IGHV1-69 (Human) | IGHJ5 (Human) | ARGVVAATPGWFDP | |
| IGHV1-69 (Human) | IGHJ5 (Human) | ARGWFGELLKGTYWFDP | |
| IGHV1-69 (Human) | IGHJ2 (Human) | ARVGVSGFKSGSNWYFDL | |
| IGHV3-74 (Human) | IGHJ4 (Human) | AREVEQLAHMVDY | |
| IGHV1-69 (Human) | IGHJ6 (Human) | AREDYYGSGSLVDPYYYYRMDV | |
| IGHV3-33 (Human) | IGHJ4 (Human) | VRDLALFEVVIQQGV | |
| IGHV4-39 (Human) | IGHJ3 (Human) | ARHTVDCGGDCFPNDAFDI | |
| IGHV1-2 (Human) | IGHJ4 (Human) | ARVVVLGYGRPNNYYDGRNVWDY | |
| IGHV3-30-3 (Human) | IGHJ4 (Human) | ARPRGGSYQTCFDY | |
| IGHV7-4-1 (Human) | IGHJ4 (Human) | ARWGPDYGDYASNDY | |
| IGHV3-20 (Human) | IGHJ3 (Human) | AVIMSPIPRYSGYDWAGDAFDI | |
| IGHV7-4-1 (Human) | IGHJ5 (Human) | ARGLVGRIDP | |
| IGHV1-46 (Human) | IGHJ3 (Human) | ARDQQIVPHADGFDI | |
| IGHV1-69 (Human) | IGHJ1 (Human) | ARIGSYPEYFQH | |
| IGHV3-9 (Human) | IGHJ6 (Human) | AKDIIRQGEDGMDV | |
| IGHV3-7 (Human) | IGHJ6 (Human) | ARDVGGYSGYDLGFDYYYYMDV | |
| IGHV3-30 (Human) | IGHJ3 (Human) | AKDRTAVFLFFGLGDAFDI | |
| IGHV5-51 (Human) | IGHJ4 (Human) | ASALRERGVQLWSV | |
| IGHV3-23 (Human) | IGHJ4 (Human) | AKDPASGIVGPTHFDY | |
| IGHV1-24 (Human) | IGHJ4 (Human) | ATGFAVFGRAAVPY | |
| IGHV4-30-4 (Human) | IGHJ4 (Human) | ARFRRSYGSGSYYNISFDY | |
| IGHV7-4-1 (Human) | IGHJ4 (Human) | VREYGSGHPLPI | |
| IGHV4-59 (Human) | IGHJ3 (Human) | ARATWLRDAFGI | |
| IGHV1-46 (Human) | IGHJ5 (Human) | ARGFHVPAALRNWFDP | |
| IGHV4-31 (Human) | IGHJ4 (Human) | AREANDSGSFYNGPFDY | |
| IGHV4-61 (Human) | IGHJ6 (Human) | ARGGLLWFGGAGNYMDV | |
| IGHV1-8 (Human) | IGHJ4 (Human) | ARMRTGWPTHGRPDDF | |
| IGHV4-34 (Human) | IGHJ5 (Human) | ARGPPVTTFFVFSLLFDP | |
| IGHV3-30 (Human) | IGHJ3 (Human) | AKEGEWELRGNALDI | |
| IGHV3-33 (Human) | IGHJ3 (Human) | AREGQWPNQAFDI | |
| IGHV4-39 (Human) | IGHJ4 (Human) | ASGPPYMATFSYYFDY | |
| IGHV3-11 (Human) | IGHJ4 (Human) | ARDPIRDGVWGLNENDY | |
| IGHV3-30 (Human) | IGHJ4 (Human) | ARSPPASYYNPSTGYFDY | |
| IGHV1-46 (Human) | IGHJ4 (Human) | ARDVFWVPAASSFDY | |
| IGHV3-30-3 (Human) | IGHJ4 (Human) | ARDQEWFRELFLFDY | |
| IGHV1-8 (Human) | IGHJ4 (Human) | ARMRTGWPTHGRPDDF | |
| IGHV4-34 (Human) | IGHJ4 (Human) | ARGWGWGAVAGRAEYYFDY | |
| IGHV3-30-3 (Human) | IGHJ6 (Human) | ARAQGGNYYYGMDV | |
| IGHV4-61 (Human) | IGHJ4 (Human) | ARGAASFDY | |
| IGHV4-39 (Human) | IGHJ5 (Human) | ARDPRVVVTARMYNWFDP | |
| IGHV4-59 (Human) | IGHJ5 (Human) | ARDQRQFQLLGRFGWFDP | |
| IGHV4-39 (Human) | IGHJ2 (Human) | ARQWKWFGEAWYFDL | |
| IGHV1-8 (Human) | IGHJ6 (Human) | ARGGIYYLVRGFIIGYYGMDV | |
| IGHV3-15 (Human) | IGHJ4 (Human) | TTGGYSSYAASDY | |
| IGHV3-48 (Human) | IGHJ2 (Human) | ARVDYYGSGSVYWYFDL | |
| IGHV3-9 (Human) | IGHJ3 (Human) | AKVGWELSIDAFDL | |
| IGHV2-5 (Human) | IGHJ3 (Human) | AHRLWFRDAFDI | |
| IGHV2-70 (Human) | IGHJ6 (Human) | ARIQYQLNGMDV | |
| IGHV1-8 (Human) | IGHJ6 (Human) | ARGRVGYVGSGSRGYYYYYDMDV | |
| IGHV5-51 (Human) | IGHJ2 (Human) | ARPDYSSGWFSYWYFDL | |
| IGHV7-4-1 (Human) | IGHJ4 (Human) | ARGRSYGLSLGY | |
| IGHV3-33 (Human) | IGHJ4 (Human) | ARESADISSRLDY | |
| IGHV2-5 (Human) | IGHJ3 (Human) | AHRLWFRDAFDI | |
| IGHV3-72 (Human) | IGHJ4 (Human) | ASVITFGGVIVRSY | |
| IGHV1-69 (Human) | IGHJ4 (Human) | ARVSGYGDYGAYSDY | |
| IGHV3-20 (Human) | IGHJ4 (Human) | ASVITFGGVIVRSY | |
| IGHV3-23 (Human) | IGHJ6 (Human) | ARVEGDWLLGGPYYHYYGMDV | |
| IGHV3-30-3 (Human) | IGHJ4 (Human) | ARADTMVRGTYFEY | |
| IGHV3-33 (Human) | IGHJ4 (Human) | ARDYCNGVTCNSNY | |
| IGHV4-4 (Human) | IGHJ4 (Human) | ARGWYFDY | |
| IGHV1-69 (Human) | IGHJ6 (Human) | ARVEGEGVDSYYYGMDV | |
| IGHV3-9 (Human) | IGHJ6 (Human) | AKDIIRQGEDGMDV | |
| IGHV4-39 (Human) | IGHJ5 (Human) | ARHPVDGYNYGYSDL | |
| IGHV1-2 (Human) | IGHJ4 (Human) | ARVVVLGYGRPNNYYDGRNVWDY | |
| IGHV3-30 (Human) | IGHJ4 (Human) | ARAGGGSYRGPFDY | |
| IGHV3-30 (Human) | IGHJ3 (Human) | AKSYNGNYYDAFDI | |
| IGHV3-30 (Human) | IGHJ6 (Human) | ARGSAGNYYYGMDV | |
| IGHV5-51 (Human) | IGHJ6 (Human) | ARLTFGGSGSYYFYYNGMDV | |
| IGHV3-30 (Human) | IGHJ4 (Human) | AKGGDGSGWAWDGDNPPTDY | |
| IGHV2-26 (Human) | IGHJ5 (Human) | ARIVLGASGTYPSPGFDP | |
| IGHV3-30 (Human) | IGHJ5 (Human) | AKDGSGSYYGWFDP | |
| IGHV4-39 (Human) | IGHJ4 (Human) | ARLLWLRGHFDY | |
| IGHV3-20 (Human) | IGHJ3 (Human) | AVIMSPIPRYSGYDWAGGAFDI | |
| IGHV3-30 (Human) | IGHJ5 (Human) | ARALNKGFDP | |
| IGHV1-58 (Human) | IGHJ3 (Human) | AAPHCNRTSCYDAFDL | |
| IGHV1-46 (Human) | IGHJ6 (Human) | ARDRLGDGSYLGGGYYGMDV | |
| IGHV1-8 (Human) | IGHJ5 (Human) | ARMRSGWPTHGRPDDL | |
| IGHV1-69 (Human) | IGHJ4 (Human) | ARDHSGYYDSTSLMSPFFDY | |
| IGHV1-46 (Human) | IGHJ5 (Human) | ARDLAGVPAALGCWFDP | |
| IGHV3-30-3 (Human) | IGHJ4 (Human) | AKNLGPYCSGGTCYSLVGDY | |
| IGHV3-13 (Human) | IGHJ4 (Human) | ARARGGYNWNFDY | |
| IGHV3-30 (Human) | IGHJ4 (Human) | ARDQGTVVTHFDY | |
| IGHV3-30 (Human) | IGHJ4 (Human) | AKNLGPYCSGGTCYSLVGDY | |
| IGHV1-8 (Human) | IGHJ4 (Human) | ARMRSGWPTHGRPDDH | |
| IGHV3-43 (Human) | IGHJ6 (Human) | AKDEMAYPPSHHYYYYYMDV | |
| IGHV1-8 (Human) | IGHJ4 (Human) | ARGPSILTGFYNPLDY | |
| IGHV4-39 (Human) | IGHJ6 (Human) | ARRTYYDLWSAYSSTAYYCMDV | |
| IGHV1-69 (Human) | IGHJ6 (Human) | ARLSGSGWLGYAMDV | |
| IGHV3-30 (Human) | IGHJ2 (Human) | ARPSNWYFDL | |
| IGHV3-9 (Human) | IGHJ4 (Human) | AKAGYYAYVWGSYRFEYFDN | |
| IGHV3-53 (Human) | IGHJ3 (Human) | ARGPEPDAFDI | |
| IGHV4-61 (Human) | IGHJ4 (Human) | AGSPVPPTIVGASY | |
| IGHV3-30-3 (Human) | IGHJ3 (Human) | ARDLSTTWYLEMWGPDAFDI | |
| IGHV3-30 (Human) | IGHJ4 (Human) | AKKGGPYCGGGNCYAGYFDY | |
| IGHV4-34 (Human) | IGHJ6 (Human) | ARVGYSQGYYYYYMDV | |
| IGHV3-9 (Human) | IGHJ6 (Human) | ARDYCSSTTCPAETYYYMDV | |
| IGHV3-53 (Human) | IGHJ5 (Human) | ARDFLRWHDL | |
| IGHV3-13 (Human) | IGHJ4 (Human) | ARVDFDILTGYYSN | |
| IGHV3-23 (Human) | IGHJ4 (Human) | AKWAGPIVMKYYLQY | |
| IGHV3-13 (Human) | IGHJ2 (Human) | ARGGDSGYDLGAWYFDL | |
| IGHV5-51 (Human) | IGHJ5 (Human) | ARTPTLYNWFHP | |
| IGHV2-70 (Human) | IGHJ6 (Human) | ARATTFFYGMDV | |
| IGHV4-39 (Human) | IGHJ4 (Human) | ARHQRYCSSSSCHVWDY | |
| IGHV3-9 (Human) | IGHJ6 (Human) | AKGRGAGYTSYMDV | |
| IGHV3-53 (Human) | IGHJ3 (Human) | ARSYDILTGYRDAFDI | |
| IGHV3-30-3 (Human) | IGHJ6 (Human) | ARDDNSPQGSGWYFYYYYAMDV | |
| IGHV3-30-3 (Human) | IGHJ3 (Human) | ARDWAPTYYDMPSAFDI | |
| IGHV3-7 (Human) | IGHJ4 (Human) | VRLGVSSWYFDY | |
| IGHV3-7 (Human) | IGHJ6 (Human) | ARVVVEVATNKGIHGVDYYYYYYMDV | |
| IGHV4-34 (Human) | IGHJ4 (Human) | ARLRYSSSGGHIFDY | |
| IGHV1-69 (Human) | IGHJ4 (Human) | ARGLTGSSAYKDEIYFDY | |
| IGHV4-59 (Human) | IGHJ6 (Human) | ARDGGNAYSSGWYRYYYHMDV | |
| IGHV3-13 (Human) | IGHJ5 (Human) | ARVLYDSSGFYNWFDP | |
| IGHV1-46 (Human) | IGHJ5 (Human) | ARGFHVPAALRNWFDP | |
| IGHV3-53 (Human) | IGHJ3 (Human) | ARSYDILTGYRDAFDI | |
| IGHV3-7 (Human) | IGHJ4 (Human) | ARVNDGRPNPLEYYFDY | |
| IGHV4-31 (Human) | IGHJ4 (Human) | ARETYSAYEMPPYFDY | |
| IGHV5-51 (Human) | IGHJ6 (Human) | ARDLIIESTIAARPGYYGMDV | |
| IGHV1-46 (Human) | IGHJ4 (Human) | ARERSGTYFFDY | |
| IGHV2-70 (Human) | IGHJ4 (Human) | ARETPVTAIDY | |
| IGHV1-58 (Human) | IGHJ3 (Human) | AAPYCSSISCNDGFDI | |
| IGHV1-3 (Human) | IGHJ6 (Human) | ASMTRMSEQTYYGMDV | |
| IGHV3-30 (Human) | IGHJ4 (Human) | ARELMSVG | |
| IGHV3-53 (Human) | IGHJ6 (Human) | ARDALYYNGPGRDGMDV | |
| IGHV3-53 (Human) | IGHJ6 (Human) | ARDALYYNGPGRDGMDV | |
| IGHV3-30 (Human) | IGHJ6 (Human) | AKDMVEPLFSHYYYYGMDV | |
| IGHV4-59 (Human) | IGHJ4 (Human) | ARAPRERLQWGEYYFDY | |
| IGHV3-13 (Human) | IGHJ6 (Human) | ARVFETKVIRGGRYYYYYYMDV | |
| IGHV4-34 (Human) | IGHJ6 (Human) | ARCRQMGNFYYYYMDV | |
| IGHV4-31 (Human) | IGHJ5 (Human) | ARVVPTRGPVAWFDP | |
| IGHV3-33 (Human) | IGHJ6 (Human) | ARDQSQGAYILTGYRGYGMDV | |
| IGHV1-69 (Human) | IGHJ4 (Human) | TTTQGGDYGDNLYYLDY | |
| IGHV3-53 (Human) | IGHJ3 (Human) | ARGPEPDAFDI | |
| IGHV1-46 (Human) | IGHJ4 (Human) | ARGYGFVPNVLYYFDY | |
| IGHV3-21 (Human) | IGHJ4 (Human) | ARGGSILWWLIDY | |
| IGHV3-23 (Human) | IGHJ3 (Human) | AKDSRSGIAGVDAFDI | |
| IGHV3-66 (Human) | IGHJ4 (Human) | ARGDGGYYSPFDY | |
| IGHV2-5 (Human) | IGHJ5 (Human) | AHRLPTPQLLPSFENWFDP | |
| IGHV3-66 (Human) | IGHJ4 (Human) | AREVVGYFDC | |
| IGHV4-59 (Human) | IGHJ4 (Human) | ARLRWLRGGIDF | |
| IGHV1-46 (Human) | IGHJ4 (Human) | ARGYGFVPNVLYYFDY | |
| IGHV3-30-3 (Human) | IGHJ6 (Human) | ARAQGGNYYYGMDV | |
| IGHV3-30 (Human) | IGHJ6 (Human) | ARDLAYHPYRDYGDDDYYYYYGMDV | |
| IGHV1-46 (Human) | IGHJ4 (Human) | ARGAIPPNSRAEIDY | |
| IGHV1-46 (Human) | IGHJ6 (Human) | ARENDYGDYVEPRDYYYGMDV | |
| IGHV3-33 (Human) | IGHJ3 (Human) | AREGPFGDREASGAFDV | |
| IGHV3-11 (Human) | IGHJ1 (Human) | TGVVAAPAEYFQH | |
| IGHV3-7 (Human) | IGHJ4 (Human) | ARLGFYYGGADY | |
| IGHV3-30 (Human) | IGHJ4 (Human) | AKDGSIAAADY | |
| IGHV1-46 (Human) | IGHJ4 (Human) | ARGAAVPAAGEFDY | |
| IGHV3-30 (Human) | IGHJ4 (Human) | AKGDGSYLMDYFDY | |
| IGHV3-53 (Human) | IGHJ3 (Human) | ARSYDILTGYRDAFDI | |
| IGHV4-34 (Human) | IGHJ6 (Human) | ARCRQMGNFYYYYMDV | |
| IGHV5-51 (Human) | IGHJ6 (Human) | ARLGSESKIDYYYYGMDV | |
| IGHV1-69 (Human) | IGHJ6 (Human) | ARGRGYSNYGASYYMDV | |
| IGHV4-39 (Human) | IGHJ3 (Human) | ARMSRGYNYAYTFDI | |
| IGHV2-70 (Human) | IGHJ3 (Human) | ARTMATINAFDI | |
| IGHV3-30-3 (Human) | IGHJ4 (Human) | ARGGATNFDY | |
| IGHV7-4-1 (Human) | IGHJ4 (Human) | ARPGKAAAFDY | |
| IGHV3-33 (Human) | IGHJ6 (Human) | ARDLHQDWVVVVAANVYGMDV | |
| IGHV1-58 (Human) | IGHJ3 (Human) | AAPYCSSISCNDGFDI | |
| IGHV2-70 (Human) | IGHJ6 (Human) | ARATTFFYGMDV | |
| IGHV3-30 (Human) | IGHJ4 (Human) | AKKGGPYCGGGNCYAGYFDY | |
| IGHV3-9 (Human) | IGHJ3 (Human) | AKLDVGGYDFVSGHYYAFDI | |
| IGHV7-4-1 (Human) | IGHJ6 (Human) | ARGLISLFRGAIFHYYYGMDV | |
| IGHV3-66 (Human) | IGHJ6 (Human) | ARDLVTYGLDV | |
| IGHV2-5 (Human) | IGHJ5 (Human) | AHSGPPDLSPVLSQGWFDP | |
| IGHV3-30 (Human) | IGHJ6 (Human) | AKGGPNKEVLYFGELLDYGMDV | |
| IGHV1-18 (Human) | IGHJ4 (Human) | ARVQRRRLDY | |
| IGHV1-58 (Human) | IGHJ3 (Human) | AAPYCSSISCNDGFDI | |
| IGHV1-46 (Human) | IGHJ4 (Human) | ARGAIPPNSRAEIDY | |
| IGHV3-30-3 (Human) | IGHJ3 (Human) | ARDLSTTWYLEMWGPDAFDI | |
| IGHV3-13 (Human) | IGHJ5 (Human) | ARVLYDSSGFYNWFDP | |
| IGHV3-30 (Human) | IGHJ4 (Human) | AKNLGPYCSGGTCYSLVGDY | |
| IGHV1-58 (Human) | IGHJ3 (Human) | AAPYCSSISCNDGFDI | |
| IGHV3-43 (Human) | IGHJ6 (Human) | AKDEMAYPPSHHYYYYYMDV | |
| IGHV3-7 (Human) | IGHJ6 (Human) | ARLVTTVTTANGLYYYSYYYMDV | |
| IGHV3-30 (Human) | IGHJ3 (Human) | ARDRSGNYRDAFDI | |
| IGHV3-53 (Human) | IGHJ4 (Human) | AREGEVEGYNDFWSGYSRDRYYFDY | |
| IGHV3-30 (Human) | IGHJ4 (Human) | ASSSGYLFHSDY | |
| IGHV3-13 (Human) | IGHJ4 (Human) | ARGDSSGYYYYFDY | |
| IGHV1-18 (Human) | IGHJ4 (Human) | ARDYTRGAWFGESLIGGFDN | |
| IGHV2-70 (Human) | IGHJ6 (Human) | ARIPGFLRYRNRYYYYGMDV | |
| IGHV3-15 (Human) | IGHJ4 (Human) | TTGWFTGTYGDYFDY | |
| IGHV3-49 (Human) | IGHJ4 (Human) | TRVKYCSGGSCYGYHFDH | |
| IGHV4-34 (Human) | IGHJ6 (Human) | ARVVIAVAGTYPIQVYYYYGMDV | |
| IGHV3-66 (Human) | IGHJ3 (Human) | ARDFREGAFDI | |
| IGHV3-21 (Human) | IGHJ4 (Human) | ARAGFVPKRAYCGGDCWYYFDY | |
| IGHV4-4 (Human) | IGHJ4 (Human) | ATDGGWYTFDH | |
| IGHV4-31 (Human) | IGHJ3 (Human) | ARYPVWGAFDI | |
| IGHV3-30-3 (Human) | IGHJ3 (Human) | ARARGGSYSGAFDI | |
| IGHV3-30-3 (Human) | IGHJ4 (Human) | ARVLWLRGMFDY | |
| IGHV3-30-3 (Human) | IGHJ4 (Human) | ARGDYYGSGSYPGKTFDY | |
| IGHV1-69 (Human) | IGHJ5 (Human) | ARGRLDSYSGSYYSWFDP | |
| IGHV3-23 (Human) | IGHJ4 (Human) | AKNEMAMIVVVITLFDY | |
| IGHV4-4 (Human) | IGHJ4 (Human) | ATKYCSGGSCSYFGY | |
| IGHV3-7 (Human) | IGHJ4 (Human) | ARDQGVTTGPFDY | |
| IGHV3-30 (Human) | IGHJ4 (Human) | AKQGGGTYCGGGSCYRGYFDY | |
| IGHV1-46 (Human) | IGHJ3 (Human) | ARDPRVPAVTNVNDAFDL | |
| IGHV3-53 (Human) | IGHJ4 (Human) | ARSHLEVRGVFDN | |
| IGHV3-20 (Human) | IGHJ4 (Human) | ARGTGAADY | |
| IGHV3-33 (Human) | IGHJ4 (Human) | AREGVYGDIGGAGLDY | |
| IGHV3-30 (Human) | IGHJ4 (Human) | AKMGGVYCSAGNCYSGRLEY | |
| IGHV5-51 (Human) | IGHJ4 (Human) | ARREWGGSLGHIDY | |
| IGHV4-59 (Human) | IGHJ6 (Human) | ARGQGVPAALYGMDV | |
| IGHV3-53 (Human) | IGHJ6 (Human) | ARDLQLYGMDV | |
| IGHV1-18 (Human) | IGHJ6 (Human) | ARVPGLVGYSSSWYDNEKNYYYYYYGMDV | |
| IGHV3-23 (Human) | IGHJ5 (Human) | AKADTAMAWYNWFDP | |
| IGHV4-34 (Human) | IGHJ2 (Human) | ARVWVRWWYFDL | |
| IGHV1-46 (Human) | IGHJ4 (Human) | ASSLPARGGVPGRLNY | |
| IGHV5-51 (Human) | IGHJ5 (Human) | ARHHLDYDDYVGHWFDP | |
| IGHV1-58 (Human) | IGHJ3 (Human) | AAAYCSGGSCSDGFDI | |
| IGHV1-58 (Human) | IGHJ6 (Human) | AAPNCSNVVCYDGFDI | |
| IGHV1-18 (Human) | IGHJ6 (Human) | ARPDYQVLGYDFWIGYYGMDV | |
| IGHV5-51 (Human) | IGHJ4 (Human) | ARQWSHYTYDYYY | |
| IGHV1-58 (Human) | IGHJ3 (Human) | ASPYCSGGSCSDGFDI | |
| IGHV3-30 (Human) | IGHJ4 (Human) | ANLKDSRYSGSYYDY | |
| IGHV3-53 (Human) | IGHJ6 (Human) | ARDRDYYGMDV | |
| IGHV3-53 (Human) | IGHJ4 (Human) | ARALQVGATSDYFDY | |
| IGHV3-53 (Human) | IGHJ6 (Human) | ARDLQEHGMDV | |
| IGHV1-69 (Human) | IGHJ5 (Human) | ARDSRYCSGGSCYSVWFDP | |
| IGHV1-2 (Human) | IGHJ6 (Human) | ARDRSWAVVYYYMDV | |
| IGHV3-30-3 (Human) | IGHJ4 (Human) | AKDGGKLWVYYFDY | |
| IGHV3-53 (Human) | IGHJ6 (Human) | ARDLERAGGMDV | |
| IGHV3-53 (Human) | IGHJ3 (Human) | ARAHVDTAMVESGAFDI | |
| IGHV3-53 (Human) | IGHJ4 (Human) | ARGEGWELPYDY | |
| IGHV3-53 (Human) | IGHJ6 (Human) | ARDLDVSGGMDV | |
| IGHV1-24 (Human) | IGHJ6 (Human) | ATSTAVAGTPDLFDYYYGMDV | |
| IGHV1-69 (Human) | IGHJ4 (Human) | ARVSGYDSSGYWGDY | |
| IGHV1-18 (Human) | IGHJ5 (Human) | ARDGELLGWFDP | |
| IGHV3-49 (Human) | IGHJ6 (Human) | TRVRRLWFGSYYYGMDV | |
| IGHV3-49 (Human) | IGHJ6 (Human) | TRVRRLWFGSYYYGMDV | |
| IGHV1-2 (Human) | IGHJ4 (Human) | ARASVSTITDFDY | |
| IGHV1-2 (Human) | IGHJ4 (Human) | ARASVATITDFDY | |
| IGHV1-8 (Human) | IGHJ4 (Human) | ARATTDCSSTSCWSLDFWSGYYTGGREKIFD | |
| IGHV7-4-1 (Human) | IGHJ5 (Human) | ARSLRGANLVP | |
| IGHV7-4-1 (Human) | IGHJ5 (Human) | ARSLRGANLVP | |
| IGHV3-33 (Human) | IGHJ6 (Human) | ARAARRPVVTDTMAYYMDV | |
| IGHV1-58 (Human) | IGHJ3 (Human) | AAPHCSSTICYDGFDI | |
| IGHV3-30 (Human) | IGHJ5 (Human) | AKGGDYEWELLES | |
| IGHV3-66 (Human) | IGHJ4 (Human) | ARDFGDFFFDY | |
| IGHV3-66 (Human) | IGHJ4 (Human) | ARDYGDYFFDY | |
| IGHV1-58 (Human) | IGHJ3 (Human) | AAPYCSSTRCYDAFDI | |
| IGHV3-9 (Human) | IGHJ4 (Human) | AKDINYDSGGYHKNYFDY | |
| IGHV3-23 (Human) | IGHJ4 (Human) | ALASGSYFGGANY | |
| IGHV3-66 (Human) | IGHJ4 (Human) | ATGARFGESPFDY | |
| IGHV3-66 (Human) | IGHJ4 (Human) | ATGARFGESPFDY | |
| IGHV1-69 (Human) | IGHJ5 (Human) | AREGGLDYFGSRNSGWTYTWFDP | |
| IGHV4-39 (Human) | IGHJ3 (Human) | ARGVNYYDRNGYYRNDGFDI | |
| IGHV3-48 (Human) | IGHJ4 (Human) | ASSKGFCSGGSCSDY | |
| IGHV3-9 (Human) | IGHJ2 (Human) | AKDLRRQDYYADWYFDL | |
| IGHV4-39 (Human) | IGHJ3 (Human) | ARGVNYYDRNGYYRNDGFDI | |
| IGHV3-9 (Human) | IGHJ2 (Human) | AKDLRRQDYYADWYFDL | |
| IGHV4-39 (Human) | IGHJ3 (Human) | ARGVNYYDRNGYYRNDGFDI | |
| IGHV7-4-1 (Human) | IGHJ4 (Human) | AKIGSRNSLGV | |
| IGHV1-58 (Human) | IGHJ3 (Human) | AAPRCSGGSCYDGFDI | |
| IGHV3-21 (Human) | IGHJ4 (Human) | ASNRSPYDSSNYYFDY | |
| IGHV1-69 (Human) | IGHJ6 (Human) | ARDDWTTALDC | |
| IGHV1-69 (Human) | IGHJ6 (Human) | ARDRPPIVVVTTYYYYGMDV | |
| IGHV3-30 (Human) | IGHJ6 (Human) | AKAFKGNYYYGMDV | |
| IGHV1-18 (Human) | IGHJ4 (Human) | ALVGATDY | |
| IGHV1-2 (Human) | IGHJ4 (Human) | ARGAASVLRFLEWLLDY | |
| IGHV3-13 (Human) | IGHJ4 (Human) | ARAQYSSGWYLRFDY | |
| IGHV4-4 (Human) | IGHJ4 (Human) | ASRYCSGGSCGYFDY | |
| IGHV3-74 (Human) | IGHJ4 (Human) | ARDLHYDSSGWDY | |
| IGHV3-66 (Human) | IGHJ3 (Human) | ARDFREGAFDI | |
| IGHV3-30 (Human) | IGHJ4 (Human) | AKDPTIVVVAVNPTFDY | |
| IGHV3-21 (Human) | IGHJ6 (Human) | ARDLDIVVVPAARSYYYYGMDV | |
| IGHV3-48 (Human) | IGHJ6 (Human) | ARDRCGDCYGPYYYGMDV | |
| IGHV1-69 (Human) | IGHJ6 (Human) | ASHPYYDSSGYYPNYGMDA | |
| IGHV3-33 (Human) | IGHJ4 (Human) | ARDGQVGATSGIDY | |
| IGHV3-30-3 (Human) | IGHJ6 (Human) | AKSAFGSYYYGMDV | |
| IGHV3-30 (Human) | IGHJ4 (Human) | AKGGSGYRYYFDY | |
| IGHV1-24 (Human) | IGHJ5 (Human) | ATGPLTIAVAGQWFDP | |
| IGHV3-30 (Human) | IGHJ3 (Human) | ARPDSGSYWGAFDI | |
| IGHV1-18 (Human) | IGHJ3 (Human) | ARDTRGRGGHDAFDI | |
| IGHV3-15 (Human) | IGHJ4 (Human) | TTASNPDY | |
| IGHV4-59 (Human) | IGHJ5 (Human) | ARENYDFWSGYFNGWFDP | |
| IGHV3-30 (Human) | IGHJ4 (Human) | AKSHSGSYFSSGDY | |
| IGHV5-51 (Human) | IGHJ6 (Human) | ARRTLTTTYGMDV | |
| IGHV3-30 (Human) | IGHJ4 (Human) | AKGWGGYNTHFDY | |
| IGHV3-49 (Human) | IGHJ4 (Human) | SRPLTYYYDSSGYYYPYYFDY | |
| IGHV3-9 (Human) | IGHJ6 (Human) | AKGNWNDVLSHYYYYYGMDV | |
| IGHV4-34 (Human) | IGHJ4 (Human) | ARGGTIGTTGIYDILTGYDPFFDY | |
| IGHV3-7 (Human) | IGHJ6 (Human) | ARDSVHYYYDSSGYHYSYGMDV | |
| IGHV3-30 (Human) | IGHJ4 (Human) | ARPNSGSYSSYLDY | |
| IGHV3-30-3 (Human) | IGHJ4 (Human) | AKGRSGSYFNPLDY | |
| IGHV3-48 (Human) | IGHJ6 (Human) | ARDSVPRYYYHYYGMDV | |
| IGHV4-4 (Human) | IGHJ4 (Human) | ARRRLGPSSNNSGYFY | |
| IGHV3-7 (Human) | IGHJ2 (Human) | ARDYTDDYGDYGGYFDL | |
| IGHV1-46 (Human) | IGHJ4 (Human) | ARGRYCSSSSCYIGLDY | |
| IGHV3-15 (Human) | IGHJ4 (Human) | ATEEHPFAGRAKLHHLDY | |
| IGHV3-9 (Human) | IGHJ4 (Human) | AKDLTGWGLTFGGVITN | |
| IGHV4-59 (Human) | IGHJ2 (Human) | ARGYDF | |
| IGHV3-66 (Human) | IGHJ4 (Human) | ARSYGDYYFDY | |
| IGHV3-30 (Human) | IGHJ4 (Human) | AKMMGQYCSGGDCYSGYFDY | |
| IGHV3-11 (Human) | IGHJ4 (Human) | ARDVAYYYDSGSYYYFDY | |
| IGHV3-30 (Human) | IGHJ4 (Human) | AKMVGQYCSGGNCYLGYFDY | |
| IGHV3-30-3 (Human) | IGHJ4 (Human) | VKGLGGNYYYFGF | |
| IGHV1-24 (Human) | IGHJ6 (Human) | ATSEVVVPGAIRHKAAYYYNYMDV | |
| IGHV3-30 (Human) | IGHJ4 (Human) | AKKGYSYGYFDYYFDY | |
| IGHV1-69 (Human) | IGHJ5 (Human) | ARDQDSGYIWWFDP | |
| IGHV3-21 (Human) | IGHJ5 (Human) | ARDPFSRWERPEGWFDP | |
| IGHV3-9 (Human) | IGHJ4 (Human) | AKGGRRAAMLLNYFDY | |
| IGHV3-66 (Human) | IGHJ6 (Human) | ARESYGMDV | |
| IGHV3-30 (Human) | IGHJ4 (Human) | AKKGYSYGYFDYYFDY | |
| IGHV3-9 (Human) | IGHJ4 (Human) | AKDIKSFGIFGVVTAFDY | |
| IGHV3-53 (Human) | IGHJ4 (Human) | ARGPYPHFDY | |
| IGHV4-61 (Human) | IGHJ5 (Human) | ARGPDIVVVPAADPRNWFDP | |
| IGHV4-59 (Human) | IGHJ4 (Human) | TRQLVLVRGYFDY | |
| IGHV3-9 (Human) | IGHJ6 (Human) | AKDIGPFEAARPGGNYYYYAMDV | |
| IGHV3-53 (Human) | IGHJ3 (Human) | ARNIYDDAFDV | |
| IGHV3-30 (Human) | IGHJ6 (Human) | TKADYYDFWSGYQKTYYYYMDV | |
| IGHV1-18 (Human) | IGHJ4 (Human) | ARAHPFYDSGGYSDY | |
| IGHV3-21 (Human) | IGHJ6 (Human) | ASGEHNYYYYYGMDV | |
| IGHV3-30 (Human) | IGHJ4 (Human) | AKDAADSRITMFGVVIISHFDY | |
| IGHV3-66 (Human) | IGHJ4 (Human) | ARELVGYFDY | |
| IGHV3-66 (Human) | IGHJ4 (Human) | ASTPRGDSYGGGAY | |
| IGHV3-30 (Human) | IGHJ4 (Human) | AKEGWGYSYGSYYFDY | |
| IGHV4-39 (Human) | IGHJ5 (Human) | ARHPDICDFWSGYPGWFDP | |
| IGHV1-69 (Human) | IGHJ4 (Human) | ARESPYCSSTTCLSDY | |
| IGHV1-18 (Human) | IGHJ4 (Human) | ARDDDNYDTTGYYLY | |
| IGHV3-13 (Human) | IGHJ3 (Human) | ARAHRGYYDRSGYYHNPDAFDI | |
| IGHV3-21 (Human) | IGHJ3 (Human) | ARMAYFDSSGYYPNAFDI | |
| IGHV1-24 (Human) | IGHJ4 (Human) | ATDRARLDYFASGSYYGHFDY | |
| IGHV3-74 (Human) | IGHJ6 (Human) | ARDHVVAATPGMDV | |
| IGHV1-58 (Human) | IGHJ3 (Human) | AAPNCSRTSCQDGFDI | |
| IGHV1-18 (Human) | IGHJ4 (Human) | ARALENYYDRNGNYYVGAFDY | |
| IGHV3-66 (Human) | IGHJ4 (Human) | ARDRFGRINDY | |
| IGHV3-30 (Human) | IGHJ4 (Human) | AREGADRSGWWGSFDY | |
| IGHV1-58 (Human) | IGHJ3 (Human) | AAPYCTTTRCHDGFDI | |
| IGHV4-31 (Human) | IGHJ4 (Human) | ARDSAAGHFDY | |
| IGHV3-30 (Human) | IGHJ4 (Human) | AKPYSGSYWSYFDY | |
| IGHV3-53 (Human) | IGHJ5 (Human) | ARERGAKAFDP | |
| IGHV3-30-3 (Human) | IGHJ3 (Human) | AKSRGGNYFDAFDM | |
| IGHV4-38-2 (Human) | IGHJ4 (Human) | ARLPYGYDYVEAFDI | |
| IGHV1-8 (Human) | IGHJ5 (Human) | ARSKERGYYNRTGYYYPGDWFDP | |
| IGHV3-30-3 (Human) | IGHJ4 (Human) | AKDLSGGYSYWDY | |
| IGHV4-39 (Human) | IGHJ4 (Human) | ARLGSGSYYTADY | |
| IGHV3-23 (Human) | IGHJ4 (Human) | AKSMFGYDSSGYFYGEDFDY | |
| IGHV3-30 (Human) | IGHJ4 (Human) | AKDGGYYYESSGWFDY | |
| IGHV3-30 (Human) | IGHJ6 (Human) | AKDRGTLIEGMDV | |
| IGHV3-66 (Human) | IGHJ4 (Human) | ARGFGDRRLDY | |
| IGHV3-11 (Human) | IGHJ4 (Human) | TRDGVIPPRFDY | |
| IGHV1-2 (Human) | IGHJ3 (Human) | ASLSAAGPLNDVFDI | |
| IGHV1-2 (Human) | IGHJ1 (Human) | ARVARHYYDRSGNLHSADYFQH | |
| IGHV4-34 (Human) | IGHJ4 (Human) | AREMSVAVVDH | |
| IGHV1-2 (Human) | IGHJ6 (Human) | ARYSNYYYYYGMDV | |
| IGHV5-51 (Human) | IGHJ4 (Human) | ARLGSWYVYYYYYALDV | |
| IGHV3-48 (Human) | IGHJ4 (Human) | ARTIYSYDSSGYYGTERYFDY | |
| IGHV3-9 (Human) | IGHJ4 (Human) | AKGTGYSYGYAVDGGFDY | |
| IGHV3-21 (Human) | IGHJ4 (Human) | ARDFHRGWYDHSAYIFDF | |
| IGHV3-33 (Human) | IGHJ3 (Human) | ARDDPTPDGDAFDI | |
| IGHV1-24 (Human) | IGHJ6 (Human) | ATGVAVAGTQKNYSYYYGLDV | |
| IGHV4-30-2 (Human) | IGHJ3 (Human) | ARHSGYDLGGAFDI | |
| IGHV3-9 (Human) | IGHJ6 (Human) | AKDDSSSWYFYSRAKLGQYYYYGMDV | |
| IGHV4-30-4 (Human) | IGHJ4 (Human) | ARQLWLRAPFDY | |
| IGHV1-18 (Human) | IGHJ4 (Human) | AREGSDYYDSSGFHDY | |
| IGHV1-69 (Human) | IGHJ6 (Human) | ARESTTIFGVVILTSYGMDV | |
| IGHV3-30 (Human) | IGHJ6 (Human) | ARTRGGSYYYGMDV | |
| IGHV4-39 (Human) | IGHJ4 (Human) | ARHVQWLVLYYFDY | |
| IGHV3-13 (Human) | IGHJ4 (Human) | ARASFDSSGYLNYFDY | |
| IGHV1-18 (Human) | IGHJ4 (Human) | ARDGGILTGYLDYFDH | |
| IGHV4-39 (Human) | IGHJ4 (Human) | ARCRPEYYFGSGSYLDFDY | |
| IGHV1-24 (Human) | IGHJ5 (Human) | ATTTPFSSSYWFDP | |
| IGHV4-59 (Human) | IGHJ4 (Human) | ARHSRIEVAGTLDFDY | |
| IGHV5-10-1 (Human) | IGHJ6 (Human) | ARLSVRVWFGELPHYGMDV | |
| IGHV1-58 (Human) | IGHJ3 (Human) | AAPHCNSTSCYDAFDI | |
| IGHV3-30-3 (Human) | IGHJ6 (Human) | AKGGSYAYYYYMDV | |
| IGHV3-30 (Human) | IGHJ6 (Human) | ARDVVVVVAARNHYYNGMDV | |
| IGHV3-53 (Human) | IGHJ5 (Human) | ARGEGSPGNWFDP | |
| IGHV1-69 (Human) | IGHJ3 (Human) | ARDLLDPQLDDAFDI | |
| IGHV3-9 (Human) | IGHJ4 (Human) | VKGVEYSSSSNFDY | |
| IGHV3-66 (Human) | IGHJ4 (Human) | ARDTLGRGGDY | |
| IGHV3-23 (Human) | IGHJ5 (Human) | ARESDCGSTSCYQVGWFDP | |
| IGHV1-46 (Human) | IGHJ4 (Human) | ARGPERGIVGATDYFDY | |
| IGHV3-23 (Human) | IGHJ4 (Human) | AKEPIGQPLLWWDY | |
| IGHV5-51 (Human) | IGHJ5 (Human) | ARGPNLQNWFDP | |
| IGHV3-53 (Human) | IGHJ6 (Human) | ARDLMAYGMDV | |
| IGHV3-66 (Human) | IGHJ4 (Human) | ARDYGDFYFDF | |
| IGHV1-2 (Human) | IGHJ6 (Human) | ARERYFDLGGMDV | |
| IGHV3-33 (Human) | IGHJ5 (Human) | ARDVGRVTTWFDP | |
| IGHV3-23 (Human) | IGHJ5 (Human) | ASEEDYSNYVGWFDP | |
| IGHV3-13 (Human) | IGHJ2 (Human) | ARDRGSSGWYGWYFDL | |
| IGHV3-53 (Human) | IGHJ4 (Human) | ARDYGDFYFDY | |
| IGHV1-2 (Human) | IGHJ6 (Human) | ASPASRGYSGYDHGYYYYMDV | |
| IGHV1-58 (Human) | IGHJ3 (Human) | AAPHCSGGSCLDAFDI | |
| IGHV3-11 (Human) | IGHJ6 (Human) | ARRGDGSSSIYYYNYMDV | |
| IGHV3-30 (Human) | IGHJ4 (Human) | AREFGDPEWYFDY | |
| IGHV1-2 (Human) | IGHJ4 (Human) | ARDSPFSALGASNDY | |
| IGHV3-30 (Human) | IGHJ4 (Human) | ARDGIVDTALVTWFDY | |
| IGHV1-69 (Human) | IGHJ6 (Human) | ARGNRLLYCSSTSCYLDAVRQGYYYYYYMDV | |
| IGHV3-30 (Human) | IGHJ4 (Human) | AKVTAPYCSGGSCYGGNFDY | |
| IGHV3-9 (Human) | IGHJ4 (Human) | AKAGVRGIAAAGPDLNFDH | |
| IGHV3-30 (Human) | IGHJ4 (Human) | ARDFDDSSFWAFDY | |
| IGHV1-46 (Human) | IGHJ5 (Human) | ARVPREGTPGFDP | |
| IGHV4-31 (Human) | IGHJ4 (Human) | ARVWQYYDSSGSFDY | |
| IGHV3-30 (Human) | IGHJ4 (Human) | AKASGIYCSGGDCYSYYFDY | |
| IGHV4-31 (Human) | IGHJ4 (Human) | ARTMYYYDSSGSFDY | |
| IGHV3-30 (Human) | IGHJ4 (Human) | AKASGIYCSGGNCYSYYFDY | |
| IGHV3-13 (Human) | IGHJ2 (Human) | ARVGYDSSGYSGWYFDL | |
| IGHV3-53 (Human) | IGHJ4 (Human) | VRDYGDFYFDY | |
| IGHV4-34 (Human) | IGHJ3 (Human) | ARKPLLYSDFSPGAFDI | |
| IGHV4-34 (Human) | IGHJ3 (Human) | ARKPLLYSNLSPGAFDI | |
| IGHV4-61 (Human) | IGHJ4 (Human) | ARERPGGTYSNTWYTPTDTNWFDT | |
| IGHV1-18 (Human) | IGHJ4 (Human) | ARGEAVAGTTGFFDY | |
| IGHV4-4 (Human) | IGHJ3 (Human) | VRDGGRPGDAFDI | |
| IGHV3-7 (Human) | IGHJ4 (Human) | AIQLWLRGGYDY | |
| IGHV4-59 (Human) | IGHJ3 (Human) | ARVEDWGYCSSTNCYSGAFDI | |
| IGHV3-30 (Human) | IGHJ4 (Human) | AREDYYDSSGSFDY | |
| IGHV3-33 (Human) | IGHJ3 (Human) | ARGVNPDDILTGVDAFDI | |
| IGHV3-53 (Human) | IGHJ4 (Human) | ARGDGELFFDQ | |
| IGHV3-49 (Human) | IGHJ4 (Human) | TRWDGWSQHDY | |
| IGHV3-30 (Human) | IGHJ5 (Human) | ARDFYHNWFDP | |
| IGHV3-30 (Human) | IGHJ4 (Human) | ARDPIWFGELLSPPFVHFDY | |
| IGHV3-53 (Human) | IGHJ4 (Human) | AREGMGMAAAGT | |
| IGHV3-53 (Human) | IGHJ4 (Human) | ARESGDTTMAFDY | |
| IGHV3-53 (Human) | IGHJ3 (Human) | ARDLSAAFDI | |
| IGHV3-48 (Human) | IGHJ4 (Human) | AREGARVGATYDTYYFDY | |
| IGHV1-58 (Human) | IGHJ3 (Human) | AAPYCSGGSCSDAFDI | |
| IGHV4-59 (Human) | IGHJ3 (Human) | ARLQWLRGAFDI | |
| IGHV1-2 (Human) | IGHJ6 (Human) | ATAHPRRIQGVFFLGPGV | |
| IGHV3-23 (Human) | IGHJ6 (Human) | ANHPLASGDDYYHYYMDV | |
| IGHV1-46 (Human) | IGHJ2 (Human) | ARSRPTPDWYFDL | |
| IGHV1-69 (Human) | IGHJ6 (Human) | ARVNQAVTTPFSMDV | |
| IGHV4-4 (Human) | IGHJ4 (Human) | ARGGDTAMGPEYFDY | |
| IGHV3-30 (Human) | IGHJ4 (Human) | ARDSDVDTSMVTWFDY | |
| IGHV3-23 (Human) | IGHJ4 (Human) | AKDPLITGPTYQYFHY | |
| IGHV3-7 (Human) | IGHJ4 (Human) | AGGTWLRSSFDY | |
| IGHV3-30-3 (Human) | IGHJ6 (Human) | AKGGAYSYYYYMDV | |
| IGHV3-66 (Human) | IGHJ6 (Human) | ARDLYYYGMDV | |
| IGHV3-30 (Human) | IGHJ4 (Human) | ARADLGYCTNGVCYVDY | |
| IGHV3-66 (Human) | IGHJ3 (Human) | ARDSSEVRDHPGHPGRSVGAFDI | |
| IGHV3-53 (Human) | IGHJ4 (Human) | AREGEVEGYYDFWSGYSRDRYYFDY | |
| IGHV3-21 (Human) | IGHJ4 (Human) | ARDVASNYAYFDL | |
| IGHV5-51 (Human) | IGHJ5 (Human) | ARLSDRWYSPFDP | |
| IGHV3-66 (Human) | IGHJ6 (Human) | ARIANYMDV | |
| IGHV3-74 (Human) | IGHJ6 (Human) | ARPTAVAAAGNYFYYYGMDV | |
| IGHV3-21 (Human) | IGHJ4 (Human) | ARERGYDGGKTPP | |
| IGHV3-53 (Human) | IGHJ4 (Human) | ARVGGAHSGYDGSFDY | |
| IGHV3-30 (Human) | IGHJ4 (Human) | AKQAGPYCSGGSCYSAPFDY | |
| IGHV3-53 (Human) | IGHJ4 (Human) | ARDFGEFYFDY | |
| IGHV3-30 (Human) | IGHJ4 (Human) | AKDPFPLAVAGTGYFDY | |
| IGHV3-66 (Human) | IGHJ3 (Human) | ARDSSEVRDHPGHPGRSVGAFDI | |
| IGHV1-46 (Human) | IGHJ6 (Human) | ARANHETTMDTYYYYYYMDV | |
| IGHV7-4-1 (Human) | IGHJ6 (Human) | ARPQGGSSWYRDYYYGMDV | |
| IGHV1-69 (Human) | IGHJ4 (Human) | ARGPYYYDSGGYYLDY | |
| IGHV3-53 (Human) | IGHJ6 (Human) | ARDLDVYGLDV | |
| IGHV3-53 (Human) | IGHJ4 (Human) | ARDFGDFYFDY | |
| IGHV1-58 (Human) | IGHJ4 (Human) | AADPFADY | |
| IGHV5-10-1 (Human) | IGHJ4 (Human) | ARVNRVGDGPDF | |
| IGHV1-69 (Human) | IGHJ5 (Human) | ARGLWFGDSETVWFDP | |
| IGHV3-64 (Human) | IGHJ4 (Human) | VKGKIQLWLGADY | |
| IGHV4-34 (Human) | IGHJ3 (Human) | ARKPLLHSSVNPGAFDI | |
| IGHV1-69 (Human) | IGHJ4 (Human) | AREKGYSSSSSATYYLDF | |
| IGHV4-39 (Human) | IGHJ6 (Human) | ARRVPGDYYCLDV | |
| IGHV3-7 (Human) | IGHJ4 (Human) | ARGGLWGTFDY | |
| IGHV3-30-3 (Human) | IGHJ6 (Human) | ARAYGGNYYYGMDV | |
| IGHV1-69 (Human) | IGHJ5 (Human) | ASLGGDSYISGTHYDRSGYDP | |
| IGHV1-18 (Human) | IGHJ6 (Human) | ARDPASYYDFWSGYVDYYYYGMDV | |
| IGHV1-18 (Human) | IGHJ6 (Human) | ARDPASYYDLWSGYVDYYYYGMDV | |
| IGHV3-33 (Human) | IGHJ4 (Human) | AREGAVGATSGLDY | |
| IGHV4-59 (Human) | IGHJ4 (Human) | ARGFDY | |
| IGHV3-23 (Human) | IGHJ4 (Human) | AKSATIVLMVSAIY | |
| IGHV3-72 (Human) | IGHJ4 (Human) | ARVRGGEWVGDLGWYYYYGMDV | |
| IGHV3-30 (Human) | IGHJ4 (Human) | VKGATKIDY | |
| IGHV3-53 (Human) | IGHJ3 (Human) | TRGGWPSGDTFDI | |
| IGHV3-9 (Human) | IGHJ3 (Human) | AKDIGRYDHYNIFGRVGGAFDI | |
| IGHV3-66 (Human) | IGHJ6 (Human) | ARDRRIIGYYFGMDV | |
| IGHV2-5 (Human) | IGHJ3 (Human) | ARLLIEHDAFDI | |
| IGHV3-66 (Human) | IGHJ6 (Human) | VRDRRIVGYYFGLDV | |
| IGHV3-23 (Human) | IGHJ6 (Human) | AKDAFYYGSGSHFYYYYYMDV | |
| IGHV4-39 (Human) | IGHJ4 (Human) | ARDRRGGGWTASFDF | |
| IGHV3-53 (Human) | IGHJ3 (Human) | ARGGWPSGDTFDI | |
| IGHV5-51 (Human) | IGHJ6 (Human) | AGGGGISTPMDV | |
| IGHV5-51 (Human) | IGHJ6 (Human) | AGGGGISTPMDV | |
| IGHV5-51 (Human) | IGHJ6 (Human) | AGGGGISTPMDV | |
| IGHV5-51 (Human) | IGHJ6 (Human) | AGGGGISTPMDV | |
| IGHV5-51 (Human) | IGHJ6 (Human) | AGGGGISTPMDV | |
| IGHV5-51 (Human) | IGHJ6 (Human) | AGGGGISTPMDV | |
| IGHV5-51 (Human) | IGHJ6 (Human) | AGGGGISTPMDV | |
| IGHV5-51 (Human) | IGHJ6 (Human) | AGGGGISTPMDV | |
| IGHV5-51 (Human) | IGHJ6 (Human) | AGGGGISTPMDV | |
| IGHV5-51 (Human) | IGHJ6 (Human) | AGGGGISTPMDV | |
| IGHV5-51 (Human) | IGHJ6 (Human) | AGGGGISTPMDV | |
| IGHV5-51 (Human) | IGHJ6 (Human) | AGGGGISTPMDV | |
| IGHV5-51 (Human) | IGHJ6 (Human) | AGGGGISTPMDV | |
| IGHV5-51 (Human) | IGHJ6 (Human) | AGGGGISTPMDV | |
| IGHV5-51 (Human) | IGHJ6 (Human) | AGGGGISTPMDV | |
| IGHV5-51 (Human) | IGHJ6 (Human) | AGGGGISTPMDV | |
| IGHV5-51 (Human) | IGHJ6 (Human) | AGGGGISTPMDV | |
| IGHV5-51 (Human) | IGHJ6 (Human) | AGGGGISTPMDV | |
| IGHV5-51 (Human) | IGHJ6 (Human) | AGGGGISTPMDV | |
| IGHV5-51 (Human) | IGHJ6 (Human) | AGGGGISTPMDV | |
| IGHV5-51 (Human) | IGHJ6 (Human) | AGGGGISTPMDV | |
| IGHV5-51 (Human) | IGHJ6 (Human) | AGGGGISTPMDV | |
| IGHV5-51 (Human) | IGHJ6 (Human) | AGGGGISTPMDV | |
| IGHV5-51 (Human) | IGHJ6 (Human) | AGGGGISTPMDV | |
| IGHV5-51 (Human) | IGHJ6 (Human) | AGGGGISTPMDV | |
| IGHV1-69 (Human) | IGHJ4 (Human) | TTDRFVEPATGPFFDY | |
| IGHV1-69 (Human) | IGHJ5 (Human) | AESPLGGGSGYSVSWFDP | |
| IGHV3-23 (Human) | IGHJ4 (Human) | AKTRGRGLYDYVWGSKDY | |
| IGHV3-53 (Human) | IGHJ6 (Human) | ARDLGPYGMDV | |
| IGHV3-21 (Human) | IGHJ4 (Human) | ARRGSSWSFDY | |
| IGHV1-2 (Human) | IGHJ4 (Human) | ARLDY | |
| IGHV4-59 (Human) | IGHJ6 (Human) | ARDNMDV | |
| IGHV4-59 (Human) | IGHJ6 (Human) | ARDNMDV | |
| IGHV1-18 (Human) | IGHJ5 (Human) | AREITLNWNYAGWFDP | |
| IGHV3-15 (Human) | IGHJ4 (Human) | TTHSSPDY | |
| IGHV3-15 (Human) | IGHJ4 (Human) | TTHSSPDY | |
| IGHV4-4 (Human) | IGHJ6 (Human) | ARDNMDV | |
| IGHV3-15 (Human) | IGHJ4 (Human) | TTHSTPDY | |
| IGHV3-66 (Human) | IGHJ4 (Human) | ARDRGLVSDY | |
| IGHV3-53 (Human) | IGHJ6 (Human) | ARDAQNYGMDV | |
| IGHV3-66 (Human) | IGHJ3 (Human) | ARLINHYYDSSGDGGAFDI | |
| IGHV1-24 (Human) | IGHJ4 (Human) | ATGPAIAAAETNWFDL | |
| IGHV3-21 (Human) | IGHJ6 (Human) | ATNGGAHSSTWSFYGMDV | |
| IGHV4-39 (Human) | IGHJ4 (Human) | ARVSSGYYFTPFDY | |
| IGHV3-30 (Human) | IGHJ3 (Human) | ARARGGSYNDAFDI | |
| IGHV3-30 (Human) | IGHJ6 (Human) | AKDGQYYDFWSGYLGARTNPHYYYYMDV | |
| IGHV3-9 (Human) | IGHJ6 (Human) | AKDMGEAVAGTHYGMDV | |
| IGHV1-69 (Human) | IGHJ4 (Human) | ARVGAYDSSGYSNDY | |
| IGHV3-30 (Human) | IGHJ4 (Human) | AREDYYDSSGSFDY | |
| IGHV4-59 (Human) | IGHJ3 (Human) | ARHSQGWLQQAVAFDI | |
| IGHV3-66 (Human) | IGHJ4 (Human) | ARGGYYYDPSGYYSRSFSFDY | |
| IGHV1-2 (Human) | IGHJ6 (Human) | ARDLVWATVSGTMDV | |
| IGHV3-66 (Human) | IGHJ4 (Human) | ARVEWAAAGTFY | |
| IGHV3-21 (Human) | IGHJ3 (Human) | ARWKSDYYDSSGYYPAAFDI | |
| IGHV3-21 (Human) | IGHJ6 (Human) | AGDQNLYCSGDSCYYHYYGMDV | |
| IGHV3-30 (Human) | IGHJ6 (Human) | ARDSEYYDILTGYLAPTHYYYYYMDV | |
| IGHV1-18 (Human) | IGHJ6 (Human) | ARDLVDTAMVQTLDDYGMDV | |
| IGHV5-51 (Human) | IGHJ4 (Human) | ARYYYDSRGYTSIDF | |
| IGHV4-39 (Human) | IGHJ6 (Human) | ARLNYDFWSGYYSYALYYMDV | |
| IGHV4-31 (Human) | IGHJ4 (Human) | ARQQLDYYDSSGCFDY | |
| IGHV3-13 (Human) | IGHJ2 (Human) | ARGGDRYPVGYFDL | |
| IGHV4-39 (Human) | IGHJ6 (Human) | ARRSTSRWGYYYMDV | |
| IGHV3-48 (Human) | IGHJ4 (Human) | AREANSDFWSGYLGYFDY | |
| IGHV5-51 (Human) | IGHJ6 (Human) | ARHMRPSIAARPGYQYYMDV | |
| IGHV3-53 (Human) | IGHJ6 (Human) | AREAYGMDV | |
| IGHV3-23 (Human) | IGHJ1 (Human) | AKGLRGQQLVIPTEYFQH | |
| IGHV1-69 (Human) | IGHJ4 (Human) | ARGPRGCSSTSCYGSYFDY | |
| IGHV3-21 (Human) | IGHJ4 (Human) | ARDQPLPDILTGYYTGPLDY | |
| IGHV3-53 (Human) | IGHJ6 (Human) | ARDLDTMGGMDV | |
| IGHV1-69 (Human) | IGHJ4 (Human) | ARVRYYDSSGYYEDY | |
| IGHV3-23 (Human) | IGHJ4 (Human) | AKDTGYCGDDCYIKLIRGGPDY | |
| IGHV1-69 (Human) | IGHJ4 (Human) | ASSDSSGFVGSRGFDY | |
| IGHV1-69 (Human) | IGHJ4 (Human) | ASFGDDSGDEGVR | |
| IGHV1-69 (Human) | IGHJ4 (Human) | ARVYSYDSSGYYLEY | |
| IGHV3-53 (Human) | IGHJ6 (Human) | ASPLLLTPPDYYYYMDV | |
| IGHV4-4 (Human) | IGHJ4 (Human) | ARWKYNDRFDY | |
| IGHV1-69 (Human) | IGHJ4 (Human) | ARDGFGDVEEMATIKDAFDI | |
| IGHV5-10-1 (Human) | IGHJ3 (Human) | ARPNPAGGYDSSGWVDAFDI | |
| IGHV1-24 (Human) | IGHJ5 (Human) | ATGPTIAAAATNWFDP | |
| IGHV3-15 (Human) | IGHJ6 (Human) | TTDRGDSYGYYYCMDV | |
| IGHV3-33 (Human) | IGHJ4 (Human) | AKDKAPPCSSGWYYFDY | |
| IGHV3-30 (Human) | IGHJ3 (Human) | ARGVEDPVVPAAIPWCWFDP | |
| IGHV4-30-4 (Human) | IGHJ5 (Human) | ASLPVVPAAIGPLPAFDI | |
| IGHV1-2 (Human) | IGHJ3 (Human) | RKMLTIFGKVNQTMLLISGAKGQ | |
| IGHV1-69 (Human) | IGHJ4 (Human) | ARTHSYDNSGQYFDY | |
| IGHV5-10-1 (Human) | IGHJ6 (Human) | ARLKVITIFGVVRDDYGMDV | |
| IGHV3-30 (Human) | IGHJ4 (Human) | ARSASGSYYGAFDY | |
| IGHV1-24 (Human) | IGHJ5 (Human) | ATSPAVMSVGWVDP | |
| IGHV4-39 (Human) | IGHJ3 (Human) | ARQVRQWLEDDAFDI | |
| IGHV4-4 (Human) | IGHJ5 (Human) | AGRYCSGGRCGWFDP | |
| IGHV3-21 (Human) | IGHJ6 (Human) | ARVQKDIVVVPVALADYYYYGMDV | |
| IGHV1-18 (Human) | IGHJ4 (Human) | ARFDYGYPYSSWSVLSIDY | |
| IGHV3-30 (Human) | IGHJ4 (Human) | AREGSRQWLVIYFDY | |
| IGHV1-2 (Human) | IGHJ5 (Human) | ARGDGDYYDSSGYYRPTLYNWLDP | |
| IGHV4-39 (Human) | IGHJ4 (Human) | ARHPSGLYQLLN | |
| IGHV3-9 (Human) | IGHJ4 (Human) | AKVATYYYDRSGYYYGGALDY | |
| IGHV4-59 (Human) | IGHJ6 (Human) | ARGPAATYYYYMDV | |
| IGHV3-23 (Human) | IGHJ4 (Human) | AKEIAVAGCFDY | |
| IGHV3-33 (Human) | IGHJ4 (Human) | ARVGSVKSTAGYDFWSGDPFDY | |
| IGHV1-24 (Human) | IGHJ4 (Human) | ATAYSVDTAMVRGVGY | |
| IGHV1-69 (Human) | IGHJ4 (Human) | ARDAPDYYDSSGPTYFDY | |
| IGHV3-9 (Human) | IGHJ6 (Human) | AKMGPDPAHDYGRKNDAFDI | |
| IGHV5-51 (Human) | IGHJ6 (Human) | ARRGYTYGADFYGLDV | |
| IGHV4-59 (Human) | IGHJ2 (Human) | ARDRIAPVGKFFGWYFDL | |
| IGHV3-64 (Human) | IGHJ4 (Human) | ARSSSRGFDY | |
| IGHV4-39 (Human) | IGHJ4 (Human) | ARELFTAVAGKGGIDY | |
| IGHV3-30 (Human) | IGHJ6 (Human) | AKDFKGGSSSWYTPEIEYGMDV | |
| IGHV7-4-1 (Human) | IGHJ6 (Human) | ARLIRHEAHTYCSGGSCYSPDYYYGMDV | |
| IGHV3-30-3 (Human) | IGHJ6 (Human) | ARWGGGMQYLDV | |
| IGHV3-30 (Human) | IGHJ6 (Human) | AKDLGYYDILTGQLGGYYYYYGMDV | |
| IGHV3-7 (Human) | IGHJ3 (Human) | ARDWDYDILTGSWFGAFDI | |
| IGHV3-64 (Human) | IGHJ4 (Human) | ARGAEYYDFWSGYYSAYFDY | |
| IGHV3-66 (Human) | IGHJ6 (Human) | ARATWLRGVMDVW | |
| IGHV3-9 (Human) | IGHJ3 (Human) | AKDVRYCSSTSCYFSAFDI | |
| IGHV3-30 (Human) | IGHJ3 (Human) | ARGGGSYYYWFDP | |
| IGHV4-34 (Human) | IGHJ3 (Human) | ARAGYSSSWYGVRGVDP | |
| IGHV3-30 (Human) | IGHJ5 (Human) | AKGSDIVVVPVGNWFDP | |
| IGHV4-61 (Human) | IGHJ4 (Human) | ARVQRYYPDSSGFYGRRFDI | |
| IGHV3-30 (Human) | IGHJ4 (Human) | ARSGGGSYRGPFDY | |
| IGHV4-39 (Human) | IGHJ3 (Human) | ARGDRIQLWLLDAFDI | |
| IGHV3-23 (Human) | IGHJ4 (Human) | AKIGLGLGGLLRRYFDY | |
| IGHV3-11 (Human) | IGHJ6 (Human) | GTRIMITWYSRRGMDG | |
| IGHV1-46 (Human) | IGHJ6 (Human) | ARERGDSSGYYEIITTANRRFGMDV | |
| IGHV1-69 (Human) | IGHJ6 (Human) | ARIPGWDRGTDRNWNDD | |
| IGHV1-69 (Human) | IGHJ4 (Human) | ARTYSFDSSGYYYDY | |
| IGHV3-30 (Human) | IGHJ5 (Human) | ARAFYDSNWSVGSYFDS | |
| IGHV3-9 (Human) | IGHJ4 (Human) | AKDSVRREYTHARVPFDN | |
| IGHV3-30 (Human) | IGHJ4 (Human) | AKSSKIFYLGESREVDY | |
| IGHV3-7 (Human) | IGHJ4 (Human) | ARPTIGYSYGSDY | |
| IGHV3-30 (Human) | IGHJ6 (Human) | AKVSAIFWLGQGLSPIDV | |
| IGHV4-38-2 (Human) | IGHJ3 (Human) | ARAVVGIVVVPAAGRRAFDI | |
| IGHV3-30-3 (Human) | IGHJ4 (Human) | ARDQEWFRELFLFDY | |
| IGHV3-30 (Human) | IGHJ4 (Human) | AKDFGGDNTAMVEYFFDF | |
| IGHV3-7 (Human) | IGHJ4 (Human) | ARDPYDLYGDYGGTFDY | |
| IGHV7-4-1 (Human) | IGHJ6 (Human) | ARDQDSGYPTYYYYYMDV | |
| IGHV3-13 (Human) | IGHJ6 (Human) | ARADPYQLLGQHYYYGMDV | |
| IGHV3-7 (Human) | IGHJ3 (Human) | ARDWDYDILTGSWFGAFDI | |
| IGHV4-38-2 (Human) | IGHJ2 (Human) | ARTPLSLRLRYNWYFDL | |
| IGHV4-59 (Human) | IGHJ4 (Human) | ARGFDY | |
| IGHV4-31 (Human) | IGHJ4 (Human) | ARETTGHFDY | |
| IGHV3-30 (Human) | IGHJ4 (Human) | VRGGVSGPNSFDM | |
| IGHV7-4-1 (Human) | IGHJ4 (Human) | ARASARPGVATNLDF | |
| IGHV3-7 (Human) | IGHJ6 (Human) | ARDFNSYQLLWYYYYGMDV | |
| IGHV5-51 (Human) | IGHJ6 (Human) | ARQSSFYSSGWYSYGMDV | |
| IGHV1-2 (Human) | IGHJ5 (Human) | ARVDYGSGSYGWGWFDP | |
| IGHV1-24 (Human) | IGHJ5 (Human) | ATTSPIVGAITWFDP | |
| IGHV1-2 (Human) | IGHJ6 (Human) | AREYYYDSSVYPYYYYAMDV | |
| IGHV3-30 (Human) | IGHJ4 (Human) | ARVRGSYYLFDY | |
| IGHV3-15 (Human) | IGHJ4 (Human) | TTDRVYDYIWGSYRYLDY | |
| IGHV3-21 (Human) | IGHJ4 (Human) | ARDRESYDILTGYSMEGCFDY | |
| IGHV1-3 (Human) | IGHJ3 (Human) | ARVWGYCSGGSCYVDAFDI | |
| IGHV1-24 (Human) | IGHJ5 (Human) | ATAPPYSPPSSWFDP | |
| IGHV4-30-4 (Human) | IGHJ6 (Human) | ARDHHYDFWSGYSSYYYYGMDV | |
| IGHV3-30-3 (Human) | IGHJ6 (Human) | ARDEAYYDILTGYINAPKNYYYYGMDV | |
| IGHV3-30 (Human) | IGHJ6 (Human) | ARSFGGSYYYGMDV | |
| IGHV7-4-1 (Human) | IGHJ4 (Human) | ARASARPGVATNLDF | |
| IGHV1-24 (Human) | IGHJ5 (Human) | ATAPPYSPPSSWFDP | |
| IGHV1-2 (Human) | IGHJ4 (Human) | AREARDYYGSGSLDY | |
| IGHV1-18 (Human) | IGHJ6 (Human) | ARDSVAGIYYYYGMDV | |
| IGHV3-30-3 (Human) | IGHJ6 (Human) | ARSYGGSYYYGMDV | |
| IGHV4-38 (Human) | IGHJ2 (Human) | ARTPLSLRLRYNWYFDL | |
| IGHV1-2 (Human) | IGHJ6 (Human) | ARDLTTTAGTDYYYGMDV | |
| IGHV1-18 (Human) | IGHJ4 (Human) | ARARVAYDYIWGSYRYKAFDY | |
| IGHV3-30 (Human) | IGHJ4 (Human) | ARAQTAHYSSSFDY | |
| IGHV3-30 (Human) | IGHJ3 (Human) | VRGGVSGPNAFDI | |
| IGHV3-30 (Human) | IGHJ4 (Human) | ARSISGSYLGAFDY | |
| IGHV1-18 (Human) | IGHJ3 (Human) | ARVGLWWLGHPDAFDI | |
| IGHV3-30 (Human) | IGHJ4 (Human) | ARTKGGSYFAPFDY | |
| IGHV1-18 (Human) | IGHJ4 (Human) | ARDRGYAATFGVFDY | |
| IGHV3-30 (Human) | IGHJ4 (Human) | ARVTVVHFDY | |
| IGHV1-46 (Human) | IGHJ6 (Human) | ARDLTSTSSSPYSYYYGMDV | |
| IGHV1-18 (Human) | IGHJ5 (Human) | ARVTVEAIFGVVILPLKNWFDP | |
| IGHV3-30 (Human) | IGHJ4 (Human) | VRGGVSGPNSFDM | |
| IGHV1-18 (Human) | IGHJ3 (Human) | ARVGLWWLGHPDVFDI | |
| IGHV1-69 (Human) | IGHJ4 (Human) | ARDLVEDTAMVTGAAAGT | |
| IGHV1-46 (Human) | IGHJ3 (Human) | ARAGRRYSSSDDGAFDI | |
| IGHV3-33 (Human) | IGHJ4 (Human) | ARDIMFGDDWLQKQPDY | |
| IGHV3-30 (Human) | IGHJ4 (Human) | ARSISGSYLGAFDY | |
| IGHV1-18 (Human) | IGHJ6 (Human) | ARLVPTWASYYDFWSGYPGGYGMDV | |
| IGHV4-39 (Human) | IGHJ2 (Human) | ATHIVVVTATPNWYFDL | |
| IGHV3-53 (Human) | IGHJ4 (Human) | AREGDVEGYYDFWSGYSRDRYYFDY | |
| IGHV3-53 (Human) | IGHJ4 (Human) | AREGDVEGYYDFWSGYSRDRYYFDY | |
| IGHV3-53 (Human) | IGHJ4 (Human) | AREGDVEGYYDFWSGYSRDRYYFDY | |
| IGHV3-66 (Human) | IGHJ3 (Human) | ARDSSEVRDHPGHPGRSVGAFDI | |
| IGHV5-51 (Human) | IGHJ2 (Human) | ARGVAVDWYFDL | |
| IGHV3-53 (Human) | IGHJ3 (Human) | ARDLYSSGGTDI | |
| IGHV3-53 (Human) | IGHJ3 (Human) | ARDLYSSGGTDI | |
| IGHV4-4 (Human) | IGHJ4 (Human) | AKGGDTAMGPEYFDY | |
| IGHV1-69 (Human) | IGHJ6 (Human) | ARREPYGPRDYYYFFGMDV | |
| IGHV1-69-2 (Human) | IGHJ4 (Human) | ATSSGPSRLCGGGSCYHSFDY | |
| IGHV1-2 (Human) | IGHJ6 (Human) | AREVMVRGALPPYGMDV | |
| IGHV3-66 (Human) | IGHJ4 (Human) | AAGSGSSATEVYEEFDY | |
| IGHV3-66 (Human) | IGHJ4 (Human) | AAGSVVADLSVEEFDY | |
| IGHV4-59 (Human) | IGHJ3 (Human) | ARLERDWPLDAFDI | |
| IGHV3-53 (Human) | IGHJ3 (Human) | AREGPKSITGTAFDI | |
| IGHV3-30 (Human) | IGHJ4 (Human) | ARDGQAITMVQGVIGPPFDY | |
| IGHV3-53 (Human) | IGHJ4 (Human) | ARETLAFDY | |
| IGHV4-59 (Human) | IGHJ2 (Human) | ASNGQYYDILTGQPPDYWYFDL | |
| IGHV1-8 (Human) | IGHJ5 (Human) | ARYIVVVPAAKGFDP | |
| IGHV4-61 (Human) | IGHJ5 (Human) | ARERCYYGSGRAPRCVWFDP | |
| IGHV3-33 (Human) | IGHJ4 (Human) | ARWFHTGGYFDY | |
| IGHV3-53 (Human) | IGHJ4 (Human) | ARDYGDFYFDY | |
| IGHV2-5 (Human) | IGHJ4 (Human) | AHSLFLTVGYSSSWSPFDY | |
| IGHV3-53 (Human) | IGHJ6 (Human) | ARDLMVYGIDV | |
| IGHV3-53 (Human) | IGHJ6 (Human) | ARDLGSGDMDV | |
| IGHV3-30 (Human) | IGHJ6 (Human) | AKDRDDGWDWYYFMDV | |
| IGHV1-58 (Human) | IGHJ3 (Human) | AAPHCNSTSCYDAFDI | |
| IGHV1-58 (Human) | IGHJ3 (Human) | AAPHCNSTSCYDAFDI | |
| IGHV1-2 (Human) | IGHJ4 (Human) | ARDMAFSMVRGSFDY | |
| IGHV3-11 (Human) | IGHJ3 (Human) | ARGGVLRFLEWPLNAFDI | |
| IGHV3-53 (Human) | IGHJ6 (Human) | ARDLGSGDMDV | |
| IGHV4-61 (Human) | IGHJ6 (Human) | ARHCSGGTCYPKYYYGMDV | |
| IGHV3-53 (Human) | IGHJ3 (Human) | ARDFYEGSFDI | |
| IGHV3-66 (Human) | IGHJ4 (Human) | ARDLVVAGIDY | |
| IGHV3-30 (Human) | IGHJ6 (Human) | AKDMHNDYGDYVSYYFYYGMDV | |
| IGHV1-24 (Human) | IGHJ6 (Human) | ATALAVAGDRGHYHYYFGMDV | |
| IGHV4-38-2 (Human) | IGHJ2 (Human) | ARFDGEVLVYHDWPKPAWVDL | |
| IGHV1-18 (Human) | IGHJ6 (Human) | AREGYCSGGSCYSGYYYYYGMDV | |
| IGHV3-30 (Human) | IGHJ4 (Human) | AKQGGPYCSGGNCNVGYFDY | |
| IGHV3-7 (Human) | IGHJ4 (Human) | ARARRADNSGYYGFHFDC | |
| IGHV3-72 (Human) | IGHJ4 (Human) | ARVHRWAYCINGVCFGAYSDY | |
| IGHV2-5 (Human) | IGHJ4 (Human) | AHHKIERIFDY | |
| IGHV3-53 (Human) | IGHJ4 (Human) | ARGDVSGYRYGLDY | |
| IGHV3-53 (Human) | IGHJ4 (Human) | ARGDVSGYRYGLDY | |
| IGHV3-30 (Human) | IGHJ5 (Human) | AKDSGYNYGYSWFDP | |
| IGHV1-24 (Human) | IGHJ6 (Human) | ATGIAVIGPPPSTYYYYGMDV | |
| IGHV3-33 (Human) | IGHJ6 (Human) | ARDPRDYYDFWSGYDYYYGLDV | |
| IGHV3-30 (Human) | IGHJ5 (Human) | ARETGDYSSSWYDS | |
| IGHV3-30 (Human) | IGHJ6 (Human) | ARVWLYGSGYMDV | |
| IGHV3-33 (Human) | IGHJ4 (Human) | ATDPPGLRFRFDY | |
| IGHV1-46 (Human) | IGHJ2 (Human) | ARADGYEWYFDV | |
| IGHV1-69 (Human) | IGHJ4 (Human) | AREGRRYGSGWYISTGYFDY | |
| IGHV3-30 (Human) | IGHJ6 (Human) | AKDLVLRYFDSMDV | |
| IGHV3-30 (Human) | IGHJ6 (Human) | AKDVVLRYFDAMDV | |
| IGHV3-30 (Human) | IGHJ6 (Human) | AKDVVLRYFDAMDV | |
| IGHV1-46 (Human) | IGHJ6 (Human) | ARGGSHGMDV | |
| IGHV2-5 (Human) | IGHJ4 (Human) | AHHSISTIFDH | |
| IGHV4-59 (Human) | IGHJ4 (Human) | ASSQRPDGNLYYFDY | |
| IGHV3-23 (Human) | IGHJ6 (Human) | ARDNLGYRPSENLYGMDV | |
| IGHV3-66 (Human) | IGHJ4 (Human) | ARGYGDYYFDY | |
| IGHV1-46 (Human) | IGHJ4 (Human) | ADLLLDY | |
| IGHV3-66 (Human) | IGHJ6 (Human) | VRDLYSYGMDV | |
| IGHV3-30 (Human) | IGHJ4 (Human) | AREGQWLNWAFDY | |
| IGHV3-66 (Human) | IGHJ6 (Human) | AAPLLWADSYYMDV | |
| IGHV1-24 (Human) | IGHJ6 (Human) | ATALPITMVRGVQYYYYGMDV | |
| IGHV1-24 (Human) | IGHJ5 (Human) | ATFFAVRGALNWFDS | |
| IGHV1-69 (Human) | IGHJ4 (Human) | ARGIVGATPGYFDY | |
| IGHV1-69 (Human) | IGHJ2 (Human) | ARETGDQGVTAPFDL | |
| IGHV1-58 (Human) | IGHJ3 (Human) | AAPYCSSTTCHDGFDI | |
| IGHV1-24 (Human) | IGHJ4 (Human) | TTVGFPDYYDSSVYLRHFDY | |
| IGHV3-30 (Human) | IGHJ4 (Human) | ATGTYDFWSDNHYLDY | |
| IGHV1-69 (Human) | IGHJ4 (Human) | ARRAIDSDTYVEQSHFDY | |
| IGHV1-69 (Human) | IGHJ6 (Human) | ANFIGDGYNYEEDYMDV | |
| IGHV3-11 (Human) | IGHJ5 (Human) | ARGRLWGWFDP | |
| IGHV3-53 (Human) | IGHJ6 (Human) | AREAYGMDV | |
| IGHV3-53 (Human) | IGHJ6 (Human) | ARDLVVYGMDV | |
| IGHV3-66 (Human) | IGHJ6 (Human) | ARDLVVYGMDV | |
| IGHV3-53 (Human) | IGHJ6 (Human) | ARDAMSYGMDV | |
| IGHV3-53 (Human) | IGHJ6 (Human) | ARDAAVYGIDV | |
| IGHV3-66 (Human) | IGHJ6 (Human) | ARDLISRGMDV | |
| IGHV3-53 (Human) | IGHJ6 (Human) | ARDRVVYGMDV | |
| IGHV3-53 (Human) | IGHJ6 (Human) | ARDLVSYGMDV | |
| IGHV3-53 (Human) | IGHJ6 (Human) | ARDLVVYGMDV | |
| IGHV4-59 (Human) | IGHJ3 (Human) | ARGVLLWFGEPIFEI | |
| IGHV4-34 (Human) | IGHJ3 (Human) | ARRWWLRGAFDI | |
| IGHV3-30 (Human) | IGHJ4 (Human) | ARLITMVRGEDY | |
| IGHV1-24 (Human) | IGHJ6 (Human) | VTAPAITGSPEAYSYYYGMDV | |
| IGHV1-24 (Human) | IGHJ5 (Human) | ATGPAIAAAATGWFDP | |
| IGHV1-24 (Human) | IGHJ6 (Human) | VTAPVITGSPEAYSYYYGMDV | |
| IGHV3-30 (Human) | IGHJ6 (Human) | AKDHDDGYYFYYYMDV | |
| IGHV1-24 (Human) | IGHJ6 (Human) | AASPAVRGSPSNFYYYHGMDV | |
| IGHV1-24 (Human) | IGHJ6 (Human) | VAAPVITGSPEAYSYYYGMDV | |
| IGHV4-30 (Human) | IGHJ3 (Human) | ARMAYQVYYYDSSGYYDAFDI | |
| IGHV4-61 (Human) | IGHJ3 (Human) | ARMAYQVYYYDSSGYYDAFDI | |
| IGHV1-24 (Human) | IGHJ5 (Human) | ATSRVAGTPNWFHP | |
| IGHV3-30 (Human) | IGHJ5 (Human) | ARDLGSGWYP | |
| IGHV1-24 (Human) | IGHJ5 (Human) | ATSRVAGTPNWFHP | |
| IGHV1-24 (Human) | IGHJ4 (Human) | ATAAAVRGRGTIDY | |
| IGHV1-24 (Human) | IGHJ5 (Human) | ATGPAVRRGSWFDP | |
| IGHV3-30 (Human) | IGHJ4 (Human) | ARGAMAGNSSFDH | |
| IGHV3-11 (Human) | IGHJ5 (Human) | ARVGPAVAGSPFDS | |
| IGHV2-5 (Human) | IGHJ4 (Human) | SHVWVNSTHRQIDY | |
| IGHV1-2 (Human) | IGHJ4 (Human) | ARGHRIPSAISDKYDF | |
| IGHV1-24 (Human) | IGHJ5 (Human) | ATGPVRGVIGWFDP | |
| IGHV3-53 (Human) | IGHJ4 (Human) | ARDLQELGSLDY | |
| IGHV3-7 (Human) | IGHJ4 (Human) | ARVGSSSWYFDY | |
| IGHV5-51 (Human) | IGHJ6 (Human) | AGGSGISTPMDV | |
| IGHV3-7 (Human) | IGHJ4 (Human) | ARLGGSSWHFDY | |
| IGHV4-34 (Human) | IGHJ4 (Human) | ARGNTMVRGVIIPFEY | |
| IGHV3-66 (Human) | IGHJ5 (Human) | ASSRPPIGQLVPGLDLDWFDP | |
| IGHV5-51 (Human) | IGHJ6 (Human) | ARTQWGYNYGSHFFYMDV | |
| IGHV3-53 (Human) | IGHJ6 (Human) | ARDLEVVGAMDV | |
| IGHV1-18 (Human) | IGHJ4 (Human) | AIPYSSVTFDC | |
| IGHV1-8 (Human) | IGHJ6 (Human) | ARGGRYCSSTTCYSGVGMDV | |
| IGHV1-18 (Human) | IGHJ4 (Human) | ARDYTRGAWFGESLIGGFDN | |
| IGHV3-66 (Human) | IGHJ6 (Human) | AREYYYGMDV | |
| IGHV4-31 (Human) | IGHJ4 (Human) | ATPGAIMGALHI | |
| IGHV3-66 (Human) | IGHJ4 (Human) | ARVLPMYGDYLDY | |
| IGHV3-15 (Human) | IGHJ4 (Human) | TTAGSYYYDTVGPGLPEGKFDY | |
| IGHV1-58 (Human) | IGHJ3 (Human) | AAPYCSSISCNDGFDI | |
| IGHV3-73 (Human) | IGHJ4 (Human) | TSVCSGGSCYQ | |
| IGHV3-74 (Human) | IGHJ4 (Human) | STDSGSIGEF | |
| IGHV3-49 (Human) | IGHJ6 (Human) | TRISGYYGAGSGGAMDV | |
| IGHV3-30 (Human) | IGHJ3 (Human) | ARSGWDDAFDI | |
| IGHV1-8 (Human) | IGHJ6 (Human) | ARGGNGGMDV | |
| IGHV3-9 (Human) | IGHJ6 (Human) | AKVGEVGSREWSAFDV | |
| IGHV4-4 (Human) | IGHJ3 (Human) | ARHNAQFGELLVPQDAFDM | |
| IGHV1-69 (Human) | IGHJ4 (Human) | VRERGYSGYGAAYYFDY | |
| IGHV3-23 (Human) | IGHJ5 (Human) | VKDFGHLGQMAS | |
| IGHV3-23 (Human) | IGHJ4 (Human) | VKDLGFADH | |
| IGHV3-23 (Human) | IGHJ4 (Human) | AREWHSGYDY | |
| IGHV3-23 (Human) | IGHJ4 (Human) | VKDFVVGETAEFSY | |
| IGHV3-9 (Human) | IGHJ6 (Human) | ARGGWSSSAGGYYGMDV | |
| IGHV3-30 (Human) | IGHJ6 (Human) | AKAMFLGDSSGLTGLDMDV | |
| IGHV3-30 (Human) | IGHJ6 (Human) | ARAYSSSWLLQSFYYYGMDV | |
| IGHV3-30 (Human) | IGHJ4 (Human) | ARHATLMNNKDI | |
| IGHV3-53 (Human) | IGHJ6 (Human) | ARDLVVYGMDV | |
| IGHV3-66 (Human) | IGHJ6 (Human) | ARDLDILGGMDV | |
| IGHV1-69 (Human) | IGHJ6 (Human) | AREVSDFDWLYRSHYGMDV | |
| IGHV3-23 (Human) | IGHJ4 (Human) | AKGTDAFDY | |
| IGHV3-23 (Human) | IGHJ4 (Human) | AKNSDSFDY | |
| IGHV3-23 (Human) | IGHJ4 (Human) | AKDSDTFDY | |
| IGHV3-23 (Human) | IGHJ4 (Human) | AKNDSSFDY | |
| IGHV3-23 (Human) | IGHJ4 (Human) | AKSTNTFDY | |
| IGHV3-43 (Human) | IGHJ6 (Human) | AKDRSYGPPDVFNYEYGMDV | |
| IGHV3-11 (Human) | IGHJ4 (Human) | ARDRGTTMVPFDY | |
| IGHV3-15 (Human) | IGHJ2 (Human) | TTARWDWYFDL | |
| IGHV3-66 (Human) | IGHJ4 (Human) | ARDHGMAAAGYNY | |
| IGHV4-4 (Human) | IGHJ4 (Human) | ANMVRGVYEDDY | |
| IGHV1-69 (Human) | IGHJ4 (Human) | ARTPFYYDSSGYYLDY | |
| IGHV3-66 (Human) | IGHJ6 (Human) | ARGELGIPYGMDV | |
| IGHV3-66 (Human) | IGHJ4 (Human) | ARALPYGDLHFDY | |
| IGHV1-2 (Human) | IGHJ5 (Human) | ARGSRYDWNQNNWFDP | |
| IGHV3-48 (Human) | IGHJ6 (Human) | ARDGFYYYYAMDV | |
| IGHV3-66 (Human) | IGHJ4 (Human) | ARDLGGYFDY | |
| IGHV3-66 (Human) | IGHJ3 (Human) | ARERNFDAFDI | |
| IGHV3-48 (Human) | IGHJ4 (Human) | ASSSSSGYYFDY | |
| IGHV1-2 (Human) | IGHJ5 (Human) | AIITIFGVVTWFDP | |
| IGHV4-59 (Human) | IGHJ4 (Human) | ARDPLLIDY | |
| IGHV3-9 (Human) | IGHJ6 (Human) | AKDKDWNSRGYYYYGMDV | |
| IGHV3-66 (Human) | IGHJ3 (Human) | ARARGDAFDI | |
| IGHV3-66 (Human) | IGHJ6 (Human) | ARVPLGDYYYGMDV | |
| IGHV3-33 (Human) | IGHJ3 (Human) | ARDPPITGTTGGDAFDI | |
| IGHV2-5 (Human) | IGHJ4 (Human) | AHGPTGDYFDY | |
| IGHV3-66 (Human) | IGHJ4 (Human) | AREGGYSYDYN | |
| IGHV3-66 (Human) | IGHJ6 (Human) | TRDAQYYGMDV | |
| IGHV3-9 (Human) | IGHJ6 (Human) | SKDMGRLDYYSGLDV | |
| IGHV3-66 (Human) | IGHJ6 (Human) | VRDLEVRGGMDV | |
| IGHV3-66 (Human) | IGHJ5 (Human) | ARYIPRFDP | |
| IGHV3-66 (Human) | IGHJ6 (Human) | ARDLIKYGMDV | |
| IGHV3-66 (Human) | IGHJ6 (Human) | ARDLGPYGMDV | |
| IGHV3-53 (Human) | IGHJ4 (Human) | ARALPGWGGSFEYFDY | |
| IGHV3-66 (Human) | IGHJ6 (Human) | AREGRIAATGYGMDV | |
| IGHV3-66 (Human) | IGHJ4 (Human) | ARDQRDFA | |
| IGHV3-66 (Human) | IGHJ6 (Human) | VRDPVGRYYYGMDV | |
| IGHV3-53 (Human) | IGHJ6 (Human) | ARDLYYYGMDV | |
| IGHV3-66 (Human) | IGHJ6 (Human) | ARGEGANYYGMDV | |
| IGHV3-9 (Human) | IGHJ6 (Human) | AKDGERWDSVVVPSARNGMDV | |
| IGHV3-48 (Human) | IGHJ6 (Human) | ARRGDGTSSLIHHYYYMDV | |
| IGHV3-66 (Human) | IGHJ6 (Human) | ARDGVSYGMDV | |
| IGHV3-66 (Human) | IGHJ6 (Human) | ARDRPITGTTLDV | |
| IGHV3-66 (Human) | IGHJ6 (Human) | ARDGSAYGMDV | |
| IGHV3-66 (Human) | IGHJ6 (Human) | ARDLGTGGMDV | |
| IGHV3-30 (Human) | IGHJ3 (Human) | AKDREAAADAFDI | |
| IGHV3-66 (Human) | IGHJ4 (Human) | ARELRGYFDY | |
| IGHV3-66 (Human) | IGHJ4 (Human) | ARDLAAAGTDY | |
| IGHV3-11 (Human) | IGHJ5 (Human) | ARDLVVYGMDV | |
| IGHV3-11 (Human) | IGHJ5 (Human) | ARDFSHQQLVPS | |
| IGHV3-23 (Human) | IGHJ4 (Human) | AKAAGSFDY | |
| IGHV3-53 (Human) | IGHJ6 (Human) | ARDLFYYGMDV | |
| IGHV3-30 (Human) | IGHJ6 (Human) | ARSILYGGGMDV | |
| IGHV4-59 (Human) | IGHJ3 (Human) | AREVYYYDRSGYYASDGFDI | |
| IGHV3-21 (Human) | IGHJ3 (Human) | TRAGWELRLDAFDI | |
| IGHV3-53 (Human) | IGHJ6 (Human) | ARDLGEAGGMDV | |
| IGHV3-53 (Human) | IGHJ4 (Human) | ARDYGDYYFDY | |
| IGHV3-30 (Human) | IGHJ4 (Human) | AAQDSAYIKSKGSRAYEY | |
| IGHV3-23 (Human) | IGHJ4 (Human) | AAHHIPTKHPAFPDFRDY | |
| IGHV3-30 (Human) | IGHJ4 (Human) | AAEAFVQSPYSGSHTTKY | |
| IGHV3-23 (Human) | IGHJ4 (Human) | AADQYEWWVPGEVGPYLY | |
| IGHV3-30 (Human) | IGHJ4 (Human) | AAHYEFNDFVWQGYSSDY | |
| IGHV3-30 (Human) | IGHJ6 (Human) | ASGYTGYDYFVRGDYYGLDV | |
| IGHV4-39 (Human) | IGHJ1 (Human) | ARHAAAYYDRSGYYFIEYFQH | |
| IGHV1-46 (Human) | IGHJ4 (Human) | ASDPRDDIAGGY | |
| IGHV1-69 (Human) | IGHJ4 (Human) | ARDRGDTIDY | |
| IGHV3-66 (Human) | IGHJ3 (Human) | ARDVADAFDI | |
| IGHV1-69 (Human) | IGHJ4 (Human) | ARDGDSGSYYETLGFDY | |
| IGHV3-66 (Human) | IGHJ6 (Human) | ARDLDYYGMDV | |
| IGHV3-33 (Human) | IGHJ6 (Human) | ARETVSYGMDV | |
| IGHV4-59 (Human) | IGHJ3 (Human) | ARDRGYSSGWTDGFDI | |
| IGHV3-30 (Human) | IGHJ6 (Human) | AKDQDDGYYYYYYMDV | |
| IGHV4-59 (Human) | IGHJ5 (Human) | ARETRYNWFDP | |
| IGHV3-9 (Human) | IGHJ4 (Human) | AKDPGYDSSGYYYDY | |
| IGHV3-7 (Human) | IGHJ4 (Human) | ASLRWLQADFEY | |
| IGHV4-59 (Human) | IGHJ4 (Human) | ARETRWNYVDY | |
| IGHV3-30-3 (Human) | IGHJ3 (Human) | ARARGGSYSDAFDI | |
| IGHV3-48 (Human) | IGHJ6 (Human) | ARDRGGYGPYYYGMDV | |
| IGHV3-30 (Human) | IGHJ6 (Human) | AKVVAPYYYYYYGMDV | |
| IGHV3-30-3 (Human) | IGHJ4 (Human) | ARGKGGSYFGSFDY | |
| IGHV1-18 (Human) | IGHJ4 (Human) | ARSIAVAGTTAEFDY | |
| IGHV3-9 (Human) | IGHJ3 (Human) | AKGRPLDRGRSWYDEIQQGDDFDI | |
| IGHV1-46 (Human) | IGHJ1 (Human) | ARPPRNYYDRSGYYQRAEYFQH | |
| IGHV3-30 (Human) | IGHJ4 (Human) | ARGLGLRFLEWPISSY | |
| IGHV3-66 (Human) | IGHJ6 (Human) | ARGDGSDDYYYGMDV | |
| IGHV3-66 (Human) | IGHJ6 (Human) | ARGDGSDDYYYGMDV | |
| IGHV3-11 (Human) | IGHJ3 (Human) | ARGVTSYSDNSGNYRTDDAFDI | |
| IGHV3-9 (Human) | IGHJ3 (Human) | AKDFSRDGDAFDI | |
| IGHV3-15 (Human) | IGHJ4 (Human) | TTGALVESRYFDWFTGYYFDY | |
| IGHV3-21 (Human) | IGHJ3 (Human) | ARDRAYRLGELSSLWGDDAFDI | |
| IGHV5-51 (Human) | IGHJ4 (Human) | TSGSYYGTLD | |
| IGHV3-66 (Human) | IGHJ4 (Human) | ARFLPTYDYFDY | |
| IGHV5-51 (Human) | IGHJ5 (Human) | VYGSGSPSNWFHP | |
| IGHV1-58 (Human) | IGHJ4 (Human) | AAPHCSGGTCYDGFDI | |
| IGHV1-69 (Human) | IGHJ6 (Human) | AKVEVNIGMDDYYYYYGMDV | |
| IGHV4-34 (Human) | IGHJ6 (Human) | ARGWDLDYYYGMDV | |
| IGHV3-66 (Human) | IGHJ3 (Human) | ARDLAVAGAFDI | |
| IGHV1-58 (Human) | IGHJ3 (Human) | AAPYCSSTSCRDGFDI | |
| IGHV4-30 (Human) | IGHJ4 (Human) | ARWKRWLQFLYFDY | |
| IGHV4-30 (Human) | IGHJ4 (Human) | ARWKRLLQSLHFDY | |
| IGHV3-66 (Human) | IGHJ4 (Human) | ARDIAGRLDY | |
| IGHV3-30 (Human) | IGHJ3 (Human) | AKGPRFGWSYRGGSGFDI | |
| IGHV1-46 (Human) | IGHJ6 (Human) | ARADTPIVVDTTSYFYYMDV | |
| IGHV1-69 (Human) | IGHJ5 (Human) | ATPLNDYYASGNLGL | |
| IGHV1-69 (Human) | IGHJ4 (Human) | ATPLNDYYASGNLGLW | |
| IGHV3-53 (Human) | IGHJ4 (Human) | ARVVGYDFWSGYDGGYFDY | |
| IGHV3-53 (Human) | IGHJ4 (Human) | ARLVGYDFRSGSDGGYFDY | |
| IGHV3-30 (Human) | IGHJ4 (Human) | AKKGQPYCGGDCYFYYFDY | |
| IGHV3-30 (Human) | IGHJ4 (Human) | AKKGQPYCGGDCHFYYLDY | |
| IGHV1-69 (Human) | IGHJ5 (Human) | AKVSLTLPIAAAPRFWFDS | |
| IGHV1-69 (Human) | IGHJ5 (Human) | ARASLTLPIRAAPRFWFDA | |
| IGHV4-31 (Human) | IGHJ6 (Human) | AREKIEVVSIEMRPHYYGIDV | |
| IGHV4-4 (Human) | IGHJ5 (Human) | AGSYSNYIGGVWFDP | |
| IGHV4-4 (Human) | IGHJ4 (Human) | ARGGDLAMGPEYLDF | |
| IGHV3-33 (Human) | IGHJ6 (Human) | ARVYGGLPYYYAIDV | |
| IGHV3-30 (Human) | IGHJ4 (Human) | ARSFSIRIGHKDN | |
| IGHV1-46 (Human) | IGHJ4 (Human) | ARSVFPVPAAGGCDY | |
| IGHV1-46 (Human) | IGHJ4 (Human) | ARDAVGVPAINSLEY | |
| IGHV1-46 (Human) | IGHJ5 (Human) | ARGGFIPMVRGFIDH | |
| IGHV1-46 (Human) | IGHJ5 (Human) | AKGSYIPAMRSSFDP | |
| IGHV3-30 (Human) | IGHJ4 (Human) | ARTFSIRIGHHDY | |
| IGHV3-9 (Human) | IGHJ4 (Human) | AKGVDYSSSSNFDF | |
| IGHV3-30 (Human) | IGHJ4 (Human) | ARDALTSISVLFDC | |
| IGHV3-66 (Human) | IGHJ5 (Human) | ARDTFGRGDDH | |
| IGHV3-30 (Human) | IGHJ6 (Human) | AKDTPGGDDIMTGWGLYGMDV | |
| IGHV3-33 (Human) | IGHJ4 (Human) | ARDSNVDTVMVTWFDY | |
| IGHV3-66 (Human) | IGHJ6 (Human) | ARDLIAFGMDV | |
| IGHV4-39 (Human) | IGHJ4 (Human) | ARQHRFGSGSSELL | |
| IGHV3-15 (Human) | IGHJ3 (Human) | TTDCFWRLGGTTCYEHDAFDV | |
| IGHV1-69 (Human) | IGHJ6 (Human) | AREDFILESAPIRENSYYYYGMDV | |
| IGHV4-39 (Human) | IGHJ3 (Human) | ARVDSSGWYTGDVFDV | |
| IGHV3-53 (Human) | IGHJ6 (Human) | ARESYGMDV | |
| IGHV3-53 (Human) | IGHJ6 (Human) | ARESYGMDV | |
| IGHV1-46 (Human) | IGHJ4 (Human) | AREGVGGTSYFDY | |
| IGHV1-46 (Human) | IGHJ4 (Human) | AREGLGATAYFDY | |
| IGHV3-33 (Human) | IGHJ4 (Human) | ARDPAITEAEIDY | |
| IGHV1-46 (Human) | IGHJ6 (Human) | ARDIVFVPATMAMDV | |
| IGHV1-46 (Human) | IGHJ6 (Human) | SRDIVFVPATMAMDV | |
| IGHV3-9 (Human) | IGHJ6 (Human) | AKDLLGNYYYYTLDV | |
| IGHV4-61 (Human) | IGHJ2 (Human) | ARDIPPTWYFDL | |
| IGHV4-61 (Human) | IGHJ2 (Human) | ARDIPSTWYFDLW | |
| IGHV5-51 (Human) | IGHJ4 (Human) | ARMVTSGTYYYDNSGYSSSGPFDY | |
| IGHV5-51 (Human) | IGHJ4 (Human) | AKMVTSGTSYYETRGYASSGPFDN | |
| IGHV5-51 (Human) | IGHJ4 (Human) | ARRGASWELDY | |
| IGHV5-51 (Human) | IGHJ4 (Human) | ARRGSSWEVDY | |
| IGHV3-9 (Human) | IGHJ3 (Human) | AKAASRSTRIGGAFDI | |
| IGHV3-9 (Human) | IGHJ3 (Human) | AKAASRSTRIGGAFDI | |
| IGHV1-24 (Human) | IGHJ4 (Human) | ATRGRYCSSGNCYYHH | |
| IGHV3-23 (Human) | IGHJ4 (Human) | AKDVPIEQQLVPTFDY | |
| IGHV3-23 (Human) | IGHJ4 (Human) | ARDVPVEQQLVPTFDY | |
| IGHV3-30 (Human) | IGHJ3 (Human) | AKALSSTYYYDASGPDAFDI | |
| IGHV3-30 (Human) | IGHJ3 (Human) | AKALSSTFYFDASGPDAFDI | |
| IGHV1-69 (Human) | IGHJ4 (Human) | ARAHMYCSDGSCYRQSGYFDS | |
| IGHV3-66 (Human) | IGHJ3 (Human) | ARDFREGAFDI | |
| IGHV3-66 (Human) | IGHJ3 (Human) | ARDFREGAIDL | |
| IGHV3-66 (Human) | IGHJ4 (Human) | ARSYGDYYIDY | |
| IGHV3-13 (Human) | IGHJ4 (Human) | ARGGLQTTTWLFDY | |
| IGHV3-13 (Human) | IGHJ4 (Human) | ARGGLQTTTWLFDN | |
| IGHV5-51 (Human) | IGHJ4 (Human) | ARTWSPAAVAFFDS | |
| IGHV3-53 (Human) | IGHJ4 (Human) | ARGLPTGEGWNYFDY | |
| IGHV3-9 (Human) | IGHJ4 (Human) | AKGWFGELLGGSDS | |
| IGHV1-46 (Human) | IGHJ5 (Human) | ARGGIVPHLSNWFDP | |
| IGHV3-30 (Human) | IGHJ1 (Human) | ARGLLWFGESEYFQH | |
| IGHV3-11 (Human) | IGHJ6 (Human) | AREGIAAPDSKADAFDI | |
| IGHV3-9 (Human) | IGHJ4 (Human) | AKAGVRNIAAAGPDLNFDF | |
| IGHV4-4 (Human) | IGHJ4 (Human) | ARGSALNWKSIGYFDS | |
| IGHV3-30 (Human) | IGHJ4 (Human) | ARDGSVDTLMVTWFDY | |
| IGHV3-48 (Human) | IGHJ5 (Human) | AREAHDGALTGYGDYLNWFDP | |
| IGHV3-30 (Human) | IGHJ4 (Human) | ARDGTIVTLVRGVMGPPFDY | |
| IGHV3-30 (Human) | IGHJ4 (Human) | AKQIGEYCSGGNCYQGSLDY | |
| IGHV3-15 (Human) | IGHJ3 (Human) | TTGVVVVVSSSPDDAFDV | |
| IGHV4-31 (Human) | IGHJ4 (Human) | ATGGLSAFGELFPHDK | |
| IGHV3-30 (Human) | IGHJ4 (Human) | ARDQGTATTYFDH | |
| IGHV3-30 (Human) | IGHJ4 (Human) | ARDYGDYVTHFDY | |
| IGHV4-30 (Human) | IGHJ4 (Human) | ARVVRLWPRYFDS | |
| IGHV1-2 (Human) | IGHJ4 (Human) | ARTSSPHSSSTGDFDS | |
| IGHV4-61 (Human) | IGHJ4 (Human) | ARETYYYDRSGYYSSDGFDY | |
| IGHV4-39 (Human) | IGHJ6 (Human) | ARRGGRTPVRFNYGGDV | |
| IGHV1-2 (Human) | IGHJ3 (Human) | ARGGQDELTGTFDV | |
| IGHV4-31 (Human) | IGHJ4 (Human) | ARGSYSDYNGGWDY | |
| IGHV3-53 (Human) | IGHJ4 (Human) | ARDLSSSGGFDY | |
| IGHV3-30 (Human) | IGHJ4 (Human) | ARDATMITLVRGIMGPPFDH | |
| IGHV3-30 (Human) | IGHJ4 (Human) | AKQIGEYCSGGSCYQGSLDY | |
| IGHV4-31 (Human) | IGHJ4 (Human) | ASGELSAFGELFPHDY | |
| IGHV3-30 (Human) | IGHJ4 (Human) | ARDQGMATTYFDY | |
| IGHV1-2 (Human) | IGHJ4 (Human) | ARTSSPHSSSTGDLDY | |
| IGHV1-2 (Human) | IGHJ3 (Human) | ARGGQDELTGAFDI | |
| IGHV4-31 (Human) | IGHJ4 (Human) | ARGSYSNYNGGLDY | |
| IGHV4-30 (Human) | IGHJ3 (Human) | ARLNTMIVMINGVFDV | |
| IGHV3-23 (Human) | IGHJ6 (Human) | VKESDYYMASVNGMDV | |
| IGHV1-58 (Human) | IGHJ3 (Human) | AAPQCNRTTCYDAFDM | |
| IGHV3-23 (Human) | IGHJ4 (Human) | AKGPRSNYDYFES | |
| IGHV3-9 (Human) | IGHJ4 (Human) | GKQINEWSHFLDY | |
| IGHV4-31 (Human) | IGHJ3 (Human) | ASTPYTNGGAFHI | |
| IGHV4-61 (Human) | IGHJ3 (Human) | ARETFFYDRTGHYKSDGFDV | |
| IGHV3-33 (Human) | IGHJ4 (Human) | AKGQLRLGEFDDY | |
| IGHV3-48 (Human) | IGHJ6 (Human) | ARAIVGTKGYMDV | |
| IGHV3-53 (Human) | IGHJ4 (Human) | AREGDVEGISDSWSGYSRDRYYFDH | |
| IGHV3-53 (Human) | IGHJ4 (Human) | AREGDVDGNYGFWSGYSRDRYYFDY | |
| IGHV1-2 (Human) | IGHJ3 (Human) | ARGPLFHKVVYESSSGFHDGLDF | |
| IGHV3-21 (Human) | IGHJ4 (Human) | ARGPLFHKVVYESSSGFHDGLDF | |
| IGHV1-46 (Human) | IGHJ5 (Human) | ARASTSTSSWSEALSLGS | |
| IGHV3-53 (Human) | IGHJ4 (Human) | VRDFGEFYFDY | |
| IGHV3-53 (Human) | IGHJ6 (Human) | ARDLIVLGVDV | |
| IGHV3-64 (Human) | IGHJ4 (Human) | VKDKEHSTMVTIFDF | |
| IGHV4-31 (Human) | IGHJ4 (Human) | ARVKGWLRGYFDH | |
| IGHV4-34 (Human) | IGHJ4 (Human) | ARESGSYGTFDY | |
| IGHV3-53 (Human) | IGHJ4 (Human) | ARLGGVFNGFNGSFDY | |
| IGHV3-33 (Human) | IGHJ4 (Human) | ARDPMIVVVEMDY | |
| IGHV4-34 (Human) | IGHJ4 (Human) | ARETGTYGTFDH | |
| IGHV4-31 (Human) | IGHJ4 (Human) | ARVKGWLRGYFDY | |
| IGHV3-48 (Human) | IGHJ4 (Human) | ARGDCTSSSCYSLDY | |
| IGHV3-48 (Human) | IGHJ4 (Human) | ARGDCLSSSCYSLDY | |
| IGHV3-66 (Human) | IGHJ4 (Human) | ARDLRDQDGYSYGAFDY | |
| IGHV3-66 (Human) | IGHJ4 (Human) | ARDLRKDDGYSYGAFDY | |
| IGHV1-69 (Human) | IGHJ4 (Human) | TINTQWDLVPR | |
| IGHV3-15 (Human) | IGHJ4 (Human) | TSQLWLRGPGDY | |
| IGHV5-51 (Human) | IGHJ3 (Human) | ARGGWDPAEYSSSGGGGLDAFDI | |
| IGHV5-10 (Human) | IGHJ6 (Human) | GRIAPPGRGSYYPTQNYMDV | |
| IGHV1-69 (Human) | IGHJ6 (Human) | ARPEGCGSRTSCTPGAYYYGMDV | |
| IGHV3-30 (Human) | IGHJ4 (Human) | AKAGGPYYYDTSGSFWYFDY | |
| IGHV3-30 (Human) | IGHJ4 (Human) | AKGGGQYCSHGNCYLNYFDY | |
| IGHV1-69 (Human) | IGHJ4 (Human) | ARRDCSTTSCYDEVLYRLVD | |
| IGHV3-15 (Human) | IGHJ4 (Human) | TTDADYSDSSGYYVTYYFEY | |
| IGHV4-4 (Human) | IGHJ4 (Human) | ARANGILDF | |
| IGHV3-30 (Human) | IGHJ6 (Human) | ARDFESRTWDPPKYYYALDV | |
| IGHV5-10 (Human) | IGHJ5 (Human) | ARLSWSPPTRTTDEKNWFDP | |
| IGHV3-53 (Human) | IGHJ4 (Human) | ARDYGDFYFDY | |
| IGHV4-34 (Human) | IGHJ3 (Human) | ARKPLLHSNISPGAFDI | |
| IGHV4-31 (Human) | IGHJ5 (Human) | ARATVVITLHWFDP | |
| IGHV3-49 (Human) | IGHJ4 (Human) | TRWDGWSQHDY | |
| IGHV4-59 (Human) | IGHJ6 (Human) | ASYYNDTSGYSYGLDV | |
| IGHV5-51 (Human) | IGHJ4 (Human) | ARGGPPGGVKLELTDY | |
| IGHV1-8 (Human) | IGHJ4 (Human) | ARGRANWNSNFLLDS | |
| IGHV1-8 (Human) | IGHJ4 (Human) | ARGRANYNSKFLLDN | |
| IGHV3-30 (Human) | IGHJ4 (Human) | ARDLENVLIEVALQD | |
| IGHV3-30 (Human) | IGHJ4 (Human) | ARDLENVMIEVALES | |
| IGHV3-43 (Human) | IGHJ4 (Human) | AKDSEDCSSTSCYVDH | |
| IGHV4-30 (Human) | IGHJ4 (Human) | ARAMITFGGVIVVLDY | |
| IGHV4-30 (Human) | IGHJ4 (Human) | ARAMITFGGVIVLYDY | |
| IGHV3-13 (Human) | IGHJ4 (Human) | ARVRYDSSGYFWSLDY | |
| IGHV3-13 (Human) | IGHJ4 (Human) | ARVRFDTSGYFWSLDY | |
| IGHV3-11 (Human) | IGHJ3 (Human) | ARVPPPQRLHPFDV | |
| IGHV3-11 (Human) | IGHJ3 (Human) | ARVPPPQRLHPFDV | |
| IGHV4-39 (Human) | IGHJ4 (Human) | ARHFADGSGRVVDS | |
| IGHV4-39 (Human) | IGHJ4 (Human) | ARHFADGSGRVVDY | |
| IGHV3-11 (Human) | IGHJ4 (Human) | ARDGGGYDRFDH | |
| IGHV3-11 (Human) | IGHJ4 (Human) | ARDGGAYDRFDY | |
| IGHV3-30 (Human) | IGHJ5 (Human) | AKSWWLSENWFDP | |
| IGHV3-30 (Human) | IGHJ5 (Human) | AKSWWLSENWFDP | |
| IGHV3-30 (Human) | IGHJ4 (Human) | ARDKRGVIRGLLNF | |
| IGHV3-30 (Human) | IGHJ4 (Human) | AKPVDAAMFDF | |
| IGHV3-30 (Human) | IGHJ4 (Human) | AKPVDTAMFDS | |
| IGHV3-53 (Human) | IGHJ6 (Human) | ARDLVVWGMDV | |
| IGHV3-53 (Human) | IGHJ6 (Human) | ARDLVVWGMDV | |
| IGHV3-30 (Human) | IGHJ6 (Human) | AKVVVRGVIISLYYGMDV | |
| IGHV3-30 (Human) | IGHJ6 (Human) | AKVALRGVFISLYYGMDV | |
| IGHV1-2 (Human) | IGHJ6 (Human) | ARDVIVSMVRGVIFRMDV | |
| IGHV1-2 (Human) | IGHJ6 (Human) | ARDVIITMGRGVVFRMDV | |
| IGHV3-33 (Human) | IGHJ3 (Human) | ARDFSNSDMVTLSDAFDI | |
| IGHV3-53 (Human) | IGHJ4 (Human) | ARDLGTGLFDY | |
| IGHV3-53 (Human) | IGHJ4 (Human) | ARDLGTGLFDY | |
| IGHV3-30 (Human) | IGHJ6 (Human) | ASGLLWFETREISGAPDYGMAV | |
| IGHV3-30 (Human) | IGHJ6 (Human) | ASGLLWFETAGGSGAPDYGMAV | |
| IGHV4-30 (Human) | IGHJ3 (Human) | ARFCLSGSHYLFAFDI | |
| IGHV1-69 (Human) | IGHJ4 (Human) | ARNRGYSDYGSVYYFDY | |
| IGHV3-30 (Human) | IGHJ4 (Human) | ARDGTTMTPTDLLTD | |
| IGHV3-30 (Human) | IGHJ4 (Human) | AKDIGGGSSPPFFDY | |
| IGHV3-30 (Human) | IGHJ6 (Human) | AKGSGSQLYYYYGMDV | |
| IGHV1-46 (Human) | IGHJ6 (Human) | ARDYVLVPARSGMDV | |
| IGHV1-46 (Human) | IGHJ6 (Human) | ARDLVFVPATSAMDV | |
| IGHV4-4 (Human) | IGHJ3 (Human) | ARSSRFLPPLPDAFDL | |
| IGHV3-13 (Human) | IGHJ5 (Human) | ARANYDSSGYHNWFDP | |
| IGHV4-30 (Human) | IGHJ4 (Human) | ARVVRVLPAASVDC | |
| IGHV3-11 (Human) | IGHJ6 (Human) | ARRGDGNVPLFHYYYMDV | |
| IGHV4-4 (Human) | IGHJ3 (Human) | ARDGGRPGDPFDI | |
| IGHV5-51 (Human) | IGHJ2 (Human) | VRGIAVDWYFDL | |
| IGHV1-69 (Human) | IGHJ6 (Human) | ARDSEYSSSWYSRGYYGMDV | |
| IGHV1-69 (Human) | IGHJ6 (Human) | QSYDTSLSGSRV | |
| IGHV4-34 (Human) | IGHJ4 (Human) | ARAGFGFVITSRSGTDPLFDY | |
| IGHV3-49 (Human) | IGHJ4 (Human) | TRAWIPTPHDY | |
| IGHV3-49 (Human) | IGHJ4 (Human) | SRAWIPTPHDY | |
| IGHV5-51 (Human) | IGHJ4 (Human) | ARRGSSWEIDH | |
| IGHV4-59 (Human) | IGHJ5 (Human) | ARHYDILTALSWFDP | |
| IGHV3-30 (Human) | IGHJ4 (Human) | AKSPINYCANGVCYPDS | |
| IGHV3-66 (Human) | IGHJ6 (Human) | TRLGGYRYGMDV | |
| IGHV3-66 (Human) | IGHJ6 (Human) | ARLGGYRYGMDV | |
| IGHV1-69 (Human) | IGHJ2 (Human) | ARGVGYSGSGSNWYFDL | |
| IGHV3-9 (Human) | IGHJ3 (Human) | AKAVPTSCYVFCALDI | |
| IGHV3-49 (Human) | IGHJ4 (Human) | TRLRQVQGVPGYYFDQ | |
| IGHV3-13 (Human) | IGHJ4 (Human) | ARASGVLTTHFDS | |
| IGHV3-33 (Human) | IGHJ3 (Human) | AREGVALAGNGVDGFDI | |
| IGHV3-33 (Human) | IGHJ3 (Human) | AREGVAVGGNGVDGFDM | |
| IGHV4-59 (Human) | IGHJ5 (Human) | ARLLSTEWLFNWFDP | |
| IGHV4-59 (Human) | IGHJ5 (Human) | ARLLSTEWSFNWFDP | |
| IGHV3-15 (Human) | IGHJ3 (Human) | TTVDVQGIWELLENDAFDI | |
| IGHV3-66 (Human) | IGHJ4 (Human) | ARDLGDSRLDY | |
| IGHV3-49 (Human) | IGHJ4 (Human) | TRRAHYSGSGLSSYVDY | |
| IGHV3-13 (Human) | IGHJ2 (Human) | ARTEYAWGSYRSYWYFDL | |
| IGHV3-13 (Human) | IGHJ2 (Human) | ARTEYAWGSYRSYWYFDL | |
| IGHV3-53 (Human) | IGHJ4 (Human) | ARLTMTTYYFDS | |
| IGHV3-13 (Human) | IGHJ4 (Human) | ARGDSSGLYTFFDY | |
| IGHV1-69 (Human) | IGHJ4 (Human) | ATGKGYSSSSAAYYFDH | |
| IGHV3-30 (Human) | IGHJ6 (Human) | AKEGNGYGYQYAGMDV | |
| IGHV1-69 (Human) | IGHJ5 (Human) | ARGGIAVGGWWFDP | |
| IGHV3-13 (Human) | IGHJ2 (Human) | ARGGSSSWLWYFDL | |
| IGHV3-13 (Human) | IGHJ6 (Human) | VRGDTLVQGVIKAYYYYFMDV | |
| IGHV3-30 (Human) | IGHJ4 (Human) | AKVLGSYCSASSCHGQRPDY | |
| IGHV1-2 (Human) | IGHJ6 (Human) | AREPIEGVIGGMIVNYYYMDV | |
| IGHV1-2 (Human) | IGHJ6 (Human) | AREPIEAVPAGIIVNYYYMDV | |
| IGHV5-51 (Human) | IGHJ2 (Human) | VRGLPVDWYFDL | |
| IGHV4-59 (Human) | IGHJ5 (Human) | ARMLSTEWSFNWFDP | |
| IGHV1-69 (Human) | IGHJ6 (Human) | ARETGYSGFLAVAYMDV | |
| IGHV3-66 (Human) | IGHJ6 (Human) | ANQGYYYYMDV | |
| IGHV4-31 (Human) | IGHJ4 (Human) | ARGDTFGRGYYFDY | |
| IGHV4-31 (Human) | IGHJ4 (Human) | ARGDTFGRGYYFDF | |
| IGHV3-23 (Human) | IGHJ3 (Human) | AKSDHGDYVIGAFDI | |
| IGHV4-4 (Human) | IGHJ4 (Human) | ARAQTPEFGELLY | |
| IGHV3-49 (Human) | IGHJ4 (Human) | ARNDRYIVIVPAEMLY | |
| IGHV3-13 (Human) | IGHJ3 (Human) | ARGQRGYYDRSGYYWGWRAFDI | |
| IGHV3-23 (Human) | IGHJ6 (Human) | ANHPLGAAEGYYYYYMDV | |
| IGHV1-46 (Human) | IGHJ3 (Human) | ARGGAIPALRTAFDI | |
| IGHV1-46 (Human) | IGHJ3 (Human) | ARGGAIPALRSAFDI | |
| IGHV3-7 (Human) | IGHJ4 (Human) | ARVWWLRGSFDY | |
| IGHV3-7 (Human) | IGHJ4 (Human) | ALSSGYSGYAGNY | |
| IGHV3-15 (Human) | IGHJ4 (Human) | TTGSETYYYDSSGPFDY | |
| IGHV1-46 (Human) | IGHJ4 (Human) | ARANEGAAVSFDY | |
| IGHV3-64D (Human) | IGHJ6 (Human) | VKDQGGRGWPSYYYYHYMDV | |
| IGHV1-18 (Human) | IGHJ6 (Human) | ARVPASYGDDDYYYYYGMDV | |
| IGHV3-23 (Human) | IGHJ4 (Human) | AKVGEYCGDDCYRGLDY | |
| IGHV3-23 (Human) | IGHJ4 (Human) | AKVGEYCGGDCYRGLDY | |
| IGHV3-66 (Human) | IGHJ6 (Human) | ARIANYMDV | |
| IGHV3-66 (Human) | IGHJ6 (Human) | ARIANYMDV | |
| IGHV4-59 (Human) | IGHJ4 (Human) | ARYQLAPGSGSYYNWGGYPRESEYYFDY | |
| IGHV4-59 (Human) | IGHJ4 (Human) | ARYQLAPGSGSYYNWGGYPRESEYYFDY | |
| IGHV3-30 (Human) | IGHJ6 (Human) | AKTYSGYGYYYYGMDV | |
| IGHV3-48 (Human) | IGHJ4 (Human) | SRGDCTSSSCYSIDF | |
| IGHV3-53 (Human) | IGHJ4 (Human) | ARGPYCGGDCDY | |
| IGHV3-53 (Human) | IGHJ6 (Human) | ARETYGMDV | |
| IGHV4-39 (Human) | IGHJ5 (Human) | ARQARTDASTYGHNFDS | |
| IGHV4-39 (Human) | IGHJ4 (Human) | ARQARTDGSTYGHNFDY | |
| IGHV1-2 (Human) | IGHJ6 (Human) | ARESHGGVWSAPGYYYGMDV | |
| IGHV3-21 (Human) | IGHJ4 (Human) | ARDDGIAAASDY | |
| IGHV3-21 (Human) | IGHJ4 (Human) | ARDDGIAAASDY | |
| IGHV4-34 (Human) | IGHJ4 (Human) | ARGHTQEKWELREGYYFDY | |
| IGHV3-21 (Human) | IGHJ5 (Human) | ARERGYHGGKTSPFL | |
| IGHV1-2 (Human) | IGHJ4 (Human) | ARPPRDYYDSSGYQIRDDHFDY | |
| IGHV1-8 (Human) | IGHJ6 (Human) | ARFPKVPAAIFPGDYYYGMDV | |
| IGHV3-33 (Human) | IGHJ4 (Human) | ARDEGSLTTTFDY | |
| IGHV3-7 (Human) | IGHJ4 (Human) | ARDTIPFWSGYYTSPDYYFDY | |
| IGHV4-31 (Human) | IGHJ4 (Human) | ASSRYDFWSGSFDY | |
| IGHV3-23 (Human) | IGHJ4 (Human) | ARRGNSGSYPDPDY | |
| IGHV1-2 (Human) | IGHJ3 (Human) | ARGPLPHRLVYDFWSGFHDAFDI | |
| IGHV3-15 (Human) | IGHJ4 (Human) | YRFCSDGSCYRHFDY | |
| IGHV3-21 (Human) | IGHJ4 (Human) | ARECSDESFDY | |
| IGHV3-33 (Human) | IGHJ3 (Human) | AREGDGPDAFDI | |
| IGHV3-53 (Human) | IGHJ5 (Human) | ARVGDWYFS | |
| IGHV4-4 (Human) | IGHJ6 (Human) | ARGLFYNSGGYYSSNYYYYIDV | |
| IGHV5-10-1 (Human) | IGHJ4 (Human) | ARLNTDLRSRFGELYYYFDY | |
| IGHV1-46 (Human) | IGHJ4 (Human) | ARVGRGFSYGYFDY | |
| IGHV1-69 (Human) | IGHJ6 (Human) | ARVGAEWPRDHKYYYYGMDV | |
| IGHV3-13 (Human) | IGHJ4 (Human) | ARVKYGGYVGYFDY | |
| IGHV3-15 (Human) | IGHJ4 (Human) | ATHRSEAGDLDY | |
| IGHV3-66 (Human) | IGHJ4 (Human) | ARETLGRGGDY | |
| IGHV3-9 (Human) | IGHJ3 (Human) | AKIADIVRAYDFWSGQHFDAFDI | |
| IGHV3-9 (Human) | IGHJ6 (Human) | AKDKGSGSGYGMDV | |
| IGHV3-9 (Human) | IGHJ6 (Human) | AKDQTKDGGGAYSYGLGGMDV | |
| IGHV4-39 (Human) | IGHJ5 (Human) | ATGGRFWGWFDP | |
| IGHV5-51 (Human) | IGHJ5 (Human) | ARRVTIPNGWFDP | |
| IGHV1-69 (Human) | IGHJ6 (Human) | ARNRAVSEREDYYYGMDV | |
| IGHV3-66 (Human) | IGHJ4 (Human) | ARDLRDQDGYSYGAFDY | |
| IGHV1-2 (Human) | IGHJ3 (Human) | ARGPLFHRLVYDFWSGYHDGFDM | |
| IGHV1-18 (Human) | IGHJ6 (Human) | ARVPASYGDDDYYYYYGMDV | |
| IGHV3-53 (Human) | IGHJ6 (Human) | ARESYGMDV | |
| IGHV4-34 (Human) | IGHJ4 (Human) | ARGHTQEKWELREGYYFDY | |
| IGHV3-30 (Human) | IGHJ4 (Human) | AKEGRPSDIVVVVAFDY | |
| IGHV1-58 (Human) | IGHJ3 (Human) | AAPHCSGGSCYDAFDI | |
| IGHV1-58 (Human) | IGHJ3 (Human) | AAPHCSGGSCYDAFDI | |
| IGHV3-53 (Human) | IGHJ4 (Human) | AREGDVEGYYDFWSGYSRDRYYFDY | |
| IGHV1-2 (Human) | IGHJ3 (Human) | ARDLGWSRLHGAFDI | |
| IGHV4-4 (Human) | IGHJ4 (Human) | ARGLSYYPLYGSHINYIDY | |
| IGHV3-53 (Human) | IGHJ3 (Human) | ARGGWYYDSSGYYSGRTDAFDI | |
| IGHV4-4 (Human) | IGHJ4 (Human) | ARGLSYYPLYGSHINYIDY | |
| IGHV3-66 (Human) | IGHJ4 (Human) | ARDLRDQDGYSYGAFDY | |
| IGHV4-4 (Human) | IGHJ4 (Human) | ATHLRLGELRGVDY | |
| IGHV3-21 (Human) | IGHJ4 (Human) | ARERGYYGGKTPPFL | |
| IGHV1-69 (Human) | IGHJ6 (Human) | ARGNRLLYCSSTSCYLDAVRQGYYYYYYMDV | |
| IGHV1-2 (Human) | IGHJ4 (Human) | ARAPPFYDFWSGIDY | |
| IGHV1-69 (Human) | IGHJ5 (Human) | ARDLRNCSSTSCYYWFDP | |
| IGHV1-69 (Human)+K3788 | IGHJ6 (Human) | ARGNRLLYCSSTSCYLDAVRQGYYYYYYMDV | |
| IGHV1-2 (Human) | IGHJ6 (Human) | ASPASRGYSGYDHGYYYYMDV | |
| IGHV1-2 (Human) | IGHJ6 (Human) | ARDDTLLRYSDWLPTTSFGGMDV | |
| IGHV3-33 (Human) | IGHJ4 (Human) | AREDYYDSSGSLDY | |
| IGHV6-1 (Human) | IGHJ4 (Human) | ARDPSSHFDY | |
| IGHV3-53 (Human) | IGHJ3 (Human) | ARGGWYYDSSGYYSGRTDAFDI | |
| IGHV3-66 (Human) | IGHJ6 (Human) | HRGSYSTFSYYYYMDV | |
| IGHV1-58 (Human) | IGHJ3 (Human) | AAPHCSGGSCYDAFDI | |
| IGHV1-2 (Human) | IGHJ3 (Human) | AREALGYGDFPHDGFDL | |
| IGHV3-30-3 (Human) | IGHJ4 (Human) | ARGPRGYYDFWSGYENEYYFDY | |
| IGHV4-59 (Human) | IGHJ4 (Human) | ARYQLAPGSGSYYNWGGYPRESEYYFDY | |
| IGHV3-30-3 (Human) | IGHJ4 (Human) | AREGAGPYYYDSSGYYTLFDD | |
| IGHV1-46 (Human) | IGHJ6 (Human) | ARVGCGGDNCYFWRPLGNGMDV | |
| IGHV3-66 (Human) | IGHJ6 (Human) | HRGSYSTFSYYYYMDV | |
| IGHV4-59 (Human) | IGHJ4 (Human) | ARGFDY | |
| IGHV4-59 (Human) | IGHJ6 (Human) | ARGPAATYYYYMDV | |
| IGHV3-9 (Human) | IGHJ3 (Human) | AKAEPEVGGYDYYMDV | |
| IGHV4-59 (Human) | IGHJ5 (Human) | ARHCPWQQLVSNWFDP | |
| IGHV3-33 (Human) | IGHJ6 (Human) | VRGDHSSGWYGTYYYYMDV | |
| IGHV4-34 (Human) | IGHJ4 (Human) | ARHRRDYITMIVRPTRLWAFDY | |
| **Set2: 1,147 SARS-CoV2 neutralizing antibody sequences** | | |  |
| **IGHV gene** | **IGHJ gene** | **CDRH3** |  |
| IGHV3-23 (Human) | IGHJ4 (Human) | VSNWASGSTGDY |  |
| IGHV3-23 (Human) | IGHJ4 (Human) | VSNWASGSTGDY |  |
| IGHV1-2 (Human) | IGHJ5 (Human) | ATDGDDGENWFDP |  |
| IGHV1-24 (Human) | IGHJ6 (Human) | ATAGAITGTPRNFYYYYGMDV |  |
| IGHV1-24 (Human) | IGHJ5 (Human) | VTSQPFVSPNWFDP |  |
| IGHV1-24 (Human) | IGHJ5 (Human) | ATSPAVVERGWFDP |  |
| IGHV1-24 (Human) | IGHJ6 (Human) | ATAGAITGTPTNYSYYYGMDF |  |
| IGHV1-24 (Human) | IGHJ4 (Human) | TTGPAVTNRPADY |  |
| IGHV1-24 (Human) | IGHJ4 (Human) | ATTPAFMAAAGDFDY |  |
| IGHV3-53 (Human) | IGHJ4 (Human) | ASFLVGATGQPWY |  |
| IGHV3-30-3 (Human) | IGHJ4 (Human) | ARDGVSVTMVRGVIGPLCDY |  |
| IGHV3-30 (Human) | IGHJ1 (Human) | AKDSPYYYDSSGYYPGYFQD |  |
| IGHV3-48 (Human) | IGHJ4 (Human) | ARVARRDVAPATIAAYFLDY |  |
| IGHV4-39 (Human) | IGHJ5 (Human) | ARERPPFDVVVVPAARPYNWFDP |  |
| IGHV1-69 (Human) | IGHJ4 (Human) | AKVADRVGASTGELDY |  |
| IGHV3-33 (Human) | IGHJ3 (Human) | ARGPLGSNYFNYDAFDI |  |
| IGHV3-53 (Human) | IGHJ6 (Human) | ARDLSEKGGMDV |  |
| IGHV3-66 (Human) | IGHJ4 (Human) | ARGPYPRGFDY |  |
| IGHV3-53 (Human) | IGHJ4 (Human) | ARDYGDFYFDY |  |
| IGHV3-66 (Human) | IGHJ4 (Human) | ARSYGDFYMDY |  |
| IGHV1-69 (Human) | IGHJ4 (Human) | ASRRLSTDYDRHY |  |
| IGHV3-30 (Human) | IGHJ4 (Human) | VSLITMIRGVPDC |  |
| IGHV3-66 (Human) | IGHJ3 (Human) | ARETYAFDI |  |
| IGHV3-9 (Human) | IGHJ6 (Human) | AKDIMVWYGLSRDGYNFAYYGMDV |  |
| IGHV3-53 (Human) | IGHJ4 (Human) | ARDYGDFYFDY |  |
| IGHV1-18 (Human) | IGHJ6 (Human) | ARASGFMVRGVNPNYYYYMDV |  |
| IGHV3-53 (Human) | IGHJ6 (Human) | ARYMGDGMDV |  |
| IGHV3-53 (Human) | IGHJ6 (Human) | ARDLSYYGMDV |  |
| IGHV3-53 (Human) | IGHJ3 (Human) | ARDLELKGAFDI |  |
| IGHV3-53 (Human) | IGHJ4 (Human) | ARGYGDLYFDY |  |
| IGHV3-53 (Human) | IGHJ3 (Human) | ARDLELKGAFDI |  |
| IGHV3-53 (Human) | IGHJ4 (Human) | ARDLEEAGGFDY |  |
| IGHV1-2 (Human) | IGHJ6 (Human) | ARNYPPLLQQLGFMDV |  |
| IGHV1-69D (Human) | IGHJ5 (Human) | ARAGYCTSTVCSRLAPRGWLDP |  |
| IGHV1-69 (Human) | IGHJ6 (Human) | ARDARGCSHDSVWGSFQDCYHGMDV |  |
| IGHV3-7 (Human) | IGHJ4 (Human) | ARDSRGHYYDSSGYPAFDY |  |
| IGHV3-9 (Human) | IGHJ6 (Human) | AKERAGPQIKTYYYYGLDV |  |
| IGHV1-69 (Human) | IGHJ6 (Human) | ARDARGCFHDDVWGSFRDCYNGMAV |  |
| IGHV3-53 (Human) | IGHJ4 (Human) | ARGYGDLYFDY |  |
| IGHV1-46 (Human) | IGHJ4 (Human) | ARASWGSTLDY |  |
| IGHV3-23 (Human) | IGHJ4 (Human) | AKVWPRSSEGYFFDL |  |
| IGHV3-9 (Human) | IGHJ6 (Human) | AKDILGNYYYYSMHV |  |
| IGHV4-59 (Human) | IGHJ4 (Human) | ASGRDESKTGY |  |
| IGHV3-53 (Human) | IGHJ4 (Human) | AREYGDWE |  |
| IGHV3-9 (Human) | IGHJ6 (Human) | AKGLAARHYYYYGMDV |  |
| IGHV3-66 (Human) | IGHJ3 (Human) | ARDLEEKGAFDI |  |
| IGHV1-2 (Human) | IGHJ5 (Human) | ARGPLVPAAIRGNWFDP |  |
| IGHV3-66 (Human) | IGHJ4 (Human) | ARGYGDYYFDY |  |
| IGHV4-59 (Human) | IGHJ4 (Human) | ASGRDESKTGY |  |
| IGHV3-53 (Human) | IGHJ4 (Human) | AREYGDWE |  |
| IGHV1-2 (Human) | IGHJ5 (Human) | ARGPLLPVAIRGNWFDP |  |
| IGHV3-23 (Human) | IGHJ3 (Human) | AKDPFYRWFNHGDAFDI |  |
| IGHV3-66 (Human) | IGHJ4 (Human) | ARGYGDYYFDY |  |
| IGHV3-53 (Human) | IGHJ6 (Human) | ARDLQQLGGMDV |  |
| IGHV3-53 (Human) | IGHJ6 (Human) | ARFGGYELGGVDV |  |
| IGHV3-53 (Human) | IGHJ4 (Human) | ARDFGGLRFDY |  |
| IGHV3-21 (Human) | IGHJ4 (Human) | ARERRSDYSSDWYDPPTTPRSFDY |  |
| IGHV3-53 (Human) | IGHJ4 (Human) | ARDRGDSLFDY |  |
| IGHV3-53 (Human) | IGHJ6 (Human) | AREVYAMDV |  |
| IGHV3-53 (Human) | IGHJ6 (Human) | ARDLQQRGGMDV |  |
| IGHV3-53 (Human) | IGHJ6 (Human) | ARDLLEAGGMDV |  |
| IGHV1-18 (Human) | IGHJ5 (Human) | ARDGELMGWFDP |  |
| IGHV3-53 (Human) | IGHJ4 (Human) | ARDFGEFYFDY |  |
| IGHV1-69D (Human) | IGHJ4 (Human) | ASCRYYYDSSGYAQPEDYFDY |  |
| IGHV3-53 (Human) | IGHJ6 (Human) | ARDIVGAIRGMDV |  |
| IGHV1-46 (Human) | IGHJ4 (Human) | ARVEPPITQEMATILPYFDY |  |
| IGHV3-53 (Human) | IGHJ4 (Human) | ARDLGPSGGIDY |  |
| IGHV3-53 (Human) | IGHJ6 (Human) | ARDLLAYGMDV |  |
| IGHV1-3 (Human) | IGHJ3 (Human) | AREPPSHSSGYYNESGGAFDI |  |
| IGHV3-53 (Human) | IGHJ6 (Human) | ARDLYVYGMDV |  |
| IGHV3-53 (Human) | IGHJ4 (Human) | ARVLPMNWYYFDY |  |
| IGHV3-30 (Human) | IGHJ6 (Human) | ARGNVDIAMARVNYFYYGMDV |  |
| IGHV1-58 (Human) | IGHJ3 (Human) | AANYCSGGSCSDAFDI |  |
| IGHV3-23 (Human) | IGHJ1 (Human) | AKGGDTAMVNAEYFQH |  |
| IGHV1-69D (Human) | IGHJ5 (Human) | ARGRAAAAPPFTWFDP |  |
| IGHV4-39 (Human) | IGHJ4 (Human) | ARLTWLRGNFDY |  |
| IGHV3-66 (Human) | IGHJ4 (Human) | ARDYGAYGRRNDF |  |
| IGHV3-30 (Human) | IGHJ3 (Human) | ARDGRTITMVQGVISGAFDI |  |
| IGHV3-30 (Human) | IGHJ4 (Human) | AKDSTPISWIQLWGTDYFDY |  |
| IGHV1-46 (Human) | IGHJ4 (Human) | AREHGGNSYFDQ |  |
| IGHV3-30 (Human) | IGHJ3 (Human) | AREPHCTGGVCDAFDM |  |
| IGHV3-30 (Human) | IGHJ3 (Human) | ASHSAGYGDSYETFDI |  |
| IGHV3-66 (Human) | IGHJ5 (Human) | ARGEGYDIPFDP |  |
| IGHV4-59 (Human) | IGHJ2 (Human) | ARQGWLRGNFDL |  |
| IGHV3-66 (Human) | IGHJ2 (Human) | ARALPFFGDLGYFDF |  |
| IGHV4-4 (Human) | IGHJ2 (Human) | ARDREDGMGLWYFDL |  |
| IGHV3-66 (Human) | IGHJ5 (Human) | ARFGSYGRRLYDS |  |
| IGHV3-33 (Human) | IGHJ2 (Human) | AREGDCSGGNCWKGIWYFDL |  |
| IGHV3-66 (Human) | IGHJ6 (Human) | ARESYGMDV |  |
| IGHV1-69 (Human) | IGHJ1 (Human) | ARGSFRDPAMWGPMPEYFQH |  |
| IGHV3-30 (Human) | IGHJ6 (Human) | VKDPVGGYCRGGNCYDFYAMDV |  |
| IGHV1-69 (Human) | IGHJ4 (Human) | ARASYYYDSSGYHFDY |  |
| IGHV3-30 (Human) | IGHJ6 (Human) | AKDGGPYYYDRSGYARDDYYGMDV |  |
| IGHV1-69 (Human) | IGHJ3 (Human) | ARVLFYYDTSGPRSDDAFDI |  |
| IGHV3-23 (Human) | IGHJ6 (Human) | AGRYCSSNTYSGCYYYGMDV |  |
| IGHV1-69 (Human) | IGHJ4 (Human) | ARETWYYDRSGYPRSDEYFQY |  |
| IGHV3-53 (Human) | IGHJ3 (Human) | ARDRGNDAFDI |  |
| IGHV3-66 (Human) | IGHJ4 (Human) | ARELRGYFDY |  |
| IGHV3-66 (Human) | IGHJ6 (Human) | ARESYGMDV |  |
| IGHV1-69 (Human) | IGHJ1 (Human) | ARGSFRDPAMWGPMPEKFQH |  |
| IGHV1-46 (Human) | IGHJ4 (Human) | GRFYDSTGVFDY |  |
| IGHV3-53 (Human) | IGHJ5 (Human) | ARDLEMAGGFDS |  |
| IGHV3-66 (Human) | IGHJ4 (Human) | ARDLQQAGGFDY |  |
| IGHV3-66 (Human) | IGHJ6 (Human) | ARGAGYYYGMDV |  |
| IGHV3-48 (Human) | IGHJ4 (Human) | AREYEGSYGAFDY |  |
| IGHV4-4 (Human) | IGHJ4 (Human) | ARSSGLLDY |  |
| IGHV4-39 (Human) | IGHJ4 (Human) | ARLLGAVFG |  |
| IGHV3-53 (Human) | IGHJ6 (Human) | ARDLQVYGMDV |  |
| IGHV4-59 (Human) | IGHJ5 (Human) | ARGPPGFDS |  |
| IGHV3-49 (Human) | IGHJ3 (Human) | TRVVDILTGYYYPDAFDI |  |
| IGHV1-69 (Human) | IGHJ3 (Human) | AREDIEEVPAAFAYAFDI |  |
| IGHV1-69 (Human) | IGHJ3 (Human) | ATHIVLVEFGGGDDAFDI |  |
| IGHV3-30 (Human) | IGHJ3 (Human) | ARQLYYYDRSGFLHGDAFDI |  |
| IGHV4-4 (Human) | IGHJ4 (Human) | ARSSGLLDY |  |
| IGHV4-39 (Human) | IGHJ4 (Human) | ARLPLGYCSGGTCFPKSL |  |
| IGHV4-39 (Human) | IGHJ4 (Human) | ARGLGAFDY |  |
| IGHV1-69D (Human) | IGHJ6 (Human) | ARDLGEGILHDYGGPYYYYAMDV |  |
| IGHV4-4 (Human) | IGHJ3 (Human) | ARERSAVTTWGSAAFHI |  |
| IGHV3-66 (Human) | IGHJ3 (Human) | AREGSLEVAGPKLDAFDI |  |
| IGHV3-9 (Human) | IGHJ4 (Human) | AKDKIHSSWYGEEAGDFDY |  |
| IGHV3-66 (Human) | IGHJ4 (Human) | ARDRGGFIDY |  |
| IGHV3-53 (Human) | IGHJ3 (Human) | ARDLAAAGAFDI |  |
| IGHV3-66 (Human) | IGHJ6 (Human) | ARDLSVRGGMDV |  |
| IGHV3-53 (Human) | IGHJ4 (Human) | ARDVPRNSGGY |  |
| IGHV3-66 (Human) | IGHJ4 (Human) | ARDYGDFYFDY |  |
| IGHV3-53 (Human) | IGHJ3 (Human) | ARDLSTMGAFDI |  |
| IGHV1-69 (Human) | IGHJ6 (Human) | ARDPRPLVPAAINYGMDV |  |
| IGHV3-30 (Human) | IGHJ4 (Human) | AKAPRGYYDRSGYYRIQDNFDY |  |
| IGHV3-9 (Human) | IGHJ6 (Human) | VKDYHSVRYCSSTSCYRALDDYYGMDV |  |
| IGHV5-51 (Human) | IGHJ3 (Human) | ARRDRWELEPFDI |  |
| IGHV3-53 (Human) | IGHJ4 (Human) | ARDVPRNSGGY |  |
| IGHV3-30 (Human) | IGHJ4 (Human) | AKGHDFGAPDY |  |
| IGHV4-39 (Human) | IGHJ4 (Human) | ARQNGLCDY |  |
| IGHV3-9 (Human) | IGHJ4 (Human) | AKDTVRGIIITKENYYFDY |  |
| IGHV3-66 (Human) | IGHJ4 (Human) | ARDLHIYGLDC |  |
| IGHV4-39 (Human) | IGHJ5 (Human) | ASQFVGYSGWYLGNNWFDS |  |
| IGHV3-66 (Human) | IGHJ3 (Human) | ARDVYEGAFDI |  |
| IGHV1-69D (Human) | IGHJ4 (Human) | ARGLIPAAWYFDR |  |
| IGHV3-9 (Human) | IGHJ4 (Human) | AKNVYDSSGFYLDY |  |
| IGHV3-53 (Human) | IGHJ4 (Human) | ARDYMLRGGED |  |
| IGHV3-9 (Human) | IGHJ5 (Human) | AKSIRQWQVRGAEYYFDS |  |
| IGHV3-66 (Human) | IGHJ4 (Human) | ARDLHTYGLDY |  |
| IGHV3-53 (Human) | IGHJ6 (Human) | ARGGGHYYGMDV |  |
| IGHV3-66 (Human) | IGHJ4 (Human) | ARDLGYFDF |  |
| IGHV3-9 (Human) | IGHJ4 (Human) | AKDLGSGDGYNFSLDY |  |
| IGHV4-4 (Human) | IGHJ3 (Human) | ARVALWGINLPSYFSRGFDI |  |
| IGHV4-59 (Human) | IGHJ5 (Human) | ARHYDSSGYTYNWFDP |  |
| IGHV1-69 (Human) | IGHJ5 (Human) | ARTSYASFAGLGWFDP |  |
| IGHV5-51 (Human) | IGHJ4 (Human) | ARSDGYSYGYY |  |
| IGHV1-69 (Human) | IGHJ3 (Human) | ARQGKRTYYDILTGSPAGAFDI |  |
| IGHV3-23 (Human) | IGHJ2 (Human) | AKPPRGYYDSSGYYYEGWYFDL |  |
| IGHV3-24 (Human) | IGHJ3 (Human) | AKPPRGYYDSSGYYYEGWYFDL |  |
| IGHV3-25 (Human) | IGHJ4 (Human) | AKSPRGYYDSSGYYYEGWYFDL |  |
| IGHV3-26 (Human) | IGHJ5 (Human) | AKPPRGYYDSSGYYYEGWYFDL |  |
| IGHV1-2 (Human) | IGHJ3 (Human) | ARHYGDYGEDAFDI |  |
| IGHV1-3 (Human) | IGHJ4 (Human) | ARHYGDYGEDAFDI |  |
| IGHV1-4 (Human) | IGHJ5 (Human) | ARHYGDYGEDAFDI |  |
| IGHV1-5 (Human) | IGHJ6 (Human) | ARHYGDYGEDAFDI |  |
| IGHV3-21 (Human) | IGHJ4 (Human) | ARDFSGHTAWAGTGFEY |  |
| IGHV1-18 (Human) | IGHJ4 (Human) | ARDYTRGAWFGESLIGGFDN |  |
| IGHV3-66 (Human) | IGHJ4 (Human) | AAPKVGLGPRTALGHLAFMTLPALNY |  |
| IGHV7-4-1 (Human) | IGHJ6 (Human) | SSEITTLGGMDV |  |
| IGHV3-23 (Human) | IGHJ4 (Human) | ARDKILWFGETTYYFDY |  |
| IGHV5-51 (Human) | IGHJ4 (Human) | ARGDYGDYGEFDY |  |
| IGHV3-21 (Human) | IGHJ4 (Human) | ARPSGADYYDSSGDYDLRY |  |
| IGHV3-23 (Human) | IGHJ6 (Human) | AKSQGDDSSGYYPLYYYYYGMDV |  |
| IGHV5-51 (Human) | IGHJ4 (Human) | ARVGSYQPSFDY |  |
| IGHV1-69 (Human) | IGHJ6 (Human) | ARDIDSSGYYYGTYYYYGMDV |  |
| IGHV3-30 (Human) | IGHJ1 (Human) | AGEPYLRGYCRGGSCYGPSAGYFQH |  |
| IGHV3-23 (Human) | IGHJ4 (Human) | ARALYGPPGHAIWFGELLEPGFDY |  |
| IGHV3-23 (Human) | IGHJ3 (Human) | AAVGAAAGTGAFDI |  |
| IGHV4-39 (Human) | IGHJ3 (Human) | VKSQWLRGAFDI |  |
| IGHV1-69 (Human) | IGHJ4 (Human) | ALPLGQPYYFDY |  |
| IGHV3-30 (Human) | IGHJ6 (Human) | AKDKLTGYLYYYYGMDV |  |
| IGHV1-69 (Human) | IGHJ6 (Human) | AREAGYMGYYYYYMDV |  |
| IGHV1-69 (Human) | IGHJ3 (Human) | ARDGTKGGPAFNAFDI |  |
| IGHV1-69 (Human) | IGHJ5 (Human) | ARDPTRDCSGGSCYSGWFDP |  |
| IGHV1-69 (Human) | IGHJ5 (Human) | AREVDTAMVIAGDDAFDI |  |
| IGHV1-2 (Human) | IGHJ3 (Human) | ARVTHSSSWADAFDI |  |
| IGHV4-4 (Human) | IGHJ4 (Human) | ARLTSNDY |  |
| IGHV1-69 (Human) | IGHJ4 (Human) | ARDPAYCGGDCYSVPWVK |  |
| IGHV5-51 (Human) | IGHJ3 (Human) | ARPYLRYFDYDAFDI |  |
| IGHV3-30 (Human) | IGHJ6 (Human) | ALRGYSYGSIKYGMDV |  |
| IGHV3-30 (Human) | IGHJ6 (Human) | ARARGYDSSGYYFFYYYYGMDV |  |
| IGHV3-9 (Human) | IGHJ3 (Human) | AKGQLWGGYDAFDI |  |
| IGHV3-9 (Human) | IGHJ6 (Human) | AKPIFVRGYSGYDSLEYGMDV |  |
| IGHV1-69 (Human) | IGHJ3 (Human) | ARGWVYSSGWYDAFDI |  |
| IGHV1-46 (Human) | IGHJ4 (Human) | ARETSLWGLGY |  |
| IGHV3-23 (Human) | IGHJ5 (Human) | AKSRQLAFDP |  |
| IGHV5-51 (Human) | IGHJ3 (Human) | ARGGYSSGFDI |  |
| IGHV1-69 (Human) | IGHJ4 (Human) | AWGILDY |  |
| IGHV1-69 (Human) | IGHJ6 (Human) | ARDRYYYDSSGYYYDYYYGMDV |  |
| IGHV3-9 (Human) | IGHJ4 (Human) | AKDIYSTYGAHFDY |  |
| IGHV3-30 (Human) | IGHJ6 (Human) | ARALTGGYYYGMDV |  |
| IGHV1-69 (Human) | IGHJ4 (Human) | AREELGCSSTSCALIDY |  |
| IGHV3-21 (Human) | IGHJ4 (Human) | ARPSGADYYDSSGDYDLRY |  |
| IGHV3-20 (Human) | IGHJ4 (Human) | ARGSGGLDY |  |
| IGHV1-69 (Human) | IGHJ6 (Human) | ARDDIGYCSSTSCSYYYYGMDV |  |
| IGHV3-30 (Human) | IGHJ4 (Human) | AKDRGNGWSSFDY |  |
| IGHV3-21 (Human) | IGHJ4 (Human) | ARAGHRFLEWLPSYIDY |  |
| IGHV1-46 (Human) | IGHJ6 (Human) | ARDLTEDYGDYARIYYYYGMDV |  |
| IGHV3-30 (Human) | IGHJ4 (Human) | ARPRGGGYNFQWDY |  |
| IGHV1-18 (Human) | IGHJ4 (Human) | ARDRGLNWWLQFDGKDY |  |
| IGHV3-48 (Human) | IGHJ3 (Human) | ARGGNEYCSGSRCYQDAFDT |  |
| IGHV3-30 (Human) | IGHJ6 (Human) | ARALYGTYYYGMDV |  |
| IGHV3-66 (Human) | IGHJ4 (Human) | ATAAYYYDSSYYQY |  |
| IGHV3-30 (Human) | IGHJ4 (Human) | ARDSWGDY |  |
| IGHV3-30 (Human) | IGHJ4 (Human) | ARAKGGYYYFDY |  |
| IGHV3-21 (Human) | IGHJ4 (Human) | ARAWPRFLEWLAPLFDY |  |
| IGHV3-23 (Human) | IGHJ4 (Human) | AKDQGYDYVWGSYRPTPCFDY |  |
| IGHV3-30 (Human) | IGHJ4 (Human) | ARGGGGSYTYFDY |  |
| IGHV5-51 (Human) | IGHJ4 (Human) | ARYGYSYGSDY |  |
| IGHV1-69D (Human) | IGHJ3 (Human) | AREGEIKYYDSSGYWSDAFDI |  |
| IGHV3-21 (Human) | IGHJ3 (Human) | TRGDAFDI |  |
| IGHV1-2 (Human) | IGHJ4 (Human) | ARGKIDY |  |
| IGHV3-9 (Human) | IGHJ4 (Human) | AKDFDTIWFGEFQPPDY |  |
| IGHV3-30 (Human) | IGHJ6 (Human) | AREYDFWSGYSPESEAYYYYMDV |  |
| IGHV3-53 (Human) | IGHJ6 (Human) | ARDLDYYGMDV |  |
| IGHV3-30 (Human) | IGHJ4 (Human) | ARDSEDCSSLSCYLDF |  |
| IGHV1-69D (Human) | IGHJ6 (Human) | ARVGAPIERTSSSWHYYYYGMDV |  |
| IGHV3-53 (Human) | IGHJ4 (Human) | ARDYGDFYFDY |  |
| IGHV3-53 (Human) | IGHJ6 (Human) | ARDLVARGMDV |  |
| IGHV1-58 (Human) | IGHJ3 (Human) | AAPYCSGGSCHDGFDI |  |
| IGHV1-69D (Human) | IGHJ4 (Human) | ASFGGDDGLDK |  |
| IGHV1-24 (Human) | IGHJ6 (Human) | ATLAYCTNGVCYILGPPPLRKHEELYYFYMDV |  |
| IGHV1-69D (Human) | IGHJ4 (Human) | ARLGRGDYDSSGYYKVYFDY |  |
| IGHV3-53 (Human) | IGHJ3 (Human) | ARDQGEDVFDI |  |
| IGHV4-34 (Human) | IGHJ5 (Human) | SRGVVLNNVVWFDP |  |
| IGHV3-66 (Human) | IGHJ3 (Human) | ARNIYDAFDI |  |
| IGHV3-66 (Human) | IGHJ4 (Human) | ASPGIVPAAMGVFDY |  |
| IGHV3-53 (Human) | IGHJ4 (Human) | ARLPYGDPA |  |
| IGHV1-24 (Human) | IGHJ6 (Human) | AASFAVSGTPGPPSHYNYYYGMDV |  |
| IGHV1-24 (Human) | IGHJ6 (Human) | ATGWSITGTPSNYKYYYGMDV |  |
| IGHV1-69D (Human) | IGHJ4 (Human) | ARLGRGDYDSSGYYKVYFDY |  |
| IGHV3-21 (Human) | IGHJ6 (Human) | ASILEWFAGYMDV |  |
| IGHV1-58 (Human) | IGHJ3 (Human) | AAPYCYSTSCADGFDI |  |
| IGHV1-58 (Human) | IGHJ3 (Human) | AAPYCNKTRCSDGFDI |  |
| IGHV3-30 (Human) | IGHJ4 (Human) | AREELRALADY |  |
| IGHV1-24 (Human) | IGHJ5 (Human) | TTQGSGPFGEPRGWFDP |  |
| IGHV1-58 (Human) | IGHJ3 (Human) | AANNCGRTTCSDAFDI |  |
| IGHV1-58 (Human) | IGHJ3 (Human) | AAPYCGGDCNDGFDV |  |
| IGHV5-10-1 (Human) | IGHJ5 (Human) | AKHKFFGELPIRGFDP |  |
| IGHV1-69D (Human) | IGHJ6 (Human) | ARTYSTYPKNRLDWDYYYYYGMDV |  |
| IGHV3-15 (Human) | IGHJ4 (Human) | TTDPGWWRIAVAGTNY |  |
| IGHV3-30 (Human) | IGHJ4 (Human) | ARDSEDCSSLSCYLDY |  |
| IGHV1-58 (Human) | IGHJ3 (Human) | AAPYCNTTSCDDGFDI |  |
| IGHV3-7 (Human) | IGHJ5 (Human) | AADLGILWFGDLRKSEP |  |
| IGHV1-58 (Human) | IGHJ3 (Human) | AAPNCNRTICADGFDI |  |
| IGHV1-58 (Human) | IGHJ3 (Human) | AAPGCNTTICPDGFDI |  |
| IGHV5-10-1 (Human) | IGHJ6 (Human) | ARENFWSVYYTGIDYYMDV |  |
| IGHV1-58 (Human) | IGHJ3 (Human) | AAPYCSGGTCHDGFDI |  |
| IGHV3-66 (Human) | IGHJ6 (Human) | ARDLAVYGMDV |  |
| IGHV3-23 (Human) | IGHJ4 (Human) | AKDFVVVVAARSHDDYYFDY |  |
| IGHV1-58 (Human) | IGHJ3 (Human) | AAPHCGGGSCYDGFDI |  |
| IGHV4-39 (Human) | IGHJ1 (Human) | ARHTGYYDSSGYYRLEYFQH |  |
| IGHV1-58 (Human) | IGHJ3 (Human) | AAPNCSGGSCYDAFDI |  |
| IGHV1-58 (Human) | IGHJ3 (Human) | AAPACSSTRCYDGFDI |  |
| IGHV3-53 (Human) | IGHJ6 (Human) | ARDLITYGMDV |  |
| IGHV3-7 (Human) | IGHJ3 (Human) | TRAGWVRGAFDI |  |
| IGHV3-66 (Human) | IGHJ4 (Human) | ARDRGDYLFDY |  |
| IGHV3-66 (Human) | IGHJ6 (Human) | ARDAVYYGMDV |  |
| IGHV3-15 (Human) | IGHJ3 (Human) | TTDLCRSTSCEHDAFDI |  |
| IGHV1-46 (Human) | IGHJ6 (Human) | AKDRVTIFWGNGMDV |  |
| IGHV4-34 (Human) | IGHJ5 (Human) | VGGVVLDNVVWFDP |  |
| IGHV3-11 (Human) | IGHJ6 (Human) | ARSPPTGDSSDWYDSPAYNNYYMDV |  |
| IGHV1-58 (Human) | IGHJ3 (Human) | AAPNCSRTLCYDGFNM |  |
| IGHV3-53 (Human) | IGHJ6 (Human) | ARVGGYCSSANCVSDV |  |
| IGHV1-58 (Human) | IGHJ3 (Human) | AAPNCSGGSCYDGFDL |  |
| IGHV3-23 (Human) | IGHJ4 (Human) | ARDLWGSGFFAFDV |  |
| IGHV3-23 (Human) | IGHJ4 (Human) | ARGRDLAAFTKTAFDV |  |
| IGHV3-23 (Human) | IGHJ4 (Human) | ARDRWASGWLAFDV |  |
| IGHV3-23 (Human) | IGHJ4 (Human) | ARDRDHAYDWGFDV |  |
| IGHV3-15 (Human) | IGHJ4 (Human) | TTAGSYYYDTVGPGLPEGKFDY |  |
| IGHV1-58 (Human) | IGHJ3 (Human) | AAPYCSSISCNDGFDI |  |
| IGHV3-33 (Human) | IGHJ6 (Human) | ARETVSYGMDV |  |
| IGHV4-59 (Human) | IGHJ3 (Human) | ARDRGYSSGWTDGFDI |  |
| IGHV1-2 (Human) | IGHJ6 (Human) | ARGLGVGCSGGNCYLDYYYMDV |  |
| IGHV1-24 (Human) | IGHJ6 (Human) | ATAPAVAGPFYYYYYGMDV |  |
| IGHV1-24 (Human) | IGHJ6 (Human) | ATAPAVAGPFYYYYYGMDV |  |
| IGHV1-24 (Human) | IGHJ6 (Human) | ATAPAVAGPFYYYYYGMDV |  |
| IGHV3-33 (Human) | IGHJ6 (Human) | VRETVDGMDV |  |
| IGHV1-24 (Human) | IGHJ6 (Human) | ATAPAVAGPLYYYYYGMDV |  |
| IGHV1-24 (Human) | IGHJ6 (Human) | ATAPAVAGPFYYYYYGMDV |  |
| IGHV3-33 (Human) | IGHJ6 (Human) | VRETVDGMDV |  |
| IGHV3-33 (Human) | IGHJ6 (Human) | VRETVDGMDV |  |
| IGHV1-24 (Human) | IGHJ6 (Human) | ATAPAVAGPFYNFYYGIDV |  |
| IGHV1-2 (Human) | IGHJ3 (Human) | ARGGSRCSGGNCYGWAYDAFDI |  |
| IGHV1-2 (Human) | IGHJ4 (Human) | ARAAPFYDFWSGYSYFDY |  |
| IGHV3-21 (Human) | IGHJ4 (Human) | ARDGNAYKWLLAENVRFDY |  |
| IGHV3-33 (Human) | IGHJ4 (Human) | ARAVAGEWYFDY |  |
| IGHV3-33 (Human) | IGHJ4 (Human) | ARAFPDSSSWSGFTIDY |  |
| IGHV1-2 (Human) | IGHJ2 (Human) | ARVPYCSSTSCHRDWYFDL |  |
| IGHV1-69 (Human) | IGHJ4 (Human) | ARADYYYDSSGYFFDY |  |
| IGHV5-51 (Human) | IGHJ4 (Human) | ARIRGVYSSGWIGGDY |  |
| IGHV1-46 (Human) | IGHJ4 (Human) | ARGSPKGAFDY |  |
| IGHV1-46 (Human) | IGHJ6 (Human) | ARDREPHSDSSGYWDSLKYYYYYALDV |  |
| IGHV1-69 (Human) | IGHJ4 (Human) | ARGFNGNYYGWGDDDAFDI |  |
| IGHV3-30 (Human) | IGHJ4 (Human) | ASGSDYGDYLLVY |  |
| IGHV3-53 (Human) | IGHJ6 (Human) | ARDLSEGGMDV |  |
| IGHV1-69 (Human) | IGHJ6 (Human) | ATRKETTVTTSLVYGMDV |  |
| IGHV1-2 (Human) | IGHJ6 (Human) | ARGPFYYDNSGTLGGLDV |  |
| IGHV3-11 (Human) | IGHJ4 (Human) | ARDGVIPPRFDY |  |
| IGHV1-2 (Human) | IGHJ3 (Human) | ARGPYYYDSSGSLGAFDI |  |
| IGHV1-18 (Human) | IGHJ6 (Human) | AGSDNYGFPYNGMDV |  |
| IGHV1-2 (Human) | IGHJ4 (Human) | ARDSRFSYVNGEFDY |  |
| IGHV1-2 (Human) | IGHJ6 (Human) | ARVGWYDFGTPGDYYYYYGMDV |  |
| IGHV1-2 (Human) | IGHJ6 (Human) | VRGPFYYDSSGPLGGMDV |  |
| IGHV3-9 (Human) | IGHJ6 (Human) | AKDFLWDLHPPRYYGMDV |  |
| IGHV3-53 (Human) | IGHJ4 (Human) | ARGGRLADAAGDY |  |
| IGHV3-53 (Human) | IGHJ4 (Human) | ARGHYDLFDY |  |
| IGHV3-53 (Human) | IGHJ6 (Human) | ARGDGWDNYYYGMDV |  |
| IGHV3-66 (Human) | IGHJ4 (Human) | ARGFGDYYFDY |  |
| IGHV1-2 (Human) | IGHJ4 (Human) | ARGPRYSGTHFDY |  |
| IGHV1-2 (Human) | IGHJ4 (Human) | ARGPRYSGTYFDY |  |
| IGHV1-2 (Human) | IGHJ4 (Human) | ARGPRYSGTYFDY |  |
| IGHV3-53 (Human) | IGHJ4 (Human) | ARDPYGYSSIWDGQGGH |  |
| IGHV3-21 (Human) | IGHJ6 (Human) | ARGGYCSDGSCYVQDRLIYYYSGLDV |  |
| IGHV3-48 (Human) | IGHJ4 (Human) | ARDRRRRYCTNGVCYRPEEIDY |  |
| IGHV3-53 (Human) | IGHJ4 (Human) | ARDYGDLYFDY |  |
| IGHV3-23 (Human) | IGHJ4 (Human) | AKANKYSSSEFDF |  |
| IGHV1-2 (Human) | IGHJ3 (Human) | ATESWVYGSGSYSSGAFDI |  |
| IGHV1-2 (Human) | IGHJ4 (Human) | ARGPRYSGTYFDY |  |
| IGHV1-2 (Human) | IGHJ4 (Human) | ARGPRYSGTYFDY |  |
| IGHV1-2 (Human) | IGHJ4 (Human) | ARGPRYSGTYFDY |  |
| IGHV1-2 (Human) | IGHJ4 (Human) | ARGPRYSGTYFDY |  |
| IGHV7-4-1 (Human) | IGHJ5 (Human) | AVYYYDSGSPGWFDP |  |
| IGHV1-69 (Human) | IGHJ5 (Human) | ARDFRYCSSTRCYFWFDP |  |
| IGHV1-46 (Human) | IGHJ4 (Human) | ARWYDSTGSIDY |  |
| IGHV3-9 (Human) | IGHJ4 (Human) | AKDQGYSYGNYFDY |  |
| IGHV1-69 (Human) | IGHJ4 (Human) | ALRNQWDLLVY |  |
| IGHV1-58 (Human) | IGHJ3 (Human) | AAPYCSGGTCLDGFDI |  |
| IGHV3-9 (Human) | IGHJ4 (Human) | AKAGVRGIAAAGPDLNFDY |  |
| IGHV4-61 (Human) | IGHJ3 (Human) | ARETYYYDSSGYYISDAFDI |  |
| IGHV4-61 (Human) | IGHJ3 (Human) | ARESFYYDRSGYYGSDAFDI |  |
| IGHV3-53 (Human) | IGHJ4 (Human) | AREGDVEGLHDFWSGYSRDRYYFDY |  |
| IGHV1-2 (Human) | IGHJ3 (Human) | ARGPLFHKLVYDSWSGYHDGFDI |  |
| IGHV1-2 (Human) | IGHJ3 (Human) | ARGPLFHRLVYDFWSGYHDGFDM |  |
| IGHV1-2 (Human) | IGHJ3 (Human) | ARGPLFHKLVYDSWTGYHDGFDI |  |
| IGHV3-21 (Human) | IGHJ4 (Human) | ARERGYYGGKTPPFL |  |
| IGHV3-53 (Human) | IGHJ6 (Human) | ARDVGDYYGMDV |  |
| IGHV3-30 (Human) | IGHJ3 (Human) | ARDMEVDYYDRSGHYHVFHAFDI |  |
| IGHV3-33 (Human) | IGHJ6 (Human) | ARDHSSSSFVYYYYMDV |  |
| IGHV3-15 (Human) | IGHJ4 (Human) | TTTNDYGDYSPAY |  |
| IGHV3-21 (Human) | IGHJ4 (Human) | ARERYGDN |  |
| IGHV4-59 (Human) | IGHJ5 (Human) | ARLKQQLVGFGWFDP |  |
| IGHV4-59 (Human) | IGHJ5 (Human) | ARLKQQLVGFGWFDP |  |
| IGHV1-46 (Human) | IGHJ5 (Human) | ARASTSTTSWSDALSLGS |  |
| IGHV3-33 (Human) | IGHJ4 (Human) | AREQEANYYDISGYYHWGESLGY |  |
| IGHV1-69 (Human) | IGHJ6 (Human) | ARDSGYSGYGSTYYMDV |  |
| IGHV1-69 (Human) | IGHJ6 (Human) | ARDSGYSGYGSTYYMDV |  |
| IGHV4-59 (Human) | IGHJ5 (Human) | ARHYDILTALSWFDP |  |
| IGHV4-59 (Human) | IGHJ5 (Human) | ARHYDILTSLSWFDP |  |
| IGHV3-30 (Human) | IGHJ4 (Human) | VKEGRPSDTVVVVAFDY |  |
| IGHV3-15 (Human) | IGHJ4 (Human) | TTGPQYDDFGHSYIVDS |  |
| IGHV4-31 (Human) | IGHJ4 (Human) | ARVWQYYDSTGSFDY |  |
| IGHV1-69 (Human) | IGHJ3 (Human) | ARGVVGATPGSFDL |  |
| IGHV4-4 (Human) | IGHJ5 (Human) | ARGDVLDWFDP |  |
| IGHV3-23 (Human) | IGHJ6 (Human) | AKNIAEMSTFDDYFYYYGMDV |  |
| IGHV3-30 (Human) | IGHJ4 (Human) | AKAALGYCTNGVCYCDN |  |
| IGHV4-4 (Human) | IGHJ5 (Human) | AGSYSNYIGGVWFDP |  |
| IGHV3-66 (Human) | IGHJ4 (Human) | ARDLVVYGLDC |  |
| IGHV3-23 (Human) | IGHJ6 (Human) | ANHPLASGDEYYYYYMDV |  |
| IGHV3-66 (Human) | IGHJ4 (Human) | ARDLAGRLDY |  |
| IGHV3-66 (Human) | IGHJ4 (Human) | ARDLVVYGADY |  |
| IGHV1-69 (Human) | IGHJ3 (Human) | ARGVVAATPGNFDI |  |
| IGHV3-66 (Human) | IGHJ4 (Human) | ARDIAGRLDY |  |
| IGHV4-4 (Human) | IGHJ4 (Human) | GVCAGDCYAASVFDY |  |
| IGHV3-66 (Human) | IGHJ6 (Human) | ANHGYYYYMDV |  |
| IGHV3-53 (Human) | IGHJ4 (Human) | ARDRGGGILDY |  |
| IGHV3-53 (Human) | IGHJ4 (Human) | AKTPRGDYDSSGTSAY |  |
| IGHV3-9 (Human) | IGHJ4 (Human) | AKGLDSSSSASPDY |  |
| IGHV1-46 (Human) | IGHJ4 (Human) | ARGGYCGSTSCSPDDYFDY |  |
| IGHV3-23 (Human) | IGHJ3 (Human) | AKVLSPTYYDSWSGPDAFDF |  |
| IGHV3-9 (Human) | IGHJ4 (Human) | VKGYRYYYDILTGYYNDAGAFDY |  |
| IGHV5-51 (Human) | IGHJ5 (Human) | ARLSERWYSPFDS |  |
| IGHV3-15 (Human) | IGHJ3 (Human) | TTDCFWRLGGTTCYEHDAFDV |  |
| IGHV3-9 (Human) | IGHJ4 (Human) | ARGLDGSSSASPDS |  |
| IGHV3-49 (Human) | IGHJ3 (Human) | SRGGYYDGSPYYWNRPDAFDI |  |
| IGHV3-9 (Human) | IGHJ4 (Human) | AKGVEYSSSSNCDY |  |
| IGHV3-66 (Human) | IGHJ4 (Human) | ARETLGRGGDC |  |
| IGHV3-30 (Human) | IGHJ4 (Human) | TTDDPGSYYYGMDV |  |
| IGHV3-15 (Human) | IGHJ6 (Human) | ARPLLPGETGSLNRLDY |  |
| IGHV3-9 (Human) | IGHJ3 (Human) | AKIADIVRAYDFWSGQHFDAFDI |  |
| IGHV3-9 (Human) | IGHJ3 (Human) | AKIADLVGAYDFRSGQHFAAFDV |  |
| IGHV3-15 (Human) | IGHJ3 (Human) | STVGSYYYDSRGPTSDAFDI |  |
| IGHV3-53 (Human) | IGHJ3 (Human) | ARDLSVVGAFDI |  |
| IGHV4-31 (Human) | IGHJ5 (Human) | ARAIVVVTLNWFDL |  |
| IGHV4-59 (Human) | IGHJ6 (Human) | ATYYFDNSGYSYGLDV |  |
| IGHV3-53 (Human) | IGHJ4 (Human) | ARGDGELIFDQ |  |
| IGHV3-49 (Human) | IGHJ4 (Human) | TRWDGWSQHDY |  |
| IGHV4-4 (Human) | IGHJ3 (Human) | ARDGGRPGDAFDL |  |
| IGHV1-69 (Human) | IGHJ4 (Human) | ASFHVAYGDYIPFDY |  |
| IGHV1-69 (Human) | IGHJ4 (Human) | ATFHVAYGDYIPFDS |  |
| IGHV3-30 (Human) | IGHJ6 (Human) | AKDPLPFRDFFYYYMDV |  |
| IGHV3-30 (Human) | IGHJ6 (Human) | AKDPLPFRDYYYYYMDV |  |
| IGHV5-51 (Human) | IGHJ4 (Human) | ARGGPPGGVKLELTDF |  |
| IGHV5-51 (Human) | IGHJ4 (Human) | ARGGPPGGVKLELTDY |  |
| IGHV3-30 (Human) | IGHJ4 (Human) | AKPVDAAMFDF |  |
| IGHV3-53 (Human) | IGHJ6 (Human) | ARPVVGGRAGMDV |  |
| IGHV4-59 (Human) | IGHJ4 (Human) | ARCAWLRGSFDY |  |
| IGHV3-53 (Human) | IGHJ6 (Human) | ARHPYGTDV |  |
| IGHV1-69 (Human) | IGHJ4 (Human) | ASRWEQLNGGSWHYFDY |  |
| IGHV3-73 (Human) | IGHJ5 (Human) | TKDIAAGIPALNWFDS |  |
| IGHV4-30 (Human) | IGHJ1 (Human) | ARDAIGSASYGVEYFQH |  |
| IGHV1-24 (Human) | IGHJ4 (Human) | ATGGLFMIRGLEI |  |
| IGHV1-2 (Human) | IGHJ6 (Human) | ARYKGTTVNTNYYYGMDV |  |
| IGHV1-2 (Human) | IGHJ6 (Human) | ARYKGTTVNTNYYYGMDV |  |
| IGHV1-8 (Human) | IGHJ6 (Human) | ASRRWDPLTFYYYMVV |  |
| IGHV3-30 (Human) | IGHJ5 (Human) | AKSWWLSENWFDP |  |
| IGHV3-30 (Human) | IGHJ5 (Human) | AKSWWLSENWFDP |  |
| IGHV1-58 (Human) | IGHJ3 (Human) | AAVDCNSTSCYDAFDI |  |
| IGHV1-58 (Human) | IGHJ3 (Human) | AAPYCNVTTCFDGFNI |  |
| IGHV3-66 (Human) | IGHJ6 (Human) | ARDLMEVGGMDV |  |
| IGHV5-51 (Human) | IGHJ3 (Human) | ARSFRDDPRIAVAGPADAFDI |  |
| IGHV3-53 (Human) | IGHJ4 (Human) | ARGDVSGYRYGLDY |  |
| IGHV3-53 (Human) | IGHJ4 (Human) | ARGDVSGYRYGLDY |  |
| IGHV3-53 (Human) | IGHJ5 (Human) | ARIYGDYA |  |
| IGHV3-53 (Human) | IGHJ4 (Human) | ARDLGTGLFDY |  |
| IGHV3-53 (Human) | IGHJ4 (Human) | ARDYGDYYFDY |  |
| IGHV3-30 (Human) | IGHJ4 (Human) | AKEGRPSDIVVVVAFDY |  |
| IGHV4-59 (Human) | IGHJ6 (Human) | ARAAGVRSVLAAASSYYYYYGLDV |  |
| IGHV3-49 (Human) | IGHJ4 (Human) | ARGLRPTRKADY |  |
| IGHV1-69 (Human) | IGHJ5 (Human) | ARGGEEGIAVAAKGWTEQNYENWFDP |  |
| IGHV1-46 (Human) | IGHJ3 (Human) | ASGYCSSGSCHAGDAFDI |  |
| IGHV1-69 (Human) | IGHJ1 (Human) | AREGSAHYDSSGLNAEYFQH |  |
| IGHV3-53 (Human) | IGHJ6 (Human) | ARDLDYYGMDV |  |
| IGHV3-53 (Human) | IGHJ4 (Human) | ARDWGEYYFDY |  |
| IGHV3-53 (Human) | IGHJ6 (Human) | ARDLGVSGMDV |  |
| IGHV3-48 (Human) | IGHJ4 (Human) | ARVGARITGTSTYYFDY |  |
| IGHV1-69 (Human) | IGHJ5 (Human) | AREFAVVPVASTWWFDP |  |
| IGHV3-11 (Human) | IGHJ3 (Human) | ARVFLRSAYPSSRVVWAFDI |  |
| IGHV3-11 (Human) | IGHJ5 (Human) | ARGGYCTSTSCYKGFPNWFDP |  |
| IGHV3-33 (Human) | IGHJ3 (Human) | ARSQYYYDRSGYANHDAFDI |  |
| IGHV3-53 (Human) | IGHJ4 (Human) | ARVVPGNED |  |
| IGHV1-69 (Human) | IGHJ6 (Human) | ARDDYGTLWAHYYYGMVV |  |
| IGHV3-23 (Human) | IGHJ4 (Human) | AKVTFRTELTQVVPNYFDF |  |
| IGHV3-49 (Human) | IGHJ4 (Human) | TRRIMYYSDNSGSRTFDY |  |
| IGHV3-33 (Human) | IGHJ3 (Human) | ARELYYYDRSGYYGPDDYAFDI |  |
| IGHV3-53 (Human) | IGHJ4 (Human) | ARPRYGSRSYYAGDY |  |
| IGHV3-53 (Human) | IGHJ4 (Human) | ARDLGAAGALDF |  |
| IGHV3-53 (Human) | IGHJ4 (Human) | ARSYGDYYLDF |  |
| IGHV3-53 (Human) | IGHJ4 (Human) | ARDWGDNYFDY |  |
| IGHV3-53 (Human) | IGHJ6 (Human) | AREVYGMDV |  |
| IGHV3-53 (Human) | IGHJ6 (Human) | ARSLEAYGMDV |  |
| IGHV3-53 (Human) | IGHJ3 (Human) | ARFIPPDSRGYSSGAFDI |  |
| IGHV1-69 (Human) | IGHJ4 (Human) | ARVRGTAAAGPNEHIAAAGTFDY |  |
| IGHV1-69 (Human) | IGHJ5 (Human) | ARGGGSSGYNWFDP |  |
| IGHV1-8 (Human) | IGHJ5 (Human) | ARGGRYCSGDSCYSNIWFDP |  |
| IGHV3-53 (Human) | IGHJ4 (Human) | ARGPYPAADNY |  |
| IGHV3-53 (Human) | IGHJ4 (Human) | ARDYGDYYFDY |  |
| IGHV3-53 (Human) | IGHJ5 (Human) | ARWARGFDP |  |
| IGHV3-9 (Human) | IGHJ6 (Human) | AKDMSGPDYGGIEEYGMDV |  |
| IGHV4-39 (Human) | IGHJ4 (Human) | ARLLGLIDY |  |
| IGHV4-31 (Human) | IGHJ4 (Human) | ARALGLIDY |  |
| IGHV4-38-2 (Human) | IGHJ3 (Human) | VRALGAFDI |  |
| IGHV4-59 (Human) | IGHJ3 (Human) | ARDRGYDSSGPDAFDI |  |
| IGHV1-69 (Human) | IGHJ5 (Human) | ARDRVESSGWGYWFDP |  |
| IGHV3-53 (Human) | IGHJ4 (Human) | ARVAYHMGVTHFDS |  |
| IGHV3-53 (Human) | IGHJ3 (Human) | ASLYTSGWNFRGAPQV |  |
| IGHV1-69 (Human) | IGHJ4 (Human) | ARGRGYSGYGASYYFDY |  |
| IGHV4-4 (Human) | IGHJ4 (Human) | ARAFQDGTSGPYYFDS |  |
| IGHV3-53 (Human) | IGHJ6 (Human) | ARDLDYYGMDV |  |
| IGHV1-18 (Human) | IGHJ6 (Human) | ARVKSNSGPQLRFLEWLLFDYYMDV |  |
| IGHV1-46 (Human) | IGHJ6 (Human) | ARVGSVTYYDILTGHPPYYYYGMDV |  |
| IGHV1-58 (Human) | IGHJ3 (Human) | AAPHCNRTSCFDGFDI |  |
| IGHV3-7 (Human) | IGHJ6 (Human) | ARDLGLLWFGEDPRARDV |  |
| IGHV1-69 (Human) | IGHJ4 (Human) | ARDRWLADVVVPAAIYPLGY |  |
| IGHV1-18 (Human) | IGHJ4 (Human) | ARSTGILDC |  |
| IGHV3-53 (Human) | IGHJ6 (Human) | ARDLYYYGMDV |  |
| IGHV4-28 (Human) | IGHJ5 (Human) | ARGEVLTSGGYNARFDP |  |
| IGHV3-53 (Human) | IGHJ6 (Human) | ARDLHIYGMDV |  |
| IGHV1-69 (Human) | IGHJ6 (Human) | ARGNDDSSGYYSHYYYGMDV |  |
| IGHV1-2 (Human) | IGHJ6 (Human) | ARARGSSGWYRIGTRWGNWFDP |  |
| IGHV1-58 (Human) | IGHJ3 (Human) | AAPYCSSTNCYDAFDI |  |
| IGHV3-30 (Human) | IGHJ6 (Human) | ARGWAYWELLPDYYYGMDV |  |
| IGHV3-30 (Human) | IGHJ6 (Human) | ARDLAIAVAGTWHYYNGMDV |  |
| IGHV1-58 (Human) | IGHJ3 (Human) | AAPYCSGGSCFDGFDI |  |
| IGHV5-51 (Human) | IGHJ6 (Human) | AGGSGISTPMDV |  |
| IGHV1-69 (Human) | IGHJ6 (Human) | ARDTATGGMDV |  |
| IGHV3-23 (Human) | IGHJ4 (Human) | ARSYYYGGFGMDY |  |
| IGHV3-23 (Human) | IGHJ4 (Human) | ARSYYYGGFGMDY |  |
| IGHV3-23 (Human) | IGHJ4 (Human) | AKDADSFDY |  |
| IGHV3-23 (Human) | IGHJ3 (Human) | AKDDYGDYVLGAFDI |  |
| IGHV3-23 (Human) | IGHJ4 (Human) | VKDFVGADGPFVFDY |  |
| IGHV4-59 (Human) | IGHJ6 (Human) | ARSAKHWLAPPGDYYYYMDV |  |
| IGHV1-46 (Human) | IGHJ4 (Human) | ARPGGGSYQEFDY |  |
| IGHV1-24 (Human) | IGHJ6 (Human) | ATGWAVAGSSDVWYYYYGMDV |  |
| IGHV1-69 (Human) | IGHJ5 (Human) | ARGVGYRGVIPLNWFDP |  |
| IGHV1-24 (Human) | IGHJ4 (Human) | ATGWAYKSTWYFGY |  |
| IGHV1-69 (Human) | IGHJ6 (Human) | ASLQTVDTAIEKYYGMDV |  |
| IGHV2-70 (Human) | IGHJ4 (Human) | ARMIVTTSTYFDY |  |
| IGHV2-70 (Human) | IGHJ4 (Human) | ARMIVTTSTYFDY |  |
| IGHV2-5 (Human) | IGHJ4 (Human) | ARLTAADTIFDC |  |
| IGHV1-24 (Human) | IGHJ5 (Human) | ATTTVHCSGGSCSSYWFDP |  |
| IGHV1-69 (Human) | IGHJ4 (Human) | ARDAPDYDFWSGRPRYFDS |  |
| IGHV1-8 (Human) | IGHJ5 (Human) | ARGGRYCSGGSCFSGIWFDP |  |
| IGHV4-59 (Human) | IGHJ4 (Human) | ASDYSDSTGYYYGFDH |  |
| IGHV3-13 (Human) | IGHJ6 (Human) | ARVKFRFLEWFLDV |  |
| IGHV4-34 (Human) | IGHJ2 (Human) | ARGVGWFYYWYFDL |  |
| IGHV2-70 (Human) | IGHJ4 (Human) | ARMRVGGYDSYFDY |  |
| IGHV5-51 (Human) | IGHJ6 (Human) | TRHQYGYNYGYFYYYIDV |  |
| IGHV2-5 (Human) | IGHJ4 (Human) | AHYSSSSLYFAY |  |
| IGHV3-66 (Human) | IGHJ4 (Human) | ARGEGWDLPFDF |  |
| IGHV3-43 (Human) | IGHJ4 (Human) | GRDLYRRISGYFGTTFDY |  |
| IGHV3-33 (Human) | IGHJ3 (Human) | ARGRIGNSMIVDVTGEDAFDI |  |
| IGHV3-21 (Human) | IGHJ3 (Human) | VRARMNYYDSKGYYPDAFDI |  |
| IGHV1-24 (Human) | IGHJ5 (Human) | ATSSPFTSYNWFDP |  |
| IGHV2-5 (Human) | IGHJ4 (Human) | AHRCVDTAVLAFDY |  |
| IGHV4-39 (Human) | IGHJ5 (Human) | ARPIAVYQVLKQHNNWFDP |  |
| IGHV1-69 (Human) | IGHJ5 (Human) | ARERVYSGSGAAYWFDP |  |
| IGHV1-18 (Human) | IGHJ4 (Human) | ARDLELGGAFDY |  |
| IGHV1-46 (Human) | IGHJ5 (Human) | ARSRVAPSEDWFDP |  |
| IGHV3-20 (Human) | IGHJ6 (Human) | ARVGSTIFGVVRRNYYYYMDV |  |
| IGHV3-53 (Human) | IGHJ4 (Human) | AREVAGTYDY |  |
| IGHV3-53 (Human) | IGHJ5 (Human) | ARSPYGGNS |  |
| IGHV1-24 (Human) | IGHJ5 (Human) | ATGSPFGVVTDWFDP |  |
| IGHV1-46 (Human) | IGHJ4 (Human) | ARDVRVDDSWSGYDLLSGGTYFDY |  |
| IGHV1-2 (Human) | IGHJ6 (Human) | ARAPLFPTGVLAGDYYYYGMDV |  |
| IGHV3-30 (Human) | IGHJ4 (Human) | ARHATLMNNKDI |  |
| IGHV1-69 (Human) | IGHJ6 (Human) | ARGYYEARHYYYYYAMDV |  |
| IGHV5-51 (Human) | IGHJ4 (Human) | ARQESGWSFDY |  |
| IGHV3-23 (Human) | IGHJ4 (Human) | AKGQRGSPDFFDY |  |
| IGHV1-3 (Human) | IGHJ4 (Human) | ARAGWELNY |  |
| IGHV3-15 (Human) | IGHJ5 (Human) | STTNDYGDYSANY |  |
| IGHV1-8 (Human) | IGHJ5 (Human) | ARGLWFGDLTRTKYNWFDP |  |
| IGHV2-70 (Human) | IGHJ4 (Human) | ARITPHLVYDY |  |
| IGHV3-53 (Human) | IGHJ6 (Human) | ARDLENGGLDV |  |
| IGHV1-58 (Human) | IGHJ3 (Human) | AAPNCNSTTCHDGFDI |  |
| IGHV3-30 (Human) | IGHJ5 (Human) | AKDPTSLYCSGGSCYNNWFDP |  |
| IGHV3-30 (Human) | IGHJ3 (Human) | AGGGVLVTSDPDAFDI |  |
| IGHV3-66 (Human) | IGHJ4 (Human) | ARDYGDYYFDY |  |
| IGHV3-66 (Human) | IGHJ6 (Human) | ARDPMRPGMDV |  |
| IGHV1-69 (Human) | IGHJ4 (Human) | ATGRYTYGYGYYFDY |  |
| IGHV2-5 (Human) | IGHJ3 (Human) | AHRLAPDYDFLTGYYNGDDAFDV |  |
| IGHV1-18 (Human) | IGHJ2 (Human) | ARARQLVLNWYFDL |  |
| IGHV3-7 (Human) | IGHJ4 (Human) | ARLMYYYGNFDY |  |
| IGHV3-13 (Human) | IGHJ2 (Human) | ARVGYYGSGSYPLYWYFDL |  |
| IGHV1-69 (Human) | IGHJ6 (Human) | ATDGGGGSYYYAHYYYGMDV |  |
| IGHV3-33 (Human) | IGHJ4 (Human) | ARDGVDFGMVTLFDY |  |
| IGHV3-30 (Human) | IGHJ4 (Human) | AKQASPYCSGGSCYSGNFDY |  |
| IGHV4-31 (Human) | IGHJ4 (Human) | ARDYGGNSNYFHY |  |
| IGHV3-66 (Human) | IGHJ6 (Human) | AETGWDGMDV |  |
| IGHV2-70 (Human) | IGHJ4 (Human) | ARVQVAAAGSPYDY |  |
| IGHV3-9 (Human) | IGHJ4 (Human) | AKDRGYEILTPASFDY |  |
| IGHV4-59 (Human) | IGHJ5 (Human) | ASTYWDSSGYYYGVDY |  |
| IGHV1-69 (Human) | IGHJ6 (Human) | ARYMVTRDQYYYDMDV |  |
| IGHV4-39 (Human) | IGHJ4 (Human) | ASQQWLRGNFDY |  |
| IGHV3-23 (Human) | IGHJ6 (Human) | AKDLFYDFWSGFDFGPGESFGMDV |  |
| IGHV3-48 (Human) | IGHJ4 (Human) | ARMAIVGAYANWGFDY |  |
| IGHV1-24 (Human) | IGHJ5 (Human) | ATTTPFGVVNAIWFDP |  |
| IGHV1-24 (Human) | IGHJ5 (Human) | ATGSPFGVVTSWFDP |  |
| IGHV3-33 (Human) | IGHJ4 (Human) | ARDRVDSSSWGFYFDY |  |
| IGHV3-33 (Human) | IGHJ5 (Human) | ARDVAHCTTVGCHGNWFDT |  |
| IGHV3-30 (Human) | IGHJ4 (Human) | AKKGSPYCGVDCYKGYFDY |  |
| IGHV3-30 (Human) | IGHJ4 (Human) | AKAPYSYAYSVYFFDY |  |
| IGHV1-46 (Human) | IGHJ6 (Human) | ARDYFLIPAANTMEV |  |
| IGHV3-48 (Human) | IGHJ4 (Human) | ACMVGPTAPFDF |  |
| IGHV3-9 (Human) | IGHJ6 (Human) | ARGGFKSGFYIHGMDV |  |
| IGHV3-23 (Human) | IGHJ4 (Human) | AKLAVAGGRGANY |  |
| IGHV3-64D (Human) | IGHJ3 (Human) | VKDKVTTIFDVFDL |  |
| IGHV3-53 (Human) | IGHJ4 (Human) | ARDYGDYYFDY |  |
| IGHV3-53 (Human) | IGHJ4 (Human) | ARDLQQLGIDY |  |
| IGHV3-23 (Human) | IGHJ4 (Human) | ARHNELDY |  |
| IGHV3-23 (Human) | IGHJ4 (Human) | ARHNELDY |  |
| IGHV3-23 (Human) | IGHJ4 (Human) | ARHNELDH |  |
| IGHV4-61 (Human) | IGHJ4 (Human) | ARDVDIVGVFDY |  |
| IGHV4-4 (Human) | IGHJ2 (Human) | ARGFTNWYFDL |  |
| IGHV1-46 (Human) | IGHJ5 (Human) | ARERVGVGIYSRSSESDFPTFDP |  |
| IGHV3-9 (Human) | IGHJ6 (Human) | AKDILSRVHYYDSSGSYGMDV |  |
| IGHV1-2 (Human) | IGHJ6 (Human) | ARDLEDFWSGYPPLGYALDV |  |
| IGHV3-53 (Human) | IGHJ3 (Human) | ARDLGSRGAFDI |  |
| IGHV4-4 (Human) | IGHJ4 (Human) | AMVQGVMVY |  |
| IGHV3-13 (Human) | IGHJ6 (Human) | ARSRYYDFWSGYYSVDDYGMDV |  |
| IGHV4-61 (Human) | IGHJ4 (Human) | ARGLRFCSDTRCYPYFDY |  |
| IGHV1-18 (Human) | IGHJ6 (Human) | ARWIHRDFDWLPYGMFMDV |  |
| IGHV1-18 (Human) | IGHJ6 (Human) | ARWIHRDFDWLPYGMFMDV |  |
| IGHV1-18 (Human) | IGHJ6 (Human) | ARWIHRDFDWLPYGMFMDV |  |
| IGHV1-69 (Human) | IGHJ6 (Human) | SGFGGDDGMDV |  |
| IGHV4-31 (Human) | IGHJ4 (Human) | ARADIVVVPAAAFDY |  |
| IGHV1-2 (Human) | IGHJ6 (Human) | ARETSFAIFGGGGMDV |  |
| IGHV3-30-3 (Human) | IGHJ4 (Human) | AREDYYDSSGSFDH |  |
| IGHV3-30-3 (Human) | IGHJ4 (Human) | AREDYYDSSGSLDY |  |
| IGHV3-30-3 (Human) | IGHJ4 (Human) | AREDYYDSSGSLDY |  |
| IGHV1-69 (Human) | IGHJ4 (Human) | ATKPYYYDSSGYYGGFDY |  |
| IGHV1-69 (Human) | IGHJ4 (Human) | ARANYYYESSGYYFDY |  |
| IGHV5-10-1 (Human) | IGHJ4 (Human) | ARLVPGQWHFDY |  |
| IGHV3-30 (Human) | IGHJ6 (Human) | AKVLTWSIHPEDYYYGMDV |  |
| IGHV7-4-1 (Human) | IGHJ4 (Human) | AGGYDFWSGYPNPTDY |  |
| IGHV3-66 (Human) | IGHJ6 (Human) | ARDPSSRGYYGMDV |  |
| IGHV3-53 (Human) | IGHJ6 (Human) | ARGEYNWNDEDYYYGMDV |  |
| IGHV1-69-2 (Human) | IGHJ4 (Human) | GSGSEAHYGIDY |  |
| IGHV1-69 (Human) | IGHJ4 (Human) | AENLQDDSGTLYSGG |  |
| IGHV1-69 (Human) | IGHJ4 (Human) | AENLQDDSGTLYSGG |  |
| IGHV3-66 (Human) | IGHJ4 (Human) | ARGEGGSIVGVTSDY |  |
| IGHV3-53 (Human) | IGHJ6 (Human) | ARDLNEHGLDV |  |
| IGHV3-9 (Human) | IGHJ6 (Human) | AKAHSTGHQYYYGMDV |  |
| IGHV1-2 (Human) | IGHJ4 (Human) | ARVVVLGYGRPNNYYDGRNVWDY |  |
| IGHV1-8 (Human) | IGHJ4 (Human) | ARMRTGWPTHGRPDDF |  |
| IGHV3-53 (Human) | IGHJ3 (Human) | ARSYDILTGYRDAFDI |  |
| IGHV1-58 (Human) | IGHJ3 (Human) | AAPHCNRTSCYDAFDL |  |
| IGHV1-8 (Human) | IGHJ4 (Human) | ARMRSGWPTHGRPDDF |  |
| IGHV3-9 (Human) | IGHJ6 (Human) | AKDIIRQGEDGMDV |  |
| IGHV3-23 (Human) | IGHJ3 (Human) | VKGLFDWFPL |  |
| IGHV3-7 (Human) | IGHJ4 (Human) | ARLGFYYGGADY |  |
| IGHV3-53 (Human) | IGHJ5 (Human) | ARDFLRWHDL |  |
| IGHV3-53 (Human) | IGHJ5 (Human) | ARDFLRWHDL |  |
| IGHV3-66 (Human) | IGHJ6 (Human) | ARDLVTYGLDV |  |
| IGHV3-20 (Human) | IGHJ3 (Human) | ARARGPSEQYYDLLTGYYDAFDI |  |
| IGHV2-5 (Human) | IGHJ3 (Human) | ARHQIVVLFDM |  |
| IGHV3-23 (Human) | IGHJ3 (Human) | VKGLFDWFPL |  |
| IGHV5-51 (Human) | IGHJ5 (Human) | ARLGVSKYCSGGRCLSGGSNWFDP |  |
| IGHV3-53 (Human) | IGHJ3 (Human) | ASSSWLRGAFDI |  |
| IGHV1-58 (Human) | IGHJ3 (Human) | AAPHCNRTSCYDAFDL |  |
| IGHV1-58 (Human) | IGHJ3 (Human) | AAPYCSRTSCHDAFDI |  |
| IGHV3-9 (Human) | IGHJ6 (Human) | AKDIIRQGEDGMDV |  |
| IGHV1-8 (Human) | IGHJ4 (Human) | ARMRSGWPTHGRPDDF |  |
| IGHV1-69 (Human) | IGHJ5 (Human) | ARGVVAATPGWFDP |  |
| IGHV4-39 (Human) | IGHJ3 (Human) | ARHTVDCGGDCFPNDAFDI |  |
| IGHV1-2 (Human) | IGHJ4 (Human) | ARVVVLGYGRPNNYYDGRNVWDY |  |
| IGHV4-59 (Human) | IGHJ3 (Human) | ARATWLRDAFGI |  |
| IGHV1-8 (Human) | IGHJ4 (Human) | ARMRTGWPTHGRPDDF |  |
| IGHV1-8 (Human) | IGHJ4 (Human) | ARMRTGWPTHGRPDDF |  |
| IGHV4-39 (Human) | IGHJ4 (Human) | ARLLWLRGHFDY |  |
| IGHV3-20 (Human) | IGHJ3 (Human) | AVIMSPIPRYSGYDWAGGAFDI |  |
| IGHV1-69 (Human) | IGHJ6 (Human) | ARLSGSGWLGYAMDV |  |
| IGHV3-53 (Human) | IGHJ3 (Human) | ARGPEPDAFDI |  |
| IGHV3-53 (Human) | IGHJ5 (Human) | ARDFLRWHDL |  |
| IGHV3-53 (Human) | IGHJ3 (Human) | ARSYDILTGYRDAFDI |  |
| IGHV2-70 (Human) | IGHJ4 (Human) | ARETPVTAIDY |  |
| IGHV3-53 (Human) | IGHJ6 (Human) | ARDALYYNGPGRDGMDV |  |
| IGHV3-53 (Human) | IGHJ6 (Human) | ARDALYYNGPGRDGMDV |  |
| IGHV4-31 (Human) | IGHJ5 (Human) | ARVVPTRGPVAWFDP |  |
| IGHV3-21 (Human) | IGHJ4 (Human) | ARGGSILWWLIDY |  |
| IGHV3-66 (Human) | IGHJ4 (Human) | ARGDGGYYSPFDY |  |
| IGHV3-66 (Human) | IGHJ4 (Human) | AREVVGYFDC |  |
| IGHV4-59 (Human) | IGHJ4 (Human) | ARLRWLRGGIDF |  |
| IGHV2-70 (Human) | IGHJ3 (Human) | ARTMATINAFDI |  |
| IGHV3-66 (Human) | IGHJ6 (Human) | ARDLVTYGLDV |  |
| IGHV3-30 (Human) | IGHJ6 (Human) | AKGGPNKEVLYFGELLDYGMDV |  |
| IGHV1-58 (Human) | IGHJ3 (Human) | AAPYCSSISCNDGFDI |  |
| IGHV1-58 (Human) | IGHJ3 (Human) | AAPYCSSISCNDGFDI |  |
| IGHV3-53 (Human) | IGHJ4 (Human) | AREGEVEGYNDFWSGYSRDRYYFDY |  |
| IGHV3-30 (Human) | IGHJ4 (Human) | ASSSGYLFHSDY |  |
| IGHV1-18 (Human) | IGHJ4 (Human) | ARDYTRGAWFGESLIGGFDN |  |
| IGHV2-70 (Human) | IGHJ6 (Human) | ARIPGFLRYRNRYYYYGMDV |  |
| IGHV1-58 (Human) | IGHJ6 (Human) | AAPNCSNVVCYDGFDI |  |
| IGHV1-18 (Human) | IGHJ6 (Human) | ARPDYQVLGYDFWIGYYGMDV |  |
| IGHV5-51 (Human) | IGHJ4 (Human) | ARQWSHYTYDYYY |  |
| IGHV1-58 (Human) | IGHJ3 (Human) | ASPYCSGGSCSDGFDI |  |
| IGHV3-53 (Human) | IGHJ6 (Human) | ARDRDYYGMDV |  |
| IGHV3-53 (Human) | IGHJ4 (Human) | ARALQVGATSDYFDY |  |
| IGHV3-53 (Human) | IGHJ6 (Human) | ARDLQEHGMDV |  |
| IGHV1-69 (Human) | IGHJ5 (Human) | ARDSRYCSGGSCYSVWFDP |  |
| IGHV1-2 (Human) | IGHJ6 (Human) | ARDRSWAVVYYYMDV |  |
| IGHV3-30-3 (Human) | IGHJ4 (Human) | AKDGGKLWVYYFDY |  |
| IGHV3-53 (Human) | IGHJ6 (Human) | ARDLERAGGMDV |  |
| IGHV3-53 (Human) | IGHJ3 (Human) | ARAHVDTAMVESGAFDI |  |
| IGHV3-53 (Human) | IGHJ4 (Human) | ARGEGWELPYDY |  |
| IGHV3-53 (Human) | IGHJ6 (Human) | ARDLDVSGGMDV |  |
| IGHV1-24 (Human) | IGHJ6 (Human) | ATSTAVAGTPDLFDYYYGMDV |  |
| IGHV1-18 (Human) | IGHJ5 (Human) | ARDGELLGWFDP |  |
| IGHV3-49 (Human) | IGHJ6 (Human) | TRVRRLWFGSYYYGMDV |  |
| IGHV3-49 (Human) | IGHJ6 (Human) | TRVRRLWFGSYYYGMDV |  |
| IGHV1-2 (Human) | IGHJ4 (Human) | ARASVSTITDFDY |  |
| IGHV1-2 (Human) | IGHJ4 (Human) | ARASVATITDFDY |  |
| IGHV1-8 (Human) | IGHJ4 (Human) | ARATTDCSSTSCWSLDFWSGYYTGGREKIFD |  |
| IGHV7-4-1 (Human) | IGHJ5 (Human) | ARSLRGANLVP |  |
| IGHV7-4-1 (Human) | IGHJ5 (Human) | ARSLRGANLVP |  |
| IGHV3-33 (Human) | IGHJ6 (Human) | ARAARRPVVTDTMAYYMDV |  |
| IGHV1-58 (Human) | IGHJ3 (Human) | AAPHCSSTICYDGFDI |  |
| IGHV3-30 (Human) | IGHJ5 (Human) | AKGGDYEWELLES |  |
| IGHV3-66 (Human) | IGHJ4 (Human) | ARDFGDFFFDY |  |
| IGHV3-66 (Human) | IGHJ4 (Human) | ARDYGDYFFDY |  |
| IGHV1-58 (Human) | IGHJ3 (Human) | AAPYCSSTRCYDAFDI |  |
| IGHV3-9 (Human) | IGHJ4 (Human) | AKDINYDSGGYHKNYFDY |  |
| IGHV3-23 (Human) | IGHJ4 (Human) | ALASGSYFGGANY |  |
| IGHV3-66 (Human) | IGHJ4 (Human) | ATGARFGESPFDY |  |
| IGHV3-66 (Human) | IGHJ4 (Human) | ATGARFGESPFDY |  |
| IGHV1-69 (Human) | IGHJ5 (Human) | AREGGLDYFGSRNSGWTYTWFDP |  |
| IGHV4-39 (Human) | IGHJ3 (Human) | ARGVNYYDRNGYYRNDGFDI |  |
| IGHV3-48 (Human) | IGHJ4 (Human) | ASSKGFCSGGSCSDY |  |
| IGHV3-9 (Human) | IGHJ2 (Human) | AKDLRRQDYYADWYFDL |  |
| IGHV4-39 (Human) | IGHJ3 (Human) | ARGVNYYDRNGYYRNDGFDI |  |
| IGHV3-9 (Human) | IGHJ2 (Human) | AKDLRRQDYYADWYFDL |  |
| IGHV4-39 (Human) | IGHJ3 (Human) | ARGVNYYDRNGYYRNDGFDI |  |
| IGHV7-4-1 (Human) | IGHJ4 (Human) | AKIGSRNSLGV |  |
| IGHV1-58 (Human) | IGHJ3 (Human) | AAPRCSGGSCYDGFDI |  |
| IGHV3-21 (Human) | IGHJ4 (Human) | ASNRSPYDSSNYYFDY |  |
| IGHV3-30 (Human) | IGHJ6 (Human) | AKAFKGNYYYGMDV |  |
| IGHV1-2 (Human) | IGHJ4 (Human) | ARGAASVLRFLEWLLDY |  |
| IGHV3-13 (Human) | IGHJ4 (Human) | ARAQYSSGWYLRFDY |  |
| IGHV3-74 (Human) | IGHJ4 (Human) | ARDLHYDSSGWDY |  |
| IGHV3-66 (Human) | IGHJ3 (Human) | ARDFREGAFDI |  |
| IGHV4-34 (Human) | IGHJ4 (Human) | ARGGTIGTTGIYDILTGYDPFFDY |  |
| IGHV3-9 (Human) | IGHJ4 (Human) | AKDLTGWGLTFGGVITN |  |
| IGHV3-66 (Human) | IGHJ4 (Human) | ARSYGDYYFDY |  |
| IGHV3-30 (Human) | IGHJ4 (Human) | AKMVGQYCSGGNCYLGYFDY |  |
| IGHV3-30 (Human) | IGHJ4 (Human) | AKKGYSYGYFDYYFDY |  |
| IGHV1-69 (Human) | IGHJ5 (Human) | ARDQDSGYIWWFDP |  |
| IGHV3-21 (Human) | IGHJ5 (Human) | ARDPFSRWERPEGWFDP |  |
| IGHV3-66 (Human) | IGHJ6 (Human) | ARESYGMDV |  |
| IGHV3-30 (Human) | IGHJ4 (Human) | AKKGYSYGYFDYYFDY |  |
| IGHV3-53 (Human) | IGHJ4 (Human) | ARGPYPHFDY |  |
| IGHV4-61 (Human) | IGHJ5 (Human) | ARGPDIVVVPAADPRNWFDP |  |
| IGHV4-59 (Human) | IGHJ4 (Human) | TRQLVLVRGYFDY |  |
| IGHV3-9 (Human) | IGHJ6 (Human) | AKDIGPFEAARPGGNYYYYAMDV |  |
| IGHV3-53 (Human) | IGHJ3 (Human) | ARNIYDDAFDV |  |
| IGHV3-30 (Human) | IGHJ6 (Human) | TKADYYDFWSGYQKTYYYYMDV |  |
| IGHV3-66 (Human) | IGHJ4 (Human) | ARELVGYFDY |  |
| IGHV3-66 (Human) | IGHJ4 (Human) | ASTPRGDSYGGGAY |  |
| IGHV3-30 (Human) | IGHJ4 (Human) | AKEGWGYSYGSYYFDY |  |
| IGHV3-13 (Human) | IGHJ3 (Human) | ARAHRGYYDRSGYYHNPDAFDI |  |
| IGHV1-58 (Human) | IGHJ3 (Human) | AAPNCSRTSCQDGFDI |  |
| IGHV3-66 (Human) | IGHJ4 (Human) | ARDRFGRINDY |  |
| IGHV3-30 (Human) | IGHJ4 (Human) | AREGADRSGWWGSFDY |  |
| IGHV1-58 (Human) | IGHJ3 (Human) | AAPYCTTTRCHDGFDI |  |
| IGHV4-31 (Human) | IGHJ4 (Human) | ARDSAAGHFDY |  |
| IGHV3-53 (Human) | IGHJ5 (Human) | ARERGAKAFDP |  |
| IGHV4-38-2 (Human) | IGHJ4 (Human) | ARLPYGYDYVEAFDI |  |
| IGHV4-39 (Human) | IGHJ4 (Human) | ARLGSGSYYTADY |  |
| IGHV3-30 (Human) | IGHJ4 (Human) | AKDGGYYYESSGWFDY |  |
| IGHV3-66 (Human) | IGHJ4 (Human) | ARGFGDRRLDY |  |
| IGHV3-11 (Human) | IGHJ4 (Human) | TRDGVIPPRFDY |  |
| IGHV1-2 (Human) | IGHJ1 (Human) | ARVARHYYDRSGNLHSADYFQH |  |
| IGHV4-34 (Human) | IGHJ4 (Human) | AREMSVAVVDH |  |
| IGHV1-2 (Human) | IGHJ6 (Human) | ARYSNYYYYYGMDV |  |
| IGHV1-24 (Human) | IGHJ6 (Human) | ATGVAVAGTQKNYSYYYGLDV |  |
| IGHV4-30-2 (Human) | IGHJ3 (Human) | ARHSGYDLGGAFDI |  |
| IGHV4-30-4 (Human) | IGHJ4 (Human) | ARQLWLRAPFDY |  |
| IGHV1-69 (Human) | IGHJ6 (Human) | ARESTTIFGVVILTSYGMDV |  |
| IGHV3-13 (Human) | IGHJ4 (Human) | ARASFDSSGYLNYFDY |  |
| IGHV1-18 (Human) | IGHJ4 (Human) | ARDGGILTGYLDYFDH |  |
| IGHV4-39 (Human) | IGHJ4 (Human) | ARCRPEYYFGSGSYLDFDY |  |
| IGHV1-24 (Human) | IGHJ5 (Human) | ATTTPFSSSYWFDP |  |
| IGHV3-53 (Human) | IGHJ5 (Human) | ARGEGSPGNWFDP |  |
| IGHV1-69 (Human) | IGHJ3 (Human) | ARDLLDPQLDDAFDI |  |
| IGHV3-53 (Human) | IGHJ6 (Human) | ARDLMAYGMDV |  |
| IGHV3-66 (Human) | IGHJ4 (Human) | ARDYGDFYFDF |  |
| IGHV1-2 (Human) | IGHJ6 (Human) | ASPASRGYSGYDHGYYYYMDV |  |
| IGHV1-58 (Human) | IGHJ3 (Human) | AAPHCSGGSCLDAFDI |  |
| IGHV1-2 (Human) | IGHJ4 (Human) | ARDSPFSALGASNDY |  |
| IGHV3-9 (Human) | IGHJ4 (Human) | AKAGVRGIAAAGPDLNFDH |  |
| IGHV3-53 (Human) | IGHJ4 (Human) | VRDYGDFYFDY |  |
| IGHV4-34 (Human) | IGHJ3 (Human) | ARKPLLYSDFSPGAFDI |  |
| IGHV4-34 (Human) | IGHJ3 (Human) | ARKPLLYSNLSPGAFDI |  |
| IGHV3-53 (Human) | IGHJ4 (Human) | AREGMGMAAAGT |  |
| IGHV3-53 (Human) | IGHJ4 (Human) | ARESGDTTMAFDY |  |
| IGHV3-53 (Human) | IGHJ3 (Human) | ARDLSAAFDI |  |
| IGHV1-58 (Human) | IGHJ3 (Human) | AAPYCSGGSCSDAFDI |  |
| IGHV1-2 (Human) | IGHJ6 (Human) | ATAHPRRIQGVFFLGPGV |  |
| IGHV3-23 (Human) | IGHJ6 (Human) | ANHPLASGDDYYHYYMDV |  |
| IGHV1-69 (Human) | IGHJ6 (Human) | ARVNQAVTTPFSMDV |  |
| IGHV3-66 (Human) | IGHJ6 (Human) | ARDLYYYGMDV |  |
| IGHV3-53 (Human) | IGHJ4 (Human) | AREGEVEGYYDFWSGYSRDRYYFDY |  |
| IGHV3-53 (Human) | IGHJ4 (Human) | ARDFGEFYFDY |  |
| IGHV1-46 (Human) | IGHJ6 (Human) | ARANHETTMDTYYYYYYMDV |  |
| IGHV7-4-1 (Human) | IGHJ6 (Human) | ARPQGGSSWYRDYYYGMDV |  |
| IGHV1-69 (Human) | IGHJ4 (Human) | ARGPYYYDSGGYYLDY |  |
| IGHV3-53 (Human) | IGHJ6 (Human) | ARDLDVYGLDV |  |
| IGHV3-53 (Human) | IGHJ4 (Human) | ARDFGDFYFDY |  |
| IGHV1-58 (Human) | IGHJ4 (Human) | AADPFADY |  |
| IGHV5-10-1 (Human) | IGHJ4 (Human) | ARVNRVGDGPDF |  |
| IGHV1-69 (Human) | IGHJ5 (Human) | ARGLWFGDSETVWFDP |  |
| IGHV4-34 (Human) | IGHJ3 (Human) | ARKPLLHSSVNPGAFDI |  |
| IGHV1-18 (Human) | IGHJ6 (Human) | ARDPASYYDFWSGYVDYYYYGMDV |  |
| IGHV3-53 (Human) | IGHJ3 (Human) | TRGGWPSGDTFDI |  |
| IGHV3-66 (Human) | IGHJ6 (Human) | ARDRRIIGYYFGMDV |  |
| IGHV3-66 (Human) | IGHJ6 (Human) | VRDRRIVGYYFGLDV |  |
| IGHV3-53 (Human) | IGHJ3 (Human) | ARGGWPSGDTFDI |  |
| IGHV5-51 (Human) | IGHJ6 (Human) | AGGGGISTPMDV |  |
| IGHV5-51 (Human) | IGHJ6 (Human) | AGGGGISTPMDV |  |
| IGHV5-51 (Human) | IGHJ6 (Human) | AGGGGISTPMDV |  |
| IGHV3-23 (Human) | IGHJ4 (Human) | AKTRGRGLYDYVWGSKDY |  |
| IGHV3-53 (Human) | IGHJ6 (Human) | ARDLGPYGMDV |  |
| IGHV3-66 (Human) | IGHJ4 (Human) | ARDRGLVSDY |  |
| IGHV3-53 (Human) | IGHJ6 (Human) | ARDAQNYGMDV |  |
| IGHV3-66 (Human) | IGHJ3 (Human) | ARLINHYYDSSGDGGAFDI |  |
| IGHV1-24 (Human) | IGHJ4 (Human) | ATGPAIAAAETNWFDL |  |
| IGHV3-21 (Human) | IGHJ6 (Human) | ATNGGAHSSTWSFYGMDV |  |
| IGHV3-30 (Human) | IGHJ6 (Human) | AKDGQYYDFWSGYLGARTNPHYYYYMDV |  |
| IGHV1-2 (Human) | IGHJ6 (Human) | ARDLVWATVSGTMDV |  |
| IGHV3-66 (Human) | IGHJ4 (Human) | ARVEWAAAGTFY |  |
| IGHV3-30 (Human) | IGHJ6 (Human) | ARDSEYYDILTGYLAPTHYYYYYMDV |  |
| IGHV3-53 (Human) | IGHJ6 (Human) | AREAYGMDV |  |
| IGHV3-23 (Human) | IGHJ4 (Human) | AKDTGYCGDDCYIKLIRGGPDY |  |
| IGHV1-69 (Human) | IGHJ4 (Human) | ARVYSYDSSGYYLEY |  |
| IGHV3-30 (Human) | IGHJ3 (Human) | ARGVEDPVVPAAIPWCWFDP |  |
| IGHV4-59 (Human) | IGHJ2 (Human) | ARDRIAPVGKFFGWYFDL |  |
| IGHV3-64 (Human) | IGHJ4 (Human) | ARGAEYYDFWSGYYSAYFDY |  |
| IGHV4-38-2 (Human) | IGHJ3 (Human) | ARAVVGIVVVPAAGRRAFDI |  |
| IGHV4-38-2 (Human) | IGHJ2 (Human) | ARTPLSLRLRYNWYFDL |  |
| IGHV1-2 (Human) | IGHJ6 (Human) | AREVMVRGALPPYGMDV |  |
| IGHV4-59 (Human) | IGHJ3 (Human) | ARLERDWPLDAFDI |  |
| IGHV3-53 (Human) | IGHJ3 (Human) | AREGPKSITGTAFDI |  |
| IGHV3-30 (Human) | IGHJ4 (Human) | ARDGQAITMVQGVIGPPFDY |  |
| IGHV3-53 (Human) | IGHJ4 (Human) | ARETLAFDY |  |
| IGHV4-59 (Human) | IGHJ2 (Human) | ASNGQYYDILTGQPPDYWYFDL |  |
| IGHV1-8 (Human) | IGHJ5 (Human) | ARYIVVVPAAKGFDP |  |
| IGHV4-61 (Human) | IGHJ5 (Human) | ARERCYYGSGRAPRCVWFDP |  |
| IGHV3-33 (Human) | IGHJ4 (Human) | ARWFHTGGYFDY |  |
| IGHV3-53 (Human) | IGHJ4 (Human) | ARDYGDFYFDY |  |
| IGHV2-5 (Human) | IGHJ4 (Human) | AHSLFLTVGYSSSWSPFDY |  |
| IGHV3-30 (Human) | IGHJ6 (Human) | AKDMHNDYGDYVSYYFYYGMDV |  |
| IGHV1-18 (Human) | IGHJ6 (Human) | AREGYCSGGSCYSGYYYYYGMDV |  |
| IGHV3-7 (Human) | IGHJ4 (Human) | ARARRADNSGYYGFHFDC |  |
| IGHV3-72 (Human) | IGHJ4 (Human) | ARVHRWAYCINGVCFGAYSDY |  |
| IGHV2-5 (Human) | IGHJ4 (Human) | AHHKIERIFDY |  |
| IGHV3-53 (Human) | IGHJ4 (Human) | ARGDVSGYRYGLDY |  |
| IGHV3-53 (Human) | IGHJ4 (Human) | ARGDVSGYRYGLDY |  |
| IGHV3-30 (Human) | IGHJ5 (Human) | AKDSGYNYGYSWFDP |  |
| IGHV1-24 (Human) | IGHJ6 (Human) | ATGIAVIGPPPSTYYYYGMDV |  |
| IGHV3-33 (Human) | IGHJ6 (Human) | ARDPRDYYDFWSGYDYYYGLDV |  |
| IGHV3-30 (Human) | IGHJ6 (Human) | ARVWLYGSGYMDV |  |
| IGHV3-33 (Human) | IGHJ4 (Human) | ATDPPGLRFRFDY |  |
| IGHV1-46 (Human) | IGHJ2 (Human) | ARADGYEWYFDV |  |
| IGHV1-69 (Human) | IGHJ4 (Human) | AREGRRYGSGWYISTGYFDY |  |
| IGHV2-5 (Human) | IGHJ4 (Human) | AHHSISTIFDH |  |
| IGHV4-59 (Human) | IGHJ4 (Human) | ASSQRPDGNLYYFDY |  |
| IGHV3-23 (Human) | IGHJ6 (Human) | ARDNLGYRPSENLYGMDV |  |
| IGHV3-66 (Human) | IGHJ4 (Human) | ARGYGDYYFDY |  |
| IGHV1-46 (Human) | IGHJ4 (Human) | ADLLLDY |  |
| IGHV3-66 (Human) | IGHJ6 (Human) | VRDLYSYGMDV |  |
| IGHV3-30 (Human) | IGHJ4 (Human) | AREGQWLNWAFDY |  |
| IGHV3-66 (Human) | IGHJ6 (Human) | AAPLLWADSYYMDV |  |
| IGHV1-69 (Human) | IGHJ4 (Human) | ARGIVGATPGYFDY |  |
| IGHV1-69 (Human) | IGHJ2 (Human) | ARETGDQGVTAPFDL |  |
| IGHV1-58 (Human) | IGHJ3 (Human) | AAPYCSSTTCHDGFDI |  |
| IGHV1-69 (Human) | IGHJ4 (Human) | ARRAIDSDTYVEQSHFDY |  |
| IGHV1-69 (Human) | IGHJ6 (Human) | ANFIGDGYNYEEDYMDV |  |
| IGHV3-53 (Human) | IGHJ6 (Human) | AREAYGMDV |  |
| IGHV3-53 (Human) | IGHJ6 (Human) | ARDLVVYGMDV |  |
| IGHV3-66 (Human) | IGHJ6 (Human) | ARDLVVYGMDV |  |
| IGHV3-53 (Human) | IGHJ6 (Human) | ARDAMSYGMDV |  |
| IGHV3-53 (Human) | IGHJ6 (Human) | ARDAAVYGIDV |  |
| IGHV3-66 (Human) | IGHJ6 (Human) | ARDLISRGMDV |  |
| IGHV3-53 (Human) | IGHJ6 (Human) | ARDRVVYGMDV |  |
| IGHV3-53 (Human) | IGHJ6 (Human) | ARDLVSYGMDV |  |
| IGHV3-53 (Human) | IGHJ6 (Human) | ARDLVVYGMDV |  |
| IGHV4-59 (Human) | IGHJ3 (Human) | ARGVLLWFGEPIFEI |  |
| IGHV3-30 (Human) | IGHJ4 (Human) | ARLITMVRGEDY |  |
| IGHV1-24 (Human) | IGHJ6 (Human) | VTAPAITGSPEAYSYYYGMDV |  |
| IGHV1-24 (Human) | IGHJ5 (Human) | ATGPAIAAAATGWFDP |  |
| IGHV1-24 (Human) | IGHJ6 (Human) | VTAPVITGSPEAYSYYYGMDV |  |
| IGHV3-30 (Human) | IGHJ6 (Human) | AKDHDDGYYFYYYMDV |  |
| IGHV1-24 (Human) | IGHJ6 (Human) | AASPAVRGSPSNFYYYHGMDV |  |
| IGHV1-24 (Human) | IGHJ6 (Human) | VAAPVITGSPEAYSYYYGMDV |  |
| IGHV4-30 (Human) | IGHJ3 (Human) | ARMAYQVYYYDSSGYYDAFDI |  |
| IGHV4-61 (Human) | IGHJ3 (Human) | ARMAYQVYYYDSSGYYDAFDI |  |
| IGHV1-24 (Human) | IGHJ5 (Human) | ATSRVAGTPNWFHP |  |
| IGHV3-30 (Human) | IGHJ5 (Human) | ARDLGSGWYP |  |
| IGHV1-24 (Human) | IGHJ5 (Human) | ATSRVAGTPNWFHP |  |
| IGHV1-24 (Human) | IGHJ4 (Human) | ATAAAVRGRGTIDY |  |
| IGHV1-24 (Human) | IGHJ5 (Human) | ATGPAVRRGSWFDP |  |
| IGHV3-11 (Human) | IGHJ5 (Human) | ARVGPAVAGSPFDS |  |
| IGHV1-2 (Human) | IGHJ4 (Human) | ARGHRIPSAISDKYDF |  |
| IGHV1-24 (Human) | IGHJ5 (Human) | ATGPVRGVIGWFDP |  |
| IGHV3-53 (Human) | IGHJ4 (Human) | ARDLQELGSLDY |  |
| IGHV4-34 (Human) | IGHJ4 (Human) | ARGNTMVRGVIIPFEY |  |
| IGHV3-66 (Human) | IGHJ5 (Human) | ASSRPPIGQLVPGLDLDWFDP |  |
| IGHV5-51 (Human) | IGHJ6 (Human) | ARTQWGYNYGSHFFYMDV |  |
| IGHV3-53 (Human) | IGHJ6 (Human) | ARDLEVVGAMDV |  |
| IGHV1-18 (Human) | IGHJ4 (Human) | AIPYSSVTFDC |  |
| IGHV1-8 (Human) | IGHJ6 (Human) | ARGGRYCSSTTCYSGVGMDV |  |
| IGHV3-66 (Human) | IGHJ6 (Human) | AREYYYGMDV |  |
| IGHV4-31 (Human) | IGHJ4 (Human) | ATPGAIMGALHI |  |
| IGHV3-66 (Human) | IGHJ4 (Human) | ARVLPMYGDYLDY |  |
| IGHV3-15 (Human) | IGHJ4 (Human) | TTAGSYYYDTVGPGLPEGKFDY |  |
| IGHV1-58 (Human) | IGHJ3 (Human) | AAPYCSSISCNDGFDI |  |
| IGHV3-73 (Human) | IGHJ4 (Human) | TSVCSGGSCYQ |  |
| IGHV3-74 (Human) | IGHJ4 (Human) | STDSGSIGEF |  |
| IGHV3-49 (Human) | IGHJ6 (Human) | TRISGYYGAGSGGAMDV |  |
| IGHV3-30 (Human) | IGHJ3 (Human) | ARSGWDDAFDI |  |
| IGHV1-8 (Human) | IGHJ6 (Human) | ARGGNGGMDV |  |
| IGHV3-9 (Human) | IGHJ6 (Human) | AKVGEVGSREWSAFDV |  |
| IGHV4-4 (Human) | IGHJ3 (Human) | ARHNAQFGELLVPQDAFDM |  |
| IGHV1-69 (Human) | IGHJ4 (Human) | VRERGYSGYGAAYYFDY |  |
| IGHV3-23 (Human) | IGHJ5 (Human) | VKDFGHLGQMAS |  |
| IGHV3-23 (Human) | IGHJ4 (Human) | VKDLGFADH |  |
| IGHV3-23 (Human) | IGHJ4 (Human) | AREWHSGYDY |  |
| IGHV3-23 (Human) | IGHJ4 (Human) | VKDFVVGETAEFSY |  |
| IGHV3-9 (Human) | IGHJ6 (Human) | ARGGWSSSAGGYYGMDV |  |
| IGHV3-30 (Human) | IGHJ6 (Human) | AKAMFLGDSSGLTGLDMDV |  |
| IGHV3-30 (Human) | IGHJ6 (Human) | ARAYSSSWLLQSFYYYGMDV |  |
| IGHV3-30 (Human) | IGHJ4 (Human) | ARHATLMNNKDI |  |
| IGHV3-53 (Human) | IGHJ6 (Human) | ARDLVVYGMDV |  |
| IGHV3-66 (Human) | IGHJ6 (Human) | ARDLDILGGMDV |  |
| IGHV1-69 (Human) | IGHJ6 (Human) | AREVSDFDWLYRSHYGMDV |  |
| IGHV3-43 (Human) | IGHJ6 (Human) | AKDRSYGPPDVFNYEYGMDV |  |
| IGHV3-11 (Human) | IGHJ4 (Human) | ARDRGTTMVPFDY |  |
| IGHV3-15 (Human) | IGHJ2 (Human) | TTARWDWYFDL |  |
| IGHV3-66 (Human) | IGHJ4 (Human) | ARDHGMAAAGYNY |  |
| IGHV4-4 (Human) | IGHJ4 (Human) | ANMVRGVYEDDY |  |
| IGHV1-69 (Human) | IGHJ4 (Human) | ARTPFYYDSSGYYLDY |  |
| IGHV3-66 (Human) | IGHJ6 (Human) | ARGELGIPYGMDV |  |
| IGHV3-66 (Human) | IGHJ4 (Human) | ARALPYGDLHFDY |  |
| IGHV1-2 (Human) | IGHJ5 (Human) | ARGSRYDWNQNNWFDP |  |
| IGHV3-48 (Human) | IGHJ4 (Human) | ASSSSSGYYFDY |  |
| IGHV1-2 (Human) | IGHJ5 (Human) | AIITIFGVVTWFDP |  |
| IGHV4-59 (Human) | IGHJ4 (Human) | ARDPLLIDY |  |
| IGHV3-9 (Human) | IGHJ6 (Human) | AKDKDWNSRGYYYYGMDV |  |
| IGHV3-66 (Human) | IGHJ4 (Human) | AREGGYSYDYN |  |
| IGHV3-66 (Human) | IGHJ6 (Human) | TRDAQYYGMDV |  |
| IGHV3-9 (Human) | IGHJ6 (Human) | SKDMGRLDYYSGLDV |  |
| IGHV3-66 (Human) | IGHJ6 (Human) | VRDLEVRGGMDV |  |
| IGHV3-66 (Human) | IGHJ5 (Human) | ARYIPRFDP |  |
| IGHV3-66 (Human) | IGHJ6 (Human) | ARDLIKYGMDV |  |
| IGHV3-66 (Human) | IGHJ6 (Human) | ARDLGPYGMDV |  |
| IGHV3-53 (Human) | IGHJ4 (Human) | ARALPGWGGSFEYFDY |  |
| IGHV3-66 (Human) | IGHJ6 (Human) | AREGRIAATGYGMDV |  |
| IGHV3-66 (Human) | IGHJ4 (Human) | ARDQRDFA |  |
| IGHV3-66 (Human) | IGHJ6 (Human) | VRDPVGRYYYGMDV |  |
| IGHV3-53 (Human) | IGHJ6 (Human) | ARDLYYYGMDV |  |
| IGHV3-66 (Human) | IGHJ6 (Human) | ARGEGANYYGMDV |  |
| IGHV3-9 (Human) | IGHJ6 (Human) | AKDGERWDSVVVPSARNGMDV |  |
| IGHV3-48 (Human) | IGHJ6 (Human) | ARRGDGTSSLIHHYYYMDV |  |
| IGHV3-53 (Human) | IGHJ6 (Human) | ARDLFYYGMDV |  |
| IGHV3-30 (Human) | IGHJ6 (Human) | ARSILYGGGMDV |  |
| IGHV4-59 (Human) | IGHJ3 (Human) | AREVYYYDRSGYYASDGFDI |  |
| IGHV3-21 (Human) | IGHJ3 (Human) | TRAGWELRLDAFDI |  |
| IGHV3-53 (Human) | IGHJ6 (Human) | ARDLGEAGGMDV |  |
| IGHV3-53 (Human) | IGHJ4 (Human) | ARDYGDYYFDY |  |
| IGHV3-30 (Human) | IGHJ4 (Human) | AAQDSAYIKSKGSRAYEY |  |
| IGHV3-23 (Human) | IGHJ4 (Human) | AAHHIPTKHPAFPDFRDY |  |
| IGHV3-30 (Human) | IGHJ4 (Human) | AAEAFVQSPYSGSHTTKY |  |
| IGHV3-23 (Human) | IGHJ4 (Human) | AADQYEWWVPGEVGPYLY |  |
| IGHV3-30 (Human) | IGHJ4 (Human) | AAHYEFNDFVWQGYSSDY |  |
| IGHV4-39 (Human) | IGHJ1 (Human) | ARHAAAYYDRSGYYFIEYFQH |  |
| IGHV1-46 (Human) | IGHJ4 (Human) | ASDPRDDIAGGY |  |
| IGHV1-69 (Human) | IGHJ4 (Human) | ARDRGDTIDY |  |
| IGHV3-66 (Human) | IGHJ3 (Human) | ARDVADAFDI |  |
| IGHV1-69 (Human) | IGHJ4 (Human) | ARDGDSGSYYETLGFDY |  |
| IGHV3-66 (Human) | IGHJ6 (Human) | ARDLDYYGMDV |  |
| IGHV3-33 (Human) | IGHJ6 (Human) | ARETVSYGMDV |  |
| IGHV4-59 (Human) | IGHJ3 (Human) | ARDRGYSSGWTDGFDI |  |
| IGHV3-30 (Human) | IGHJ6 (Human) | AKDQDDGYYYYYYMDV |  |
| IGHV1-46 (Human) | IGHJ1 (Human) | ARPPRNYYDRSGYYQRAEYFQH |  |
| IGHV3-30 (Human) | IGHJ4 (Human) | ARGLGLRFLEWPISSY |  |
| IGHV3-66 (Human) | IGHJ6 (Human) | ARGDGSDDYYYGMDV |  |
| IGHV3-66 (Human) | IGHJ6 (Human) | ARGDGSDDYYYGMDV |  |
| IGHV3-11 (Human) | IGHJ3 (Human) | ARGVTSYSDNSGNYRTDDAFDI |  |
| IGHV3-9 (Human) | IGHJ3 (Human) | AKDFSRDGDAFDI |  |
| IGHV3-15 (Human) | IGHJ4 (Human) | TTGALVESRYFDWFTGYYFDY |  |
| IGHV3-21 (Human) | IGHJ3 (Human) | ARDRAYRLGELSSLWGDDAFDI |  |
| IGHV5-51 (Human) | IGHJ4 (Human) | TSGSYYGTLD |  |
| IGHV3-66 (Human) | IGHJ4 (Human) | ARFLPTYDYFDY |  |
| IGHV5-51 (Human) | IGHJ5 (Human) | VYGSGSPSNWFHP |  |
| IGHV1-58 (Human) | IGHJ4 (Human) | AAPHCSGGTCYDGFDI |  |
| IGHV4-34 (Human) | IGHJ6 (Human) | ARGWDLDYYYGMDV |  |
| IGHV3-66 (Human) | IGHJ3 (Human) | ARDLAVAGAFDI |  |
| IGHV1-58 (Human) | IGHJ3 (Human) | AAPYCSSTSCRDGFDI |  |
| IGHV3-66 (Human) | IGHJ4 (Human) | ARDIAGRLDY |  |
| IGHV3-30 (Human) | IGHJ3 (Human) | AKGPRFGWSYRGGSGFDI |  |
| IGHV1-46 (Human) | IGHJ6 (Human) | ARADTPIVVDTTSYFYYMDV |  |
| IGHV1-69 (Human) | IGHJ5 (Human) | ATPLNDYYASGNLGL |  |
| IGHV1-69 (Human) | IGHJ4 (Human) | ATPLNDYYASGNLGLW |  |
| IGHV3-53 (Human) | IGHJ4 (Human) | ARVVGYDFWSGYDGGYFDY |  |
| IGHV3-53 (Human) | IGHJ4 (Human) | ARLVGYDFRSGSDGGYFDY |  |
| IGHV1-69 (Human) | IGHJ5 (Human) | AKVSLTLPIAAAPRFWFDS |  |
| IGHV1-69 (Human) | IGHJ5 (Human) | ARASLTLPIRAAPRFWFDA |  |
| IGHV4-4 (Human) | IGHJ4 (Human) | ARGGDLAMGPEYLDF |  |
| IGHV3-33 (Human) | IGHJ6 (Human) | ARVYGGLPYYYAIDV |  |
| IGHV3-30 (Human) | IGHJ4 (Human) | ARSFSIRIGHKDN |  |
| IGHV3-30 (Human) | IGHJ4 (Human) | ARTFSIRIGHHDY |  |
| IGHV3-9 (Human) | IGHJ4 (Human) | AKGVDYSSSSNFDF |  |
| IGHV3-30 (Human) | IGHJ4 (Human) | ARDALTSISVLFDC |  |
| IGHV3-66 (Human) | IGHJ5 (Human) | ARDTFGRGDDH |  |
| IGHV3-30 (Human) | IGHJ6 (Human) | AKDTPGGDDIMTGWGLYGMDV |  |
| IGHV3-33 (Human) | IGHJ4 (Human) | ARDSNVDTVMVTWFDY |  |
| IGHV3-66 (Human) | IGHJ6 (Human) | ARDLIAFGMDV |  |
| IGHV3-15 (Human) | IGHJ3 (Human) | TTDCFWRLGGTTCYEHDAFDV |  |
| IGHV4-39 (Human) | IGHJ3 (Human) | ARVDSSGWYTGDVFDV |  |
| IGHV3-53 (Human) | IGHJ6 (Human) | ARESYGMDV |  |
| IGHV3-53 (Human) | IGHJ6 (Human) | ARESYGMDV |  |
| IGHV1-46 (Human) | IGHJ4 (Human) | AREGVGGTSYFDY |  |
| IGHV1-46 (Human) | IGHJ4 (Human) | AREGLGATAYFDY |  |
| IGHV3-33 (Human) | IGHJ4 (Human) | ARDPAITEAEIDY |  |
| IGHV3-9 (Human) | IGHJ6 (Human) | AKDLLGNYYYYTLDV |  |
| IGHV4-61 (Human) | IGHJ2 (Human) | ARDIPPTWYFDL |  |
| IGHV5-51 (Human) | IGHJ4 (Human) | AKMVTSGTSYYETRGYASSGPFDN |  |
| IGHV5-51 (Human) | IGHJ4 (Human) | ARRGASWELDY |  |
| IGHV5-51 (Human) | IGHJ4 (Human) | ARRGSSWEVDY |  |
| IGHV1-24 (Human) | IGHJ4 (Human) | ATRGRYCSSGNCYYHH |  |
| IGHV3-30 (Human) | IGHJ3 (Human) | AKALSSTFYFDASGPDAFDI |  |
| IGHV3-66 (Human) | IGHJ3 (Human) | ARDFREGAFDI |  |
| IGHV3-66 (Human) | IGHJ3 (Human) | ARDFREGAIDL |  |
| IGHV3-66 (Human) | IGHJ4 (Human) | ARSYGDYYIDY |  |
| IGHV5-51 (Human) | IGHJ4 (Human) | ARTWSPAAVAFFDS |  |
| IGHV3-53 (Human) | IGHJ4 (Human) | ARGLPTGEGWNYFDY |  |
| IGHV3-9 (Human) | IGHJ4 (Human) | AKGWFGELLGGSDS |  |
| IGHV3-30 (Human) | IGHJ1 (Human) | ARGLLWFGESEYFQH |  |
| IGHV3-11 (Human) | IGHJ6 (Human) | AREGIAAPDSKADAFDI |  |
| IGHV3-9 (Human) | IGHJ4 (Human) | AKAGVRNIAAAGPDLNFDF |  |
| IGHV3-30 (Human) | IGHJ4 (Human) | ARDGTIVTLVRGVMGPPFDY |  |
| IGHV3-15 (Human) | IGHJ3 (Human) | TTGVVVVVSSSPDDAFDV |  |
| IGHV4-31 (Human) | IGHJ4 (Human) | ATGGLSAFGELFPHDK |  |
| IGHV3-30 (Human) | IGHJ4 (Human) | ARDQGTATTYFDH |  |
| IGHV3-30 (Human) | IGHJ4 (Human) | ARDYGDYVTHFDY |  |
| IGHV4-61 (Human) | IGHJ4 (Human) | ARETYYYDRSGYYSSDGFDY |  |
| IGHV4-39 (Human) | IGHJ6 (Human) | ARRGGRTPVRFNYGGDV |  |
| IGHV1-2 (Human) | IGHJ3 (Human) | ARGGQDELTGTFDV |  |
| IGHV4-31 (Human) | IGHJ4 (Human) | ARGSYSDYNGGWDY |  |
| IGHV3-53 (Human) | IGHJ4 (Human) | ARDLSSSGGFDY |  |
| IGHV3-30 (Human) | IGHJ4 (Human) | ARDATMITLVRGIMGPPFDH |  |
| IGHV4-31 (Human) | IGHJ4 (Human) | ARGSYSNYNGGLDY |  |
| IGHV3-23 (Human) | IGHJ6 (Human) | VKESDYYMASVNGMDV |  |
| IGHV1-58 (Human) | IGHJ3 (Human) | AAPQCNRTTCYDAFDM |  |
| IGHV4-31 (Human) | IGHJ3 (Human) | ASTPYTNGGAFHI |  |
| IGHV4-61 (Human) | IGHJ3 (Human) | ARETFFYDRTGHYKSDGFDV |  |
| IGHV3-33 (Human) | IGHJ4 (Human) | AKGQLRLGEFDDY |  |
| IGHV3-53 (Human) | IGHJ4 (Human) | AREGDVEGISDSWSGYSRDRYYFDH |  |
| IGHV3-53 (Human) | IGHJ4 (Human) | AREGDVDGNYGFWSGYSRDRYYFDY |  |
| IGHV1-2 (Human) | IGHJ3 (Human) | ARGPLFHKVVYESSSGFHDGLDF |  |
| IGHV3-21 (Human) | IGHJ4 (Human) | ARGPLFHKVVYESSSGFHDGLDF |  |
| IGHV1-46 (Human) | IGHJ5 (Human) | ARASTSTSSWSEALSLGS |  |
| IGHV3-53 (Human) | IGHJ4 (Human) | VRDFGEFYFDY |  |
| IGHV3-53 (Human) | IGHJ6 (Human) | ARDLIVLGVDV |  |
| IGHV4-31 (Human) | IGHJ4 (Human) | ARVKGWLRGYFDH |  |
| IGHV4-34 (Human) | IGHJ4 (Human) | ARESGSYGTFDY |  |
| IGHV3-53 (Human) | IGHJ4 (Human) | ARLGGVFNGFNGSFDY |  |
| IGHV4-34 (Human) | IGHJ4 (Human) | ARETGTYGTFDH |  |
| IGHV3-48 (Human) | IGHJ4 (Human) | ARGDCTSSSCYSLDY |  |
| IGHV3-48 (Human) | IGHJ4 (Human) | ARGDCLSSSCYSLDY |  |
| IGHV3-66 (Human) | IGHJ4 (Human) | ARDLRDQDGYSYGAFDY |  |
| IGHV3-66 (Human) | IGHJ4 (Human) | ARDLRKDDGYSYGAFDY |  |
| IGHV1-69 (Human) | IGHJ4 (Human) | TINTQWDLVPR |  |
| IGHV3-15 (Human) | IGHJ4 (Human) | TSQLWLRGPGDY |  |
| IGHV5-51 (Human) | IGHJ3 (Human) | ARGGWDPAEYSSSGGGGLDAFDI |  |
| IGHV5-10 (Human) | IGHJ6 (Human) | GRIAPPGRGSYYPTQNYMDV |  |
| IGHV3-15 (Human) | IGHJ4 (Human) | TTDADYSDSSGYYVTYYFEY |  |
| IGHV4-4 (Human) | IGHJ4 (Human) | ARANGILDF |  |
| IGHV3-30 (Human) | IGHJ6 (Human) | ARDFESRTWDPPKYYYALDV |  |
| IGHV5-10 (Human) | IGHJ5 (Human) | ARLSWSPPTRTTDEKNWFDP |  |
| IGHV3-53 (Human) | IGHJ4 (Human) | ARDYGDFYFDY |  |
| IGHV4-34 (Human) | IGHJ3 (Human) | ARKPLLHSNISPGAFDI |  |
| IGHV4-31 (Human) | IGHJ5 (Human) | ARATVVITLHWFDP |  |
| IGHV3-49 (Human) | IGHJ4 (Human) | TRWDGWSQHDY |  |
| IGHV4-59 (Human) | IGHJ6 (Human) | ASYYNDTSGYSYGLDV |  |
| IGHV5-51 (Human) | IGHJ4 (Human) | ARGGPPGGVKLELTDY |  |
| IGHV1-8 (Human) | IGHJ4 (Human) | ARGRANWNSNFLLDS |  |
| IGHV1-8 (Human) | IGHJ4 (Human) | ARGRANYNSKFLLDN |  |
| IGHV3-43 (Human) | IGHJ4 (Human) | AKDSEDCSSTSCYVDH |  |
| IGHV4-30 (Human) | IGHJ4 (Human) | ARAMITFGGVIVVLDY |  |
| IGHV4-30 (Human) | IGHJ4 (Human) | ARAMITFGGVIVLYDY |  |
| IGHV3-11 (Human) | IGHJ3 (Human) | ARVPPPQRLHPFDV |  |
| IGHV3-11 (Human) | IGHJ3 (Human) | ARVPPPQRLHPFDV |  |
| IGHV4-39 (Human) | IGHJ4 (Human) | ARHFADGSGRVVDS |  |
| IGHV4-39 (Human) | IGHJ4 (Human) | ARHFADGSGRVVDY |  |
| IGHV3-11 (Human) | IGHJ4 (Human) | ARDGGGYDRFDH |  |
| IGHV3-11 (Human) | IGHJ4 (Human) | ARDGGAYDRFDY |  |
| IGHV3-30 (Human) | IGHJ5 (Human) | AKSWWLSENWFDP |  |
| IGHV3-30 (Human) | IGHJ5 (Human) | AKSWWLSENWFDP |  |
| IGHV3-30 (Human) | IGHJ4 (Human) | ARDKRGVIRGLLNF |  |
| IGHV3-30 (Human) | IGHJ4 (Human) | AKPVDAAMFDF |  |
| IGHV3-30 (Human) | IGHJ4 (Human) | AKPVDTAMFDS |  |
| IGHV3-53 (Human) | IGHJ6 (Human) | ARDLVVWGMDV |  |
| IGHV3-53 (Human) | IGHJ6 (Human) | ARDLVVWGMDV |  |
| IGHV3-30 (Human) | IGHJ6 (Human) | AKVVVRGVIISLYYGMDV |  |
| IGHV3-30 (Human) | IGHJ6 (Human) | AKVALRGVFISLYYGMDV |  |
| IGHV1-2 (Human) | IGHJ6 (Human) | ARDVIVSMVRGVIFRMDV |  |
| IGHV1-2 (Human) | IGHJ6 (Human) | ARDVIITMGRGVVFRMDV |  |
| IGHV3-33 (Human) | IGHJ3 (Human) | ARDFSNSDMVTLSDAFDI |  |
| IGHV3-53 (Human) | IGHJ4 (Human) | ARDLGTGLFDY |  |
| IGHV3-53 (Human) | IGHJ4 (Human) | ARDLGTGLFDY |  |
| IGHV3-30 (Human) | IGHJ6 (Human) | ASGLLWFETREISGAPDYGMAV |  |
| IGHV3-30 (Human) | IGHJ6 (Human) | ASGLLWFETAGGSGAPDYGMAV |  |
| IGHV4-30 (Human) | IGHJ3 (Human) | ARFCLSGSHYLFAFDI |  |
| IGHV1-69 (Human) | IGHJ4 (Human) | ARNRGYSDYGSVYYFDY |  |
| IGHV3-30 (Human) | IGHJ4 (Human) | AKDIGGGSSPPFFDY |  |
| IGHV4-4 (Human) | IGHJ3 (Human) | ARSSRFLPPLPDAFDL |  |
| IGHV3-11 (Human) | IGHJ6 (Human) | ARRGDGNVPLFHYYYMDV |  |
| IGHV4-4 (Human) | IGHJ3 (Human) | ARDGGRPGDPFDI |  |
| IGHV5-51 (Human) | IGHJ2 (Human) | VRGIAVDWYFDL |  |
| IGHV1-69 (Human) | IGHJ6 (Human) | QSYDTSLSGSRV |  |
| IGHV4-34 (Human) | IGHJ4 (Human) | ARAGFGFVITSRSGTDPLFDY |  |
| IGHV3-49 (Human) | IGHJ4 (Human) | TRAWIPTPHDY |  |
| IGHV3-49 (Human) | IGHJ4 (Human) | SRAWIPTPHDY |  |
| IGHV5-51 (Human) | IGHJ4 (Human) | ARRGSSWEIDH |  |
| IGHV4-59 (Human) | IGHJ5 (Human) | ARHYDILTALSWFDP |  |
| IGHV3-30 (Human) | IGHJ4 (Human) | AKSPINYCANGVCYPDS |  |
| IGHV3-66 (Human) | IGHJ6 (Human) | TRLGGYRYGMDV |  |
| IGHV3-66 (Human) | IGHJ6 (Human) | ARLGGYRYGMDV |  |
| IGHV1-69 (Human) | IGHJ2 (Human) | ARGVGYSGSGSNWYFDL |  |
| IGHV3-49 (Human) | IGHJ4 (Human) | TRLRQVQGVPGYYFDQ |  |
| IGHV3-13 (Human) | IGHJ4 (Human) | ARASGVLTTHFDS |  |
| IGHV3-33 (Human) | IGHJ3 (Human) | AREGVALAGNGVDGFDI |  |
| IGHV3-33 (Human) | IGHJ3 (Human) | AREGVAVGGNGVDGFDM |  |
| IGHV4-59 (Human) | IGHJ5 (Human) | ARLLSTEWLFNWFDP |  |
| IGHV4-59 (Human) | IGHJ5 (Human) | ARLLSTEWSFNWFDP |  |
| IGHV3-15 (Human) | IGHJ3 (Human) | TTVDVQGIWELLENDAFDI |  |
| IGHV3-66 (Human) | IGHJ4 (Human) | ARDLGDSRLDY |  |
| IGHV3-49 (Human) | IGHJ4 (Human) | TRRAHYSGSGLSSYVDY |  |
| IGHV1-69 (Human) | IGHJ4 (Human) | ATGKGYSSSSAAYYFDH |  |
| IGHV1-69 (Human) | IGHJ5 (Human) | ARGGIAVGGWWFDP |  |
| IGHV3-13 (Human) | IGHJ2 (Human) | ARGGSSSWLWYFDL |  |
| IGHV1-2 (Human) | IGHJ6 (Human) | AREPIEGVIGGMIVNYYYMDV |  |
| IGHV1-2 (Human) | IGHJ6 (Human) | AREPIEAVPAGIIVNYYYMDV |  |
| IGHV5-51 (Human) | IGHJ2 (Human) | VRGLPVDWYFDL |  |
| IGHV4-59 (Human) | IGHJ5 (Human) | ARMLSTEWSFNWFDP |  |
| IGHV1-69 (Human) | IGHJ6 (Human) | ARETGYSGFLAVAYMDV |  |
| IGHV3-66 (Human) | IGHJ6 (Human) | ANQGYYYYMDV |  |
| IGHV4-31 (Human) | IGHJ4 (Human) | ARGDTFGRGYYFDY |  |
| IGHV4-31 (Human) | IGHJ4 (Human) | ARGDTFGRGYYFDF |  |
| IGHV3-23 (Human) | IGHJ3 (Human) | AKSDHGDYVIGAFDI |  |
| IGHV4-4 (Human) | IGHJ4 (Human) | ARAQTPEFGELLY |  |
| IGHV3-49 (Human) | IGHJ4 (Human) | ARNDRYIVIVPAEMLY |  |
| IGHV3-13 (Human) | IGHJ3 (Human) | ARGQRGYYDRSGYYWGWRAFDI |  |
| IGHV3-23 (Human) | IGHJ6 (Human) | ANHPLGAAEGYYYYYMDV |  |
| IGHV3-7 (Human) | IGHJ4 (Human) | ALSSGYSGYAGNY |  |
| IGHV3-15 (Human) | IGHJ4 (Human) | TTGSETYYYDSSGPFDY |  |
| IGHV3-23 (Human) | IGHJ4 (Human) | AKVGEYCGGDCYRGLDY |  |
| IGHV3-21 (Human) | IGHJ5 (Human) | ARERGYHGGKTSPFL |  |
| IGHV1-8 (Human) | IGHJ6 (Human) | ARFPKVPAAIFPGDYYYGMDV |  |
| IGHV3-7 (Human) | IGHJ4 (Human) | ARDTIPFWSGYYTSPDYYFDY |  |
| IGHV1-69 (Human) | IGHJ6 (Human) | ARVGAEWPRDHKYYYYGMDV |  |
| IGHV3-9 (Human) | IGHJ3 (Human) | AKIADIVRAYDFWSGQHFDAFDI |  |
| IGHV4-39 (Human) | IGHJ5 (Human) | ATGGRFWGWFDP |  |
| IGHV1-69 (Human) | IGHJ6 (Human) | ARNRAVSEREDYYYGMDV |  |
| IGHV3-66 (Human) | IGHJ4 (Human) | ARDLRDQDGYSYGAFDY |  |
| IGHV1-2 (Human) | IGHJ3 (Human) | ARGPLFHRLVYDFWSGYHDGFDM |  |
| IGHV3-53 (Human) | IGHJ6 (Human) | ARESYGMDV |  |
| IGHV3-30 (Human) | IGHJ4 (Human) | AKEGRPSDIVVVVAFDY |  |
| IGHV1-58 (Human) | IGHJ3 (Human) | AAPHCSGGSCYDAFDI |  |
| IGHV1-58 (Human) | IGHJ3 (Human) | AAPHCSGGSCYDAFDI |  |
| IGHV3-53 (Human) | IGHJ4 (Human) | AREGDVEGYYDFWSGYSRDRYYFDY |  |
| IGHV1-2 (Human) | IGHJ3 (Human) | ARDLGWSRLHGAFDI |  |
| IGHV4-4 (Human) | IGHJ4 (Human) | ARGLSYYPLYGSHINYIDY |  |
| IGHV3-53 (Human) | IGHJ3 (Human) | ARGGWYYDSSGYYSGRTDAFDI |  |
| IGHV4-4 (Human) | IGHJ4 (Human) | ARGLSYYPLYGSHINYIDY |  |
| IGHV3-66 (Human) | IGHJ4 (Human) | ARDLRDQDGYSYGAFDY |  |
| IGHV4-4 (Human) | IGHJ4 (Human) | ATHLRLGELRGVDY |  |
| IGHV3-21 (Human) | IGHJ4 (Human) | ARERGYYGGKTPPFL |  |
| IGHV1-69 (Human) | IGHJ6 (Human) | ARGNRLLYCSSTSCYLDAVRQGYYYYYYMDV |  |
| IGHV1-2 (Human) | IGHJ4 (Human) | ARAPPFYDFWSGIDY |  |
| IGHV1-69 (Human) | IGHJ5 (Human) | ARDLRNCSSTSCYYWFDP |  |
| IGHV1-69 (Human)+K3788 | IGHJ6 (Human) | ARGNRLLYCSSTSCYLDAVRQGYYYYYYMDV |  |
| IGHV1-2 (Human) | IGHJ6 (Human) | ASPASRGYSGYDHGYYYYMDV |  |
| IGHV1-2 (Human) | IGHJ6 (Human) | ARDDTLLRYSDWLPTTSFGGMDV |  |
| IGHV3-33 (Human) | IGHJ4 (Human) | AREDYYDSSGSLDY |  |
| IGHV6-1 (Human) | IGHJ4 (Human) | ARDPSSHFDY |  |
| IGHV3-53 (Human) | IGHJ3 (Human) | ARGGWYYDSSGYYSGRTDAFDI |  |
| IGHV3-66 (Human) | IGHJ6 (Human) | HRGSYSTFSYYYYMDV |  |
| IGHV1-58 (Human) | IGHJ3 (Human) | AAPHCSGGSCYDAFDI |  |
| IGHV1-2 (Human) | IGHJ3 (Human) | AREALGYGDFPHDGFDL |  |
| IGHV3-30-3 (Human) | IGHJ4 (Human) | ARGPRGYYDFWSGYENEYYFDY |  |
| IGHV1-46 (Human) | IGHJ6 (Human) | ARVGCGGDNCYFWRPLGNGMDV |  |
| IGHV3-66 (Human) | IGHJ6 (Human) | HRGSYSTFSYYYYMDV |  |
| IGHV4-59 (Human) | IGHJ5 (Human) | ARHCPWQQLVSNWFDP |  |
